# Dual vicinal functionalisation of heterocycles *via* an interrupted Pummerer coupling/[3,3]-sigmatropic rearrangement cascade†
†Electronic supplementary information (ESI) available: Full experimental details, NMR spectra, CCDC numbers for X-ray structures. CCDC 1570504, 1570698. For ESI and crystallographic data in CIF or other electronic format see DOI: 10.1039/c7sc04723a


**DOI:** 10.1039/c7sc04723a

**Published:** 2017-11-17

**Authors:** Mindaugas Šiaučiulis, Selma Sapmaz, Alexander P. Pulis, David J. Procter

**Affiliations:** a School of Chemistry , University of Manchester , Oxford Rd , Manchester , M13 9PL , UK . Email: david.j.procter@manchester.ac.uk; b Lilly Research Laboratories , Eli Lilly and Company Limited , Erl Wood Manor, Sunninghill Road , Windlesham , Surrey GU20 6PH , UK

## Abstract

A dual vicinal functionalisation cascade involving the union of heterocycles and allyl sulfoxides is described. In particular, the approach provides efficient one-step access to biologically relevant and synthetically important C3 thio, C2 carbo substituted indoles. The reaction operates under mild, metal free conditions and without directing groups, *via* an interrupted Pummerer coupling of activated allyl sulfoxides, generating allyl heteroaryl sulfonium salts that are predisposed to a charge accelerated [3,3]-sigmatropic rearrangement.

## Abstract 2

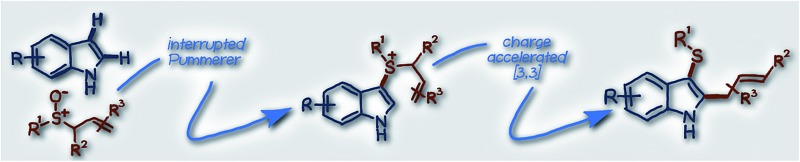
An interrupted Pummerer coupling/[3,3]-sigmatropic rearrangement cascade allows the direct and metal free dual vicinal functionalisation of heterocycles. For example, C3 thio, C2 allyl indoles are prepared in one synthetic operation from the union of the parent indoles and allyl sulfoxides.

## Introduction

**Scheme 1:**
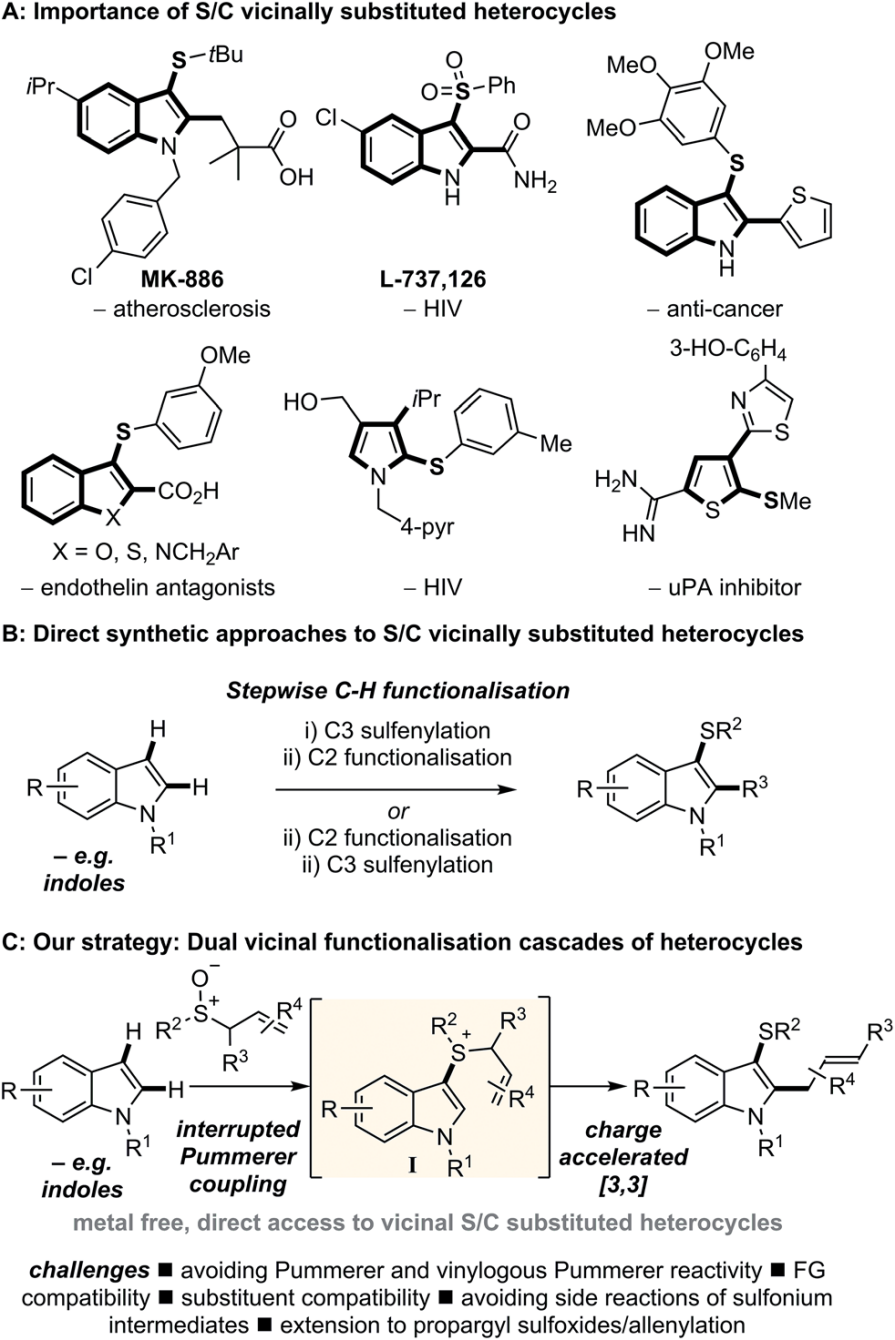
Vicinal S/C substituted heterocycles: important molecular architectures (A); stepwise C–H functionalisation strategy illustrated for indole (B); and, our approach utilising a dual vicinal functionalisation cascade, again, illustrated using indole.

Functionalised heterocycles constitute one of the most important families of molecules in chemistry. In particular, indoles are amongst the most common heterocyclic motifs found in biologically active compounds.^1^ Indoles that are substituted with sulfur at C3 and carbon at C2 display rich activity as biological probes,^2^ and in therapeutic areas such as atherosclerosis,^3^ HIV,^4^ and cancer^5^ amongst many others, and are also valuable building blocks for synthesis.^6^–^8^ Vicinal sulfur and carbon substitution has also proved to be of value in other heterocyclic systems (Scheme 1A).

Direct functionalisation of heterocycles is conceptually the most straightforward approach to decorated heterocycles and arguably the most attractive.^9^ For example, in considering indoles, the introduction of sulfur and carbon-containing groups by direct functionalisation^9^ has involved introduction of sulfur at C3 and a carbon-based group at C2 (or *vice versa*) in a stepwise fashion (Scheme 1B). Sulfenylation at C3 of indole is possible,^10^ however, direct regioselective introduction of a carbon based group at C2 requires highly basic organometallics^11^ or the use of expensive transition metals. Whilst transition metal catalysed regioselective C2 arylation, alkenylation^12^ and alkylation^13^ of indoles is possible, allylation at C2 requires superfluous directing groups.^14^ In addition, these stepwise approaches reduce overall process efficiency.^15^ An attractive alternative, direct functionalisation route to C3 thio, C2 carbo indoles would involve a cascade sequence, operating under mild and metal free conditions, in which the C3 sulfur and C2 carbo substituents are introduced in the same step *via* dual functionalisation of the readily accessible parent indole. However, a general process that constructs C3 thio, C2 carbo indoles in one synthetic operation from indoles was, until now, absent from the literature.

Herein, we report an efficient method for the construction of indoles bearing sulfur at C3 and a versatile allyl unit at C2, *via* dual vicinal functionalisation, enabled by an interrupted Pummerer coupling/[3,3]-sigmatropic rearrangement cascade (Scheme 1C).^16^,^17^ Importantly, the approach also extends to other important heterocycle classes. We employ readily accessible allylic sulfoxides that, once activated, are excellent coupling partners for the sulfenylation of various heterocycles. The generated heteroaryl–allyl sulfonium salt intermediates **I** are predisposed to a charge accelerated [3,3]-sigmatropic rearrangement and deliver useful allyl groups to heterocycles under metal free conditions. An analogous process uniting propargyl sulfoxides and indoles delivers sulfenylated/allenylated heterocycles.

## Results and discussion

### Dual vicinal functionalisation of indoles

We^18^ and others^19^ have previously shown that aryl allyl sulfonium salts, formed from aryl sulfoxides and allyl silanes, enable the ortho allylation of aryl sulfoxides.^20^,^21^ We postulated that, provided alternative Pummerer processes could be avoided, an interrupted Pummerer coupling^22^ of activated allyl sulfoxides with indoles, would allow direct access to indole–allyl sulfonium salts **I** that are predisposed to [3,3]-sigmatropic rearrangement and therefore C2 allylation, accomplishing the dual vicinal functionalisation cascade of indoles in one straightforward synthetic operation.^23^

**Scheme 2:**
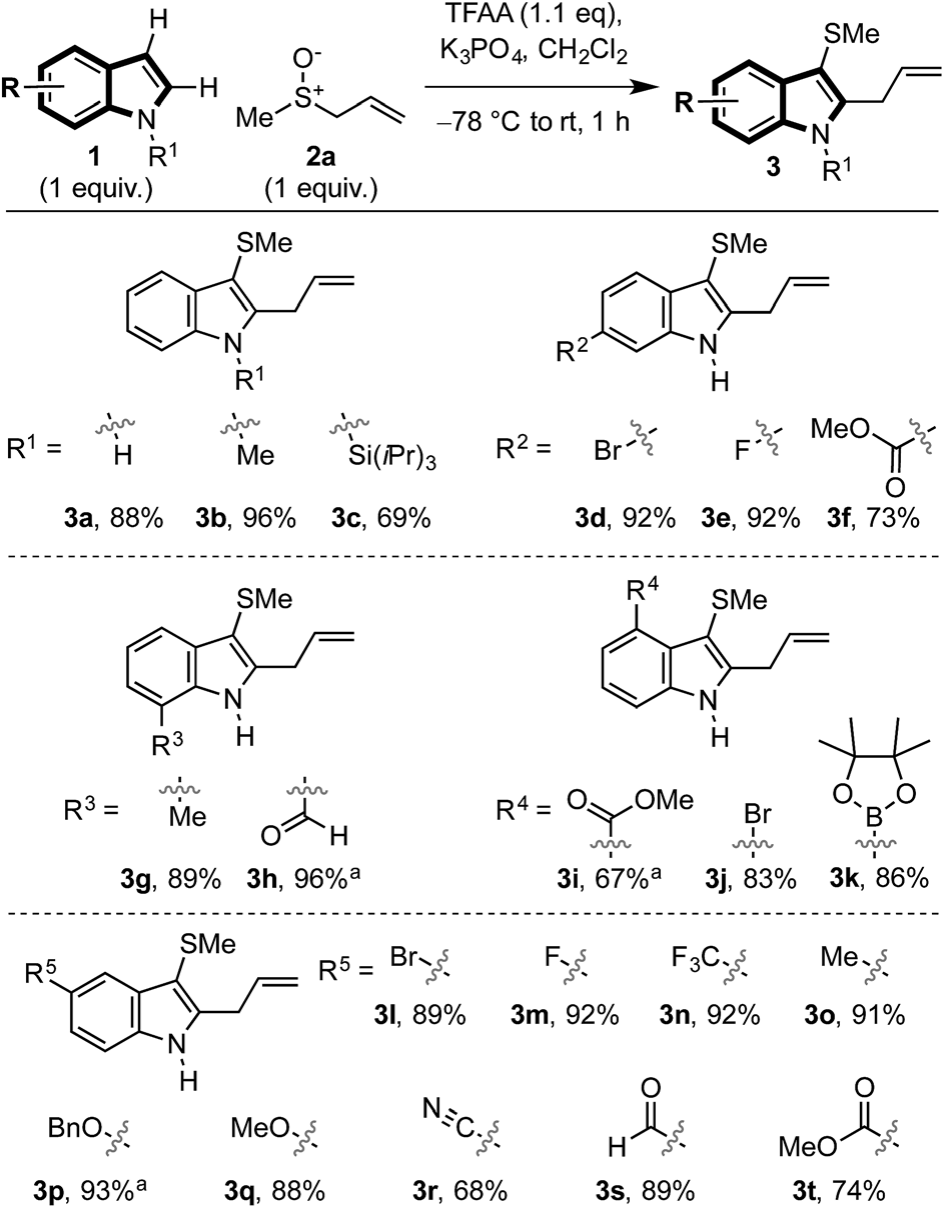
Exploring the scope of indoles in the dual vicinal functionalisation cascade. ^a^ 1.5 equivalents of sulfoxide and 1.6 equivalents of TFAA were used.

We began our investigation with unprotected N–H indole **1a** (R = R^1^ = H) and methyl allyl sulfoxide **2a** (Scheme 2). Upon treatment of sulfoxide **2a** with trifluoroacetic anhydride (TFAA) in the presence of indole (**1a**) and K_3_PO_4_, we were pleased to observe exclusive C3 sulfenylation. Addition of indole to the activated allyl sulfoxide exclusively occurred at sulfur and not at the α- or γ-carbons of the sulfoxonium salt (*cf.***II**, Scheme 5) as might be expected in the classic and vinylogous Pummerer reactions.^16^ The subsequent [3,3]-sigmatropic rearrangement of the generated sulfonium salt (*cf.***I**) was facile, and resulted in C2 allylation and formation of C3 thio, C2 allyl indole **3a** in high yield (88%).

In exploring the scope of the reaction, we found that *N*-methyl- (**3b**) and the more hindered *N*-triisopropyl silyl indole (**3c**) were also compatible with the process. Indoles bearing substituents at all positions of the benzo ring were also amenable to cascade difunctionalisation (**3d–t**), including those bearing versatile substituents such as halides (**3d**,**e**,**j**,**l**,**m**), esters (**3f**,**i**,**t**), aldehydes (**3h**,**s**), trifluoromethyl (**3n**), ether (**3p**,**q**), nitrile (**3r**) and boronic ester (**3k**). In all cases, the expected products were obtained in good to excellent yield (67–96%).

**Scheme 3:**
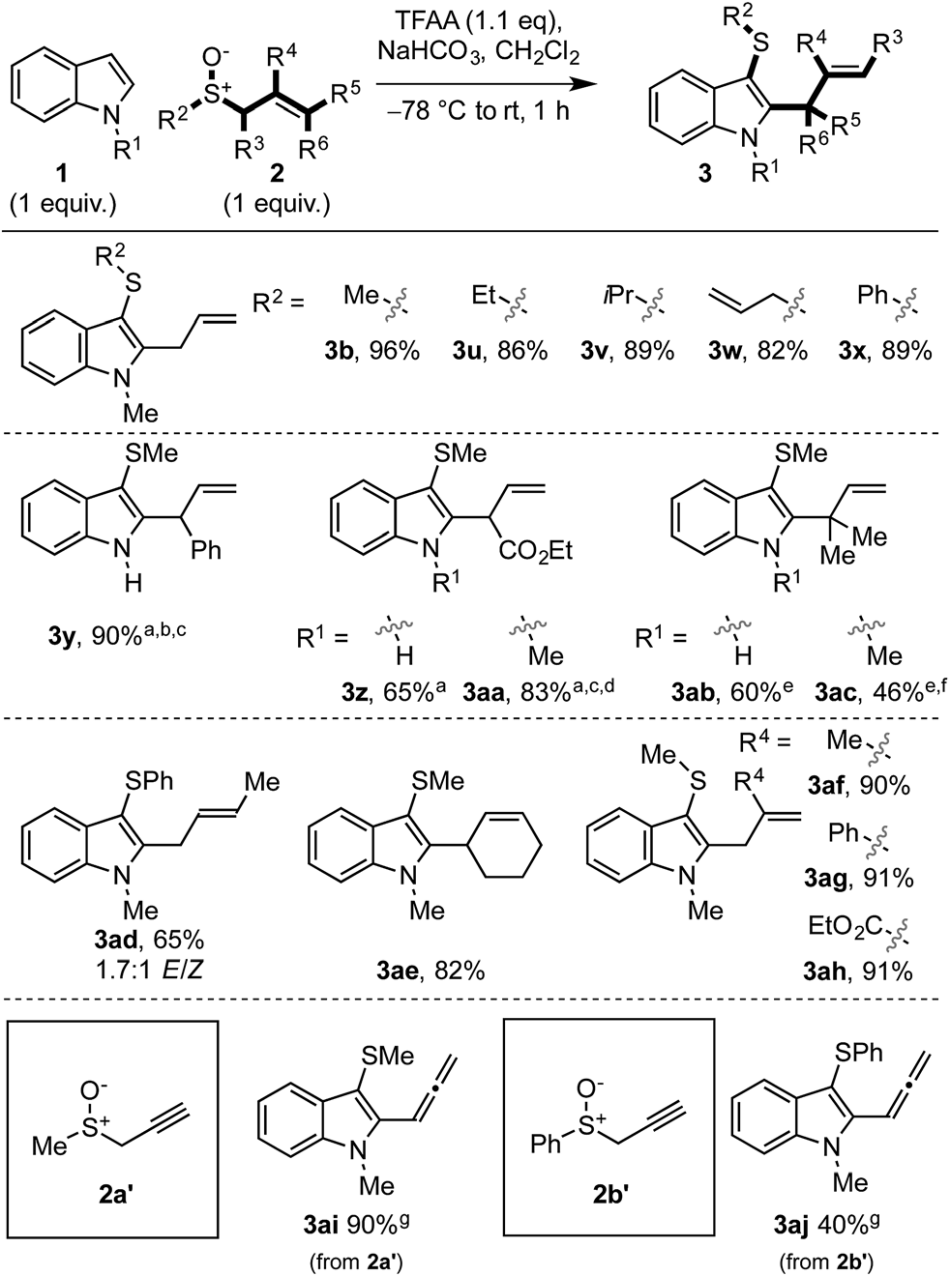
Exploring the scope of the allylic sulfoxide in the dual functionalisation cascade of indoles. ^a^ The corresponding *E*-allylic sulfoxides were used (*i.e.* R^6^ = H). ^b^**3y**/**4y** = 4.8 : 1. ^c^ Yield determined by ^1^H-NMR. ^d^**3aa**/**4aa** = 3 : 1. ^e^ Heated at 60 °C, 30 min. ^f^**3ac**/**4ac** = 3.3 : 1. ^g^ Heated at 60 °C, 1 h. Regioisomer ratios determined from ^1^H-NMR spectra of the crude reaction mixture.

We then examined the scope of allylic sulfoxide **2** in the dual functionalisation of indoles (Scheme 3). A variety of groups attached to sulfur of the allylic sulfoxide **2** were amenable to the process, such as alkyl (**3b**,**u–w**), including the more hindered isopropyl (**3v**), allyl (**3w**) and phenyl (**3x**). Interestingly, in the case of phenyl allyl sulfoxide (**3x**) where [3,3]-sigmatropic rearrangement of the intermediate sulfonium salt (*cf.***I**) might result in allylation of either indole or phenyl rings, allylation occurred exclusively on the indole moiety.

The substitution along the allyl portion of the allylic sulfoxide **2** was next investigated. When γ-substituted allyl sulfoxides were employed in the cascade, products **3y–ac** were obtained in high yield. In the cases using N–Me indole enroute to **3aa** and **3ac**, a minor regioisomeric product, C2 thio C3 allyl indole **4**, was also formed (see Scheme 5 and related discussion). Pleasingly, α- and β-substituted allyl sulfoxides gave exclusively the desired C3 thio, C2 allyl cascade products (*cf.***3ad–ah**). Due to the mild conditions employed, internalisation of the alkene was not observed in these or any other cases. Finally, the use of propargyl sulfoxides in the cascade sequence, in place of allyl sulfoxides, delivered allenylated indoles **3ai** and **3aj** in 90% and 40% yield, respectively. Notably, previously attempted Pummerer-type allenylations using aryl and heteroaryl sulfoxides and allenylsilanes had proved unsuccessful.21h,i

### Dual vicinal functionalisation of other aromatic heterocycles

**Scheme 4:**
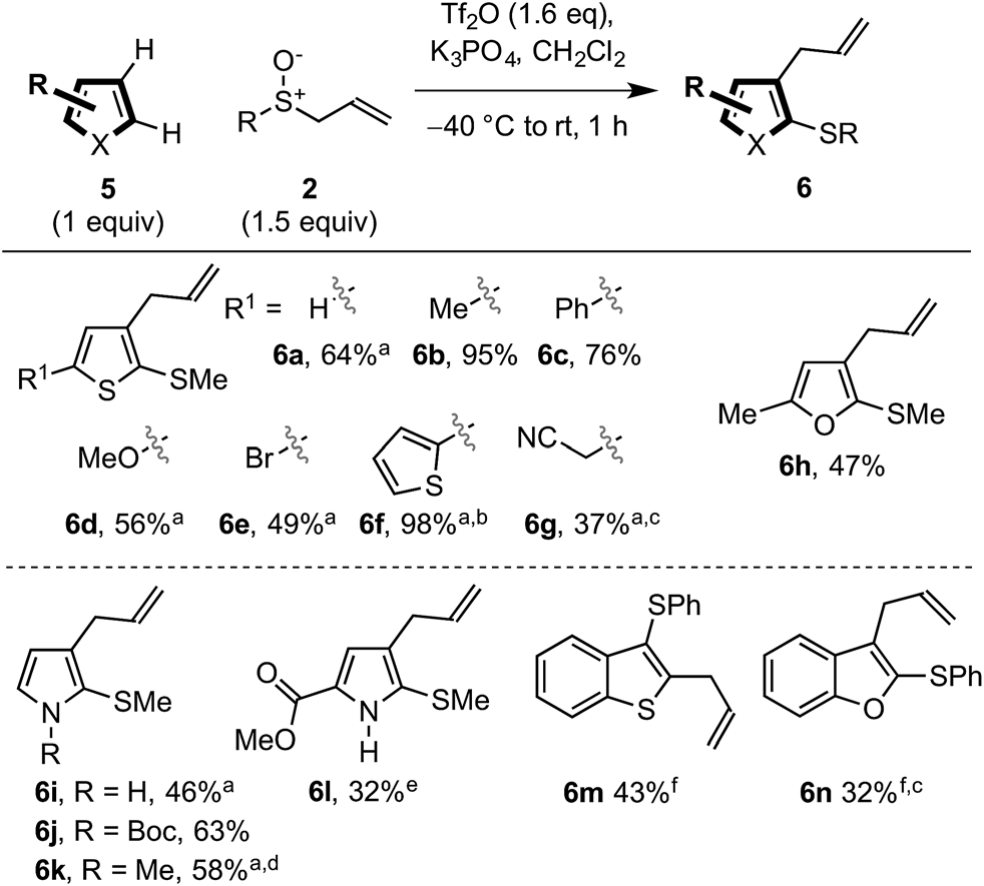
Preliminary results exploring other heterocycles in the dual vicinal functionalisation cascade. ^a^ using Na_2_CO_3_ instead of K_3_PO_4_. ^b^ 2 equivalents of sulfoxide and 2.2 equivalents of Tf_2_O were used. ^c^ Yield determined by ^1^H-NMR. ^d^**6k**/C3 thio, C2 allyl pyrrole = 1 : 1.6. ^e^**6l**/C3 thio, C2 allyl pyrrole = 1.5 : 1. ^f^ –78 °C to rt, overnight.

We also explored the dual vicinal functionalisation cascade of other important aromatic heterocycles14*a* since other S/C vicinally substituted systems also display biological activity (Scheme 1A)^24^ and our preliminary results are presented in Scheme 4. A variety of heterocycles including thiophene (**6a–g**), furan (**6h**) and pyrrole (**6i–l**) scaffolds, bearing various functionalities, afforded the desired C2 thio C3 allyl heterocycles. The use of triflic anhydride to activate the sulfoxide was necessary in these cases. In comparison with indole, the inherent nucleophilicity of the C2 position switched the regiochemistry of the initial sulfenylation to C2. However, pyrroles used in the formation of **6k** and **6l** also gave C3 thio, C2 allyl regioisomers. Finally, benzothiophene and benzofuran also underwent dual vicinal functionalisation (formation of **6m** and **6n**). As expected, compared to the analogous reactions of indoles, the processes were less efficient and showed decreasing selectivity for C3 sulfenylation. In fact, dual functionalisation of benzofuran gave the C2 thio C3 allyl isomer **6n** as the major product.^25^

### Proposed mechanism

**Scheme 5:**
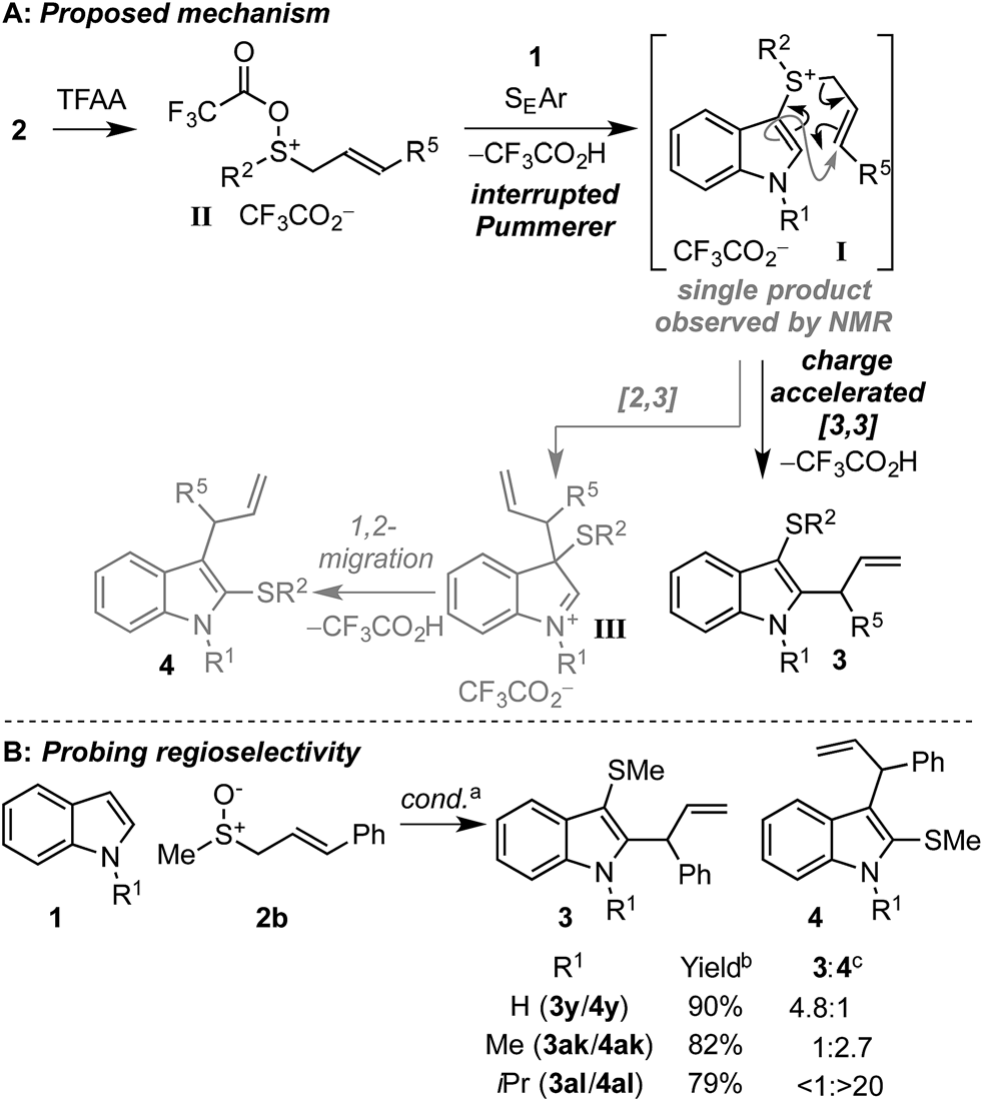
Proposed mechanism of the dual functionalisation cascade of indoles (A) and probing the effect of N-substitution for the selective generation of C3 thio, C2 allyl indole **3** or C2 thio, C3 allyl indole **4** (B). ^a^ Conditions as in Scheme 3. ^b^ Combined yield of **3** + **4**. ^c^ Regioisomer ratios determined by ^1^H-NMR of the crude reaction mixture.

Based on previous work^18^,^19^ and experimental observations, we propose the following mechanism for the dual functionalisation cascade (Scheme 5A). Activation of the allylic sulfoxide **2** with TFAA generates sulfoxonium salt **II**, which is trapped by indole through the inherently nucleophilic C3 position to form sulfonium salt **I**.22a–c Formation of sulfonium **I** is key to the prevention of over functionalisation since it is deactivated towards further electrophilic aromatic substitution, thus precluding further sulfenylation. In the case of sulfonium **I** generated enroute to **3z** and **3aa**, where the subsequent [3,3]-rearrangement was slow, a single C3 indolyl sulfonium product was observed by ^1^H-NMR (*i.e.* C2 sulfonium salts were not observed). Subsequent charge accelerated [3,3]-sigmatropic rearrangement^17^–^21^ in **I** accomplishes the second functionalisation event and delivers the desired C3 thio, C2 allyl indoles **3**.

In some cases where γ-substituted allyl sulfoxides **2** were employed, the C2 thio, C3 allyl indoles **4** were also formed.^26^ We propose that in these cases, the [3,3]-sigmatropic rearrangement is disfavoured on steric grounds (*vide infra*) and instead a [2,3]-sigmatropic rearrangement of allyl sulfonium salt **I**^27^ forms intermediate **III**, which then undergoes facile sulfur migration to C2, likely *via* an episulfonium ion intermediate.^28^ Since C2 thio, C3 carbo indoles also have interesting biological activity, perhaps most notably in the amatoxins and phallotoxins found in several poisonous mushrooms,^29^ we further investigated this interesting facet of the method. Postulating that varying the sterics at nitrogen of indole would affect the relative rates of [3,3] and [2,3] rearrangements leading to different ratios of **3** and **4** respectively, we studied the reactivity of N–H, N–Me, and N–iPr indoles with γ-phenyl substituted allyl sulfoxide **2b** (Scheme 5B). Pleasingly, when N–H indole **1a** was employed, the selectivity for **3y** was significantly increased (**3y**/**4y** = 4.8 : 1) relative to N–Me indole (**3ak**/**4ak** = 1 : 2.7). The trend of increased selectivity upon switching to the less sterically encumbered N–H indole **1a** was also observed with γ-CO_2_Et and γ-dimethyl allyl sulfoxides, where the C3 thio C2 allyl regioisomers **3** were formed exclusively in both cases (*cf.***3z***vs.***3aa**, and **3ab***vs.***3ac**, Scheme 3). Interestingly, upon utilising the more hindered N–iPr indole, C2 thio C3 allyl regioisomer **4al** was obtained as the sole regioisomeric product.^30^

### Scalability and iterative functionalisation

**Scheme 6:**
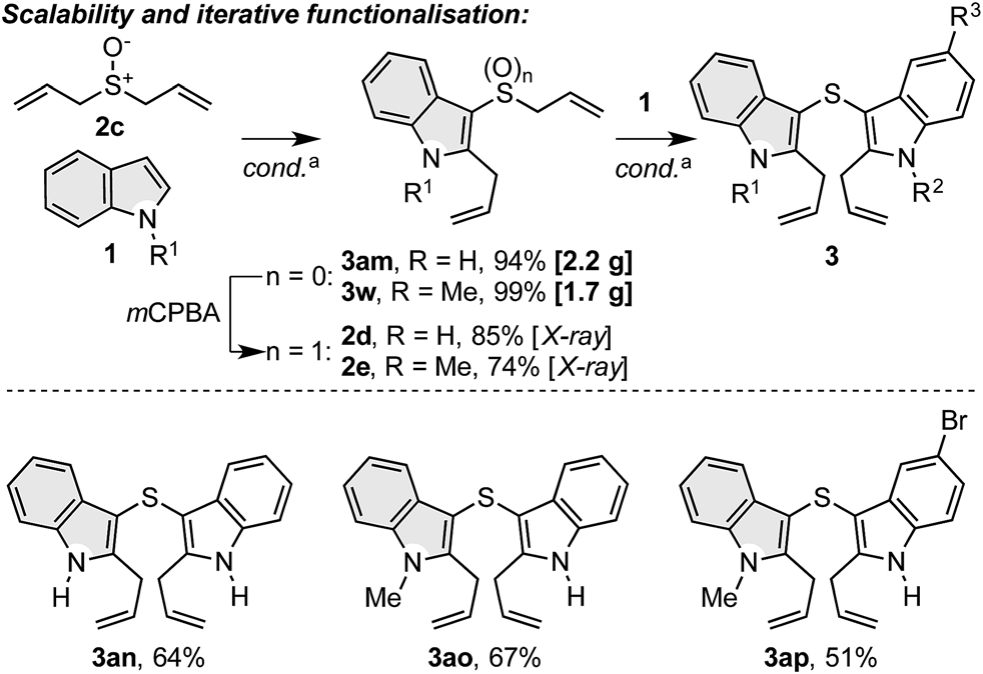
Scalability of the dual functionalisation cascade of indoles and iterative dual functionalisation cascades for the generation of novel bis indolyl sulfides. ^a^ Conditions as in Scheme 2.

To further explore the synthetic capability of the dual functionalisation cascade products,^31^ we successfully accomplished gram scale reactions between diallyl sulfoxide (**2c**) and indoles **1a** and **1b**, which gave **3am** (2.2 g) and **3w** (1.7 g) in excellent yield (Scheme 6). In addition, products **3am** and **3w** were readily oxidised to indole–allyl sulfoxides **2d** and **2e**, which served as allyl sulfoxide units in the dual functionalisation cascade with other indoles to produce novel symmetrical (**3an**) and unsymmetrical bis indolyl sulfides (**3ao**, **3ap**), a class of compounds that display antioxidant activity.^32^

## Conclusions

In summary, we have described a dual vicinal functionalisation cascade of indoles that, without the need for superfluous directing groups or metals, provides efficient access to biologically relevant C3 thio, C2 carbo indoles under mild conditions. The process has also been applied to other heterocycles and to propargyl sulfoxide partners. The reaction operates *via* an interrupted Pummerer coupling between an activated sulfoxide partner and a heteroaromatic nucleophile, where the generated sulfonium salt formed then undergoes facile charge accelerated [3,3]-sigmatropic rearrangement and accomplishes the vicinal difunctionalisation of heterocycles in a single straightforward synthetic operation.

## Conflicts of interest

There are no conflicts to declare.

## Supplementary Material

Supplementary information

Crystal structure data
