# Supplementary material for: Dual vicinal functionalisation of heterocycles via an interrupted Pummerer coupling/[3,3]-sigmatropic rearrangement cascade
Source: Chem Sci. 2017 Nov 17;9(3):754–9. doi: 10.1039/c7sc04723a (PMC5870476; doi:10.1039/c7sc04723a)
Supplement: Supplementary file 1 [file SC-009-C7SC04723A-s001.pdf]

Electronic Supplementary Material (ESI) for Chemical Science.
This journal is © The Royal Society of Chemistry 2017

Table of Contents

General Experimental........2

Assignment of C2/C3 regioselectivity in products........2

Experimental data........3

General procedure A: Synthesis of allylic sulfoxides by oxidation of sulfides........3

General procedure B: Synthesis of allylic sulfoxides from allyl halides.........3

General procedure C: Dual vicinal functionalisation of indoles using a range of allylic sulfoxides:........9

General procedure D: Dual vicinal functionalisation of a range of indoles:........19

General procedure E: Dual vicinal functionalisation of other heteroaromatics:........26

^{1}H and ^{13}C NMR spectra of compounds........34

X-ray structures........108

References........110

General Experimental

All experiments were performed under an atmosphere of nitrogen, using anhydrous solvents. Dichloromethane was distilled from CaH_2 before use or commercial anhydrous dichloromethane was used. All other reagents were purchased from commercial sources and used as supplied unless otherwise noted. Routine TLC analysis was performed on aluminium backed silica gel 60 F254, visualised by UV lamp and $KMnO_4$ stain. Column chromatography was performed using 35 – 70 µ, 60A silica gel.

^{1}H NMR spectra were recorded at 400 or 500 MHz, ^{13}C NMR spectra were recorded at 101 or 125 MHz, ^{19}F NMR spectra were recorded at 376 MHz. All chemical shifts are reported in ppm, and referenced to $CDCl_3$. NMR yields were determined using 0.7 mL of 0.119 M solution (0.0833 mmol) of $MeNO_2$ as internal standard.

High resolution mass spectra were obtained using either APCI (atmospheric pressure chemical ionisation) or ESI (electrospray ionisation). Infra-red spectra were recorded neat as evaporated films or solids using an ATR FT/IR spectrometer. Melting points are uncorrected and measured on solids obtained after chromatography.

Assignment of C2/C3 regioselectivity in products

C2/C3 regioselectivity was confirmed by ^{1}H-^{13}C HMBC correlations between SR (R = alkyl) and C3/C2 or between $CH_2C\mathit{H}{=}CH_2$ and C3/C2 (R = Ph). Literature reported characteristic C3/C2 ^{13}C NMR chemical shifts,[1] ^{1}H-^{13}C HMBC correlations between NR (R = alkyl) and C2, and X-ray structures were used to confirm assignment of ^{13}C NMR chemical shifts corresponding to C3/C2. For details see included ^{1}H-^{13}C HMBC spectra.

Experimental data

General procedure A: Synthesis of allylic sulfoxides by oxidation of sulfides

A solution of sulfide (1.00 eq.) in CH_2Cl_2 (0.1 M) was cooled to 0 °C, and *m*CPBA (1.05 eq.) was added in portions. The reaction mixture was stirred for 30 min, allowed to warm up to room temperature and stirred for 1 h. To the reaction was added aqueous NaOH (0.5 M, 3.0 eq.), and the organic phase was seperated and washed with NaOH (0.5 M, 3.0 eq x 2). The combined aqueous washings were extracted with $CHCl_3$/*i*-PrOH (3:1) and combined organic extracts were dried over $NaSO_4$, filtered, and solvent was removed *in vacuo*. The crude product was purified by column chromatography.

General procedure B: Synthesis of allylic sulfoxides from allyl halides.

To a suspension of sodium thiolate (1.0 eq.) in THF (0.1 M) at 0 °C was added allylic bromide (1.1 eq.) dropwise. The reaction mixture was allowed to warm up to room temperature and stirred for 24 h. The reaction mixture was then cooled to 0 °C, *m*CPBA (1.0 eq.) was added in portions, and the reaction mixture was stirred for 3 h. The reaction was quenched by addition of sat. $NaHCO_3$ (aq.) and CH_2Cl_2 was added until the phases seperated. The aqueous layer was then washed (CH_2Cl_2 x 3), the organic extracts were combined, washed with brine, and dried over $MgSO_4$. The solvent was removed *in vacuo* and the crude product was purified by column chromatography.

3-(Methylsulfinyl)prop-1-ene (2a)

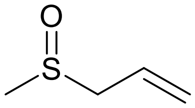

Following general procedure A using methyl allyl sulfide (4.40 mL, 40.0 mmol) and *m*CPBA (9.41 g, 77% wt. 42.0 mmol) afforded the title compound as a pale yellow oil (3.05 g, 73%).

^{1}H NMR (400 MHz, Chloroform-*d*) δ 5.96 – 5.75 (m, 1H, $CH_2CH{=}CH_2$), 5.43 (ddd, *J* = 10.2, 1.4, 0.7 Hz, 1H, $CH_2CH{=}CH_2$), 5.37 (dt, *J* = 17.0, 1.3 Hz, 1H, $CH_2CH{=}CH_2$), 3.47 (ddd, *J* = 13.0, 7.5, 1.1 Hz, 1H, $CH_2CH{=}CH_2$), 3.44 – 3.37 (m, 1H, $CH_2CH{=}CH_2$), 2.53 (s, 3H, SCH_3). **^{13}C NMR** (101 MHz, $CDCl_3$) δ 125.7 ($CH_2CH{=}CH_2$), 123.9 ($CH_2CH{=}CH_2$), 58.0 ($CH_2CH{=}CH_2$), 37.4 (SCH_3). **ν_{max}** (neat)/ cm^{-1} 3459, 1635, 1421, 1301, 1029, 930. **HRMS** (ESI) calculated for C_4H_8SONa ($M+Na^+$): 127.0189, found 127.0188.

(*E*)-(3-(Methylsulfinyl)prop-1-en-1-yl)benzene (2b)

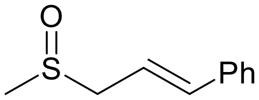

Following general procedure B using sodium methanethiolate (840 mg, 12.0 mmol), 3-phenylallyl bromide (2.60 g, 13.2 mmol), and *m*CPBA (2.68 g, 77% wt., 12.0 mmol) afforded the title compound as a white solid (1.10 g, 50%).

^{1}H NMR (400 MHz, Chloroform-*d*) δ 7.43 – 7.38 (m, 2H, Ar-*H* x 2), 7.37 – 7.31 (m, 2H, Ar-*H* x 2), 7.31 – 7.25 (m, 1H, Ar-*H*), 6.68 (dt, *J* = 15.8, 1.2 Hz, 1H, $CH_2CH{=}CHPh$), 6.26 (dt, *J* = 15.7, 7.7 Hz, 1H, , $CH_2CH{=}CHPh$), 3.67

(ddd, *J* = 13.0, 7.6, 1.2 Hz, 1H, C*H*$_2$CH=CHPh), 3.61 (ddd, *J* = 12.9, 7.8, 1.2 Hz, 1H, C*H*$_2$CH=CHPh), 2.59 (s, 3H, SC*H*$_3$). **^{13}C NMR** (101 MHz, CDCl$_3$) δ 138.4 (CH$_2$CH=*C*H), 135.9 (*C*$_q$), 128.8 (*C*H x 2), 128.5 (*C*H), 126.7 (*C*H *x 2*), 116.4 (CH$_2$*C*H=CH), 57.7 (*C*H$_2$CH=CH), 37.4 (S*C*H$_3$). **ν$_{max}$** (neat)/ cm^{-1} 3033, 2909, 1491, 1452, 1409, 1020, 968. **HRMS** (ESI) calculated for $C_{10}H_{12}OSNa$ (M+Na$^+$): 203.0501, found 203.0497.

3-(Allylsulfinyl)prop-1-ene (2c)

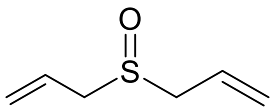

Following general procedure A using diallyl sulfoxide (5.10 mL, 40 mmol) and mCPBA (9.41 g, 77% wt., 42.0 mmol) afforded the title compound as a colourless oil (4.69 g, 90%).

^{1}H NMR (400 MHz, Chloroform-*d*) δ 5.89 (ddt, *J* = 17.3, 10.3, 7.5 Hz, 2H, CH$_2$C*H*=CH$_2$ x 2), 5.45 (ddt, *J* = 10.3, 1.3, 1.0 Hz, 2H, CH$_2$CH=C*H*$_2$), 5.39 (dq, *J* = 17.3, 1.3 Hz, 2H, CH$_2$CH=C*H*$_2$), 3.54 (dt, *J* = 7.5, 1.0 Hz, 1H, C*H*$_2$CH=CH$_2$), 3.51 (dt, *J* = 7.5, 1.0 Hz, 1H, C*H*$_2$CH=CH$_2$), 3.41 (dt, *J* = 7.5, 1.0 Hz, 1H, C*H*$_2$CH=CH$_2$), 3.38 (dt, *J* = 7.5, 1.0 Hz, 1H, C*H*$_2$CH=CH$_2$). **^{13}C NMR** (101 MHz, CDCl$_3$) δ 125.9 (CH$_2$CH=*C*H$_2$), 123.8 (CH$_2$*C*H=CH$_2$), 54.4 (*C*H$_2$CH=CH$_2$). **ν$_{max}$** (neat)/ cm^{-1} 3082, 3015, 2979, 2961, 2915, 1635, 1033. **HRMS** (ESI) calculated for $C_6H_{11}SO$ (M+H$^+$): 131.0525, found 131.0525.

3-(Ethylsulfinyl)prop-1-ene (SI-2f)

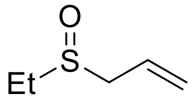

Following general procedure B using sodium ethanethiolate (20.0 mmol) (prepared from sodium hydride (800 mg, 60% in mineral oil, 20.0 mmol) and ethanethiol (1.44 mL, 20.0 mmol)), allyl bromide (1.90 mL, 22.0 mmol), and *m*CPBA (4.48 g, 77% wt., 20.0 mmol) afforded the title compound as a pale yellow oil (1.16 g, 49%).

^{1}H NMR (400 MHz, Chloroform-*d*) δ 5.85 (ddt, *J* = 17.0, 10.2, 7.5 Hz, 1H, CH$_2$C*H*=CH$_2$), 5.39 (ddt, *J* = 10.2, 1.4, 0.8 Hz, 1H, CH$_2$CH=C*H*$_2$), 5.35 (dq, *J* = 17.0, 1.3 Hz, 1H, CH$_2$CH=C*H*$_2$), 3.46 (dddd, *J* = 13.0, 7.4, 1.2, 0.7 Hz, 1H, C*H*$_2$CH=CH$_2$), 3.42 – 3.32 (m, 1H, C*H*$_2$CH=CH$_2$), 2.77 – 2.69 (m, 1H, C*H*$_2$CH$_3$), 2.68 – 2.60 (m, 1H, C*H*$_2$CH$_3$), 1.29 (t, *J* = 7.5 Hz, 3H, CH$_2$C*H*$_3$). **^{13}C NMR** (101 MHz, CDCl$_3$) δ 125.8 (CH$_2$CH=*C*H$_2$), 123.5 (CH$_2$*C*H=CH$_2$), 55.1 (*C*H$_2$CH=CH$_2$), 44.2 (*C*H$_2$CH$_3$), 6.8 (CH$_2$*C*H$_3$). **ν$_{max}$** (neat)/ cm^{-1} 3459, 2976, 1636, 1039, 1014, 928. **HRMS** (ESI) calculated for $C_5H_{10}OSNa$ (M+Na$^+$): 141.0345, found 141.0342.

3-(Isopropylsulfinyl)prop-1-ene (SI-2g)

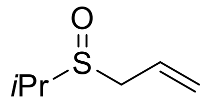

Following general procedure B using sodium *iso*propylthiolate (20.0 mmol) (prepared from sodium hydride (800 mg, 60% in mineral oil, 20.0 mmol) and *iso*propanethiol (1.85 mL, 20.0 mmol)), allyl bromide (1.90 mL, 22.0 mmol), and *m*CPBA (4.48 g, 77% wt., 20.0 mmol) afforded the title compound as a pale yellow oil (1.53 g, 58%).

^{1}H NMR (400 MHz, Chloroform-*d*) δ 5.87 (ddt, *J* = 17.0, 10.2, 7.5 Hz, 1H, CH$_2$C*H*=CH$_2$), 5.41 – 5.31 (m, 2H, CH$_2$CH=C*H*$_2$), 3.41 (ddt, *J* = 13.2, 7.3, 1.1 Hz, 1H, C*H*$_2$CH=CH$_2$), 3.32 (ddt, *J* = 13.2, 7.7, 1.0 Hz, 1H, C*H*$_2$CH=CH$_2$), 2.80 (hept, *J* = 6.9 Hz, 1H, C*H*(CH$_3$)$_2$), 1.28 (d, *J* = 6.9 Hz, 3H, CH(C*H*$_3$)$_2$), 1.22 (d, *J* = 6.9 Hz, 3H, CH(C*H*$_3$)$_2$). **^{13}C NMR** (101 MHz, CDCl$_3$) δ 126.3 (CH$_2$*C*H=CH$_2$), 123.1 (CH$_2$CH=*C*H$_2$), 52.6 (*C*H$_2$CH=CH$_2$), 48.6

(*C*H(CH_3)$_2$), 16.4 (CH(*C*H_3)$_2$), 14.4 (CH(*C*H_3)$_2$). **ν_{max}** (neat)/ cm^{-1} 3459, 2966, 1463, 1017, 927. **HRMS** (ESI) calculated for $C_6H_{12}OSNa$ ($M+Na^+$): 155.0501, found 155.0498.

(Allylsulfinyl)benzene (SI-2h)

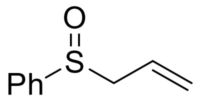

Following general procedure A using phenyl allyl sulfide (4.40 mL, 30.0 mmol) and *m*CPBA (7.06 g, 77% wt., 31.5 mmol) afforded the title compound as a pale yellow oil (4.52 g, 91%).

^{1}H NMR (400 MHz, Chloroform-*d*) δ 7.61 – 7.54 (m, 2H, Ar-*H* x 2), 7.52 – 7.43 (m, 3H, Ar-*H* x 3), 5.71 – 5.52 (m, 1H, CH_2C*H*=CH_2), 5.30 (d, *J* = 10.2 Hz, 1H, CH_2CH=C*H*$_2$), 5.16 (dq, *J* = 17.0, 1.3 Hz, 1H, CH_2CH=C*H*$_2$), 3.54 (dd, *J* = 12.8, 7.5 Hz, 1H, C*H*$_2$CH=CH_2), 3.47 (dd, *J* = 12.8, 7.5 Hz, 1H, C*H*$_2$CH=CH_2). **^{13}C NMR** (101 MHz, $CDCl_3$) δ 143.0 (*C*$_q$), 131.2 (*C*H), 129.1 (*C*H x 2), 125.3 (CH_2*C*H=CH_2), 124.4 (*C*H x 2), 124.0 (CH_2CH=*C*H_2), 60.9 (*C*H_2CH=CH_2). **ν_{max}** (neat)/ cm^{-1} 3463, 3057, 1443, 1088, 1037, 996, 927. **HRMS** (ESI) calculated for $C_9H_{10}OSNa$ ($M+Na^+$): 189.0345, found 189.0341.

3-(Methylsulfinyl)cyclohex-1-ene (SI-2i)

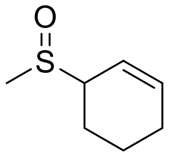

Following general procedure B using sodium methanethiolate (350 mg, 5.00 mmol), 3-bromocyclohexene (0.633 mL, 5.50 mmol), and *m*CPBA (1.12 g, 77% wt., 5.00 mmol) afforded the title compound as a pale yellow oil (523 mg, 73%) as a mixture of diastereoisomers (1:1).

^{1}H NMR (400 MHz, Chloroform-*d*) δ 6.14 (dtd, *J* = 9.9, 3.8, 2.0 Hz, 1H, CHC*H*=CH), 6.09 (dtd, *J* = 10.1, 3.8, 1.5 Hz, 1H, CHC*H*=CH), 5.84 (dq, *J* = 10.2, 2.5 Hz, 1H, CHCH=C*H*), 5.57 (ddtd, *J* = 9.7, 4.3, 2.1, 0.7 Hz, 1H CHCH=C*H*), 3.37 – 3.29 (m, 1H, C*H*CH=CH), 3.25 (dqt, *J* = 6.4, 4.1, 1.8 Hz, 1H, C*H*CH=CH), 2.55 (s, 3H, SC*H*$_3$), 2.54 (s, 3H, SC*H*$_3$), 2.13 – 2.05 (m, 4H, C*H*$_2$ x 2), 2.02 – 1.56 (m, 8H, C*H*$_2$ x 4). **^{13}C NMR** (101 MHz, $CDCl_3$) δ 135.8 (CH*C*H=CH), 134.5 (CH*C*H=CH), 120.0 (CHCH=*C*H), 119.4 (CHCH=*C*H), 60.7 (*C*HCH=CH), 58.6 (*C*HCH=CH), 35.9 (S*C*H_3), 35.5 (S*C*H_3), 25.1 (*C*H_2), 24.9 (*C*H_2), 22.7 (*C*H_2), 21.6 (*C*H_2), 20.4 (*C*H_2), 19.0 (*C*H_2). **ν_{max}** (neat)/ cm^{-1} 3029, 2994, 2928, 2861, 2834, 1644, 1031. **HRMS** (ESI) calculated for $C_7H_{12}SONa$ ($M+Na^+$): 167.0501, found 167.0492.

Ethyl (*E*)-4-(methylsulfinyl)but-2-enoate (SI-2j)

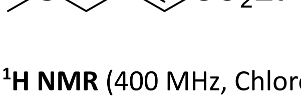

Following general procedure B using sodium methanethiolate (350 mg, 5.00 mmol), ethyl-4-bromocrotonate (757 µL, 5.50 mmol), and *m*CPBA (1.12 g, 77% wt. 5.00 mmol) afforded the title compound as a pale yellow oil (0.360 g, 41%).

^{1}H NMR (400 MHz, Chloroform-*d*) δ 6.93 (dt, *J* = 15.7, 7.9 Hz, 1H, CH_2C*H*=CH), 6.09 (dt, *J* = 15.6, 1.3 Hz, 1H, CH_2CH=C*H*), 4.20 (q, *J* = 7.1 Hz, 2H, OC*H*$_2$$CH_3$), 3.61 (ddd, *J* = 13.0, 7.7, 1.3 Hz, 1H, C*H*$_2$CH=CH), 3.53 (ddd, *J* = 12.9, 8.0, 1.3 Hz, 1H, C*H*$_2$CH=CH), 2.59 (s, 3H, SC*H*$_3$), 1.28 (t, *J* = 7.2 Hz, 3H, OCH_2C*H*$_3$). **^{13}C NMR** (101 MHz, $CDCl_3$) δ 165.2 (*C*O_2Et), 134.5 (CH_2*C*H=CH), 129.0 (CH_2CH=*C*H), 61.0 (O*C*$H_2$$CH_3$), 56.2 (*C*$H_2$CH=CH), 38.0 (S*C*$H_3$), 14.3 (O$CH_2$*C*$H_3$). **$\nu_{max}$** (neat)/ cm^{-1} 2983, 2937, 2916, 1711, 1651, 1196, 1036**.** **HRMS** (ESI) calculated for $C_7H_{12}SO_3Na$ ($M+Na^+$): 199.0399, found 199.0388.

3-Methyl-1-(methylsulfinyl)but-2-ene (SI-2k)

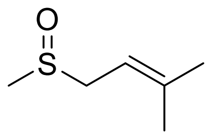

Following general procedure B using sodium methanethiolate (840 mg, 12.0 mmol), 3,3-dimethylallyl bromide (1.52 mL, 13.2 mmol) and *m*CPBA (2.68 g, 77% wt., 12.0 mmol) afforded the title compound as a yellow oil (699 mg, 43%).

^{1}H NMR (400 MHz, Chloroform-*d*) δ 5.22 (ddq, *J* = 9.4, 6.6, 1.4 Hz, 1H, CH_2CH=C), 3.50 (dd, *J* = 13.1, 7.9 Hz, 1H, CH_2CH=C), 3.44 – 3.31 (m, 1H, CH_2CH=C), 2.50 (s, 3H, SCH_3), 1.79 (q, *J* = 1.0 Hz, 3H, $C(CH_3)_2$), 1.71 (d, *J* = 1.4 Hz, 3H, $C(CH_3)_2$). **^{13}C NMR** (101 MHz, $CDCl_3$) δ 142.0 (CH_2CH=*C*), 111.3 (CH_2*C*H=C), 53.6 (*C*H_2CH=C), 37.4 (SCH_3), 26.1 ($C(CH_3)_2$), 18.7 ($C(CH_3)_2$). **ν_{max}** (neat)/ cm^{-1} 3441, 2970, 2914, 1666, 1424, 1037, 964. **HRMS** (ESI) calculated for $C_6H_{12}OSNa$ ($M+Na^+$): 155.0501, found 155.0497.

2-Methyl-3-(methylsulfinyl)prop-1-ene (SI-2l)

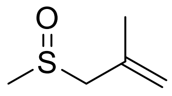

Following general procedure B using sodium methanethiolate (840 mg, 12.0 mmol), 3-bromo-2-methylpropene (1.33 mL, 13.2 mmol), and *m*CPBA (2.68 g, 77% wt., 12.0 mmol) afforded the title compound as a pale yellow oil (501 mg, 35%).

^{1}H NMR (400 MHz, Chloroform-*d*) δ 5.08 (p, *J* = 1.5 Hz, 1H, $CH_2C(Me)=CH_2$), 5.00 (dq, *J* = 1.5, 1.0 Hz, 1H, $CH_2C(Me)=CH_2$), 3.47 (dd, *J* = 12.4, 1.0 Hz, 1H, $CH_2C(CH_3)=CH_2$), 3.35 (dd, *J* = 12.5, 1.0 Hz, 1H, $CH_2C(Me)=CH_2$), 2.56 (s, 3H, SCH_3), 1.87 (dd, *J* = 1.5, 1.0 Hz, 3H, $CH_2C(CH_3)=CH_2$). **^{13}C NMR** (101 MHz, $CDCl_3$) δ 135.8 ($CH_2C(CH_3)=CH_2$), 118.3 ($CH_2C(CH_3)=CH_2$), 63.8 ($CH_2C(CH_3)=CH_2$), 38.3 (SCH_3), 23.1 ($CH_2C(CH_3)=CH_2$). **ν_{max}** (neat)/ cm^{-1} 3460, 2974, 2913, 1651, 1424, 1038, 899. **HRMS** (ESI) calculated for $C_5H_{10}OSNa$ ($M+Na^+$): 141.0345, found 141.0342.

(3-(Methylsulfinyl)prop-1-en-2-yl)benzene (SI-2m)

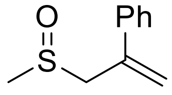

Following general procedure B using sodium methanethiolate (393 mg, 5.60 mmol), 3-bromo-2-phenylpropene (1.21 g, 6.16 mmol), and *m*CPBA (1.26 g, 77% wt., 5.60 mmol) afforded the title compound as a pale yellow oil (735 mg, 73%).

^{1}H NMR (400 MHz, Chloroform-*d*) δ 7.50 – 7.42 (m, 2H, Ar-*H* x 2), 7.42 – 7.29 (m, 3H, Ar-*H* x 3), 5.66 (q, *J* = 0.8 Hz, 1H, $CH_2C(Ph)=CH_2$), 5.40 (q, *J* = 0.8 Hz, 1H, $CH_2C(Ph)=CH_2$), 4.06 (dt, *J* = 12.8, 0.8 Hz, 1H, $CH_2C(Ph)=CH_2$), 3.79 (dt, *J* = 12.8, 0.8 Hz, 1H, $CH_2C(Ph)=CH_2$), 2.53 (s, 3H, SCH_3). **^{13}C NMR** (101 MHz, $CDCl_3$) δ 138.9 (C_q), 138.2 (C_q), 128.9 (*C*H), 128.7 (*C*H), 126.2 (*C*H), 119.5 ($CH_2C(Ph)=CH_2$), 61.5 ($CH_2C(Ph)=CH_2$), 38.4 (SCH_3). **ν_{max}** (neat)/ cm^{-1} 3071, 3025, 2990, 2956, 2913, 1495, 1449, 1408, 1026. **HRMS** (ESI) calculated for $C_{10}H_{12}SONa$ ($M+Na^+$): 203.0501, found 203.0488.

Ethyl 2-((methylsulfinyl)methyl)acrylate (SI-2n)

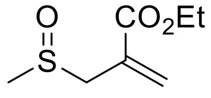

Following general procedure B using sodium methanethiolate (300 mg, 4.28 mmol), ethyl-2-(bromomethyl)acrylate (650 µL, 4.71 mmol), and *m*CPBA (959 mg, 77% wt., 4.28 mmol) afforded the title compound as a pale yellow oil (349 mg, 46%).

^{1}H NMR (400 MHz, Chloroform-*d*) δ 6.52 (d, *J* = 1.0 Hz, 1H, $CH_2C=CH_2$), 5.96 (q, *J* = 1.0 Hz, 1H, $CH_2C=CH_2$), 4.25 (q, *J* = 7.1 Hz, 2H, OCH_2CH_3), 3.75 (dd, *J* = 12.7, 1.0 Hz, 1H, $CH_2C=CH_2$), 3.62 (dd, *J* = 12.7, 1.0 Hz, 1H, $CH_2C=CH_2$), 2.55 (s, 3H, SCH_3), 1.31 (t, *J* = 7.1 Hz, 3H, OCH_2CH_3). **^{13}C NMR** (101 MHz, $CDCl_3$) δ 165.8 (CO_2Et), 132.2 ($CH_2C=CH_2$), 129.9 ($CH_2C=CH_2$), 61.7 ($CH_2C=CH_2$), 56.3 (OCH_2CH_3), 38.3 (SCH_3), 14.3 (OCH_2CH_3). **ν_{max}** (neat)/ cm^{-1} 2983, 2933, 2914, 1709, 1626, 1185, 1125, 1046. **HRMS** (ESI) calculated for $C_7H_{12}SO_3Na$ ($M+Na^+$): 199.0399, found 199.0390.

(But-3-en-2-ylsulfinyl)benzene (SI-2o)

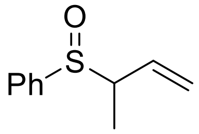

To a suspension of *N*-chlorosuccinimide (681 mg, 5.10 mmol) in DCM (2.5 mL) was added added a portion (*ca.* 1/5th) of thiophenol (500 µL, 5.0 mmol) and the reaction mixture was heated vigorously until deep orange colour appeared. The remaining thiophenol was added dropwise at a rate to maintain gentle reflux. The reaction mixture was then stirred at room temperature for 30 min and the resulting phenyl sulfenyl chloride was used immediately without isolation.

To a solution of crotyl alcohol (510 µL, 6.0 mmol) in THF (3 mL) at -78 °C was added *n*BuLi (1.6M in hexane, 3.75 mL, 6.00 mmol) dropwise, the reaction mixture was allowed to warm up to room temperature and stirred for 30 min. To the reaction mixture was then added phenyl sulfenyl chloride and the reaction mixture was stirred for 2 h. The solvent was removed *in vacuo*, to the residue was added $CHCl_3$ (5 mL), and the resulting mixture was stirred overnight. The precipitate was filtered, and the solvent was removed from the filtrate *in vacuo*. The residue was purified by column chromatography (40% EtOAc/hexane) to afford the title compound as a yellow oil (308 mg, 34%) and as a mixture of diastereoisomers (1:1).

^{1}H NMR (400 MHz, Chloroform-*d*) δ 7.60 – 7.53 (m, 4H, Ar-*H* x 4), 7.49 (ddd, *J* = 5.9, 2.9, 1.7 Hz, 6H, Ar-*H* x 6), 5.67 – 5.54 (m, 2H $CHCH=CH_2$ x 2), 5.32 – 5.24 (m, 2H, $CHCH=CH_2$), 5.13 (dt, *J* = 10.5, 1.2 Hz, 1H, $CHCH=CH_2$), 5.09 (dt, *J* = 10.6, 1.2 Hz, 1H, $CHCH=CH_2$), 3.50 – 3.42 (m, 1H, $CHCH=CH_2$), 3.42 – 3.34 (m, 1H, $CHCH=CH_2$), 1.33 (d, *J* = 1.4 Hz, 3H, $CHCH_3$), 1.31 (d, *J* = 1.4 Hz, 3H, $CHCH_3$). **^{13}C NMR** (101 MHz, $CDCl_3$) δ 141.5 (C_q), 141.0 (C_q), 132.6, 132.4, 131.8, 131.4, 131.2, 128.8 (*C*H x 2), 128.8 (*C*H x 2), 125.6 (*C*H x 2), 125.4 (*C*H x 2), 121.0, 63.9 ($CHCH=CH_2$), 62.7 ($CHCH=CH_2$), 12.4 ($CH(CH_3)$), 12.2 ($CH(CH_3)$). **ν_{max}** (neat)/ cm^{-1} 3465, 3059, 2971, 1733, 1443, 1146, 1045, 1022, 749. **HRMS** (ESI) calculated for $C_{10}H_{12}OSNa$ ($M+Na^+$): 203.0501, found 203.0498.

3-(Methylsulfinyl)prop-1-yne (2a')

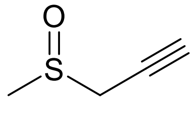

Following general procedure B using sodium methanethiolate (1.05 g, 15.0 mmol), freshly distilled propargyl bromide (1.89 g, 16.0 mmol), and *m*CPBA (3.36 g, 77% wt., 15.0 mmol) afforded the title compound as a yellow oil (513 mg, 33%).

^{1}H NMR (400 MHz, Chloroform-*d*) δ 3.57 (dd, *J* = 2.7, 1.6 Hz, 2H, CH_2), 2.73 (s, 3H, CH_3), 2.46 (t, *J* = 2.7 Hz, 1H, C*H*). **^{13}C NMR** (101 MHz, CDCl3) δ 76.6 (*C*H), 72.5 (C_q), 44.0 (CH_2), 38.0 (CH_3). **ν_{max}** (neat)/ cm^{-1} 3221, 2914, 2360, 2341, 1652, 1419, 1034, 975, 946. **HRMS** (APCI) calculated for C_4H_7SO ($M+H^+$): 103.0212, found 103.0217.

(Prop-2-yn-1-ylsulfinyl)benzene (2b')

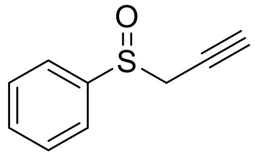

To a solution of phenyl propargyl sulfide (687 µL, 5.00 mmol) in MeOH (25 mL) at 0 °C was added aqueous solution (50 mL) of sodium periodate (1.12 g, 5.25 mmol) in portions and stirred at 0 °C for 2 h. The reaction mixture was concentrated *in vacuo* and the aqueous solution was extracted with $CHCl_3$/*iso*-propanol (3:1) (40 mL x 2). The combined organic extracts were dried over Na_2SO_4, filtered, and the solvent was removed *in vacuo*. Purification by column chromatography (CH_2Cl_2 -> 10% MeOH/CH_2Cl_2) afforded the title compound as a brown oil (596 mg, 73%).

^{1}**H NMR** (400 MHz, Chloroform-*d*) δ 7.75 – 7.68 (m, 2H, Ar-*H* x 2), 7.58 – 7.52 (m, 3H, Ar-*H* x 3), 3.67 (dd, *J* = 15.7, 2.7 Hz, 1H, CH_2CCH), 3.61 (dd, *J* = 15.7, 2.7 Hz, 2H, CH_2CCH), 2.34 (t, *J* = 2.7 Hz, 1H, CH_2CC*H*). ^{13}**C NMR** (101 MHz, $CDCl_3$) δ 143.0 (C_q), 131.9 (*C*H), 129.3 (*C*H x 2), 124.6 (*C*H x 2), 82.5 (CH_2*C*CH), 76.6 (CH_2C*C*H), 48.0 (*C*H_2CCH). **ν_{max}** (neat)/ cm^{-1} 3291, 3057, 2951, 2905, 2360, 2341, 1443, 1395, 1086, 1047. **HRMS** (APCI) calculated for C_9H_9SO ($M+H^+$): 165.0369, found 165.0373.

General procedure C: Dual vicinal functionalisation of indoles using a range of allylic sulfoxides:

A stirred mixture of sulfoxide **2** (0.250 mmol), 1-methyl-indole (32.8 mg, 31.0 µL, 0.250 mmol), and sodium bicarbonate (46.2 mg, 0.550 mmol, 2.20 eq.) in CH_2Cl_2 (2.5 mL) under N_2 was cooled to -78 °C and TFAA (38 µL, 0.275 mmol, 1.1 eq.) was added dropwise. The reaction mixture was stirred at -78 °C for 15 min, then allowed to warm up to room temperature and stirred for 1 h. The crude reaction mixture was filtered through a plug of silica, eluted with CH_2Cl_2, and concentrated *in vacuo* to give the desired products.

2-Allyl-1-methyl-3-(methylthio)-1*H*-indole (3b)

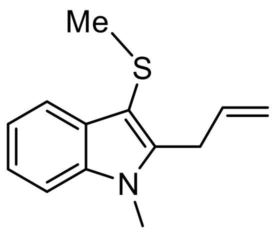

Following general procedure C using methyl allyl sulfoxide **2a** (26.0 mg, 0.250 mmol) afforded the title compound as a pale yellow oil (52.3 mg, 96%).

^{1}H NMR (400 MHz, Chloroform-*d*) δ 7.81 (ddd, *J* = 7.7, 1.5, 0.8 Hz, 1H, Ar-*H*), 7.37 – 7.34 (m, 1H, Ar-*H*), 7.29 (ddd, *J* = 7.7, 7.0, 1.4 Hz, 1H, Ar-*H*), 7.24 (ddd, *J* = 8.2, 7.0, 1.5 Hz, 1H, Ar-*H*), 6.00 (ddt, *J* = 17.1, 10.1, 5.6 Hz, 1H, $CH_2CH{=}CH_2$), 5.16 (dq, *J* = 10.1, 1.8 Hz, 1H, $CH_2CH{=}CH_2$), 5.01 (dq, *J* = 17.1, 1.8 Hz, 1H, $CH_2CH{=}CH_2$), 3.84 (dt, *J* = 5.6, 1.8 Hz, 2H, $CH_2CH{=}CH_2$), 3.73 (s, 3H, NCH_3), 2.31 (s, 3H, SCH_3). **^{13}C NMR** (101 MHz, Chloroform-d) δ 142.0 (C_q), 137.2 (C_q), 135.0 ($CH_2CH{=}CH_2$), 129.5 (C_q), 121.9 (*C*H), 120.2 (*C*H), 119.1 (*C*H), 116.5 ($CH_2CH{=}CH_2$), 109.3 (*C*H), 104.3 (*C*SCH_3), 30.4 (NCH_3), 29.4 ($CH_2CH{=}CH_2$), 20.7 (SCH_3). **ν_{max}** (neat)/ cm^{-1} 2916, 1636, 1522, 1465, 1422, 1340, 1242, 1012, 993, 963, 733. **HRMS** (APCI) calculated for $C_{13}H_{16}NS$ $(M+H^+)$: 218.0998, found 218.0997.

2-allyl-3-(ethylthio)-1-methyl-1*H*-indole (3u)

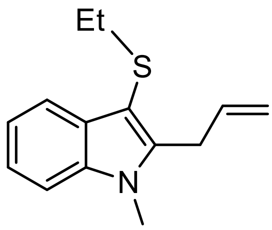

Following general procedure C using ethyl allyl sulfoxide (29.5 mg, 0.250 mmol) afforded the title compound as a pale yellow oil (49.5 mg, 86%).

^{1}H NMR (400 MHz, Chloroform-*d*) δ 7.75 (ddd, *J* = 8.0, 1.5, 1.4 Hz, 1H, Ar-*H*), 7.32-7.29 (m, 1H, Ar-*H*), 7.23 (ddd, *J* = 8.0, 7.0, 1.5 Hz, 1H, Ar-*H*), 7.17 (ddd, *J* = 8.0, 7.0, 1.4 Hz, 1H, Ar-*H*), 5.94 (ddt, *J* = 17.1, 10.2, 5.7 Hz, 1H, $CH_2CH{=}CH_2$), 5.11 (dq, *J* = 10.2, 1.8 Hz, 1H, $CH_2CH{=}CH_2$), 4.97 (dq, *J* = 17.1, 1.8 Hz, 1H, $CH_2CH{=}CH_2$), 3.80 (dt, *J* = 5.7, 1.8 Hz, 2H, $CH_2CH{=}CH_2$), 3.70 (s, 3H, NCH_3), 2.65 (q, *J* = 7.3 Hz, 2H, SCH_2CH_3), 1.16 (t, *J* = 7.3 Hz, 3H, SCH_2CH_3). **^{13}C NMR** (101 MHz, Chloroform-d) δ 142.7 (C_q), 137.2 (C_q), 135.1 ($CH_2CH{=}CH_2$), 130.3 (C_q), 121.8 (*C*H), 120.2 (*C*H), 119.3 (*C*H), 116.6 ($CH_2CH{=}CH_2$), 109.2 (*C*H), 102.3 (*C*SCH_2CH_3), 30.9 (SCH_2CH_3), 30.5 (NCH_3), 29.4 ($CH_2CH{=}CH_2$), 15.5 (SCH_2CH_3). **ν_{max}** (neat)/ cm^{-1} 3054, 2922, 1637, 1523, 1466, 1392, 1241, 1168, 915, 745, 735. **HRMS** (APCI) calculated for $C_{14}H_{18}NS$ $(M+H^+)$: 232.1154, found 232.1155.

2-Allyl-3-(isopropylthio)-1-methyl-1H-indole (3v)

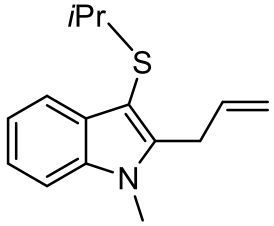

Following general procedure C using isopropyl allyl sulfoxide (33.0 mg, 0.250 mmol) afforded the title compound as a pale yellow oil (54.3 mg, 89%).

^{1}H NMR (400 MHz, Chloroform-*d*) δ 7.82 – 7.77 (m, 1H, Ar-*H*), 7.36 – 7.31 (m, 1H, Ar-*H*), 7.26 (ddd, J = 7.9, 6.9, 1.4 Hz, 1H, Ar-*H*), 7.21 (ddd, J = 8.1, 6.9, 1.3 Hz, 1H, Ar-*H*), 5.97 (ddt, J = 17.2, 10.2, 5.7 Hz, 1H, $CH_2CH{=}CH_2$), 5.15 (dq, J = 10.2, 1.8 Hz, 1H, $CH_2CH{=}CH_2$), 5.01 (dq, J = 17.2, 1.8 Hz, 1H, $CH_2CH{=}CH_2$), 3.84 (dt, J = 5.7, 1.8 Hz, 2H, $CH_2CH{=}CH_2$), 3.72 (s, 3H, NCH_3), 3.12 (hept, J = 6.7 Hz, 1H, $SCH(CH_3)_2$), 1.26 (d, J = 6.7 Hz, 6H, $SCH(CH_3)_2$). **^{13}C NMR** (101 MHz, Chloroform-d) δ 143.0 (C_q), 137.1 (C_q), 134.9 ($CH_2CH{=}CH_2$), 130.6 (C_q), 121.7 (*C*H), 120.0 (*C*H), 119.5 (*C*H), 116.5 ($CH_2CH{=}CH_2$), 109.0 (*C*H), 102.0 ($CSCH(CH_3)_2$), 39.7 ($SCH(CH_3)_2$), 30.5 (NCH_3), 29.3 ($CH_2CH{=}CH_2$), 23.5 ($SCH(CH_3)_2$). **ν_{max}** (neat)/ cm^{-1} 2957, 2923, 1672, 1526, 1465, 1392, 1012, 915, 877, 735. **HRMS** (APCI) calculated for $C_{15}H_{20}NS$ ($M+H^+$): 246.1311, found 246.1316.

2-Allyl-3-(allylthio)-1-methyl-1H-indole (3w)

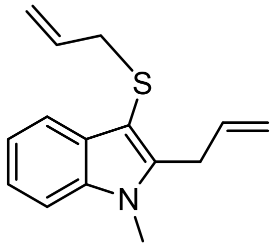

Following general procedure C using diallyl sulfoxide (32.5 mg, 0.250 mmol) afforded the title compound as a pale yellow oil (49.9 mg, 82%).

To a solution of diallyl sulfoxide (900 mg, 6.9 mmol) and 1-methyl-indole (865 µL, 10.0 mmol) in CH_2Cl_2 (100 mL) containing potassium phosphate (3.22 g, 15.2 mmol) under N_2 was cooled to -78 °C and TFAA (1.00 mL, 7.17 mmol) was added dropwise. The reaction mixture was stirred at -78 °C for 15 min, then allowed to warm up to room temperature, and stirred for 1 h. The crude reaction mixture was filtered through a plug of silica, and eluted with CH_2Cl_2. Solvent was removed *in vacuo* to give title compound as an orange oil (1.66 g, >99%).

^{1}H NMR (400 MHz, Chloroform-*d*) δ 7.77 – 7.72 (m, 1H, Ar-*H*), 7.32 – 7.28 (m, 1H, Ar-*H*), 7.23 (ddd, J = 8.1, 7.0, 1.4 Hz, 1H, Ar-*H*), 7.18 (ddd, J = 8.1, 7.0, 1.3 Hz, 1H, Ar-*H*), 5.92 (ddt, J = 17.3, 10.0, 5.8 Hz, 1H, $CH_2CH{=}CH_2$), 5.84 (ddt, J = 17.0, 10.0, 7.4 Hz, 1H, $SCH_2CH{=}CH_2$), 5.11 (dq, J = 10.0, 1.6 Hz, 1H, $CH_2CH{=}CH_2$), 4.97 (dq, J = 17.3, 1.8 Hz, 1H, $CH_2CH{=}CH_2$), 4.90 – 4.86 (m, 1H, $SCH_2CH{=}CH_2$), 4.83 (dq, J = 17.0, 1.2 Hz, 1H, $SCH_2CH{=}CH_2$), 3.77 (dt, J = 5.8, 1.8 Hz, 2H, $CH_2CH{=}CH_2$), 3.69 (s, 3H, NCH_3), 3.26 (dt, J = 7.4, 1.2 Hz, 2H, $SCH_2CH{=}CH_2$). **^{13}C NMR** (101 MHz, Chloroform-*d*) δ 143.2 (C_q), 137.2 (C_q), 135.2 ($CH_2CH{=}CH_2$), 135.1 ($CH_2CH{=}CH_2$), 130.1 (C_q), 121.8 (*C*H), 120.2 (*C*H), 119.3 (*C*H), 116.7 ($CH_2CH{=}CH_2$), 116.6 ($SCH_2CH{=}CH_2$), 109.2 (*C*H), 101.8 ($CSCH_2CH{=}CH_2$), 40.1 ($CH_2CH{=}CH_2$), 30.6 (NCH_3), 29.5 ($SCH_2CH{=}CH_2$). **ν_{max}** (neat)/ cm^{-1} 3078, 2912, 1635, 1466, 1392, 1339, 1167, 1012, 989, 912, 734. **HRMS** (APCI) calculated for $C_{15}H_{18}NS$ ($M+H^+$): 244.1154, found 244.1145.

2-Allyl-1-methyl-3-(phenylthio)-1*H*-indole (3x)

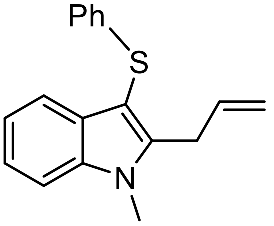

Following general procedure C using phenyl allyl sulfoxide (41.5 mg, 0.250 mmol) afforded the title compound as a yellow solid (61.9 mg, 89%).

Following general procedure C using phenyl allyl sulfoxide (830 mg, 5.00 mmol), 1-methyl-1*H*-indole (625 µL, 5.00 mmol), trifluoroacetic anhydride (768 µL, 5.50 mmol), and potassium phosphate (2.33 g, 11.0 mmol) in CH_2Cl_2 (50 mL) afforded the title compound as an orange solid (1.31 g, 94%)

M.p.: 63-65 °C. **^{1}H NMR** (400 MHz, Chloroform-*d*) δ 7.64 – 7.58 (m, 1H, Ar-*H*), 7.37 (dt, *J* = 8.2, 0.9 Hz, 1H, Ar-*H*), 7.27 (ddd, *J* = 8.2, 7.0, 1.2 Hz, 1H, Ar-*H*), 7.19 – 7.11 (m, 3H, Ar-*H* x 3), 7.09 – 7.01 (m, 3H, Ar-*H* x 3), 5.86 (ddt, *J* = 17.0, 10.0, 5.8 Hz, 1H, $CH_2CH{=}CH_2$), 5.09 (dt, *J* = 10.2, 1.6 Hz, 1H, $CH_2CH{=}CH_2$), 4.99 (dq, *J* = 17.2, 1.7 Hz, 1H, $CH_2CH{=}CH_2$), 3.85 – 3.61 (m, 5H, $CH_2CH{=}CH_2$ and NCH_3). **^{13}C NMR** (101 MHz, Chloroform-d) δ 143.8 (C_q), 139.9 (C_q), 137.5 (C_q), 134.4 ($CH_2CH{=}CH_2$), 129.8 (C_q), 128.8 (*C*H), 125.7 (*C*H), 124.6 (*C*H), 122.2 (*C*H), 120.7 (*C*H), 119.4 (*C*H), 116.9 ($CH_2CH{=}CH_2$), 109.4 (*C*H), 99.0 (*C*SPh), 30.7 ($CH_2CH{=}CH_2$), 29.5 (NCH_3). **ν_{max}** (neat)/ cm^{-1} 3055, 2928, 1637, 1581, 1476, 1467, 1393, 917, 736, 690. **HRMS** (APCI) calculated for $C_{18}H_{18}NS$ ($M+H^+$): 280.1154, found 280.1144.

3-(methylthio)-2-(1-phenylallyl)-1*H*-indole (3y) and **2-(methylthio)-3-(1-phenylallyl)-1*H*-indole (4y)**

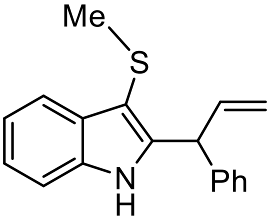

3y

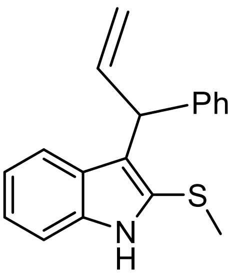

4y

Following general procedure C using (*E*)-(3-(methylsulfinyl)prop-1-en-1-yl)benzene (44.0 mg, 0.250 mmol) and 1-*H*-indole (29.3 mg, 0.250 mmol) afforded the title compounds as a pale yellow oil (90%) and as a mixture of regioisomers (**3y** : **4y** = 4.8 : 1). The yield and the ratio of regioisomers were determined by ^{1}H NMR analysis of the crude product with $MeNO_2$ as internal standard.

3y: **^{1}H NMR** (400 MHz, Chloroform-*d*) δ 7.93 (br s, 1H, N*H*), 7.82 – 7.73 (m, 1H, Ar-*H*), 7.35 (ddd, *J* = 7.4, 6.3, 2.0 Hz, 2H, Ar-*H* x 2), 7.32 – 7.25 (m, 4H, Ar-*H* x 4), 7.23 – 7.17 (m, 2H, Ar-*H* x 2), 6.34 (ddd, *J* = 17.0, 10.2, 6.0 Hz, 1H, $CHCH{=}CH_2$), 5.58 (dd, *J* = 5.8, 1.8 Hz, 1H, $CHCH{=}CH_2$), 5.36 (dd, *J* = 10.2, 1.4 Hz, 1H, $CHCH{=}CH_2$), 5.07 (dt, *J* = 17.2, 1.5 Hz, 1H, $CHCH{=}CH_2$), 2.22 (s, 3H, SCH_3). **^{13}C NMR** (101 MHz, Chloroform-*d*) δ 142.8 (C_q), 141.1 (C_q), 138.2 ($CHCH{=}CH_2$), 135.6 (C_q), 130.1 (C_q), 129.0 (*C*H), 128.4 (*C*H), 127.2 (*C*H), 122.4 (*C*H), 120.6 (*C*H), 119.3 (*C*H), 118.1 ($CHCH{=}CH_2$), 111.2 (*C*H), 105.3 ($CSCH_3$), 46.1 ($CHCH{=}CH_2$), 19.9 (SCH_3).

4y: **^{1}H NMR** (400 MHz, Chloroform-*d*) δ 8.16 (br s, 1H, N*H*), 7.42 – 7.37 (m, 1H, Ar-*H*), 7.36 – 7.30 (m, 3H, Ar-*H* x 3), 7.30 – 7.24 (m, 2H, Ar-*H* x 2), 7.22 – 7.14 (m, 2H, Ar-*H* x 2), 6.99 (ddd, *J* = 8.0, 7.0, 1.0 Hz, 1H, Ar-*H*), 6.55 (ddd, *J* = 17.2, 10.2, 7.2 Hz, 1H, $CHCH{=}CH_2$), 5.39 – 5.33 (m, 1H, $CHCH{=}CH_2$), 5.24 (dt, *J* = 10.1, 1.5 Hz, 1H, $CHCH{=}CH_2$), 5.14 (dt, *J* = 17.1, 1.6 Hz, 1H, $CHCH{=}CH_2$), 2.32 (s, 3H, SCH_3). **^{13}C NMR** (101 MHz, Chloroform-*d*) δ 143.2 (C_q), 139.9 ($CHCH{=}CH_2$), 136.8 (C_q), 128.3 (*C*H), 128.3 (*C*H), 127.4 ($CSCH_3$), 127.0 (C_q), 126.2 (*C*H), 123.0 (*C*H), 122.3 (C_q), 120.9 (*C*H), 119.8 (*C*H), 116.2 ($CHCH{=}CH_2$), 110.9 (*C*H), 46.7 ($CHCH{=}CH_2$), 20.4 (SCH_3).

3y and **4y: ν_{max}** (neat)/ cm^{-1} 3403, 3057, 2917, 1491, 1452, 1415, 1291, 968, 923, 743, 700. **HRMS** (APCI) calculated for $C_{18}H_{18}NS$ ($M+H^+$): 280.1154, found 280.1156.

Ethyl 2-(3-(methylthio)-1*H*-indol-2-yl)but-3-enoate (3z)

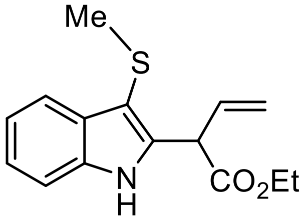

Following general procedure C using ethyl (*E*)-4-(methylsulfinyl)but-2-enoate (44.0 mg, 0.250 mmol) and 1-*H*-indole (29.3 mg, 0.250 mmol) afforded the title compound as a yellow oil (47.0 mg, 65%).

^{1}H NMR (400 MHz, Chloroform-*d*) δ 9.02 (br s, 1H, N*H*), 7.86 – 7.72 (m, 1H, Ar-*H*), 7.45 – 7.35 (m, 1H, Ar-*H*), 7.28 – 7.16 (m, 2H, Ar-*H* x 2), 6.14 (ddd, *J* = 16.9, 10.1, 6.9 Hz, 1H, CHC*H*=CH_2), 5.28 – 5.17 (m, 3H, CHCH=CH_2 and C*H*CH=CH_2), 4.30 – 4.19 (m, 2H, OC$H_2$$CH_3$), 2.28 (s, 3H, SC$H_3$), 1.31 (t, *J* = 7.1 Hz, 3H, OCH_2CH_3). **^{13}C NMR** (101 MHz, Chloroform-*d*) δ 172.1 (*C*OO$CH_2$$CH_3$), 137.4 ($C_q$), 135.8 ($C_q$), 134.3 (CH*C*H=$CH_2$), 129.6 ($C_q$), 122.8 (*C*H), 120.6 (*C*H), 119.3 (*C*H), 118.4 (CHCH=*C*H_2), 111.6 (*C*H), 105.9 (*C*SCH_3), 61.9 (O*C*$H_2$$CH_3$), 46.9 (*C*HCH=$CH_2$), 20.1 (S*C*$H_3$), 14.3 (O$CH_2$*C*$H_3$). **$\nu_{max}$** (neat)/ cm^{-1} 3380, 2981, 2919, 1715, 1635, 1453, 1317, 1295, 1177, 1025, 927, 743. **HRMS** (APCI) calculated for $C_{15}H_{18}NO_2S$ ($M+H^+$): 276.1053, found 276.1041.

Ethyl 2-(1-methyl-3-(methylthio)-1*H*-indol-2-yl)but-3-enoate (3aa) and **ethyl 2-(1-methyl-2-(methylthio)-1*H*-indol-3-yl)but-3-enoate (4aa)**

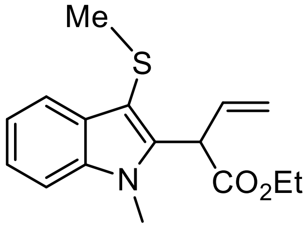

3aa

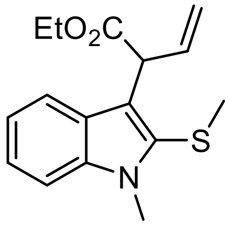

4aa

Following general procedure C using ethyl (*E*)-4-(methylsulfinyl)but-2-enoate (44.0 mg, 0.250 mmol). Reaction was stirred for 1 h at room temperature, followed by 30 min at 60 °C to afford the title compounds as a yellow oil (83%) and as a mixture of regioisomers (**3aa** : **4aa** = 3 : 1). The yield and the ratio of regioisomers were determined by ^{1}H NMR analysis of the crude product with $MeNO_2$ as internal standard.

3aa: **^{1}H NMR** (400 MHz, Chloroform-*d*) δ 7.74 – 7.67 (m, 1H, Ar-*H*), 7.26 – 7.10 (m, 3H, Ar-*H* x 3), 6.46 – 6.33 (m, 1H, CHC*H*=CH_2), 5.38 (dt, *J* = 4.6, 2.2 Hz, 1H, C*H*CH=CH_2), 5.16 (ddd, *J* = 10.5, 2.2, 1.0 Hz, 1H, CHCH=CH_2), 4.76 (ddd, *J* = 17.4, 2.2, 1.1 Hz, 1H, CHCH=CH_2), 4.17 – 3.95 (m, 2H, OC$H_2$$CH_3$), 3.54 (s, 3H, NC$H_3$), 2.17 (s, 3H, SC$H_3$), 1.13 (t, *J* = 7.1 Hz, 3H, OCH_2CH_3). **^{13}C NMR** (101 MHz, Chloroform-*d*) δ 171.2 (*C*$O_2$$CH_2$$CH_3$), 139.7 ($C_q$), 137.6 ($C_q$), 133.4 (CH*C*H=$CH_2$), 129.2 ($C_q$), 122.5 (*C*H), 120.4 (*C*H), 119.5 (*C*H), 118.0 (CHCH=*C*H_2), 109.6 (*C*H), 106.8 (*C*SCH_3), 61.7 (O*C*$H_2$$CH_3$), 45.7 (*C*HCH=$CH_2$), 31.5 (N*C*$H_3$), 20.7 (S*C*$H_3$), 14.3 (O$CH_2$*C*$H_3$).

4aa: **^{1}H NMR** (400 MHz, Chloroform-*d*) δ 7.59 (dt, *J* = 8.1, 1.0 Hz, 1H, Ar-*H*), 7.26 – 7.10 (m, 2H, Ar-*H* x 2), 7.00 (ddd, *J* = 8.1, 6.9, 1.2 Hz, 1H, Ar-*H*), 6.46 – 6.33 (m, 1H, CHC*H*=CH_2), 5.06 (dt, *J* = 10.0, 1.3 Hz, 1H, CHCH=CH_2), 5.02 – 4.93 (m, 2H, CHCH=CH_2 and C*H*CH=CH_2), 4.17 – 3.95 (m, 2H, OC$H_2$$CH_3$), 3.78 (s, 3H, NC$H_3$), 2.19 (s, 3H, SC$H_3$), 1.10 (t, *J* = 7.1 Hz, 3H, OCH_2CH_3). **^{13}C NMR** (101 MHz, Chloroform-*d*) δ 172.9 (*C*$O_2$$CH_2$$CH_3$), 138.0 ($C_q$), 135.4 (CH*C*H=$CH_2$), 130.9 (*C*S$CH_3$), 125.5 ($C_q$), 123.0 (*C*H), 120.7 (*C*H), 119.8 (*C*H), 117.7 (C_q), 116.8 (CHCH=*C*H_2), 109.8 (*C*H), 61.1 (O*C*$H_2$$CH_3$), 48.1 (*C*HCH=$CH_2$), 30.3 (N*C*$H_3$), 20.4 (S*C*$H_3$), 14.3 (O$CH_2$*C*$H_3$).

3aa and **4aa**: **ν_{max}** (neat)/ cm^{-1} 2980, 2919, 1730, 1465, 1392, 1180, 1030, 924, 740. **HRMS** (ESI) calculated for $C_{16}H_{20}NO_2S$ ($M+H^+$): 290.1209, found 290.1208.

2-(2-methylbut-3-en-2-yl)-3-(methylthio)-1*H*-indole (3ab)

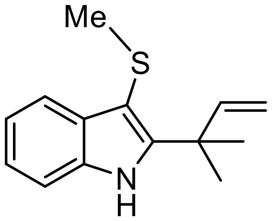

Following general procedure C using 3-methyl-1-(methylsulfinyl)but-2-ene (33.0 mg, 0.250 mmol) and 1-*H*-indole (29.3 mg, 0.250 mmol). Purification by column chromatography (10% EtOAc/hexane) afforded the title compound as a yellow oil (32.5 mg, 56%)

^{1}H NMR (400 MHz, Chloroform-*d*) δ 8.16 (br s, 1H, N*H*), 7.81 – 7.74 (m, 1H, Ar-*H*), 7.37 – 7.33 (m, 1H, Ar-*H*), 7.23 – 7.20 (m, 2H, Ar-*H* x 2), 6.31 (dd, *J* = 17.4, 10.6 Hz, 1H, CC*H*=CH$_2$), 5.26 (dd, *J* = 10.6, 1.0 Hz, 1H, CCH=C*H$_2$*), 5.25 (dd, *J* = 17.4, 1.0 Hz, 1H, CCH=C*H$_2$*), 2.33 (s, 3H, SC*H$_3$*), 1.73 (s, 6H, C(CH$_3$)$_2$). **^{13}C NMR** (101 MHz, Chloroform-*d*) δ 147.1 (*C*$_q$), 145.5 (*C*CH=CH$_2$), 134.0 (*C*$_q$), 131.6 (*C*$_q$), 122.1 (*C*H), 120.4 (*C*H), 118.7 (*C*H), 113.3 (CCH=*C*H$_2$), 111.0 (*C*H), 102.7 (*C*SCH$_3$), 39.6 (*C*(CH$_3$)$_2$), 27.4 (C*C*H$_3$ x 2), 20.2 (S*C*H$_3$). **ν_{max}** (neat)/ cm^{-1} 3431, 2966, 2917, 1453, 1403, 1289, 1217, 1008, 967, 924, 744. **HRMS** (ESI) calculated for $C_{14}H_{18}NS$ (M+H$^+$): 232.1154, found 232.1147.

1-methyl-2-(2-methylbut-3-en-2-yl)-3-(methylthio)-1*H*-indole (3ac) and **1-methyl-3-(2-methylbut-3-en-2-yl)-2-(methylthio)-1*H*-indole (4ac)**

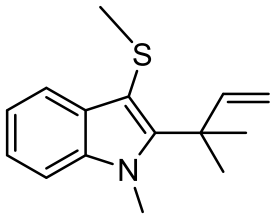

3ac

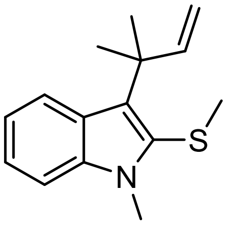

4ac

Following general procedure C using 3-methyl-1-(methylsulfinyl)but-2-ene (33.0 mg, 0.250 mmol). Purification by column chromatography (10% EtOAc/hexane) afforded the title compounds as a yellow oil (28.7 mg, 47%) and as a mixture of regioisomers (**3ac** : **4ac** = 4.5:1)

3ac: **^{1}H NMR** (400 MHz, Chloroform-*d*) δ 7.90 – 7.84 (m, 1H, Ar-*H*), 7.32 – 7.27 (m, 2H, Ar-*H* x 2), 7.27 – 7.19 (m, 1H, Ar-*H*), 6.24 (dd, *J* = 17.5, 10.6 Hz, 1H, CC*H*=CH$_2$), 5.13 (dd, *J* = 10.6, 1.0 Hz, 1H, CCH=C*H$_2$*), 5.00 (dd, *J* = 17.5, 1.0 Hz, 1H, CCH=C*H$_2$*), 3.78 (s, 3H, NC*H$_3$*), 2.32 (s, 3H, SC*H$_3$*), 1.83 (s, 6H, C(C*H$_3$*)$_2$). **^{13}C NMR** (101 MHz, Chloroform-*d*) δ 147.7 (*C*CH=CH$_2$), 146.7 (*C*$_q$), 137.9 (*C*$_q$), 130.6 (*C*$_q$), 122.4 (*C*H), 120.2 (*C*H), 119.4 (*C*H), 111.9 (CCH=*C*H$_2$), 109.1 (*C*H), 104.5 (*C*SCH$_3$), 41.5 (*C*(CH$_3$)$_2$), 33.7 (N*C*H$_3$), 30.0 (C(*C*H$_3$) x 2), 21.8 (S*C*H$_3$).

4ac: **^{1}H NMR** (400 MHz, Chloroform-*d*) δ 7.95 (dt, *J* = 8.3, 1.0 Hz, 1H, Ar-*H*), 7.33 – 7.20 (m, 2H, Ar-*H* x 2), 7.07 (ddd, *J* = 8.3, 6.8, 1.0 Hz, 1H, Ar-H), 6.44 (dd, *J* = 17.5, 10.5 Hz, 1H, CC*H*=CH$_2$), 5.12 (dd, *J* = 17.5, 1.2 Hz, 1H, CCH=C*H$_2$*), 5.07 (dd, *J* = 10.5, 1.2 Hz, 1H, CCH=C*H$_2$*), 3.88 (s, 3H, NC*H$_3$*), 2.27 (s, 3H, SC*H$_3$*), 1.77 (s, 6H, C(C*H$_3$*)$_2$). **^{13}C NMR** (101 MHz, Chloroform-*d*) δ 149.7 (*C*CH=CH$_2$), 138.0 (*C*$_q$), 128.7 (*C*SCH$_3$), 126.2 (*C*$_q$), 122.7 (*C*H), 118.8 (*C*H), 109.8 (*C*H), 109.6 (CCH=*C*H$_2$), 40.2 (*C*(CH$_3$)$_2$), 29.8 (C(*C*H$_3$) x 2), 29.6 (N*C*H$_3$), 21.1 (S*C*H$_3$). *C*H x 1 and *C*$_q$ x 1 unassignable.

3ac and **4ac**: **ν_{max}** (neat)/ cm^{-1} 2967, 2918, 1467, 1331, 1225, 912, 741. **HRMS** (APCI) calculated for $C_{15}H_{20}NS$ (M+H$^+$): 246.1311, found 246.1303.

2-(but-2-en-1-yl)-1-methyl-3-(phenylthio)-1H-indole (3ad)

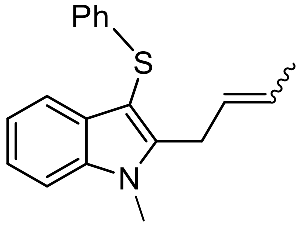

Following general procedure C using (but-3-en-2-ylsulfinyl)benzene (45.0 mg, 0.250 mmol). Purification by column chromatography (10% EtOAc/hexane) afforded the title compound as a pale yellow oil (46.2 mg, 63%) as a mixture of isomers (E:Z = 1.7 : 1.0).

^{1}H NMR (400 MHz, Chloroform-*d*) δ 7.62 (dq, *J* = 7.8, 1.0 Hz, 1.6H, Ar-*H* (*E* and *Z*)), 7.37 (dt, *J* = 8.2, 1.0 Hz, 1.6H, Ar-H (*E* and *Z*)), 7.30 – 7.24 (m, 1.6H, Ar-*H* (*E* and *Z*)), 7.19 – 7.13 (m, 4.8H, Ar-*H* x 3 (*E* and *Z*)), 7.10 – 7.03 (m, 4.8H, Ar-*H* x 3 (*E* and *Z*)), 5.62 – 5.51 (m, 0.6H, CH=C*H* (*Z*)), 5.51 – 5.29, (m, 2.6H, C*H*=C*H* (*E*) and CH=C*H* (*Z*)), 3.80 – 3.75 (m, 5.8H, NC*H*$_3$ (*E* and *Z*) and C*H*$_2$CH=CH (*Z*)), 3.72 – 3.67 (m, 2H, C*H*$_2$CH=CH$_2$ (*E*)), 1.76 (ddt, *J* = 6.9, 2.0, 1.1 Hz, 1.6H, CH=CHC*H*$_3$ (*Z*)), 1.62 (dq, *J* = 4.6, 1.4 Hz, 3H, CH=CHC*H*$_3$ (*E*)). **^{13}C NMR** (101 MHz, CDCl$_3$) δ 145.5 (C_q (*Z*)), 144.9 (C_q (*E*)), 140.0 (C_q (*Z*)), 140.0 (C_q (*E*)), 137.5 (C_q (*Z*)), 137.5 (C_q (*E*)), 129.9 (C_q (*Z*)), 129.8 (C_q (*E*)), 128.7 (*C*H (*Z*)), 128.7 (*C*H (*E*)), 127.6 (*C*H=CH (*E*)), 126.8 (*C*H=CH (*E*)), 126.1 (*C*H=CH (*Z*)), 125.9 (*C*H=CH (*Z*)), 125.7 (*C*H (*E*)), 125.6 (*C*H (*Z*)), 124.6 (*C*H (*E* and *Z*)), 122.1 (*C*H (*Z*)), 122.1 (*C*H (*E*)), 120.7 (*C*H (*Z*)), 120.6 (*C*H (*E*)), 119.4 ((*C*H (*E* and *Z*)), 109.3 (*C*H (*E*)), 109.3 (*C*H (*Z*)), 98.5 (*C*SPh (*E*)), 98.3 (*C*SPh (*Z*)), 30.6 (N*C*H$_3$ x 2), 28.4 (C*H*$_2$CH=CH (*E*)), 23.7 (*C*H$_2$CH=CH (*Z*)), 18.0 (CH=CH*C*H$_3$ (*E*)), 13.2 (CH=CH*C*H$_3$ (*Z*)). **HRMS** (APCI) calculated for $C_{19}H_{20}NS$ (M+H$^+$): 294.1311, found 294.1306.

2-(Cyclohex-2-en-1-yl)-1-methyl-3-(methylthio)-1*H*-indole (3ae)

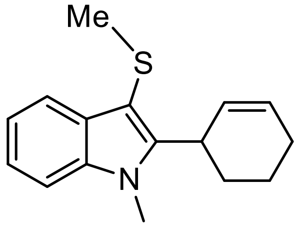

Following general procedure C using 3-(methylsulfinyl)cyclohex-1-ene (36.0mg, 0.250 mmol) afforded the title compound as a pale yellow oil (52.8 mg, 82%).

^{1}H NMR (400 MHz, Chloroform-*d*) δ 7.78 (ddd, *J* = 7.5, 1.6, 0.9 Hz, 1H, Ar-*H*), 7.34 – 7.31 (m, 1H, Ar-*H*), 7.26 (ddd, *J* = 8.1, 6.8, 1.6 Hz, 1H, Ar-*H*), 7.24 – 7.19 (m, 1H, Ar-*H*), 5.96 (ddd, *J* = 10.7, 4.2, 3.3 Hz, 1H, CHCH=C*H*), 5.81 – 5.70 (m, 1H, CHC*H*=CH), 4.59 – 4.44 (m, 1H, C*H*CH=CH), 3.80 (s, 3H, NC*H*$_3$), 2.29 (s, 3H, SC*H*$_3$), 2.26 – 2.18 (m, 2H, CH=CHC*H*$_2$), 2.06 – 1.96 (m, 2H, 1H of CHC*H*$_2$CH$_2$ and 1H of CHCH$_2$C*H*$_2$), 1.91 – 1.81 (m, 2H, 1H of CHC*H*$_2$CH$_2$ and 1H of CHCH$_2$C*H*$_2$) **^{13}C NMR** (126 MHz, Chloroform-*d*) δ 146.9 (C_q), 137.6 (C_q), 129.4 (C_q), 129.1 (CH*C*H=CH), 128.5 (CHCH=*C*H), 121.9 (*C*H), 120.2 (*C*H), 119.1 (*C*H), 109.1 (*C*H), 104.0 (*C*SCH$_3$), 34.5 (*C*HCH=CH), 31.2 (N*C*H$_3$), 29.6 (*C*H$_2$), 25.0 (CH=CH*C*H$_2$), 23.0 (*C*H$_2$), 21.1 (S*C*H$_3$). **ν_{max}** (neat)/ cm^{-1} 2917, 1672, 1528, 1514, 1466, 1386, 1182, 1128, 888, 878, 740. **HRMS** (APCI) calculated for $C_{16}H_{20}NS$ (M+H$^+$): 258.1311, found 258.1301.

1-methyl-2-(2-methylallyl)-3-(methylthio)-1*H*-indole (3af)

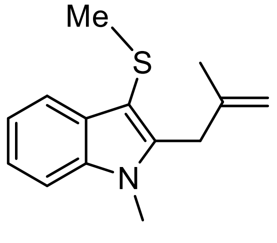

Following general procedure C using 2-methyl-3-(methylsulfinyl)prop-1-ene (29.5 mg, 0.250 mmol) afforded the title compound as a yellow oil (51.7 mg, 90%).

^{1}H NMR (400 MHz, Chloroform-*d*) δ 7.83 – 7.77 (m, 1H, Ar-*H*), 7.36 – 7.32 (m, 1H, Ar-*H*), 7.27 (ddd, *J* = 8.1, 7.0, 1.5 Hz, 1H, Ar-*H*), 7.22 (ddd, *J* = 7.0, 1.4 Hz, 1H, Ar-*H*), 4.88 (tq, *J* = 1.6, 1.0 Hz, 1H, CH$_2$C=C*H*$_2$), 4.55 (tq, *J* = 1.6, 1.0 Hz, 1H, CH$_2$C=C*H*$_2$), 3.80 – 3.71 (m, 2H, C*H*$_2$C=CH$_2$), 3.68 (s, 3H, NC*H*$_3$), 2.29 (s, 3H, SC*H*$_3$), 1.83 (dd, *J* = 1.6, 1.0

Hz, 3H, CCH_3) **^{13}C NMR** (126 MHz, Chloroform-*d*) δ 142.9 ($C=CH_2$), 142.0 (C_q), 137.3 (C_q), 129.4 (C_q), 121.9 (*C*H), 120.1 (*C*H), 119.1 (*C*H), 112.0 ($C=CH_2$), 109.3 (*C*H), 104.8 ($CSCH_3$), 33.5 ($CH_2C=CH_2$), 30.3 (NCH_3), 22.8 (CCH_3), 20.5 (SCH_3). **ν_{max}** (neat)/ cm^{-1} 2917, 1466, 1435, 1422, 1391, 1012, 888, 740. **HRMS** (APCI) calculated for $C_{14}H_{18}NS$ ($M+H^+$): 232.1154, found 232.1145.

1-methyl-3-(methylthio)-2-(2-phenylallyl)-1*H*-indole (3ag)

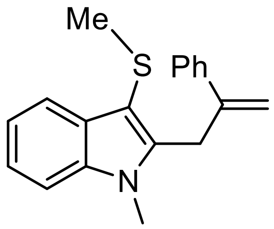

Following general procedure C using (3-(methylsulfinyl)prop-1-en-2-yl)benzene (45.0 mg, 0.250 mmol) afforded the title compound as a pale yellow solid (66.5 mg, 91%).

M.p.: 88-92 °C; **^{1}H NMR** (400 MHz, Chloroform-*d*) δ 7.91 – 7.81 (m, 1H, Ar-*H*), 7.64 – 7.60 (m, 2H, Ar-*H* x 2), 7.46 – 7.41 (m, 2H, Ar-*H* x 2), 7.40 – 7.35 (m, 2H, Ar-*H* x 2), 7.31 (ddd, *J* = 8.1, 7.0, 1.5 Hz, 1H, Ar-*H*), 7.26 (ddd, *J* = 8.1, 7.0, 1.4 Hz, 1H, Ar-*H*), 5.48 (td, *J* = 1.6, 0.9 Hz, 1H, $CH_2C=CH_2$), 4.69 (td, *J* = 1.6, 0.9 Hz, 1H, $CH_2C=CH_2$), 4.23 (t, *J* = 1.6 Hz, 2H, $CH_2C=CH_2$), 3.69 (s, 3H, NCH_3), 2.31 (s, 3H, SCH_3). **^{13}C NMR** (101 MHz, Chloroform-d) δ 145.4 (C_q), 141.7 (C_q), 140.9 (C_q), 137.3 (C_q), 129.4 (C_q), 128.6 (*C*H), 128.1 (*C*H), 126.1 (*C*H), 122.0 (*C*H), 120.2 (*C*H), 119.2 (*C*H), 113.9 ($CH_2CH=CH_2$), 109.3 (*C*H), 105.3 ($CSCH_3$), 30.8 ($CH_2C=CH_2$), 30.5 (NCH_3), 20.6 (SCH_3). **ν_{max}** (neat)/ cm^{-1} 3054, 2917, 1466, 1393, 1341, 1243, 1028, 901, 777, 740, 704. **HRMS** (APCI) calculated for $C_{19}H_{20}NS$ ($M+H^+$): 294.1311, found 294.1307.

Ethyl 2-((1-methyl-3-(methylthio)-1*H*-indol-2-yl)methyl)acrylate (3ah)

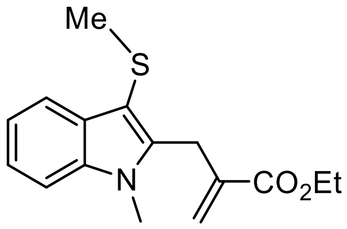

Following general procedure C using ethyl 2-((methylsulfinyl)methyl)acrylate (44.0 mg, 0.250 mmol) afforded the title compound as a pale yellow oil (66.0 mg, 91%).

^{1}H NMR (400 MHz, Chloroform-*d*) δ 7.83 – 7.77 (m, 1H, Ar-*H*), 7.34 (d, *J* = 7.8 Hz, 1H, Ar-*H*), 7.28 (ddd, *J* = 7.8, 7.1, 1.4 Hz, 1H, Ar-*H*), 7.23 (ddd, *J* = 7.4, 7.1, 1.3 Hz, 1H, Ar-*H*), 6.27 (q, *J* = 1.7 Hz, 1H, $CH_2C=CH_2$), 5.11 (q, *J* = 1.7 Hz, 1H, $CH_2C=CH_2$), 4.32 (q, *J* = 7.1 Hz, 2H, OCH_2), 4.06 (t, *J* = 1.7 Hz, 2H, $CH_2C=CH_2$), 3.64 (s, 3H, NCH_3), 2.27 (s, 3H, SCH_3), 1.39 (t, *J* = 7.1 Hz, 3H, OCH_2CH_3). **^{13}C NMR** (101 MHz, Chloroform-d) δ 166.7 ($COOCH_3$), 140.7 (C_q), 138.1 (C_q), 137.3 (C_q), 129.4 (C_q), 126.0 ($CH_2C=CH_2$), 122.1 (*C*H), 120.3 (*C*H), 119.1 (*C*H), 109.4 (*C*H), 105.6 ($CSCH_3$), 61.3 (OCH_2CH_3), 30.4 (NCH_3), 27.0 ($CH_2C=CH_2$), 20.6 (SCH_3), 14.4 (OCH_2CH_3). **ν_{max}** (neat)/ cm^{-1} 2980, 2918, 1712, 1632, 1466, 1274, 1250, 1132, 1026, 941, 738. **HRMS** (APCI) calculated for $C_{16}H_{20}NO_2S$ ($M+H^+$): 220.1209, found 220.1195.

1-Methyl-3-(methylthio)-2-(propa-1,2-dien-1-yl)-1H-indole (3ai)

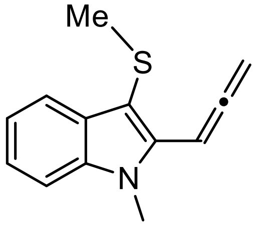

Following general procedure C using methyl propargyl sulfoxide **2a'** (25.5 mg, 0.250 mmol), afforded the title compound as a pale yellow oil (48.4 mg, 90%).

^{1}H NMR (400 MHz, Chloroform-d) δ 7.76 (dt, J = 7.6, 1.0 Hz, 1H, Ar-*H*), 7.33 – 7.25 (m, 2H, Ar-*H* x 2), 7.20 (ddd, J = 8.0, 6.7, 1.4 Hz, 1H, Ar-*H*), 6.96 (t, J = 7.1 Hz, 1H, C*H*=C=CH$_2$), 5.28 (d, J = 7.1 Hz, 2H, CH=C=C*H*$_2$), 3.85 (s, 3H NC*H*$_3$), 2.31 (s, 3H, SC*H*$_3$). **^{13}C NMR** (101 MHz, CDCl$_3$) δ 211.6 (CH=*C*=CH$_2$), 138.2 (*C*$_q$), 135.1 (*C*$_q$), 129.4 (*C*$_q$), 122.7 (*C*H), 120.3 (*C*H), 119.2 (*C*H), 109.3 (*C*H), 106.3 (*C*SCH$_3$), 84.0 (*C*H=C=CH$_2$), 79.0 (CH=CH=*C*H$_2$), 31.6 (N*C*H$_3$), 20.5 (S*C*H$_3$). **ν_{max}** (neat)/ cm^{-1} 3052, 2916, 1938, 1465, 1393, 1338, 1238, 877, 850. **HRMS** (APCI) calculated for $C_{13}H_{14}NS$ (M+H$^+$): 216.0841, found 216.0841.

1-Methyl-3-(phenylthio)-2-(propa-1,2-dien-1-yl)-1H-indole (3aj)

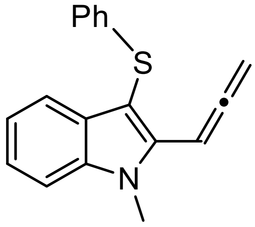

Following general procedure C using phenyl propargyl sulfoxide **2b'** (41.0 mg, 0.250 mmol), purification by column chromatography (10% EtOAc/hexane) afforded the title compound as a yellow oil (27.5 mg, 40%).

^{1}H NMR (400 MHz, Chloroform-*d*) δ 7.60 (dt, *J* = 7.8, 0.9 Hz, 1H, Ar-*H*), 7.36 (dt, *J* = 8.2, 0.9 Hz, 1H, Ar-*H*), 7.30 – 7.26 (m, 1H, Ar-*H*), 7.19 – 7.12 (m, 3H, Ar-*H* x 3), 7.09 – 7.03 (m, 3H, Ar-*H* x 3), 6.86 (t, *J* = 7.1 Hz, 1H, C*H*=C=CH$_2$), 5.24 (d, *J* = 7.1 Hz, 2H, CH=C=C*H*$_2$), 3.93 (s, 3H, NC*H*$_3$). **^{13}C NMR** (101 MHz, CDCl$_3$) δ 211.8 (CH=*C*=CH$_2$), 139.4 (*C*$_q$), 138.5 (*C*$_q$), 136.9 (*C*$_q$), 129.8 (*C*$_q$), 128.9 (*C*H x 2), 125.8 (*C*H x 2), 124.8 (*C*H), 123.0 (*C*H), 120.9 (*C*H), 119.5 (*C*H), 109.4 (*C*H), 100.8 (*C*SAr), 83.9 (*C*H=C=CH$_2$), 79.2 (CH=C=*C*H$_2$), 31.9 (N*C*H$_3$). **ν_{max}** (neat)/ cm^{-1} 3054, 1938, 1581, 1477, 1466, 1336, 1238, 1023, 877, 851. **HRMS** (APCI) calculated for $C_{18}H_{16}NS$ (M+H$^+$): 278.0998, found 278.0996.

1-methyl-3-(methylthio)-2-(1-phenylallyl)-1*H*-indole (3ak) and **1-methyl-2-(methylthio)-3-(1-phenylallyl)-1*H*-indole (4ak)**

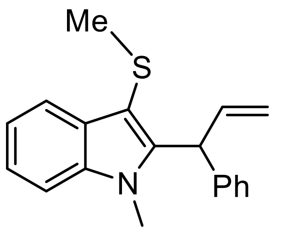

3ai

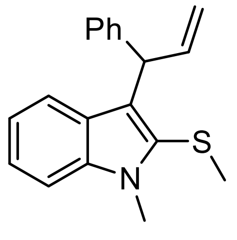

4ai

Following general procedure C using (*E*)-(3-(methylsulfinyl)prop-1-en-1-yl)benzene (45.0 mg, 0.250 mmol) afforded the title compounds as a yellow oil (60.1 mg, 82%) and as a mixture of regioisomers (**3ai** : **4ai** = 1 : 2.7). The ratio of regioisomers was determined by ^{1}H NMR analysis. Analytical samples of **3ai** and **4ai** were obtained by column chromatography (10% EtOAc/hexane).

3ai: **^{1}H NMR** (400 MHz, Chloroform-*d*) δ 7.82 (dd, *J* = 6.9, 1.8 Hz, 1H, Ar-*H*), 7.34 – 7.18 (m, 8H, Ar-*H*), 6.54 (ddd, *J* = 17.1, 10.2, 6.9 Hz, 1H, CHC*H*=CH$_2$), 5.90 (d, *J* = 6.9 Hz, 1H, C*H*CH=CH$_2$), 5.36 (d, *J* = 10.2 Hz, 1H, CHCH=C*H*$_2$), 5.18 (d, *J* = 17.1 Hz, 1H, CHCH=C*H*$_2$), 3.41 (s, 3H, NC*H*$_3$), 2.27 (s, 3H, SC*H*$_3$). **^{13}C NMR** (101 MHz, Chloroform-*d*) δ 144.3 (*C*$_q$), 141.0 (*C*$_q$), 137.6 (*C*$_q$), 136.8 (CH*C*H=CH$_2$), 129.5 (*C*$_q$), 128.3 (*CH*), 127.7 (*CH*), 126.7 (*CH*), 122.2 (*CH*), 120.3 (*CH*), 119.4 (*CH*), 118.5 (CHCH=*C*H$_2$), 109.4 (*CH*), 105.8 (*C*SCH$_3$), 45.2 (*C*HCH=CH$_2$), 31.8

(NCH_3), 20.8 (SCH_3). **ν_{max}** (neat)/ cm^{-1} 3057, 2919, 2853, 1740, 1600, 1466, 1386, 1233, 923, 743, 725, 702. **HRMS** (APCI) calculated for $C_{19}H_{20}NS$ (M+H$^+$): 294.1311, found 294.1297.

4ai: **^{1}H NMR** (400 MHz, Chloroform-*d*) δ 7.44 – 7.36 (m, 1H, Ar-*H*), 7.35 – 7.28 (m, 3H, Ar-*H* x 3), 7.27 – 7.19 (m, 3H, Ar-*H* x 3), 7.19 – 7.13 (m, 1H, Ar-*H*), 6.98 (ddd, *J* = 8.0, 6.9, 1.0 Hz, 1H, Ar-*H*), 6.55 (ddd, J = 17.1, 10.1, 7.2 Hz, 1H, CHC*H*=CH$_2$), 5.44 (d, *J* = 7.2 Hz, 1H, C*H*CH=CH$_2$), 5.21 (dt, *J* = 10.1, 1.5 Hz, 1H, CHCH=C*H$_2$*), 5.12 (dt, *J* = 17.1, 1.6 Hz, 1H, CHCH=C*H$_2$*), 3.88 (s, 3H, NC*H$_3$*), 2.17 (s, 3H, SC*H$_3$*). **^{13}C NMR** (101 MHz, Chloroform-*d*) δ 143.5 (C_q), 140.3 (CH*C*H=CH$_2$), 138.2 (C_q), 130.3 (*C*SCH$_3$), 128.3 (2 x *C*H), 126.1 (*C*H), 125.9 (C_q), 122.7 (*C*H), 122.5 (C_q), 121.1 (*C*H), 119.4 (*C*H), 116.1 (CHCH=*C*H$_2$), 109.8 (*C*H), 47.4 (*C*HCH=CH$_2$), 30.2 (N*C*H$_3$), 20.3 (S*C*H$_3$). **ν_{max}** (neat)/ cm^{-1} 3055, 2920, 1600, 1459, 1367, 1325, 1245, 916, 741, 700. **HRMS** (APCI) calculated for $C_{19}H_{20}NS$ (M+H$^+$): 294.1311, found 294.1298.

1-Isopropyl-2-(methylthio)-3-(1-phenylallyl)-1*H*-indole (4al)

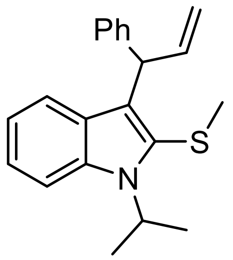

Following general procedure C using (*E*)-(3-(methylsulfinyl)prop-1-en-1-yl)benzene (45.0 mg, 0.250 mmol) and 1-isopropyl-1H-indole (39.8 mg, 0.250 mmol). Purification by column chromatography (10% EtOAc/hexane) afforded the title compound as a pale yellow solid (63.4 mg, 79%).

M.p.: 77-81 °C. **^{1}H NMR** (400 MHz, Chloroform-*d*) δ 7.60 (d, *J* = 8.4 Hz, 1H, Ar-*H*), 7.48 (dd, *J* = 8.1, 2.1 Hz, 1H, Ar-*H*), 7.41 – 7.35 (m, 2H, Ar-*H* x 2), 7.34 – 7.28 (m, 2H, Ar-*H* x 2), 7.25 – 7.17 (m, 2H, Ar-*H* x 2), 7.06 – 6.97 (m, 1H, Ar-*H*), 6.61 (dddd, *J* = 17.0, 10.0, 4.5, 1.6 Hz, 1H, CHC*H*=CH$_2$), 5.57 – 5.51 (m, 1H, C*H*CH=CH$_2$), 5.44 (hept, *J* = 7.1 Hz, 1H, NC*H*(CH$_3$)$_2$), 5.27 (dt, *J* = 10.0, 1.6 Hz, 1H, CHCH=C*H$_2$*), 5.19 (dt, *J* = 17.0, 1.6 Hz, 1H, CHCH=C*H$_2$*), 2.21 (s, 3H, SC*H$_3$*), 1.72 (d, *J* = 7.1 Hz, 3H, NCH(C*H$_3$*)$_2$), 1.71 (d, *J* = 7.1 Hz, 3H, NCH(C*H$_3$*)$_2$). **^{13}C NMR** (101 MHz, Chloroform-*d*) δ 143.5 (C_q), 140.4 (CH*C*H=CH$_2$), 136.0 (C_q), 129.9 (*C*SCH$_3$), 128.3 (*C*H), 128.3 (*C*H), 126.9 (C_q), 126.0 (*C*H), 122.5 (C_q), 122.1 (*C*H), 121.4 (*C*H), 118.8 (*C*H), 116.0 (CHCH=*C*H$_2$), 111.9 (*C*H), 47.7 (*C*HCH=CH$_2$), 47.7 (N*C*H(CH$_3$)$_2$), 21.8 (NCH(*C*H$_3$)$_2$), 20.7 (S*C*H$_3$). **ν_{max}** (neat)/ cm^{-1} 3066, 2973, 2921, 1492, 1448, 1325, 1171, 1162, 1084, 991, 912, 748, 730, 699. **HRMS** (APCI) calculated for $C_{21}H_{24}NS$ (M+H$^+$): 322.1624, found 322.1620.

2-Allyl-3-(phenylthio)-1*H*-indole (SI-3aq)

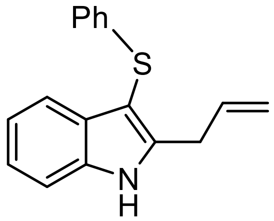

Following general procedure C using (allylsulfinyl)benzene (498 mg, 3.00 mmol), 1*H*-indole (351 mg, 3.00 mmol), trifluoroacetic anhydride (459 µL, 3.30 mmol), and potassium phosphate (1.40 g, 6.60 mmol) in CH_2Cl_2 (30 mL) afforded the title compound as a yellow oil (755 mg, 95%)

^{1}H NMR (400 MHz, Chloroform-*d*) δ 8.28 (br s, 1H, N*H*), 7.60 (dt, *J* = 7.8, 1.1 Hz, 1H, Ar-*H*), 7.38 (dt, *J* = 8.1, 0.9 Hz, 1H, Ar-*H*), 7.24 (ddd, *J* = 8.1, 7.1, 1.1 Hz, 1H, Ar-*H*), 7.21 – 7.14 (m, 3H, Ar-*H* x 3), 7.11 – 7.04 (m, 3H, Ar-*H* x 3), 5.95 (ddt, *J* = 16.7, 10.0, 6.7 Hz, 1H), 5.29 – 5.19 (m, 2H, $CH_2CH=CH_2$), 3.72 (dt, *J* = 6.7, 1.4 Hz, 2H, $CH_2CH=CH_2$). **^{13}C NMR** (101 MHz, Chloroform-d) δ 142.5 (C_q), 139.4 (C_q), 135.7 (C_q), 134.4 ($CH_2CH=CH_2$), 130.4 (C_q), 128.8 (*C*H), 125.7 (*C*H), 124.7 (*C*H), 122.6 (*C*H), 120.9 (*C*H), 119.4 (*C*H), 118.2 ($CH_2CH=CH_2$), 111.0 (*C*H), 99.6 (*C*SPh), 31.1 ($CH_2CH=CH_2$). **ν_{max}** (neat)/ cm^{-1} 3396, 3057, 1638, 1580, 1477, 1453, 1416, 1292, 992, 920, 736. **HRMS** (APCI) calculated for $C_{17}H_{16}NS$ ($M+H^+$): 266.0998, found 266.0987.

General procedure D: Dual vicinal functionalisation of a range of indoles:

A mixture of methyl allyl sulfoxide **2a** (26.0 mg, 0.250 mmol), indole **1** (0.250 mmol), and potassium phosphate (117 mg, 0.550 mmol, 2.2 eq.) in CH_2Cl_2 (2.5 mL) under N_2 was cooled to -78 °C and TFAA (38 µL, 0.275 mmol, 1.1 eq.) was added dropwise. The reaction mixture was stirred at -78 °C for 15 min, then allowed to warm up to room temperature and stirred for 1 h. The crude reaction mixture was filtered through a plug of silica, eluted with CH_2Cl_2, and concentrated *in vacuo* to give the desired products.

2-Allyl-3-(methylthio)-1*H*-indole (3a)

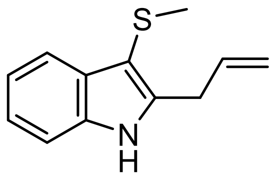

Following general procedure D using 1*H*-indole (29.3 mg, 0.250 mmol) afforded the title compound as a pale yellow oil (44.8 mg, 88%).

^{1}H NMR (400 MHz, Chloroform-*d*) δ 8.07 (br. s, 1H, N*H*), 7.80 – 7.72 (m, 1H, Ar-*H*), 7.34 – 7.30 (m, 1H, Ar-*H*), 7.23 – 7.19 (m, 2H, Ar-*H* x 2), 5.99 (ddt, *J* = 16.9, 10.0, 6.6 Hz, 1H, $CH_2CH{=}CH_2$), 5.23 (dq, J = 16.9, 1.5 Hz, 1H, $CH_2CH{=}CH_2$), 5.22 (dq, J = 10.0, 1.5 Hz, 1H, $CH_2CH{=}CH_2$), 3.75 (dt, *J* = 6.6, 1.5 Hz, 2H, $CH_2CH{=}CH_2$), 2.30 (s, 3H, SCH_3). **^{13}C NMR** (101 MHz, Chloroform-*d*) δ 140.4 (C_q), 135.5 (C_q), 134.9 ($CH_2CH{=}CH_2$), 130.2 (C_q), 122.3 (*C*H), 120.4 (*C*H), 119.1 (*C*H), 117.7 ($CH_2CH{=}CH_2$), 111.0 (*C*H), 104.7 (*C*SCH_3), 31.0 ($CH_2CH{=}CH_2$), 20.2 (SCH_3). **ν_{max}** (neat)/ cm^{-1} 3394, 3057, 2979, 2971, 1637, 1536, 1453, 1417, 1321, 1291, 1230, 1064, 1007, 918, 738. **HRMS** (ESI) calculated for $C_{12}H_{14}NS$ ($M+H^+$): 204.0841, found 204.0843.

2-Allyl-3-(methylthio)-1-(triisopropylsilyl)-1*H*-indole (3c)

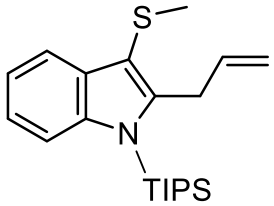

Following general procedure D using 1-(triisopropylsilyl)-1*H*-indole (68.3 mg, 0.250 mmol) afforded the title compound as a pale yellow oil (66.3 mg, 74%).

^{1}H NMR (400 MHz, Chloroform-*d*) δ 7.75 (dd, *J* = 7.8, 1.6 Hz, 1H, Ar-*H*), 7.62 (dd, *J* = 7.9, 1.1 Hz, 1H, Ar-*H*), 7.20 (td, *J* = 7.8, 7.1, 1.1 Hz, 1H, Ar-*H*), 7.15 (ddd, *J* = 7.9, 7.1, 1.6 Hz, 1H, Ar-*H*), 5.96 (ddt, *J* = 17.2, 10.4, 5.4 Hz, 1H, $CH_2CH{=}CH_2$), 5.08 (dq, *J* = 10.4, 1.8 Hz, 1H, $CH_2CH{=}CH_2$), 4.93 (dq, *J* = 17.2, 1.8 Hz, 1H, $CH_2CH{=}CH_2$), 3.95 (dt, *J* = 5.4, 1.8 Hz, 2H, $CH_2CH{=}CH_2$), 2.28 (s, 3H, SCH_3), 1.88 (hept, *J* = 7.6 Hz, 3H, $SiCH(CH_3)_2$ x 3), 1.17 (d, *J* = 7.6 Hz, 18H, $SiCH(CH_3)_2$ x 3). **^{13}C NMR** (101 MHz, Chloroform-*d*) δ 147.5 (C_q), 141.9 (C_q), 136.9 ($CH_2CH{=}CH_2$), 132.7 (C_q), 121.4 (*C*H), 120.4 (*C*H), 118.8 (*C*H), 116.1 ($CH_2CH{=}CH_2$), 114.8 (*C*H), 110.5 (*C*SCH_3), 31.7 ($CH_2CH{=}CH_2$), 19.5 (SCH_3), 19.0 ($SiCH(CH_3)_2$), 14.4 ($SiCH(CH_3)_2$). **ν_{max}** (neat)/ cm^{-1} 2949, 2920, 2868, 1468, 1449, 1267, 1016, 909, 880, 746, 683. **HRMS** (APCI) calculated for $C_{21}H_{34}NSSi$ ($M+H^+$): 360.2176, found 360.2169

2-Allyl-6-bromo-3-(methylthio)-1*H*-indole (3d)

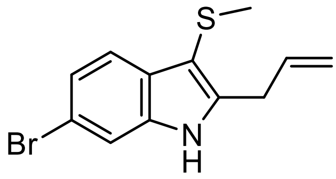

Following general procedure D using 6-bromo-1*H*-indole (48.8 mg, 0.250 mmol) afforded the title compound as a pale yellow oil (64.8 mg, 92%).

^{1}H NMR (500 MHz, Chloroform-*d*) δ 8.05 (br s, 1H, N*H*), 7.57 (d, *J* = 8.4 Hz, 1H, Ar-*H*), 7.43 (d, *J* = 1.7 Hz, 1H, Ar-*H*), 7.28 (dd, *J* = 8.4, 1.7 Hz, 1H, Ar-*H*), 5.96 (ddt, *J* = 17.8, 9.5, 6.5 Hz, 1H, $CH_2CH{=}CH_2$), 5.24 – 5.18 (m, 2H, $CH_2CH{=}CH_2$), 3.70 (dt, *J* = 6.5, 1.4 Hz, 2H, $CH_2CH{=}CH_2$), 2.26 (s, 3H, SCH_3). **^{13}C NMR** (101 MHz, Chloroform-d) δ 141.1 (C_q), 136.2 (C_q), 134.5 ($CH_2CH{=}CH_2$), 129.1 (C_q), 123.7 (*C*H), 120.3 (*C*H), 118.0 ($CH_2CH{=}CH_2$), 115.6 (C_q), 113.9 (*C*H), 105.1 (*C*SCH_3), 31.0 ($CH_2CH{=}CH_2$), 20.2 (SCH_3). **ν_{max}** (neat)/ cm^{-1} 3409, 2918, 1637, 1610, 1450, 1395, 1297, 1045, 921, 804. **HRMS** (ESI) calculated for $C_{12}H_{12}NSBr$ (M^+): 281.9868, found 281.9868.

2-Allyl-6-fluoro-3-(methylthio)-1*H*-indole (3e)

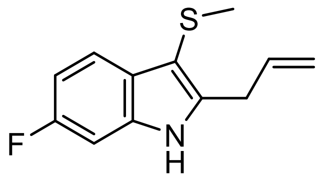

Following general procedure D using 6-fluoro-1*H*-indole (33.8 mg, 0.250 mmol) afforded the title compound as a pink oil (50.9 mg, 92%).

^{1}H NMR (500 MHz, Chloroform-*d*) δ 8.06 (br s, 1H, N*H*), 7.63 (dd, *J* = 8.6, 5.3 Hz, 1H, Ar-*H*), 6.99 (dd, *J* = 9.6, 2.3 Hz, 1H, Ar-*H*), 6.95 (ddd, *J* = 9.6, 8.6, 2.3 Hz, 1H, Ar-*H*), 5.97 (ddt, *J* = 16.8, 10.3, 6.6 Hz, 1H, $CH_2CH{=}CH_2$), 5.26 – 5.17 (m, 2H, $CH_2CH{=}CH_2$), 3.70 (dt, *J* = 6.6, 1.4 Hz, 2H, $CH_2CH{=}CH_2$), 2.27 (s, 3H, SCH_3). **^{13}C NMR** (101 MHz, Chloroform-d) δ 160.1 (d, J = 237.7 Hz, C_q), 140.7 (d, J = 3.4 Hz, C_q), 135.3 (d, J = 12.5 Hz, C_q), 134.8 ($CH_2CH{=}CH_2$), 126.6 (C_q), 119.8 (d, J = 10.0 Hz, *C*H), 117.9 ($CH_2CH{=}CH_2$), 109.0 (d, J = 24.3 Hz, *C*H), 104.8 (*C*SCH_3), 97.6 (d, J = 26.4 Hz, *C*H), 31.1 ($CH_2CH{=}CH_2$), 20.2 (SCH_3). **^{19}F NMR** (376 MHz, Chloroform-d) δ -121.03 (td, J = 9.6, 5.3 Hz). **ν_{max}** (neat)/ cm^{-1} 3408, 3079, 2981, 2919, 1625, 1494, 1451, 1401, 1345, 1220, 1136, 1108, 961, 922, 834, 804. **HRMS** (ESI) calculated for $C_{12}H_{12}NSF$ (M^+): 221.0669, found 221.0673.

Methyl 2-allyl-3-(methylthio)-1H-indole-6-carboxylate (3f)

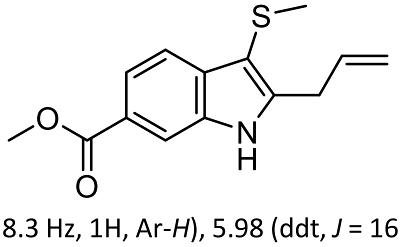

Following general procedure D using methyl 1H-indole-6-carboxylate (43.8 mg, 0.250 mmol) afforded the title compound as a white solid (47.4 mg, 73%).

M.p.: 115-116 °C; **^{1}H NMR** (400 MHz, Chloroform-*d*) δ 8.78 (br s, 1H, N*H*), 8.15 – 8.10 (m, 1H, Ar-*H*), 7.87 (dd, *J* = 8.3, 1.4 Hz, 1H, Ar-*H*), 7.72 (d, *J* = 8.3 Hz, 1H, Ar-*H*), 5.98 (ddt, *J* = 16.8, 10.1, 6.6 Hz, 1H, $CH_2CH{=}CH_2$), 5.21 (dq, J = 16.8, 1.4 Hz, 1H, $CH_2CH{=}CH_2$), 5.20 (dq, J = 10.1, 1.4 Hz, 1H, $CH_2CH{=}CH_2$), 3.94 (s, 3H, OCH_3), 3.75 (dt, *J* = 6.6, 1.4 Hz, 2H, $CH_2CH{=}CH_2$), 2.26 (s, 3H, SCH_3). **^{13}C NMR** (101 MHz, Chloroform-d) δ 168.4 (CO_2CH_3), 144.2 (C_q), 134.9 (C_q), 134.3 ($CH_2CH{=}CH_2$), 134.1 (C_q), 123.8 (C_q), 121.6 (*C*H), 118.6 (*C*H), 118.2 ($CH_2CH{=}CH_2$), 113.4 (*C*H), 105.4 (*C*SCH_3), 52.2 (OCH_3), 31.1 ($CH_2CH{=}CH_2$), 20.2 (SCH_3). **ν_{max}** (neat)/ cm^{-1} 3318, 2916, 1695, 1621, 1436, 1307, 1270, 1217, 1089, 972, 767, 744. **HRMS** (APCI) calculated for $C_{14}H_{16}NO_2S$ ($M+H^+$): 262.0896, found 262.0890.

2-Allyl-7-methyl-3-(methylthio)-1*H*-indole (3g)

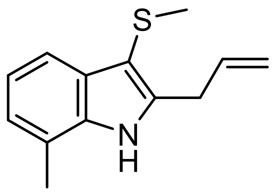

Following general procedure D using 7-methyl-1*H*-indole (32.8 mg, 0.250 mmol) Purification by column chromatography (10% EtOAc/hexane) afforded the title compound as a pale yellow oil (48.3 mg, 89%).

^{1}H NMR (500 MHz, Chloroform-*d*) δ 8.00 (br s, 1H, N*H*), 7.63 (d, *J* = 7.5 Hz, 1H, Ar-*H*), 7.15 (t, *J* = 7.5 Hz, 1H, Ar-*H*), 7.04 (d, *J* = 7.5 Hz, 1H, Ar-*H*), 6.03 (ddt, *J* = 16.6, 10.0, 6.5 Hz, 1H, $CH_2CH{=}CH_2$), 5.29 – 5.22 (m, 2H, $CH_2CH{=}CH_2$), 3.78 (dt, *J* = 6.5, 1.5 Hz, 2H, $CH_2CH{=}CH_2$), 2.51 (s, 3H, Ar-CH_3), 2.31 (s, 3H, SCH_3). **^{13}C NMR** (101 MHz, Chloroform-d) δ 140.0 (C_q), 135.0 (C_q), 135.0 ($CH_2CH{=}CH_2$), 129.7 (C_q), 123.0 (*C*H), 120.6 (*C*H), 120.2 (C_q), 117.6 ($CH_2CH{=}CH_2$), 116.8 (*C*H), 105.3 (*C*SCH_3), 31.1 ($CH_2CH{=}CH_2$), 20.2 (SCH_3), 16.6 (CCH_3). **ν_{max}** (neat)/ cm^{-1} 3406, 2917, 1639, 1539, 1422, 1295, 996, 917, 779, 746. **HRMS** (ESI) calculated for $C_{13}H_{16}NS$ ($M+H^+$): 218.0998, found 218.1001.

2-Allyl-3-(methylthio)-1H-indole-7-carbaldehyde (3h)

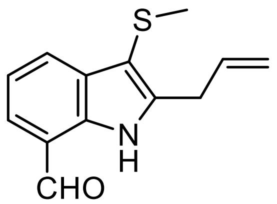

Following general procedure D using 1H-indole-7-carbaldehyde (36.3 mg, 0.250 mmol) and methyl allyl sulfoxide (39.0 mg, 0.375 mmol). Purification by column chromatography (10% EtOAc/hexane) afforded the title compound as a yellow solid (55.2 mg, 96%).

M.p.: 95-98 °C; **^{1}H NMR** (400 MHz, Chloroform-*d*) δ 10.03 (s, 1H, C*H*O), 9.95 (br s, 1H, N*H*), 7.94 (dd, *J* = 7.6, 1.0 Hz, 1H, Ar-*H*), 7.56 (dd, *J* = 7.6, 1.0 Hz, 1H, Ar-*H*), 7.24 (t, *J* = 7.6 Hz, 1H, Ar-*H*), 5.92 (ddt, *J* = 16.7, 10.1, 6.5 Hz, 1H, $CH_2CH{=}CH_2$), 5.15 (dq, J = 16.7, 1.5 Hz, 1H, $CH_2CH{=}CH_2$), 5.14 (dq, J = 10.1, 1.5 Hz, 1H, $CH_2CH{=}CH_2$), 3.69 (dt, *J* = 6.5, 1.5 Hz, 2H, $CH_2CH{=}CH_2$), 2.20 (s, 3H, SCH_3). **^{13}C NMR** (101 MHz, Chloroform-*d*) δ 193.7 (*C*HO), 142.6 (C_q), 134.3 ($CH_2CH{=}CH_2$), 133.5 (C_q), 131.4 (C_q), 128.8 (*C*H), 126.3 (*C*H), 120.4 (C_q), 120.1 (*C*H), 118.0 ($CH_2CH{=}CH_2$), 105.4 (*C*SCH_3), 31.0 ($CH_2CH{=}CH_2$), 20.5 (SCH_3). **ν_{max}** (neat)/ cm^{-1} 3342, 2915, 2820, 1669, 1590, 1532, 1356, 1216, 998, 910, 787, 699. **HRMS** (ESI) calculated for $C_{13}H_{14}NOS$ ($M+H^+$): 232.0791, found 232.0789.

Methyl 2-allyl-3-(methylthio)-1*H*-indole-4-carboxylate (3i)

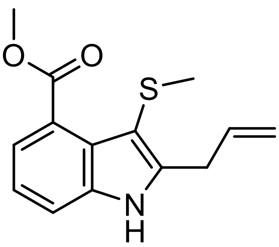

Following general procedure D using methyl 1*H*-indole-4-carboxylate (43.8 mg, 0.250 mmol) and methyl allyl sulfoxide **2a** (39.0 mg, 0.375 mmol). Purification by column chromatography (20% EtOAc/hexane) afforded the title compound as a pale yellow oil (43.6 mg, 67%).

^{1}H NMR (400 MHz, Chloroform-*d*) δ 8.58 (br s, 1H, N*H*), 7.37 (dd, *J* = 8.1, 1.0 Hz, 1H, Ar-*H*), 7.34 (dd, *J* = 7.4, 1.0 Hz, 1H, Ar-*H*), 7.15 (dd, *J* = 8.1, 7.4 Hz, 1H, Ar-*H*), 5.95 (ddt, *J* = 16.8, 10.3, 6.5 Hz, 1H, $CH_2CH{=}CH_2$), 5.19 (dt, J = 16.8, 1.5 Hz, 1H, $CH_2CH{=}CH_2$), 5.18 (dt, J = 10.3, 1.5 Hz, 1H, $CH_2CH{=}CH_2$), 4.00 (s, 3H, OCH_3), 3.74 (dt, *J* = 6.5, 1.5 Hz, 2H, $CH_2CH{=}CH_2$), 2.21 (s, 3H, SCH_3). **^{13}C NMR** (101 MHz, Chloroform-*d*) δ 170.5 (CO_2CH_3), 143.5 (C_q), 136.0 (C_q), 134.5 ($CH_2CH{=}CH_2$), 126.8 (C_q), 125.3 (C_q), 121.3 (*C*H), 120.9 (*C*H), 118.1 ($CH_2CH{=}CH_2$), 113.6 (*C*H), 104.3 (*C*SCH_3), 52.2 (OCH_3), 31.1 ($CH_2CH{=}CH_2$), 21.2 (SCH_3). **ν_{max}** (neat)/ cm^{-1} 3328, 2950, 2919, 1705, 1638, 1431, 1419, 1286, 1197, 1176, 1140, 922, 749. **HRMS** (ESI) calculated for $C_{14}H_{16}NO_2S$ ($M+H^+$): 262.0896, found 262.0887.

2-Allyl-4-bromo-3-(methylthio)-1*H*-indole (3j)

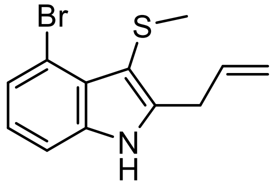

Following general procedure D using 4-bromo-1*H*-indole (48.8 mg, 31.2 µL, 0.250 mmol) affored the title compound as a yellow oil (57.9 mg, 83%).

^{1}H NMR (500 MHz, Chloroform-*d*) δ 8.21 (br s, 1H, N*H*), 7.33 (d, *J* = 7.8 Hz, 1H, Ar-*H*), 7.25 (d, *J* = 7.8 Hz, 1H, Ar-*H*), 7.00 (t, *J* = 7.8 Hz, 1H, Ar-*H*), 5.96 (ddt, J = 17.2, 9.8, 6.6 Hz, 1H, $CH_2CH{=}CH_2$), 5.25 – 5.18 (m, 2H, $CH_2CH{=}CH_2$), 3.77 (dt, *J* = 6.6, 1.5 Hz, 2H, $CH_2CH{=}CH_2$), 2.34 (s, 3H, SCH_3). **^{13}C NMR** (101 MHz, Chloroform-d) δ 142.9 (C_q), 136.7 (C_q), 134.6 ($CH_2CH{=}CH_2$), 127.5 (C_q), 125.5 (*C*H), 123.0 (*C*H), 118.1 ($CH_2CH{=}CH_2$), 114.1 (C_q), 110.4 (*C*H) 105.4 (*C*SCH_3), 31.0 ($CH_2CH{=}CH_2$), 23.0 (SCH_3). **ν_{max}** (neat)/ cm^{-1} 3404, 3079, 2978, 2915, 1638, 1531, 1427, 1414, 1315, 1262, 1179, 922, 773, 737. **HRMS** (ESI) calculated for $C_{12}H_{13}NSBr$ ($M+H^+$): 281.9947, found 281.9950.

2-Allyl-3-(methylthio)-4-(4,4,5,5-tetramethyl-1,3,2-dioxaborolan-2-yl)-1*H*-indole (3k)

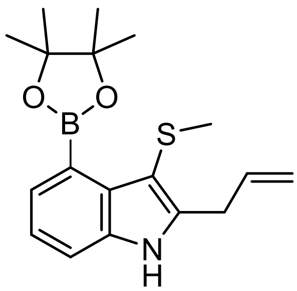

Following general procedure D using 4-(4,4,5,5-tetramethyl-1,3,2-dioxaborolan-2-yl)-1*H*-indole (60.8 mg, 0.250 mmol) afforded the title compound as a white solid (71.0 mg, 86%).

M.p.: 189-191 °C; **^{1}H NMR** (400 MHz, Chloroform-*d*) δ 8.18 (br s, 1H, N*H*), 7.34 (dd, *J* = 7.0, 1.0 Hz, 1H, Ar-*H*), 7.31 (dd, *J* = 8.1, 1.0 Hz, 1H, Ar-*H*), 7.16 (dd, *J* = 8.1, 7.0 Hz, 1H, Ar-*H*), 5.97 (ddt, *J* = 16.7, 10.2, 6.5 Hz, 1H, $CH_2CH{=}CH_2$), 5.20 (dq, J = 16.7, 1.5 Hz, 1H, $CH_2CH{=}CH_2$), 5.19 (dq, J = 10.2, 1.5 Hz, 1H, $CH_2CH{=}CH_2$), 3.74 (dt, *J* = 6.5, 1.5 Hz, 2H, $CH_2CH{=}CH_2$), 2.29 (s, 3H, SCH_3), 1.49 (s, 12H, $C(CH_3)_2$ x 2). **^{13}C NMR** (101 MHz, Chloroform-d) δ 141.6 (C_q), 135.0 ($CH_2CH{=}CH_2$), 134.8 (C_q), 133.0 (C_q), 125.9 (*C*H), 121.5 (*C*H), 117.6 ($CH_2CH{=}CH_2$), 112.3, (*C*H), 105.7 (*C*SCH_3), 84.1 ($C(CH_3)_2$), 31.1 ($CH_2CH{=}CH_2$), 25.1 ($C(CH_3)_2$), 21.4 (SCH_3). C_q adjacent to boron not observed. **ν_{max}** (neat)/ cm^{-1} 3394, 2977, 2918, 1638, 1502, 1369, 1298, 1144, 1132, 977, 854, 756. **HRMS** (ESI) calculated for $C_{18}H_{25}NO_2SB$ ($M+H^+$): 330.1694, found 330.1692.

2-Allyl-5-bromo-3-(methylthio)-1*H*-indole (3l)

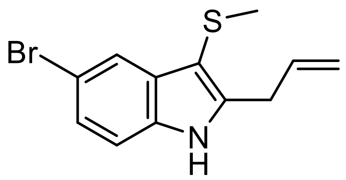

Following general procedure D using 5-bromo-1*H*-indole (48.8 mg, 0.250 mmol) afforded the title compound as a pale yellow oil (62.8 mg, 89%).

^{1}H NMR (400 MHz, Chloroform-*d*) δ 8.22 (br s, 1H, N*H*), 8.04 – 7.92 (m, 1H, Ar-*H*), 7.39 – 7.34 (m, 1H, Ar-*H*), 7.27 (d, *J* = 8.5, 1H, Ar-*H*), 6.07 (ddt, *J* = 17.1, 10.5, 6.6 Hz, 1H, $CH_2CH{=}CH_2$), 5.34 (dq, *J* = 10.5, 1.5 Hz, 1H, $CH_2CH{=}CH_2$), 5.32 (dq, *J* = 17.1, 1.5 Hz, 1H, $CH_2CH{=}CH_2$), 3.82 (dt, *J* = 6.6, 1.5 Hz, 2H, $CH_2CH{=}CH_2$), 2.37 (s, 3H, SCH_3). **^{13}C NMR** (101 MHz, Chloroform-*d*) δ 141.8 (C_q), 134.5 (C_q), 134.1 ($CH_2CH{=}CH_2$), 132.1 (C_q), 125.1 (*C*H), 121.7 (*C*H), 118.1 ($CH_2CH{=}CH_2$), 113.9 (C_q), 112.4 (*C*H), 104.5 (*C*SCH_3), 31.0 ($CH_2CH{=}CH_2$), 20.2 (SCH_3). **ν_{max}** (neat)/ cm^{-1} 3409, 3078, 2979, 2917, 1638, 1566, 1535, 1456, 1441, 1407, 1300, 914, 795. **HRMS** (APCI) calculated for $C_{12}H_{13}NSBr$ ($M+H^+$): 281.9947, found 281.9949.

2-Allyl-5-fluoro-3-(methylthio)-1*H*-indole (3m)

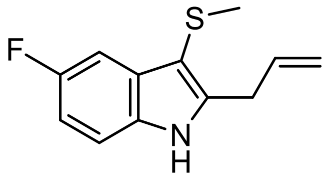

Following general procedure D using 5-fluoro-1*H*-indole (33.8 mg, 0.250 mmol) afforded the title compound as a pale yellow oil (50.3 mg, 92%).

^{1}H NMR (500 MHz, Chloroform-*d*) δ 8.08 (br s, 1H, N*H*), 7.38 (dd, *J* = 9.4, 2.5 Hz, 1H, Ar-*H*), 7.21 (dd, *J* = 8.7, 4.3 Hz, 1H, Ar-*H*), 6.92 (ddd, *J* = 9.4, 8.7, 2.5 Hz, 1H, Ar-*H*), 5.97 (ddt, *J* = 17.7, 9.4, 6.6 Hz, 1H, $CH_2CH{=}CH_2$), 5.25 – 5.20 (m, 2H, $CH_2CH{=}CH_2$), 3.72 (dt, *J* = 6.6, 1.5 Hz, 2H, $CH_2CH{=}CH_2$), 2.26 (s, 3H, SCH_3). **^{13}C NMR** (101 MHz, Chloroform-d) δ 158.6 (d, J = 235.7 Hz, C_q), 142.4 (C_q), 134.6 ($CH_2CH{=}CH_2$), 131.9 (C_q), 131.1 (d, J = 9.8 Hz, C_q), 118.0 ($CH_2CH{=}CH_2$), 111.6 (d, J = 9.6 Hz, *C*H), 110.4 (d, J = 26.4 Hz, *C*H), 104.9 (d, J = 4.5 Hz, *C*SCH_3), 104.2 (d, J = 24.0 Hz, *C*H), 31.1 ($CH_2CH{=}CH_2$), 20.1 (SCH_3). **^{19}F NMR** (376 MHz, Chloroform-d) δ -123.57 (td, J = 9.4, 4.3 Hz). **ν_{max}** (neat)/ cm^{-1} 3412, 3080, 2980, 2919, 1638, 1583, 1483, 1449, 1420, 1275, 1156, 978, 921, 855, 795. **HRMS** (ESI) calculated for $C_{12}H_{12}NSF$ (M^+): 221.0669, found 221.0673.

2-Allyl-3-(methylthio)-5-(trifluoromethyl)-1*H*-indole (3n)

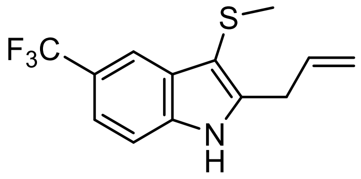

Following general procedure D using 5-(trifluoromethyl)-1*H*-indole (46.3 mg, 0.250 mmol) afforded the title compound as a brown oil (57.9 mg, 85%).

^{1}H NMR (400 MHz, Chloroform-*d*) δ 8.27 (br s, 1H, N*H*), 8.03 (dt, *J* = 1.7, 0.8 Hz, 1H, Ar-*H*), 7.43 (dd, *J* = 8.5, 1.7 Hz, 1H, Ar-*H*), 7.39 – 7.34 (m, 1H, Ar-*H*), 5.98 (ddt, *J* = 16.8, 9.6, 6.6 Hz, 1H, $CH_2CH{=}CH_2$), 5.25 (dq, *J* = 9.6, 1.5 Hz, 1H, $CH_2CH{=}CH_2$), 5.24 (dq, J = 16.8, 1.5 Hz, 1H, $CH_2CH{=}CH_2$), 3.76 (dt, *J* = 6.6, 1.5 Hz, 2H, $CH_2CH{=}CH_2$), 2.29 (s, 3H, SCH_3). **^{13}C NMR** (101 MHz, Chloroform-d) δ 142.4 (C_q), 136.9 (C_q), 134.3 ($CH_2CH{=}CH_2$), 129.8 (C_q), 125.46 (q, J = 271.7 Hz, CF_3), 123.0 (q, J = 31.8 Hz, C_q), 119.1 (q, J = 3.6 Hz, *C*H), 118.3 ($CH_2CH{=}CH_2$), 116.8 (q, J = 4.3 Hz, *C*H), 111.2 (*C*H), 106.0 (*C*SCH_3), 31.0 ($CH_2CH{=}CH_2$), 20.3 (SCH_3). **^{19}F NMR** (376 MHz, Chloroform-d) δ -60.27. **ν_{max}** (neat)/ cm^{-1} 3459, 2921, 1638, 1539, 1420, 1329, 1253, 1233, 1159, 1110, 1042, 917, 893, 809. **HRMS** (ESI) calculated for $C_{13}H_{13}NSF_3$ ($M+H^+$): 272.0715, found 272.0713.

2-Allyl-5-methyl-3-(methylthio)-1*H*-indole (3o)

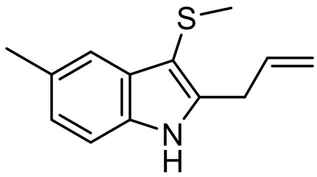

Following general procedure D using 5-methyl-1*H*-indole (32.8 mg, 0.250 mmol) afforded the title compound as a pale yellow oil (49.2 mg, 91%).

^{1}H NMR (400 MHz, Chloroform-*d*) δ 7.98 (br s, 1H, N*H*), 7.55 (d, *J* = 1.6 Hz, 1H, Ar-*H*), 7.21 (d, *J* = 8.2 Hz, 1H, Ar-*H*), 7.04 (dd, *J* = 8.2, 1.6 Hz, 1H, Ar-*H*), 5.99 (ddt, *J* = 16.0, 10.6, 6.5 Hz, 1H, $CH_2CH{=}CH_2$), 5.09 (dq, *J* = 16.0, 1.5 Hz, 1H, $CH_2CH{=}CH_2$), 5.05 (dq, J = 10.6, 1.5 Hz, 1H, $CH_2CH{=}CH_2$), 3.73 (dt, *J* = 6.5, 1.5 Hz, 2H, $CH_2CH{=}CH_2$), 2.51 (s, 3H, Ar-CH_3), 2.30 (s, 3H, SCH_3). **^{13}C NMR** (101 MHz, Chloroform-*d*) δ 140.6 (C_q), 135.1 ($CH_2CH{=}CH_2$), 133.9 (C_q), 130.5 (C_q), 130.0 (C_q), 123.9 (*C*H), 118.9 (*C*H), 117.7 ($CH_2CH{=}CH_2$), 110.8 (*C*H), 104.2 (*C*SCH_3), 31.2 ($CH_2CH{=}CH_2$), 21.8 (Ar-CH_3), 20.4 (SCH_3). **ν_{max}** (neat)/ cm^{-1} 3391, 2978, 2917, 1634, 1417, 1303, 1231, 991, 967, 916, 794, 754. **HRMS** (APCI) calculated for $C_{13}H_{16}NS$ ($M+H^+$): 218.0998, found 218.0999.

2-Allyl-5-methoxy-3-(methylthio)-1*H*-indole (3q)

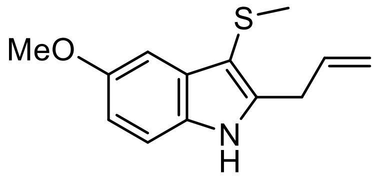

Following general procedure D using 5-methoxy-1*H*-indole (36.8 mg, 0.250 mmol) afforded the title compound as pale yellow oil. (51.4 mg, 88%).

^{1}H NMR (400 MHz, Chloroform-*d*) δ 8.04 (br s, 1H, N*H*), 7.22 – 7.17 (m, 2H, Ar-*H* x 2), 6.85 (dd, *J* = 8.7, 2.5 Hz, 1H, Ar-*H*), 5.97 (ddt, *J* = 16.9, 10.0, 6.5 Hz, 1H, $CH_2CH=CH_2$), 5.21 (dq, *J* = 16.9, 1.6 Hz, 1H, $CH_2CH=CH_2$), 5.20 (dq, J = 10.0, 1.5 Hz, 1H, $CH_2CH=CH_2$), 3.91 (s, 3H, OCH_3), 3.71 (dt, *J* = 6.5, 1.5 Hz, 2H, $CH_2CH=CH_2$), 2.28 (s, 3H, SCH_3). **^{13}C NMR** (101 MHz, Chloroform-*d*) δ 155.0 (C_q), 141.4 (C_q), 135.0 ($CH_2CH=CH_2$), 131.0 (C_q), 130.6 (C_q), 117.8 ($CH_2CH=CH_2$), 112.4 (*C*H), 111.9 (*C*H), 104.4 (*C*SCH_3), 101.0 (*C*H), 56.2 (OCH_3), 31.2 ($CH_2CH=CH_2$), 20.3 (SCH_3). **ν_{max}** (neat)/ cm^{-1} 3395, 2917, 2829, 1636, 1622, 1582, 1482, 1451, 1438, 1283, 1204, 1165, 1025, 992, 913, 838, 793, 753. **HRMS** (APCI) calculated for $C_{13}H_{16}NOS$ ($M+H^+$): 234.0947, found 234.0948.

2-Allyl-5-(benzyloxy)-3-(methylthio)-1*H*-indole (3p)

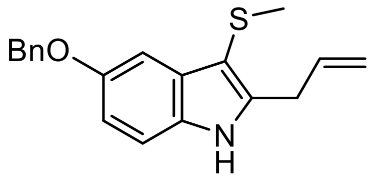

Following general procedure D using 5-(benzyloxy)-1*H*-indole (55.8 mg, 0.250 mmol) and methyl allyl sulfoxide (39.0 mg, 0.375 mmol) afforded the title compound as a pale yellow oil (71.5 mg, 93%).

^{1}H NMR (400 MHz, Chloroform-*d*) δ 7.88 (br s, 1H, N*H*), 7.39 – 7.35 (m, 2H, Ar-*H* x 2), 7.30 – 7.24 (m, 2H, Ar-*H* x 2), 7.23 – 7.18 (m, 1H, Ar-*H*), 7.16 (d, *J* = 2.5 Hz, 1H, Ar-*H*), 7.05 (d, *J* = 8.7 Hz, 1H, Ar-*H*), 6.78 (dd, *J* = 8.7, 2.5 Hz, 1H, Ar-*H*), 5.82 (ddt, *J* = 16.8, 10.3, 6.5 Hz, 1H, $CH_2CH=CH_2$), 5.08 (dt, J = 16.8, 1.5 Hz, 1H, $CH_2CH=CH_2$), 5.07 (dt, *J* = 10.3, 1.5 Hz, 1H, $CH_2CH=CH_2$), 5.01 (s, 2H, OCH_2), 3.55 (dt, *J* = 6.5, 1.5 Hz, 2H, $CH_2CH=CH_2$), 2.11 (s, 3H, SCH_3). **^{13}C NMR** (101 MHz, Chloroform-d) δ 154.0 (C_q), 141.2 (C_q), 137.7 (C_q), 134.9 ($CH_2CH=CH_2$), 130.9 (C_q), 130.6 (C_q), 128.6 (*C*H), 127.9 (*C*H), 127.8 (*C*H), 117.7 ($CH_2CH=CH_2$), 112.9 (*C*H), 111.7 (*C*H), 104.3 (*C*SCH_3), 102.5 (*C*H), 71.0 (OCH_2), 31.1 ($CH_2CH=CH_2$), 20.1 (SCH_3). **ν_{max}** (neat)/ cm^{-1} 3399, 2917, 1621, 1580, 1480, 1450, 1278, 1173, 993, 916, 798, 736, 696. **HRMS** (ESI) calculated for $C_{19}H_{20}NOS$ ($M+H^+$): 310.1260, found 310.1255.

2-Allyl-3-(methylthio)-1*H*-indole-5-carbonitrile (3r)

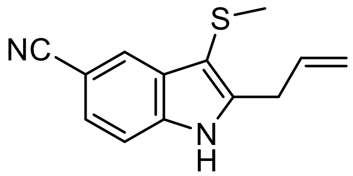

Following general procedure D using 1*H*-indole-5-carbonitrile (35.5 mg, 0.250 mmol) and methyl allyl sulfoxide (39.0 mg, 0.375 mmol) afforded the title compound as a white solid (54.3 mg, 95%).

M.p.: 148-152 °C; **^{1}H NMR** (400 MHz, Chloroform-*d*) δ 8.72 (br s, 1H, N*H*), 8.07 – 8.03 (m, 1H, Ar-*H*), 7.40 – 7.39 (m, 2H, Ar-*H* x 2), 5.97 (ddt, *J* = 17.3, 9.7, 6.7 Hz, 1H, $CH_2CH=CH_2$), 5.26 – 5.20 (m, 2H, $CH_2CH=CH_2$), 3.74 (dt, *J* = 6.7, 1.4 Hz, 2H, $CH_2CH=CH_2$), 2.26 (s, 3H, SCH_3). **^{13}C NMR** (101 MHz, Chloroform-*d*) δ 143.2 (C_q), 137.4 (C_q), 134.1 ($CH_2CH=CH_2$), 130.2 (C_q), 125.3 (*C*H), 124.4 (*C*H), 120.9 (C_q), 118.4 ($CH_2CH=CH_2$), 112.0 (*C*H), 105.9 (C_q), 103.3 (C_q), 30.9 ($CH_2CH=CH_2$), 20.3 (SCH_3). **ν_{max}** (neat)/ cm^{-1} 3272, 2916, 2227, 1637, 1611, 1467, 1423, 1315, 1237, 918, 814, 691. **HRMS** (ESI) calculated for $C_{13}H_{13}N_2S$ ($M+H^+$): 229.0794, found 229.0790.

2-Allyl-3-(methylthio)-1*H*-indole-5-carbaldehyde (3s)

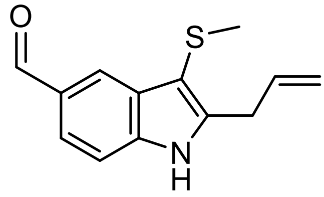

Following general procedure D using 1*H*-indole-5-carbaldehyde (36.3 mg, 0.250 mmol) afforded the title compound as a pale yellow solid (51.2 mg, 89%).

M.p.: 106-110 °C. ^{1}H NMR (400 MHz, Chloroform-*d*) δ 10.07 (s, 1H, C*H*O), 8.91 (br s, 1H, N*H*), 8.25 (d, *J* = 1.6, 1H, Ar-*H*), 7.75 (dd, *J* = 8.4, 1.6 Hz, 1H, Ar-*H*), 7.41 (d, *J* = 8.4 Hz, 1H, Ar-*H*), 5.98 (ddt, *J* = 16.7, 10.3, 6.5 Hz, 1H, $CH_2CH{=}CH_2$), 5.22 (dq, J = 16.7, 1.5 Hz, 1H, $CH_2CH{=}CH_2$), 5.21 (dq, J = 10.3, 1.5 Hz, 1H, $CH_2CH{=}CH_2$), 3.75 (dt, *J* = 6.5, 1.5 Hz, 2H, $CH_2CH{=}CH_2$), 2.29 (s, 3H, SCH_3). ^{13}C NMR (101 MHz, Chloroform-*d*) δ 193.1 (*C*HO), 142.9 (C_q), 139.5 (C_q), 134.4 ($CH_2CH{=}CH_2$), 130.4 (C_q), 130.0 (C_q) , 124.3 (*C*H), 122.9 (*C*H), 118.3 ($CH_2CH{=}CH_2$), 111.9 (*C*H), 106.9 (*C*SCH_3), 31.1 ($CH_2CH{=}CH_2$), 20.5 (SCH_3). **ν_{max}** (neat)/ cm^{-1} 3176, 2913, 1669, 1612, 1576, 1475, 1426, 1300, 1163, 1118, 1000, 967, 914, 894, 801, 759, 724. **HRMS** (APCI) calculated for $C_{13}H_{14}NOS$ ($M+H^+$): 232.0791, found 232.0789.

Methyl 2-allyl-3-(methylthio)-1*H*-indole-5-carboxylate (3t)

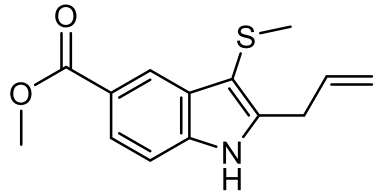

Following general procedure D using methyl 1*H*-indole-5-carboxylate (43.8 mg, 0.250 mmol) afforded the title compound as a white solid (48.5 mg, 74%).

M.p.: 134-135 °C; **^{1}H NMR** (400 MHz, Chloroform-*d*) δ 8.63 (br s, 1H, N*H*), 8.47 (dd, *J* = 1.7, 0.8 Hz, 1H, Ar-*H*), 7.90 (dd, *J* = 8.5, 1.7 Hz, 1H, Ar-*H*), 7.32 (dd, *J* = 8.5, 0.8 Hz, 1H, Ar-*H*), 5.97 (ddt, *J* = 16.8, 10.1, 6.5 Hz, 1H, $CH_2CH{=}CH_2$), 5.20 (dq, *J* = 16.8, 1.5 Hz, 1H, $CH_2CH{=}CH_2$), 5.19 (dq, *J* = 10.1, 1.5 Hz, 1H, $CH_2CH{=}CH_2$), 3.95 (s, 3H, OCH_3), 3.73 (dt, *J* = 6.5, 1.5 Hz, 2H, $CH_2CH{=}CH_2$), 2.27 (s, 3H, SCH_3). **^{13}C NMR** (101 MHz, Chloroform-d) δ 168.4 (CO_2CH_3), 142.1 (C_q), 138.3 (C_q), 134.5 ($CH_2CH{=}CH_2$), 129.9 (C_q), 123.8 (*C*H), 122.5 (C_q), 121.8 (*C*H), 118.1 ($CH_2CH{=}CH_2$), 110.8 (*C*H), 106.3 (*C*SCH_3), 52.1 (OCH_3), 31.0 ($CH_2CH{=}CH_2$), 20.4 (SCH_3). **ν_{max}** (neat)/ cm^{-1} 3286, 2916, 1691, 1613, 1433, 1290, 1234, 993, 915, 769, 743. **HRMS** (APCI) calculated for $C_{14}H_{15}NO_2S$ ($M+H^+$): 262.0896, found 262.0894.

2-Allyl-3-(phenylthio)-1-(triisopropylsilyl)-1*H*-indole (SI-3ar)

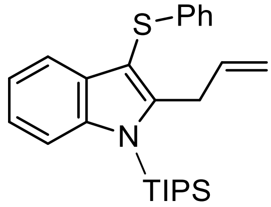

Following general procedure D using 1-(triisopropylsilyl)-1*H*-indole (68.3 mg, 0.250 mmol) and phenylallylsulfoxide (41.5 mg, 0.250 mmol) afforded the title compound as a colourless oil (82.1 mg, 78%).

^{1}H NMR (400 MHz, Chloroform-*d*) δ 7.70 (d, *J* = 7.8 Hz, 1H, Ar-*H*), 7.57 (dd, *J* = 7.7, 1.5 Hz, 1H, Ar-*H*), 7.22 – 7.12 (m, 4H, Ar-*H* x 4), 7.09 – 7.01 (m, 3H, Ar-*H* x 3), 5.89 (ddt, *J* = 17.1, 10.4, 5.6 Hz, 1H, $CH_2CH{=}CH_2$), 5.03 (dq, *J* = 10.4, 1.7 Hz, 1H, $CH_2CH{=}CH_2$), 4.92 (dq, *J* = 17.1, 1.8 Hz, 1H, $CH_2CH{=}CH_2$), 3.92 (dt, *J* = 5.6, 1.8 Hz, 2H, $CH_2CH{=}CH_2$), 1.95 (hept, *J* = 7.5 Hz, 3H, $SiCH(CH_3)_2$ x 3), 1.23 (d, *J* = 7.5 Hz, 18H, $SiCH(CH_3)_2$ x 3). **^{13}C NMR** (101 MHz, Chloroform-*d*) δ 149.5 (C_q), 142.1 (C_q), 139.3 (C_q), 136.2 ($CH_2CH{=}CH_2$), 132.7 (C_q), 128.8 (*C*H), 125.6 (*C*H), 124.5 (*C*H), 121.8 (*C*H), 120.8 (*C*H), 119.3 (*C*H), 116.4 ($CH_2CH{=}CH_2$), 114.9 (*C*H), 105.5 (C_q), 31.8 ($CH_2CH{=}CH_2$), 19.0 ($SiCH(CH_3)_2$), 14.4 ($SiCH(CH_3)_2$). **ν_{max}** (neat)/cm^{-1} 2949, 2868, 1583, 1477, 1470, 1267, 1080, 1016, 908, 879, 734, 687. **HRMS** (APCI) calculated for $C_{26}H_{36}NSSi$ ($M+H^+$): 422.2332, found 422.2321.

General procedure E: Dual vicinal functionalisation of other heteroaromatics:

A solution of methyl allyl sulfoxide (39.0 mg, 0.375 mmol) and heteroarene (0.250 mmol) in CH_2Cl_2 (2.5 mL) containing potassium phosphate (117 mg, 0.550 mmol, 2.2 eq.) under N_2 was cooled to -78 °C and Tf_2O (67 µL, 0.400 mmol, 1.6 eq.) was added dropwise. Reaction mixture was stirred at -78 °C for 15 min, then allowed to warm up to room temperature and stirred for 1 h. Crude reaction mixture was filtered through a plug of silica, eluted with CH_2Cl_2, and concentrated in vacuo to give desired products.

3-allyl-2-(methylthio)thiophene (6a)

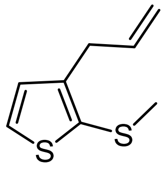

Following general procedure E using thiophene (20 µL, 0.25 mmol) and sodium carbonate (159 mg, 1.5 mmol) afforded the title compound as a yellow oil (27.2 mg, 64%).

^{1}H NMR (400 MHz, Chloroform-*d*) δ 7.28 (d, *J* = 5.4 Hz, 1H, Ar-*H*), 6.90 (d, *J* = 5.4 Hz, 1H, Ar-*H*), 5.95 (ddt, *J* = 17.3, 9.5, 6.6 Hz, 1H, $CH_2CH{=}CH_2$), 5.10 – 5.03 (m, 2H, $CH_2CH{=}CH_2$), 3.49 (dt, *J* = 6.6, 1.5 Hz, 2H, $CH_2CH{=}CH_2$), 2.39 (s, 3H, SCH_3). **^{13}C NMR** (101 MHz, Chloroform-*d*) δ 143.5 (C_q), 136.8 ($CH_2CH{=}CH_2$), 131.3 ($CSCH_3$), 129.2 (*C*H), 127.3 (*C*H), 115.9 ($CH_2CH{=}CH_2$), 33.5 ($CH_2CH{=}CH_2$), 22.4 (SCH_3). **ν_{max}** (neat)/ cm^{-1} 2921, 2852, 1721, 1638, 1429, 1058, 992, 915, 831, 704. **HRMS** (APCI) calculated for $C_8H_{11}S_2$ $(M+H^+)$: 171.0297, found 171.0292.

3-allyl-5-methyl-2-(methylthio)thiophene (6b)

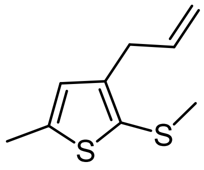

Following general procedure E using 2-methylthiophene (24 µL, 0.25 mmol) afforded the title compound as a pale yellow oil (43.8 mg, 95%).

^{1}H NMR (400 MHz, Chloroform-*d*) δ 6.56 (d, *J* = 1.1 Hz, 1H, Ar-*H*), 5.91 (ddt, *J* = 16.7, 10.1, 6.5 Hz, 1H, $CH_2CH{=}CH_2$), 5.14 – 4.92 (m, 2H, $CH_2CH{=}CH_2$), 3.41 (dt, *J* = 6.5, 1.5 Hz, 2H, $CH_2CH{=}CH_2$), 2.41 (d, *J* = 1.1 Hz, 3H, Ar-CH_3), 2.33 (s, 3H, SCH_3). **^{13}C NMR** (126 MHz, Chloroform-*d*) δ 144.2 (C_q), 142.0 (CCH_3), 137.0 ($CH_2CH{=}CH_2$), 128.0 ($CSCH_3$), 127.5 (*C*H), 115.7 ($CH_2CH{=}CH_2$), 33.5 ($CH_2CH{=}CH_2$), 22.6 (SCH_3), 15.8 (Ar-CH_3). **ν_{max}** (neat)/ cm^{-1} 2978, 2918, 2360, 1638, 1432, 1311, 1136, 992, 967, 912, 830. **HRMS** (APCI) calculated for $C_9H_{13}S$ $(M+H^+)$: 185.0453, found 185.0453.

3-allyl-2-(methylthio)-5-phenylthiophene (6c)

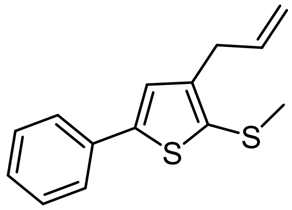

Following general procedure E using 2-phenylthiophene (40.0 mg, 0.250 mmol) and sodium carbonate (159 mg, 1.50 mmol) afforded the title compound as a yellow oil (46.9 mg, 76%).

^{1}H NMR (400 MHz, Chloroform-*d*) δ 7.49 – 7.43 (m, 2H, Ar-*H* x 2), 7.30 – 7.24 (m, 2H, Ar-*H* x 2), 7.21 – 7.13 (m, 1H, Ar-*H*), 7.01 (s, 1H, Ar-*H*), 5.88 (ddt, *J* = 16.6, 10.0, 6.4 Hz, 1H, $CH_2CH{=}CH_2$), 5.05 – 4.97 (m, 2H, $CH_2CH{=}CH_2$), 3.38 (dt, *J* = 6.5, 1.5 Hz, 2H, $CH_2CH{=}CH_2$), 2.32 (s, 3H, SCH_3).**^{13}C NMR** (126 MHz, Chloroform-*d*) δ 145.4 (C_q), 144.6 (C_q), 136.7 ($CH_2CH{=}CH_2$), 134.2 (C_q), 130.8 ($CSCH_3$), 129.0 (*C*H), 127.8 (*C*H), 125.7 (*C*H), 125.1 (*C*H), 116.1 ($CH_2CH{=}CH_2$), 33.7 ($CH_2CH{=}CH_2$), 22.4 (SCH_3). **ν_{max}** (neat)/ cm^{-1} 3059, 2977, 2918, 1637, 1600, 1493, 1449, 1429, 1312, 992, 968, 913, 756, 689. **HRMS** (APCI) calculated for $C_{14}H_{15}S_2$ $(M+H^+)$: 247.0610, found 247.0598.

3-allyl-5-methoxy-2-(methylthio)thiophene (6d)

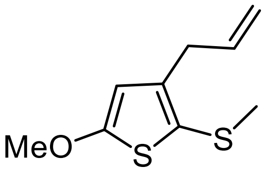

Following general procedure E using 2-methoxythiophene (25 µL, 0.25 mmol) and sodium carbonate (159 mg, 1.50 mmol) afforded the title compound as an orange oil (28.1 mg, 56%).

^{1}H NMR (400 MHz, Chloroform-*d*) δ 5.91 (s, 1H, Ar-*H*), 5.82 (ddt, *J* = 16.7, 10.0, 6.5 Hz, 1H, $CH_2CH=CH_2$), 5.02 – 4.94 (m, 2H, $CH_2CH=CH_2$), 3.76 (s, 3H, OCH_3), 3.32 (dt, *J* = 6.5, 1.5 Hz, 2H, $CH_2CH=CH_2$), 2.21 (s, 3H, SCH_3). **^{13}C NMR** (101 MHz, Chloroform-d) δ 166.7 (C_q), 143.7 (C_q), 136.8 ($CH_2CH=CH_2$), 116.0 (C_q), 115.9 ($CH_2CH=CH_2$), 105.9 (*C*H), 59.9 (OCH_3), 33.8 ($CH_2CH=CH_2$), 22.9 (SCH_3). **ν_{max}** (neat)/ cm^{-1} 2918, 2359, 1637, 1548, 1475, 1422, 1229, 1201, 1150, 993, 914, 789. **HRMS** (APCI) calculated for $C_9H_{13}OS_2$ ($M+H^+$): 201.0402, found 201.0403.

3-allyl-5-bromo-2-(methylthio)thiophene (6e)

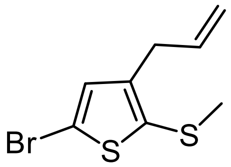

Following general procedure E using 2-bromothiophene (24 µL, 0.25 mmol) and sodium carbonate (159 mg, 1.50 mmol) afforded the title compound as a brown oil (30.4 mg, 49%).

^{1}H NMR (400 MHz, Chloroform-*d*) δ 6.85 (s, 1H, Ar-*H*), 5.94 – 5.82 (m, 1H, $CH_2CH=CH_2$), 5.07 (dt, *J* = 10.5, 1.5 Hz, 1H, $CH_2CH=CH_2$), 5.06 (dt, *J* = 16.7, 1.6 Hz, 1H, $CH_2CH=CH_2$), 3.42 (dt, *J* = 6.5, 1.5 Hz, 2H, $CH_2CH=CH_2$), 2.35 (s, 3H, SCH_3). **^{13}C NMR** (101 MHz, Chloroform-*d*) δ 145.0 (C_q), 136.2 ($CH_2CH=CH_2$), 132.4 (*C*H), 132.0 (C_q), 116.4 ($CH_2CH=CH_2$), 113.0 (C_q), 33.3 ($CH_2CH=CH_2$), 22.5 (SCH_3). **HRMS** (APCI) calculated for $C_8H_{10}S_2Br$ ($M+H^+$): 248.9402, found 248.9394.

4-allyl-5-(methylthio)-2,2'-bithiophene (6f)

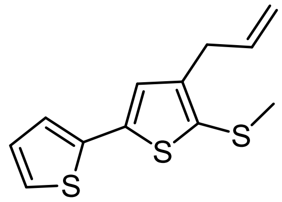

Following general procedure E using 2,2'-bithiophene (41.5 mg, 0.250 mmol), methyl allyl sulfoxide (52.0 mg, 0.500 mmol), and sodium carbonate (159 mg, 1.50 mmol) afforded the title compound as an orange oil (62.3 mg, 98%).

^{1}H NMR (400 MHz, Chloroform-*d*) δ 7.19 (dd, *J* = 3.6, 1.2 Hz, 1H, Ar-*H*), 7.14 (dd, *J* = 3.6, 1.2 Hz, 1H, Ar-*H*), 7.02 – 6.99 (m, 1H, Ar-*H*), 6.97 (s, 1H, Ar-*H*), 5.96 (ddt, *J* = 16.6, 10.1, 6.4 Hz, 1H, $CH_2CH=CH_2$), 5.20 – 5.00 (m, 2H, $CH_2CH=CH_2$), 3.46 (dt, *J* = 6.4, 1.5 Hz, 2H, $CH_2CH=CH_2$), 2.41 (s, 3H, SCH_3). **^{13}C NMR** (101 MHz, Chloroform-*d*) δ 144.4 (C_q), 138.5 (C_q), 137.3 (C_q), 136.5 ($CH_2CH=CH_2$), 130.3 (C_q), 128.0 (*C*H), 125.5 (*C*H), 124.7 (*C*H), 123.9 (*C*H), 116.1 ($CH_2CH=CH_2$), 33.5 ($CH_2CH=CH_2$), 22.3 (SCH_3). **ν_{max}** (neat)/ cm^{-1} 3075, 2977, 2917, 1637, 1510, 1416, 1312, 1241, 1174, 1047, 991, 914, 832, 818, 691. **HRMS** (APCI) calculated for $C_{12}H_{13}S_3$ ($M+H^+$): 253.0173, found 253.0174.

2-(4-allyl-5-(methylthio)thiophen-2-yl)acetonitrile (6g)

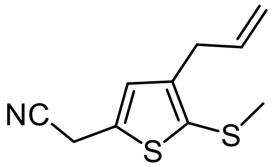

Following general procedure E using 2-(thiophen-2-yl)acetonitrile (27 µL, 0.25 mmol) and sodium carbonate (159 mg, 1.50 mmol) afforded the title compound as a brown oil (37%). Yield was determined by ^{1}H NMR analysis with $MeNO_2$ as internal standard.

^{1}H NMR (400 MHz, Chloroform-*d*) δ 6.84 (t, *J* = 1.1 Hz, 1H, Ar-*H*), 6.01 – 5.78 (m, 1H, $CH_2CH{=}CH_2$), 5.08 – 5.02 (m, 2H, $CH_2CH{=}CH_2$), 3.82 (d, *J* = 1.1 Hz, 2H, CH_2CN), 3.41 (dt, *J* = 6.5, 1.5 Hz, 2H, $CH_2CH{=}CH_2$), 2.36 (s, 3H, SCH_3). **^{13}C NMR** (101 MHz, $CDCl_3$) δ 144.0 (C_q), 136.1 ($CH_2CH{=}CH_2$), 132.1 (C_q), 132.0 ($CSCH_3$), 129.1 (*C*H), 116.6 (CH_2CN), 116.2 ($CH_2CH{=}CH_2$), 33.3 ($CH_2CH{=}CH_2$), 22.2 (SCH_3), 19.0 (CH_2CN). **ν_{max}** (neat)/ cm^{-1} 2980, 2920, 2360, 2342, 1637, 1422, 1256, 1040, 994, 919, 851, 833, 705. **HRMS** (APCI) calculated for $C_{10}H_{12}NS_2$ $(M+H^+)$: 210.0406, found 210.0398.

3-allyl-5-methyl-2-(methylthio)furan (6h)

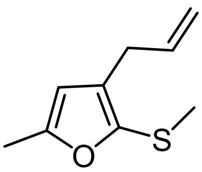

Following general procedure E using 2-methylfuran (22 µL, 0.250 mmol) afforded the title compound as a pale yellow oil (19.9 mg, 47%).

^{1}H NMR (400 MHz, Chloroform-*d*) δ 5.94 – 5.82 (m, 2H, Ar-*H,* and $CH_2CH{=}CH_2$), 5.09 – 5.00 (m, 2H, $CH_2CH{=}CH_2$), 3.20 (dt, *J* = 6.4, 1.6 Hz, 2H, $CH_2CH{=}CH_2$), 2.29 (s, 3H, SCH_3), 2.27 (d, *J* = 1.0 Hz, 3H, Ar-CH_3). **^{13}C NMR** (101 MHz, Chloroform-*d*) δ 154.5 (CCH_3), 141.0 ($CSCH_3$), 136.8 ($CH_2CH{=}CH_2$), 129.5 (C_q), 115.5 ($CH_2CH{=}CH_2$), 109.0 (*C*H), 30.4 ($CH_2CH{=}CH_2$), 19.9 (SCH_3), 14.1 (Ar-CH_3). **ν_{max}** (neat)/ cm^{-1} 2923, 2854, 1764, 1607, 1430, 1251, 1106, 1021, 914, 784. **HRMS** (APCI) calculated for $C_9H_{13}OS$ $(M+H^+)$: 169.0682, found 169.0677.

3-allyl-2-(methylthio)-1*H*-pyrrole (6i)

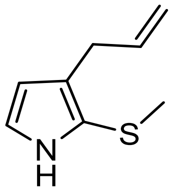

Following general procedure E using freshly distilled 1*H*-pyrrole (17 µL, 0.25 mmol) and sodium carbonate (159 mg, 1.50 mmol). Purification by column chromatography (10% EtOAc/hexane) afforded the title compound as a brown oil (17.5 mg, 46%).

^{1}H NMR (400 MHz, Chloroform-*d*) δ 8.11 (br s, 1H, N*H*), 6.77 (t, *J* = 2.8 Hz, 1H, Ar-*H*), 6.12 (t, *J* = 2.8 Hz, 1H, Ar-*H*), 5.98 (ddt, *J* = 16.8, 10.0, 6.5 Hz, 1H, $CH_2CH{=}CH_2$), 5.07 (dq, *J* = 16.8, 1.6 Hz, 1H, $CH_2CH{=}CH_2$), 5.00 (dq, *J* = 10.0, 1.6 Hz, 1H, $CH_2CH{=}CH_2$), 3.35 (dt, *J* = 6.5, 1.6 Hz, 2H, $CH_2CH{=}CH_2$), 2.24 (s, 3H, SCH_3). **^{13}C NMR** (101 MHz, Chloroform-*d*) δ 138.5 ($CH_2CH{=}CH_2$), 127.3 (C_q), 119.5 (*C*H), 118.2 ($CSCH_3$), 114.7 ($CH_2CH{=}CH_2$), 110.3 (*C*H), 31.1 ($CH_2CH{=}CH_2$), 21.3 (SCH_3). **ν_{max}** (neat)/ cm^{-1} 3395, 2919, 1637, 1546, 1421, 1132, 1070, 993, 968, 912, 726. **HRMS** (APCI) calculated for $C_8H_{12}NS$ $(M+H^+)$: 154.0685, found 154.0680.

tert-Butyl 3-allyl-2-(methylthio)-1_H_-pyrrole-1-carboxylate (6j)

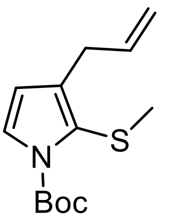

Following general procedure E using _tert_-butyl 1_H_-pyrrole-1-carboxylate (42 µL, 0.250 mmol). Purification by column chromatography (10% EtOAc/hexane) afforded the title compound as a yellow oil (40.0 mg, 63%).

^{1}H NMR (400 MHz, Chloroform-_d_) δ 7.33 (d, _J_ = 3.5 Hz, 1H, Ar-_H_), 6.08 (d, _J_ = 3.6 Hz, 1H, Ar-_H_), 5.90 (ddt, _J_ = 16.6, 10.0, 6.4 Hz, 1H, $CH_2CH{=}CH_2$), 5.09 – 4.98 (m, 2H, $CH_2CH{=}CH_2$), 3.35 (dt, _J_ = 6.5, 1.5 Hz, 2H, $CH_2CH{=}CH_2$), 2.29 (s, 3H, SCH_3), 1.61 (s, 9H, $NCO_2C(CH_3)_3$). **^{13}C NMR** (101 MHz, Chloroform-_d_) δ 148.7 ($NCO_2C(CH_3)_3$), 137.4 ($CH_2CH{=}CH_2$), 134.0 (C_q), 124.3 (_C_H), 122.0 (_C_SCH_3), 115.4 ($CH_2CH{=}CH_2$), 111.8 (_C_H), 83.9 ($OC(CH_3)_3$), 31.5 ($CH_2CH{=}CH_2$), 28.2 ($OC(CH_3)_3$), 20.8 (SCH_3). **ν_{max}** (neat)/ cm^{-1} 2979, 2923, 2360, 1750, 1732, 1341, 1327, 1156, 1091, 985, 912, 851. **HRMS** (APCI) calculated for $C_{13}H_{20}NO_2S$ $(M+H^+)$: 254.1209, found 254.1203.

3-allyl-1-methyl-2-(methylthio)-1H-pyrrole (6k) and **2-allyl-1-methyl-3-(methylthio)-1H-pyrrole (7k)**

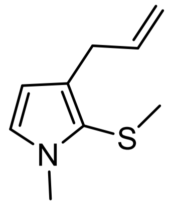

6l

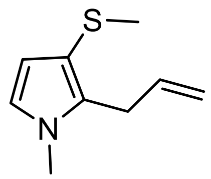

7l

Following general procedure E using freshly distilled 1-methyl-1H-pyrrole (22 µL, 0.25 mmol). Purification by column chromatography (20% EtOAc/hexane) afforded **6l** as a colourless oil (9.0 mg, 22%) and the regioisomer **7l** as a yellow oil (15.2 mg, 36 %)

6l: ^{1}H NMR (400 MHz, Chloroform-_d_) δ 6.72 (d, _J_ = 2.8 Hz, 1H, Ar-_H_), 6.03 (d, _J_ = 2.8 Hz, 1H, Ar-_H_), 5.96 (ddt, _J_ = 16.9, 10.0, 6.6 Hz, 1H, $CH_2CH{=}CH_2$), 5.06 (dq, _J_ = 16.9, 1.7 Hz, 1H, $CH_2CH{=}CH_2$), 4.98 (dq, _J_ = 10.0, 1.7 Hz, 1H, $CH_2CH{=}CH_2$), 3.69 (s, 3H, NCH_3), 3.35 (dt, _J_ = 6.6, 1.7 Hz, 2H, $CH_2CH{=}CH_2$), 2.15 (s, 3H, SCH_3). **^{13}C NMR** (101 MHz, Chloroform-_d_) δ 138.8 ($CH_2CH{=}CH_2$), 128.4 (C_q), 124.0 (_C_H), 120.4 (_C_SCH_3), 114.6 ($CH_2CH{=}CH_2$), 108.3 (_C_H), 34.2 (NCH_3), 32.0 ($CH_2CH{=}CH_2$), 20.7 (SCH_3). **ν_{max}** (neat)/ cm^{-1} 2920, 2854, 2362, 2340, 1637, 1424, 1234, 909, 725. **HRMS** (APCI) calculated for $C_9H_{14}NS$ $(M+H^+)$: 168.0841, found 168.0837.

7l: ^{1}H NMR (400 MHz, Chloroform-_d_) δ 6.54 (d, _J_ = 2.8 Hz, 1H, Ar-_H_), 6.20 (d, _J_ = 2.8 Hz, 1H, Ar-_H_), 5.87 (ddt, _J_ = 17.0, 10.1, 5.7 Hz, 1H, $CH_2CH{=}CH_2$), 5.06 (dq, _J_ = 8.4, 1.7 Hz, 1H, $CH_2CH{=}CH_2$), 4.92 (dq, _J_ = 17.0, 1.8 Hz, 1H, $CH_2CH{=}CH_2$), 3.55 – 3.48 (m, 5H, NCH_3 and $CH_2CH{=}CH_2$), 2.25 (s, 3H, SCH_3). **^{13}C NMR** (101 MHz, Chloroform-_d_) δ 135.9 ($CH_2CH{=}CH_2$), 133.1 (C_q), 121.4 (_C_H), 115.8 ($CH_2CH{=}CH_2$), 112.0 (_C_SCH_3), 111.8 (_C_H), 34.5 (NCH_3), 29.0 ($CH_2CH{=}CH_2$), 21.7 (SCH_3). **ν_{max}** (neat)/ cm^{-1} 2920, 2854, 1637, 1495, 1424, 1305, 1158, 966, 914, 713. **HRMS** (APCI) calculated for $C_9H_{14}NS$ $(M+H^+)$: 168.0841, found 168.0837.

Methyl 4-allyl-5-(methylthio)-1*H*-pyrrole-2-carboxylate (6l) and **methyl 5-allyl-4-(methylthio)-1*H*-pyrrole-2-carboxylate (7l)**

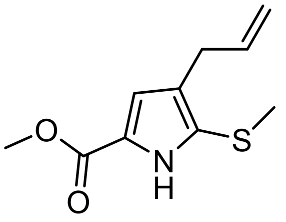

6l

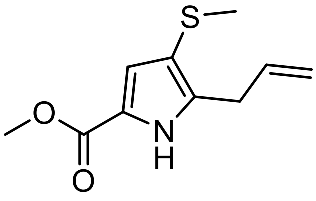

7l

Following general procedure E using methyl 1*H*-pyrrole-2-carboxylate (31.5 mg, 0.250 mmol) afforded the title compounds as a yellow oil (17.0 mg, 47%) and as a mixture of regioisomers (**6l** : **7l** = 2 : 1)

6m: ^{1}**H NMR** (400 MHz, Chloroform-*d*) δ 9.11 (br s, 1H, N*H*), 6.75 (d, *J* = 2.7 Hz, 1H, Ar-*H*), 5.92 (dtt, *J* = 16.7, 10.2, 6.4 Hz, 1H, $CH_2CH{=}CH_2$), 5.08 – 5.00 (m, 2H, $CH_2CH{=}CH_2$), 3.84 (s, 3H, OCH_3), 3.30 (dt, *J* = 6.4, 1.6 Hz, 2H, $CH_2CH{=}CH_2$), 2.30 (s, 3H, SCH_3). ^{13}**C NMR** (101 MHz, Chloroform-*d*) δ 161.1 (CO_2CH_3), 137.5 ($CH_2CH{=}CH_2$), 128.4 (C_q), 125.5 ($CSCH_3$), 123.3 (C_q), 116.1 (*C*H), 115.4 ($CH_2CH{=}CH_2$), 51.7 (OCH_3), 30.9 ($CH_2CH{=}CH_2$), 20.3 (SCH_3).

7m: ^{1}**H NMR** (400 MHz, Chloroform-*d*) δ 9.05 (br s, 1H, N*H*), 6.92 (d, *J* = 2.6 Hz, 1H, Ar-*H*), 5.92 (dtt, *J* = 16.7, 10.2, 6.4 Hz, 1H, $CH_2CH{=}CH_2$), 5.22 – 5.11 (m, 2H, $CH_2CH{=}CH_2$), 3.84 (s, 3H, OCH_3), 3.50 (dt, *J* = 6.6, 1.4 Hz, 2H, $CH_2CH{=}CH_2$), 2.27 (s, 3H, SCH_3). ^{13}**C NMR** (101 MHz, Chloroform-*d*) δ 161.3 (CO_2CH_3), 137.5 (C_q), 134.1 ($CH_2CH{=}CH_2$), 121.3 (C_q), 119.8 (*C*H), 118.1 ($CH_2CH{=}CH_2$), 114.7 ($CSCH_3$), 51.7 (OCH_3), 30.6 ($CH_2CH{=}CH_2$), 20.7 (SCH_3).

6m and **7m**: $\mathbf{v_{max}}$ (neat)/ cm^{-1} 3290, 2950, 2921, 1682, 1638, 1439, 1406, 1210, 1008, 914, 769. **HRMS** (ESI) calculated for $C_{10}H_{14}NO_2S$ ($M+H^+$): 212.0740, found 212.0734.

2-Allyl-3-(phenylthio)benzo[b]thiophene (6m)

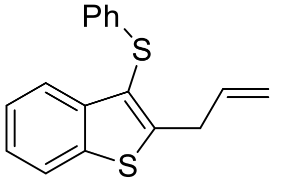

Following general procedure E using benzo[*b*]thiophene (33.5 mg, 0.250 mmol) and phenyl allyl sulfoxide (62.3 mg, 0.375 mmol), purification by column chromatography (20% $CHCl_3$/hexane) afforded the title compound as a colourless oil (30.3 mg, 43%)

^{1}**H NMR** (400 MHz, Chloroform-*d*) δ 7.87 – 7.74 (m, 2H, Ar-*H* x 2), 7.36 – 7.31 (m, 2H, Ar-*H* x 2), 7.18 (dd, *J* = 8.3, 6.9 Hz, 2H, Ar-*H* x 2), 7.11 – 7.06 (m, 1H, Ar-*H*), 7.06 – 7.01 (m, 2H, Ar-*H* x 2), 5.98 (ddt, *J* = 16.8, 9.9, 6.6 Hz, 1H, $CH_2CH{=}CH_2$), 5.20 (dq, *J* = 16.8, 1.5 Hz, 1H, $CH_2CH{=}CH_2$), 5.13 (dq, *J* = 9.9, 1.5 Hz, 1H, $CH_2CH{=}CH_2$), 3.88 (dt, *J* = 6.6, 1.5 Hz, 2H, $CH_2CH{=}CH_2$). ^{13}**C NMR** (101 MHz, $CDCl_3$) δ 150.6 (C_q), 140.6 (C_q), 138.4 (C_q), 137.4 (C_q), 135.4 ($CH_2CH{=}CH_2$), 129.1 (*C*H x 2), 126.5 (*C*H x 2), 125.4 (*C*H), 125.0 (*C*H), 124.8 (*C*H), 123.3 (*C*H), 122.5 (*C*H), 119.5 (C_q), 117.5 ($CH_2CH{=}CH_2$), 34.1 ($CH_2CH{=}CH_2$). $\mathbf{v_{max}}$ (neat)/ cm^{-1} 3057, 2361, 2341, 1639, 1581, 1477, 1432, 1082, 1024, 917. **HRMS** (APCI) calculated for $C_{17}H_{15}S_2$ ($M+H^+$): 283.0610, found 283.0610.

3-Allyl-2-(phenylthio)benzofuran (6n)

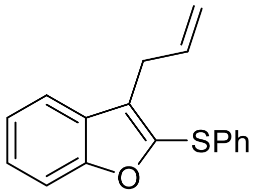

Following general procedure E using 2,3-benzofuran (27 µL, 0.25 mmol) and phenyl allyl sulfoxide (62.3 mg, 0.375 mmol), purification by column chromatography (20% $CHCl_3$/hexane) afforded the title compound as a pale yellow oil (32%). Yield was determined by ^{1}H NMR analysis with $MeNO_2$ as internal standard.

^{1}H NMR (400 MHz, Chloroform-*d*) δ 7.63 – 7.58 (m, 1H, Ar-*H*), 7.48 (d, *J* = 8.3 Hz, 1H, Ar-*H*), 7.35 (ddd, *J* = 8.3, 7.2, 1.4 Hz, 1H, Ar-*H*), 7.30 – 7.24 (m, 5H, Ar-*H*), 7.23 – 7.18 (m, 1H, Ar-*H*), 5.98 (ddt, *J* = 16.6, 10.0, 6.2 Hz, 1H, CH_2CH=CH_2), 5.16 (dq, *J* = 16.6, 1.6 Hz, 1H, CH_2CH=CH_2), 5.10 (dq, *J* = 10.0, 1.6 Hz, 1H, CH_2CH=CH_2), 3.63 (dt, *J* = 6.2, 1.6 Hz, 2H, CH_2CH=CH_2). **^{13}C NMR** (101 MHz, $CDCl_3$) δ 156.2 (C_q), 144.0 (C_q), 135.3 (C_q), 135.2 (CH_2CH=CH_2), 129.3 (*C*H x 2), 128.7 (C_q), 128.3 (*C*H x 2), 126.8 (*C*H), 125.9 (C_q), 125.7 (*C*H), 122.8 (*C*H), 120.5 (*C*H), 116.6 (CH_2CH=CH_2), 111.7 (*C*H), 29.4 (CH_2CH=CH_2). **ν_{max}** (neat)/ cm^{-1} 3061, 2361, 1581, 1478, 1446, 1232, 1075, 915. **HRMS** (APCI) calculated for $C_{17}H_{15}SO$ (M+H$^+$): 267.0838, found 267.0838.

2-Allyl-3-(allylthio)-1H-indole (3am)

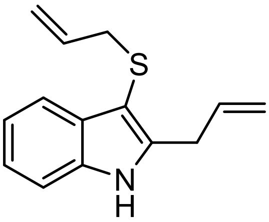

To a solution of diallyl sulfoxide (1.30 g, 10.0 mmol) and 1-*H*-indole (1.17 g, 10.0 mmol) in CH_2Cl_2 (100 mL) containing potassium phosphate (4.66 g, 22.0 mmol) under N_2 was cooled to -78 °C and TFAA (1.53 mL, 11.0 mmol) was added dropwise. The reaction mixture was stirred at -78 °C for 15 min, then allowed to warm up to room temperature, and stirred for 1 h. The crude reaction mixture was filtered through a plug of silica, and eluted with CH_2Cl_2. Solvent was removed *in vacuo* to give title compound as a yellow oil (2.15 g, 94%).

^{1}H NMR (400 MHz, Chloroform-*d*) δ 8.10 (s, 1H), 7.83 – 7.67 (m, 1H), 7.34 – 7.29 (m, 1H), 7.23 – 7.17 (m, 2H), 5.96 (ddt, *J* = 16.8, 10.1, 6.5 Hz, 1H, CH_2CH=CH_2), 5.86 (ddt, *J* = 17.1, 10.0, 7.4 Hz, 1H, SCH_2CH=CH_2), 5.28 – 5.19 (m, 2H, CH_2CH=CH_2), 4.90 (ddd, *J* = 10.0, 1.5, 0.9 Hz, 1H, SCH_2CH=CH_2), 4.80 (dq, *J* = 17.1, 1.5 Hz, 1H, SCH_2CH=CH_2), 3.72 (dt, *J* = 6.5, 1.4 Hz, 2H, CH_2CH=CH_2), 3.29 (dt, *J* = 7.4, 0.9 Hz, 2H, SCH_2CH=CH_2). **^{13}C NMR** (101 MHz, Chloroform-*d*) δ 141.9 (C_q), 135.5 (C_q), 135.1 (CH_2CH=CH_2), 134.9 (SCH_2CH=CH_2), 130.7 (C_q), 122.2 (*C*H), 120.5 (*C*H), 119.2 (*C*H), 117.8 (CH_2CH=CH_2), 116.8 (SCH_2CH=CH_2), 110.9 (*C*H), 102.0 ($CSCH_2CH$=CH_2), 39.5 (SCH_2CH=CH_2), 31.2 (CH_2CH=CH_2). **HRMS** (APCI) calculated for $C_{14}H_{16}NS$ (M+H$^+$): 230.0998, found 230.0989.

2-Allyl-3-(allylsulfinyl)-1H-indole (2d)

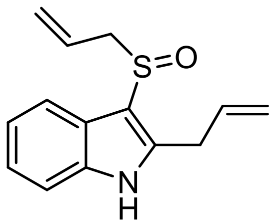

To a solution of 2-allyl-3-(allylthio)-1*H*-indole (2.13 g, 9.30 mmol) in CH_2Cl_2 (100 mL) at 0 °C was added *m*CPBA (2.24 g, 10.0 mmol, 77% wt.) in several portions. The reaction mixture was warmed to room temperature and stirred for 1 h. To the reaction mixture was added aq. NaOH (50 mL, 0.5 M). The organic phase was separated, washed with aq. NaOH (50mL, 0.5 M) and water (50 mL), dried over $NaSO_4$, and filtered. The solvent was removed *in vacuo* and the resulting solid was recrystallised from EtOAc/hexane to give title compound as an off-white solid (1.93 g, 85%).

M.p.: 98-100 °C; **^{1}H NMR** (400 MHz, Chloroform-*d*) δ 9.25 (br s, 1H, N*H*), 8.02 (dd, *J* = 6.9, 2.0 Hz, 1H, Ar-*H*), 7.38 – 7.31 (m, 1H, Ar-*H*), 7.19 – 7.11 (m, 2H, Ar-*H* x 2), 5.83 (ddt, *J* = 16.6, 9.7, 6.5 Hz, 1H, $CH_2CH=CH_2$), 5.61 (ddt, *J* = 17.6, 10.3, 7.5 Hz, 1H, $SCH_2CH=CH_2$), 5.25 – 5.17 (m, 2H, $SCH_2CH=CH_2$), 5.16 – 5.09 (m, 2H, $CH_2CH=CH_2$), 4.06 (d, *J* = 7.5 Hz, 2H, $SCH_2CH=CH_2$), 3.55 (dd, *J* = 6.5, 1.6 Hz, 2H, $CH_2CH=CH_2$). **^{13}C NMR** (101 MHz, Chloroform-d) δ 141.6 (*C*$_q$), 136.3 (*C*$_q$), 133.8 ($CH_2CH=CH_2$), 127.1 ($SCH_2CH=CH_2$), 124.8 (*C*$_q$), 123.1 ($SCH_2CH=CH_2$), 123.0 (*C*H), 121.3 (*C*H), 119.7 (*C*H), 118.2 ($CH_2CH=CH_2$), 112.1 (*C*H), 109.4 ($CS(O)CH_2CH=CH_2$), 57.5 ($SCH_2CH=CH_2$), 30.9 ($CH_2CH=CH_2$). **ν_{max}** (neat)/ cm^{-1} 3168, 1638, 1531, 1420, 1012, 992, 925, 743. **HRMS** (ESI) calculated for $C_{14}H_{16}NOS$ (M+H$^+$): 246.0947, found 246.0941.

2-Allyl-3-(allylsulfinyl)-1-methyl-1*H*-indole (2e)

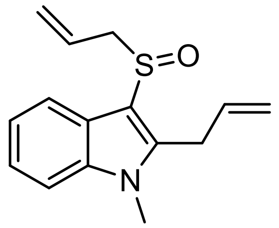

To a solution of 2-allyl-3-(allylthio)-1-methyl-1*H*-indole (1.68 g, 6.90 mmol) in CH_2Cl_2 (100 mL) at 0 °C was added *m*CPBA (1.62 g, 7.25 mmol, 77% wt.) in several portions. The reaction mixture was warmed to room temperature and stirred for 1 h. To the reaction mixture was added aq. NaOH (50 mL, 0.5 M). The organic phase was separated, washed with aq. NaOH (50mL, 0.5 M) and water (50 mL), dried over $NaSO_4$, and filtered. The solvent was removed *in vacuo* and the crude product was purified by column chromatography (EtOAc) to afford the title compound as a yellow solid (1.28 g, 72%).

M.p.: 71-74 °C; **^{1}H NMR** (400 MHz, Chloroform-*d*) δ 8.08 (dt, *J* = 7.8, 1.0 Hz, 1H, Ar-*H*), 7.35 (dt, *J* = 8.3, 0.9 Hz, 1H, Ar-*H*), 7.29 (ddd, *J* = 8.2, 7.0, 1.2 Hz, 1H, Ar-*H*), 7.22 (ddd, *J* = 8.1, 7.0, 1.2 Hz, 1H, Ar-*H*), 5.88 (ddt, *J* = 17.2, 10.2, 5.7 Hz, 1H, $CH_2CH=CH_2$), 5.63 (ddt, *J* = 17.3, 9.8, 7.5 Hz, 1H, $SCH_2CH=CH_2$), 5.26 – 5.18 (m, 2H, $SCH_2CH=CH_2$), 5.16 (dq, *J* = 10.2, 1.7 Hz, 1H, $CH_2CH=CH_2$), 4.95 (dq, *J* = 17.2, 1.7 Hz, 1H, $CH_2CH=CH_2$), 4.10 – 3.99 (m, 2H, $SCH_2CH=CH_2$), 3.73 (ddt, *J* = 5.7, 3.8, 1.7 Hz, 2H, $CH_2CH=CH_2$), 3.67 (s, 3H, NCH_3). **^{13}C NMR** (101 MHz, Chloroform-d) δ 141.4 (*C*$_q$), 137.8 (*C*$_q$), 133.7 ($CH_2CH=CH_2$), 127.4 ($SCH_2CH=CH_2$), 124.3 (*C*$_q$), 122.9 (*C*H), 122.9 ($SCH_2CH=CH_2$), 121.5 (*C*H), 120.0 (*C*H), 117.6 ($CH_2CH=CH_2$), 110.6 ($CS(O)CH_2CH=CH_2$), 110.0 (*C*H), 58.1 ($SCH_2CH=CH_2$), 30.1 (NCH_3), 29.2 ($CH_2CH=CH_2$). **ν_{max}** (neat)/ cm^{-1} 3051, 2977, 1636, 1520, 1469, 1394, 1339, 1028, 992, 918, 745, 736. **HRMS** (ESI) calculated for $C_{15}H_{18}NOS$ (M+H$^+$): 260.1104, found 260.1095.

Bis(2-allyl-1H-indol-3-yl)sulfide (3an)

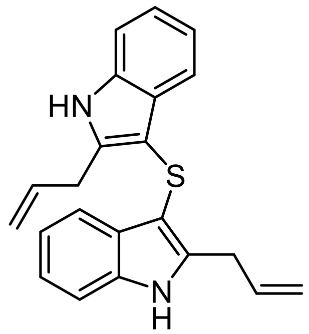

Following general procedure D using 2-allyl-3-(allylsulfinyl)-1H-indole (61.3 mg, 0.250 mmol) and 1-*H*-indole (29.3 mg, 0.250 mmol). Purification by column chromatography (50% CH_2Cl_2/hexane) afforded the title compound as a pale yellow solid (41.9 mg, 49%).

^{1}H NMR (400 MHz, Chloroform-*d*) δ 7.94 (br s, 2H, N*H* x 2), 7.82 – 7.69 (m, 2H, Ar-*H* x 2), 7.24 – 7.19 (m, 2H, Ar-*H* x 2), 7.16 – 7.08 (m, 4H, Ar-*H* x 4), 5.94 (ddt, *J* = 16.8, 10.1, 6.6 Hz, 2H, $CH_2CH=CH_2$ x 2), 5.27 – 5.18 (m, 4H, $CH_2CH=CH_2$ x 2), 3.87 (dt, *J* = 6.6, 1.4 Hz, 4H, $CH_2CH=CH_2$ x 2). **^{13}C NMR** (101 MHz, Chloroform-*d*) δ 139.5 (*C*$_q$), 135.3 (*C*$_q$), 134.6 ($CH_2CH=CH_2$), 130.3 (*C*$_q$), 122.0 (*C*H), 120.3 (*C*H), 119.4 (*C*H), 118.0 ($CH_2CH=CH_2$), 110.8 (*C*H), 104.4 (*C*S), 31.3 ($CH_2CH=CH_2$). **ν_{max}** (neat)/ cm^{-1} 3390, 1637, 1453, 1409, 1291, 1230, 991, 910, 734. **HRMS** (APCI) calculated for $C_{22}H_{21}N_2S$ (M+H$^+$): 345.1420, found 345.1417.

2-Allyl-3-((2-allyl-1*H*-indol-3-yl)thio)-1-methyl-1*H*-indole (3ao)

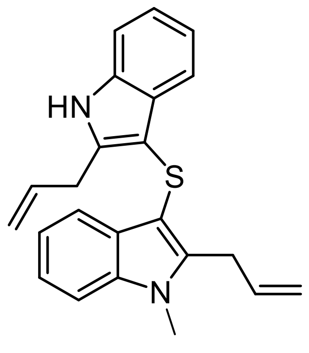

Following general procedure D using 2-allyl-3-(allylsulfinyl)-1-methyl-indole (64.8 mg, 0.250 mmol) and 1-*H*-indole (29.3 mg, 0.250 mmol). Purification by column chromatography (20% EtOAc/hexane) afforded the title compound as a yellow oil (60.0 mg, 67%).

^{1}H NMR (400 MHz, Chloroform-*d*) δ 8.04 (br s, 1H, N*H*), 7.94 (dt, *J* = 7.6, 0.8 Hz, 1H, Ar-*H*), 7.88 – 7.81 (m, 1H, Ar-*H*), 7.38 – 7.28 (m, 3H, Ar-*H* x 3), 7.28 – 7.17 (m, 3H, Ar-*H* x 3), 6.07 (ddt, *J* = 16.8, 10.0, 6.7 Hz, 1H, $CH_2CH=CH_2$), 5.93 (ddt, *J* = 17.1, 10.1, 5.7 Hz, 1H, $CH_2CH=CH_2$), 5.38 – 5.30 (m, 2H, $CH_2CH=CH_2$), 5.17 (dq, *J* = 10.1, 1.6 Hz, 1H, $CH_2CH=CH_2$), 5.04 (dq, *J* = 17.1, 1.6 Hz, 1H, $CH_2CH=CH_2$), 4.05 (dt, *J* = 5.7, 1.6 Hz, 2H, $CH_2CH=CH_2$), 3.98 (dt, *J* = 6.7, 1.4 Hz, 2H, $CH_2CH=CH_2$), 3.73 (s, 3H, NCH_3). **^{13}C NMR** (101 MHz, Chloroform-*d*) δ 141.0 (C_q), 139.2 (C_q), 137.0 (C_q), 135.3 (C_q), 134.6 ($CH_2CH=CH_2$), 134.6 ($CH_2CH=CH_2$), 130.3 (C_q), 129.7 (C_q), 121.9 (*C*H), 121.6 (*C*H), 120.2 (*C*H), 120.0 (*C*H), 119.6 (*C*H), 119.5 (*C*H), 118.0 ($CH_2CH=CH_2$), 116.6 ($CH_2CH=CH_2$), 110.7 (*C*H), 109.1 (*C*H), 104.9 (C_q), 104.0 (C_q), 31.4 ($CH_2CH=CH_2$), 30.4 (NCH_3), 29.7 ($CH_2CH=CH_2$). **ν_{max}** (neat)/ cm^{-1} 3393, 3053, 2930, 1636, 1521, 1465, 1454, 1416, 1390, 1340, 1292, 992, 911, 734. **HRMS** (ESI) calculated for $C_{23}H_{22}N_2S$ (M^+): 358.1504, found 358.1503.

2-Allyl-3-((2-allyl-1-methyl-1H-indol-3-yl)thio)-5-bromo-1*H*-indole (3ap)

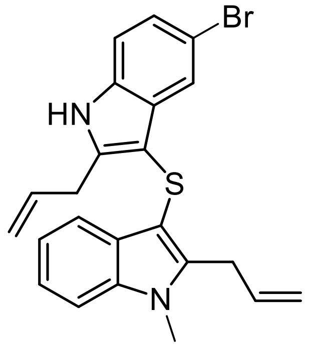

Following general procedure D using 2-allyl-3-(allylsulfinyl)-1-methyl-indole (64.8 mg, 0.250 mmol) and 5-bromo-1-*H*-indole (49.6 mg, 0.250 mmol). Purification by column chromatography (20% EtOAc/hexane) afforded the title compound as a yellow solid (55.9 mg, 51%).

M.p.: 126-129 °C; **^{1}H NMR** (400 MHz, Chloroform-*d*) δ 7.86 (br s, 1H, N*H*), 7.74 (d, *J* = 1.8 Hz, 1H, Ar-*H*), 7.66 – 7.57 (m, 1H, Ar-*H*), 7.16 – 7.12 (m, 1H, Ar-*H*), 7.10 – 7.00 (m, 3H, Ar-*H* x 3), 6.93 (d, *J* = 8.5 Hz, 1H, Ar-*H*), 5.88 – 5.69 (m, 2H, $CH_2CH=CH_2$ x 2), 5.15 – 5.08 (m, 2H, $CH_2CH=CH_2$), 4.99 (dq, *J* = 10.2, 1.7 Hz, 1H, $CH_2CH=CH_2$), 4.82 (dq, *J* = 17.2, 1.7 Hz, 1H, $CH_2CH=CH_2$), 3.84 (dt, *J* = 5.7, 1.7 Hz, 2H, $CH_2CH=CH_2$), 3.74 (dt, *J* = 6.6, 1.4 Hz, 2H, $CH_2CH=CH_2$), 3.53 (s, 3H, NCH_3). **^{13}C NMR** (101 MHz, Chloroform-*d*) δ 141.0 (C_q), 140.6 (C_q), 137.1 (C_q), 134.3 ($CH_2CH=CH_2$), 134.2 ($CH_2CH=CH_2$), 133.9 (C_q), 132.1 (C_q), 129.5 (C_q), 124.8 (*C*H), 122.2 (*C*H), 121.8 (*C*H), 120.1 (*C*H), 119.3 (*C*H), 118.4 ($CH_2CH=CH_2$), 116.9 ($CH_2CH=CH_2$), 113.7 (C_q), 112.1 (*C*H), 109.2 (*C*H), 104.6 (C_q), 103.5 (C_q), 31.4 ($CH_2CH=CH_2$), 30.5 (NCH_3), 29.7 ($CH_2CH=CH_2$). **ν_{max}** (neat)/ cm^{-1} 3400, 3077, 2926, 1637, 1524, 1465, 1441, 1407, 1302, 915, 795, 746. **HRMS** (ESI) calculated for $C_{23}H_{21}N_2SBr$ (M^+): 436.0609, found 436.0603.

^{1}H and ^{13}C NMR spectra of compounds

3a

^{1}H NMR (400 MHz, Chloroform-*d*)

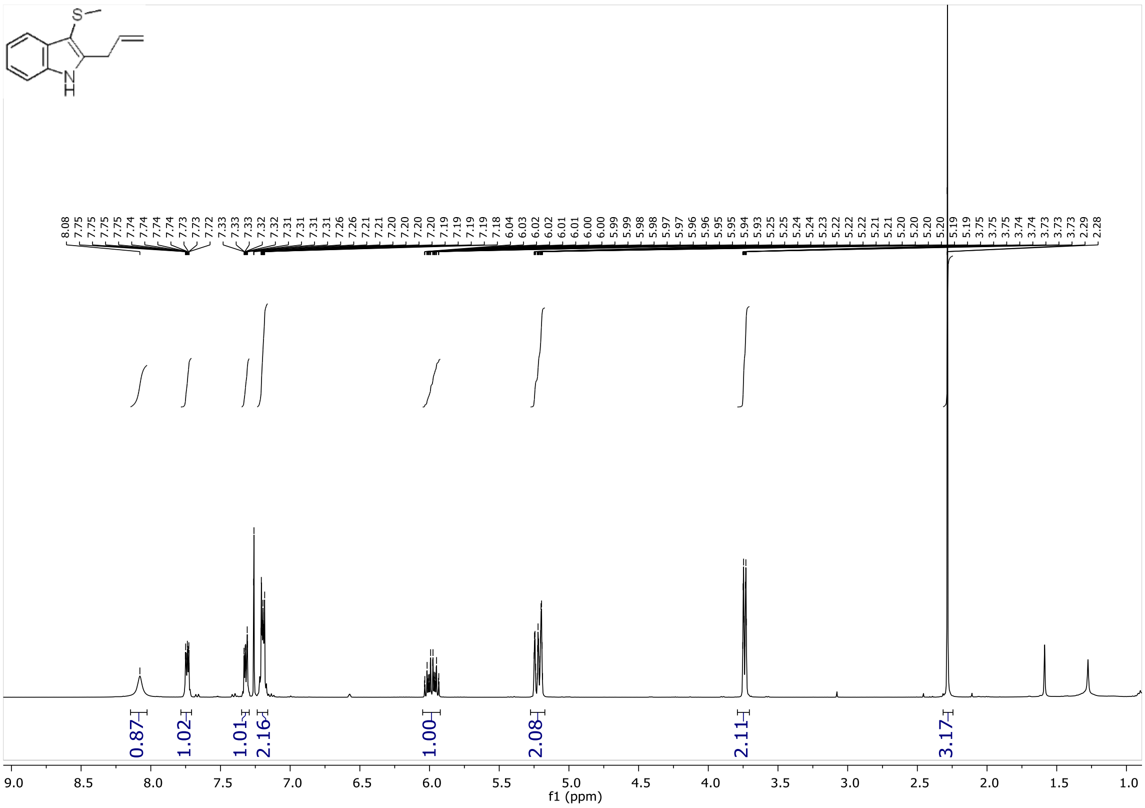

^{13}C NMR (101 MHz, Chloroform-*d*)

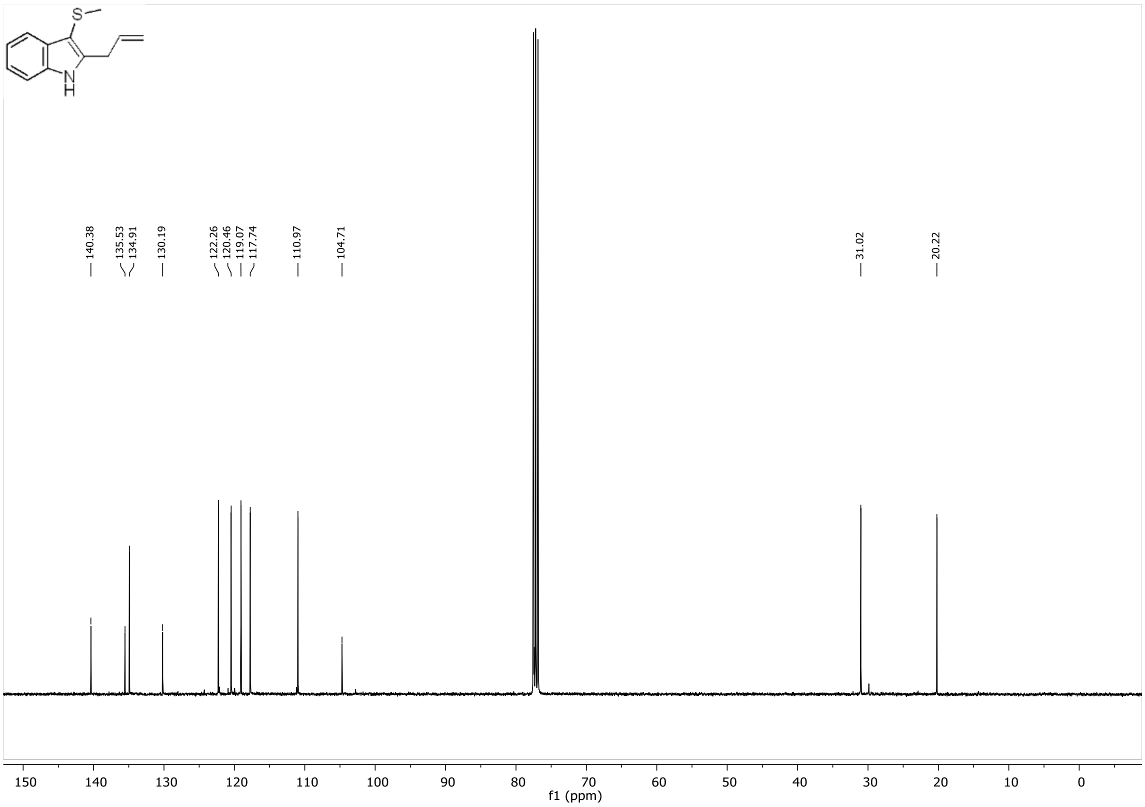

3b

^{1}H NMR (400 MHz, Chloroform-*d*)

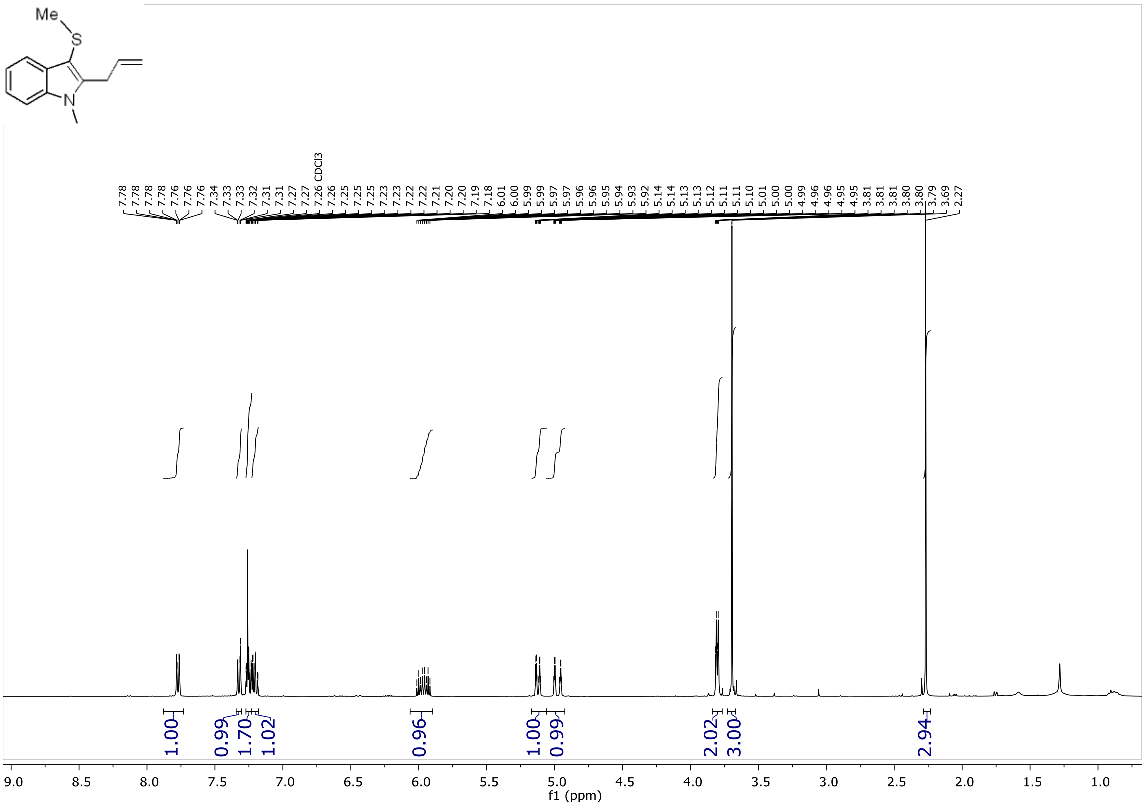

^{13}C NMR (101 MHz, Chloroform-*d*)

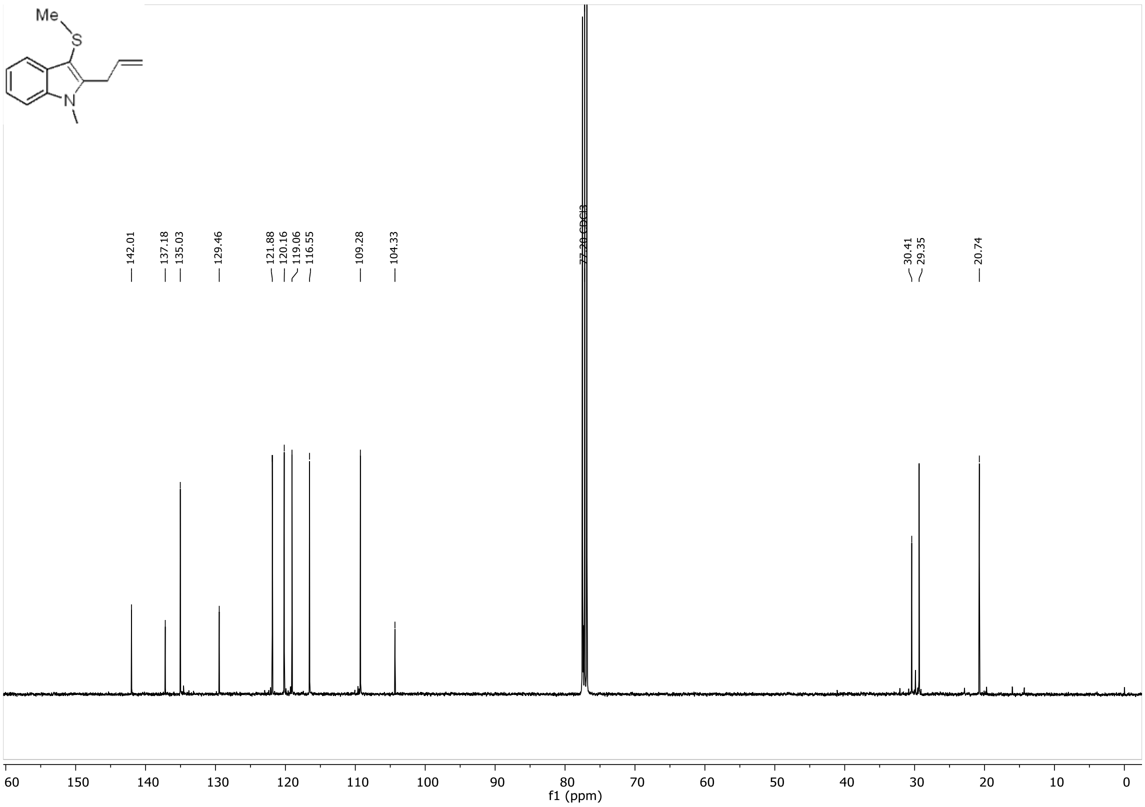

3c

^{1}H NMR (400 MHz, Chloroform-*d*)

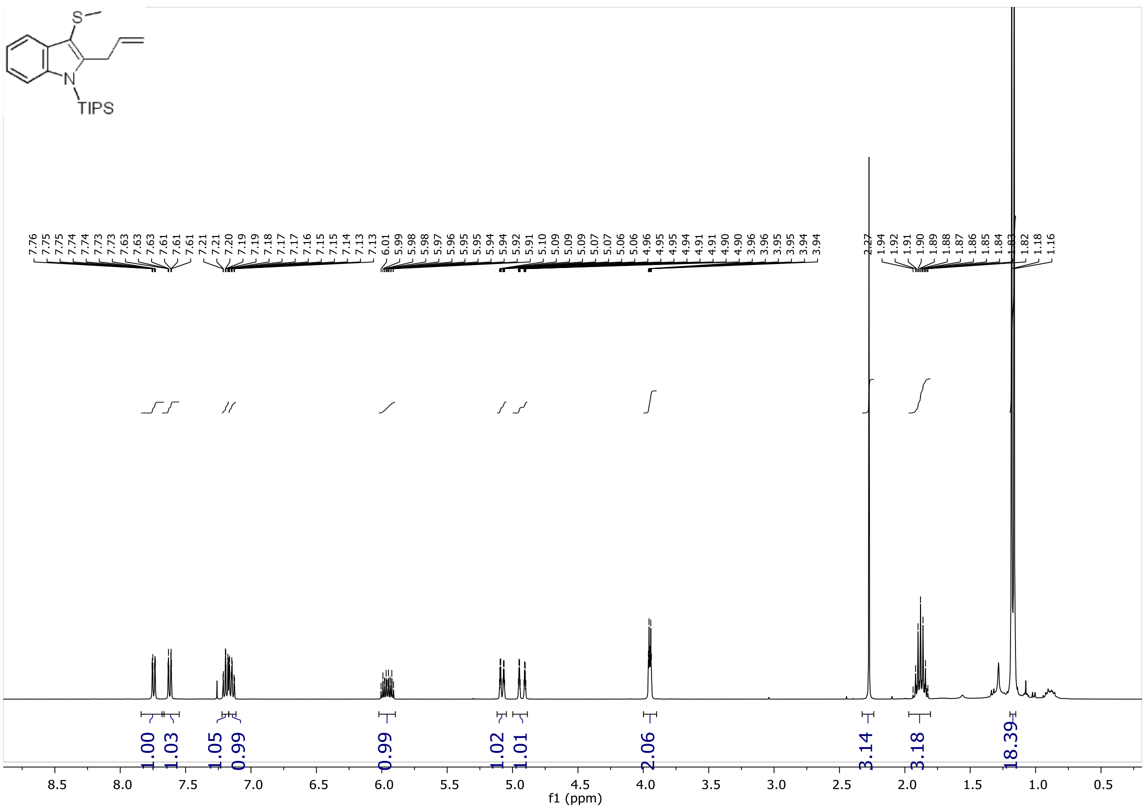

^{13}C NMR (101 MHz, Chloroform-*d*)

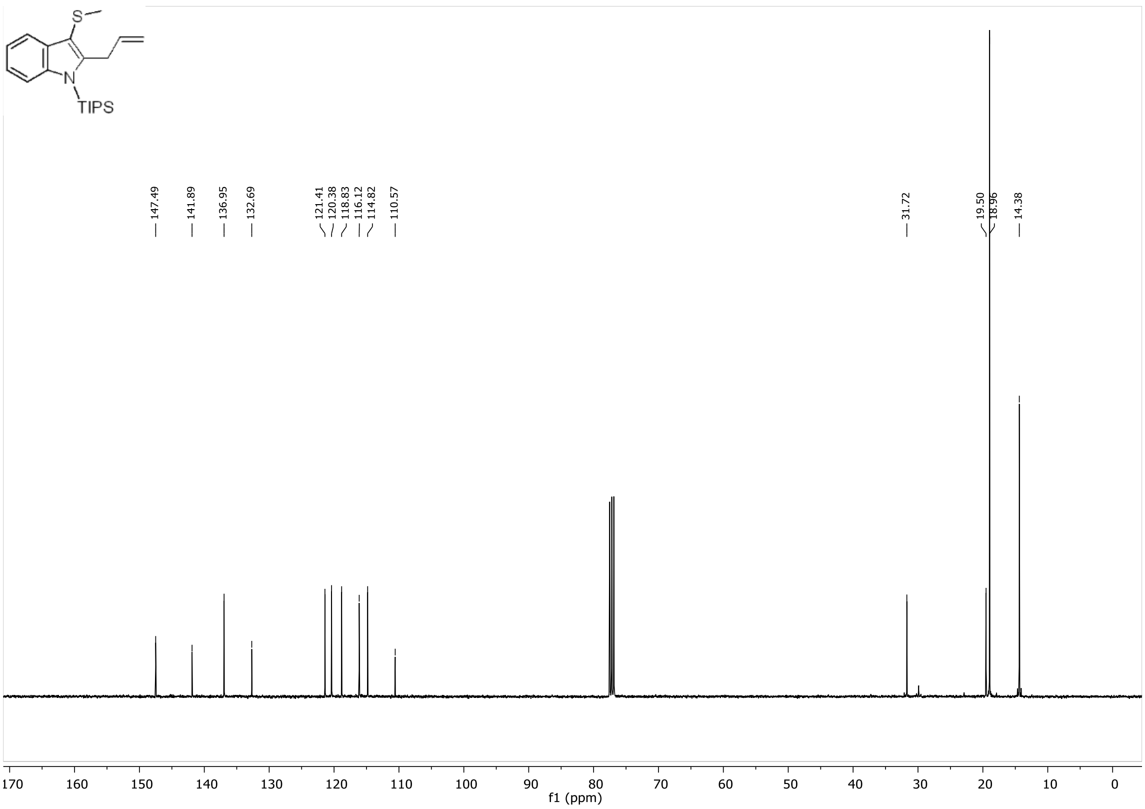

3d

^{1}H NMR (500 MHz, Chloroform-*d*)

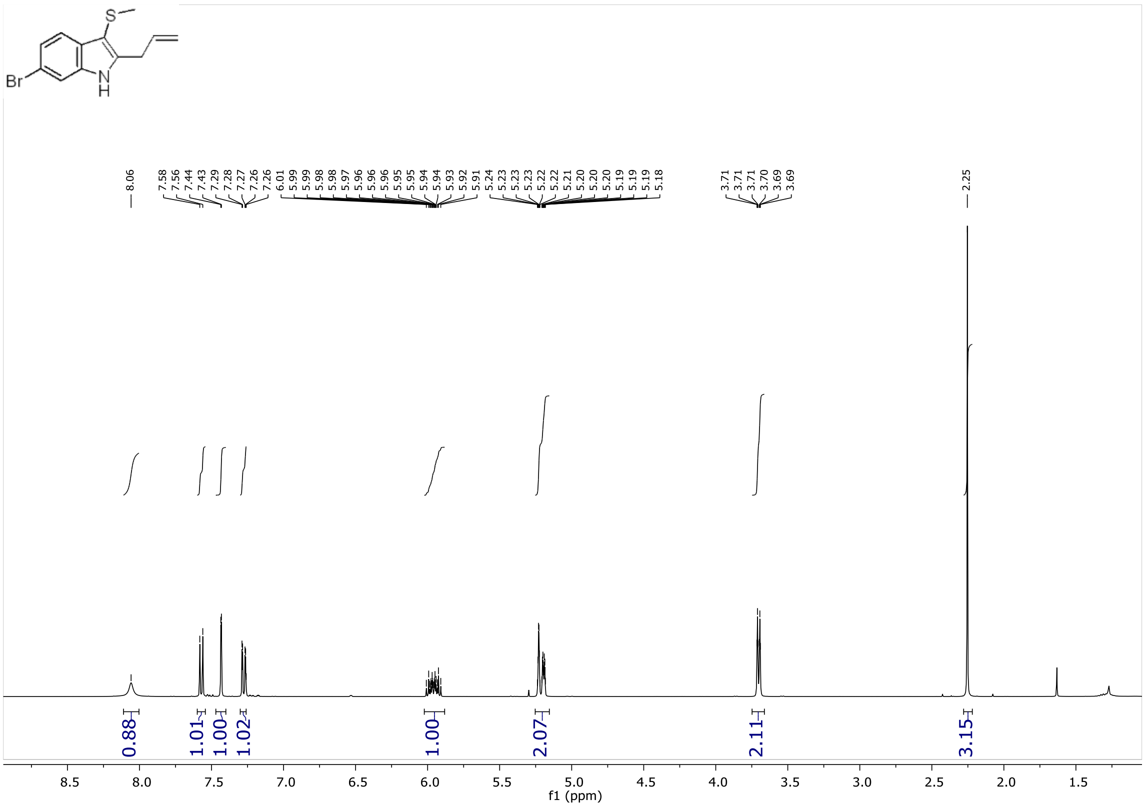

^{13}C NMR (101 MHz, Chloroform-*d*)

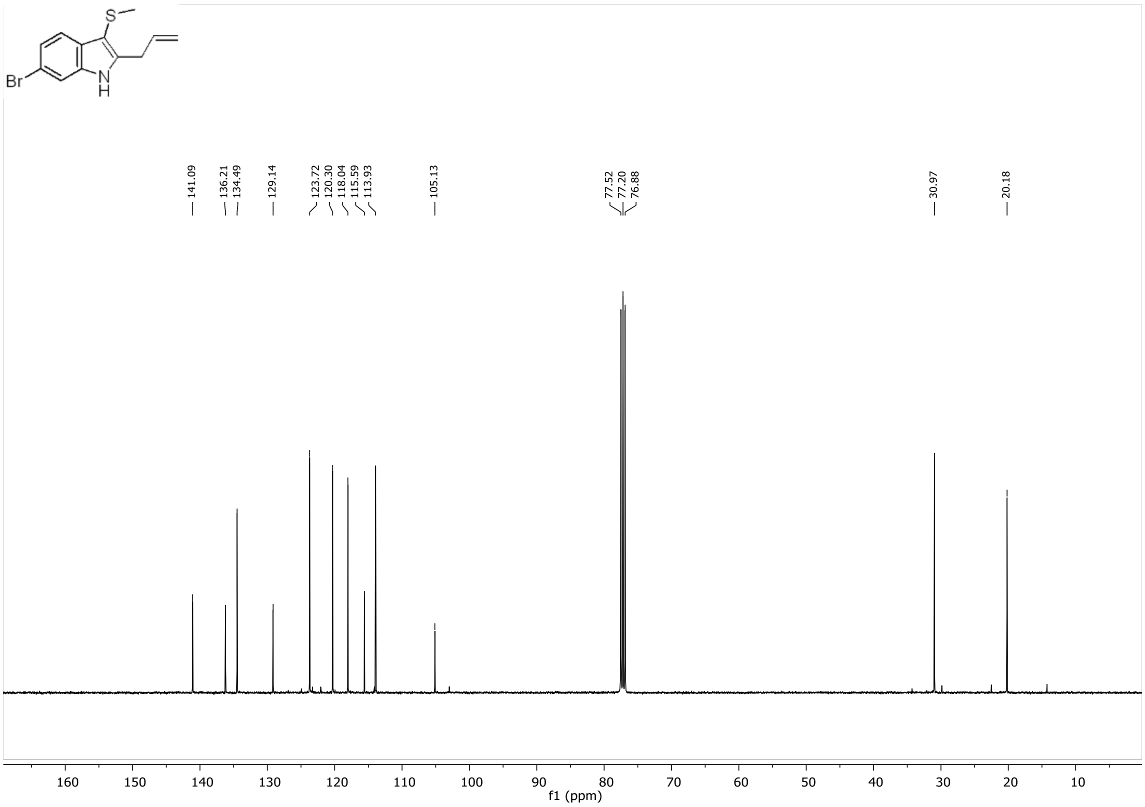

3e

^{1}H NMR (500 MHz, Chloroform-*d*)

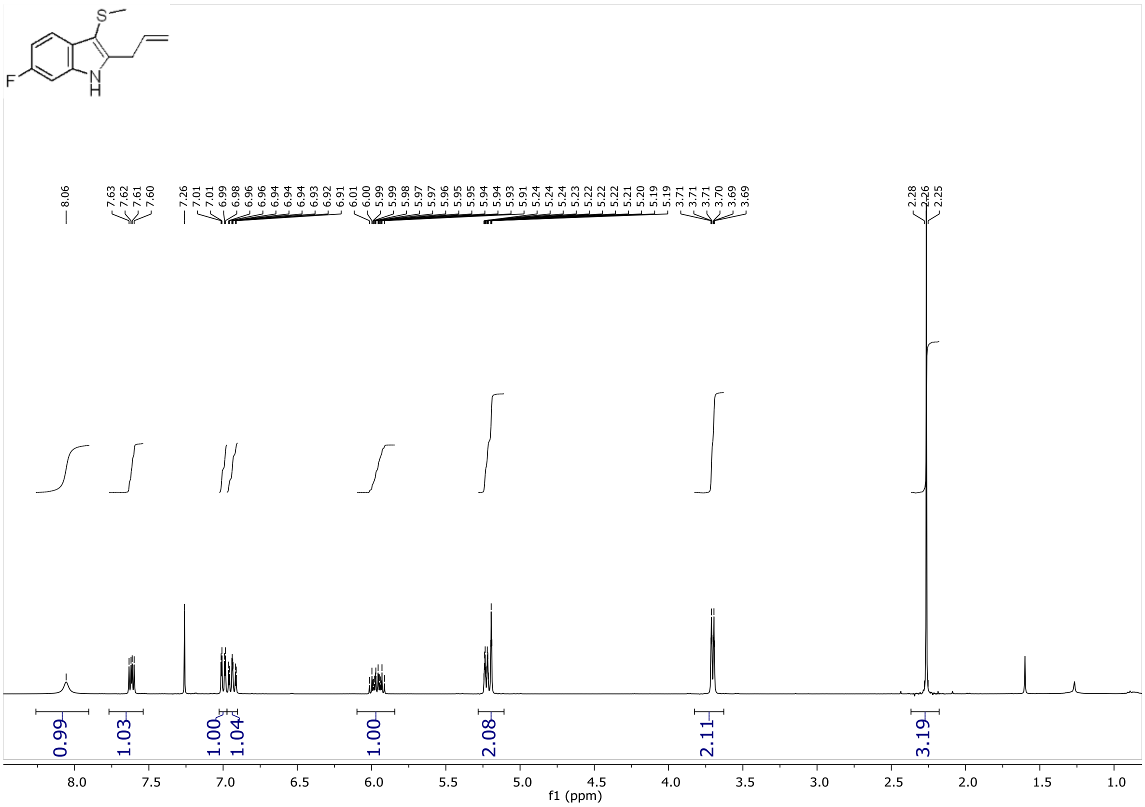

^{13}C NMR (101 MHz, Chloroform-*d*)

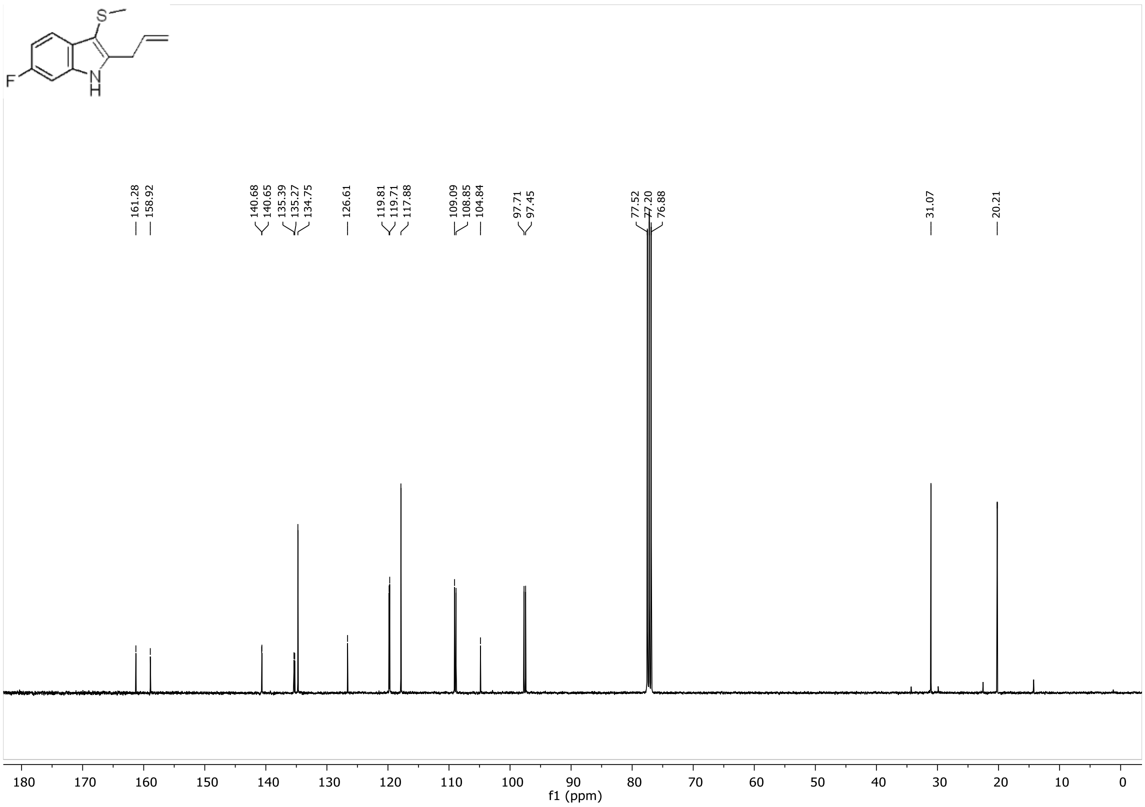

^{19}F NMR (376 MHz, Chloroform-d)

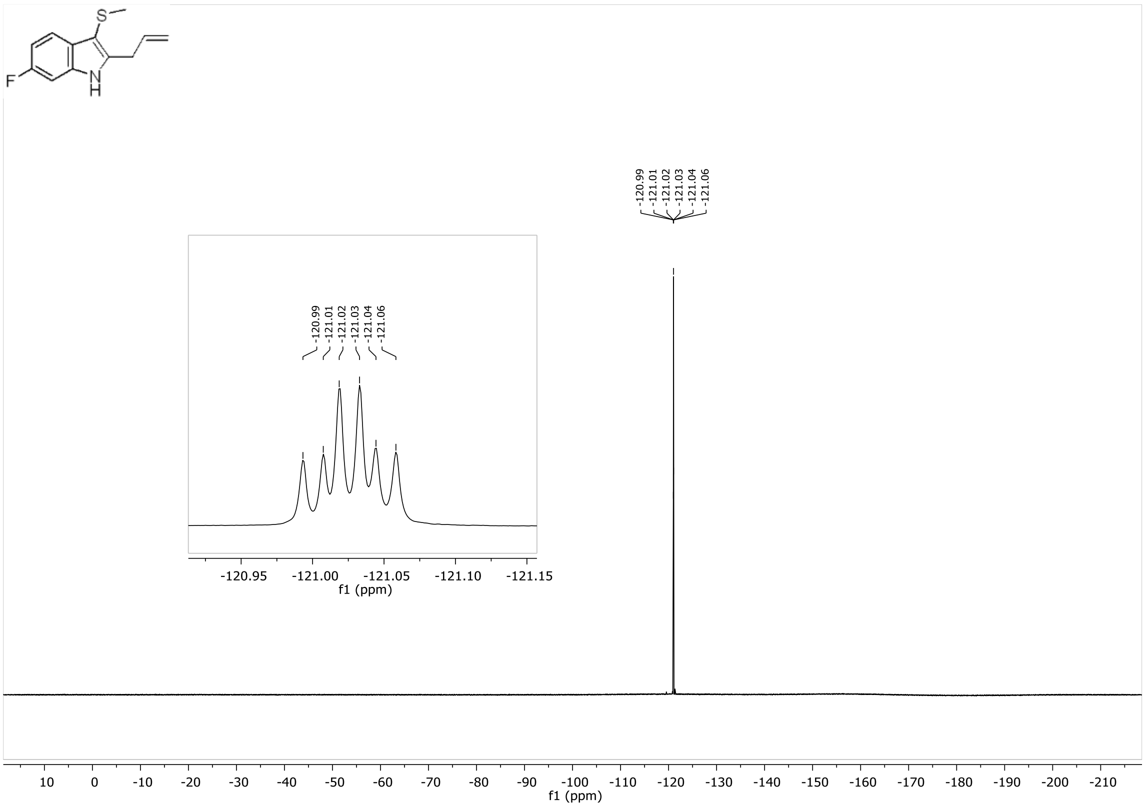

3f

^{1}H NMR (400 MHz, Chloroform-*d*)

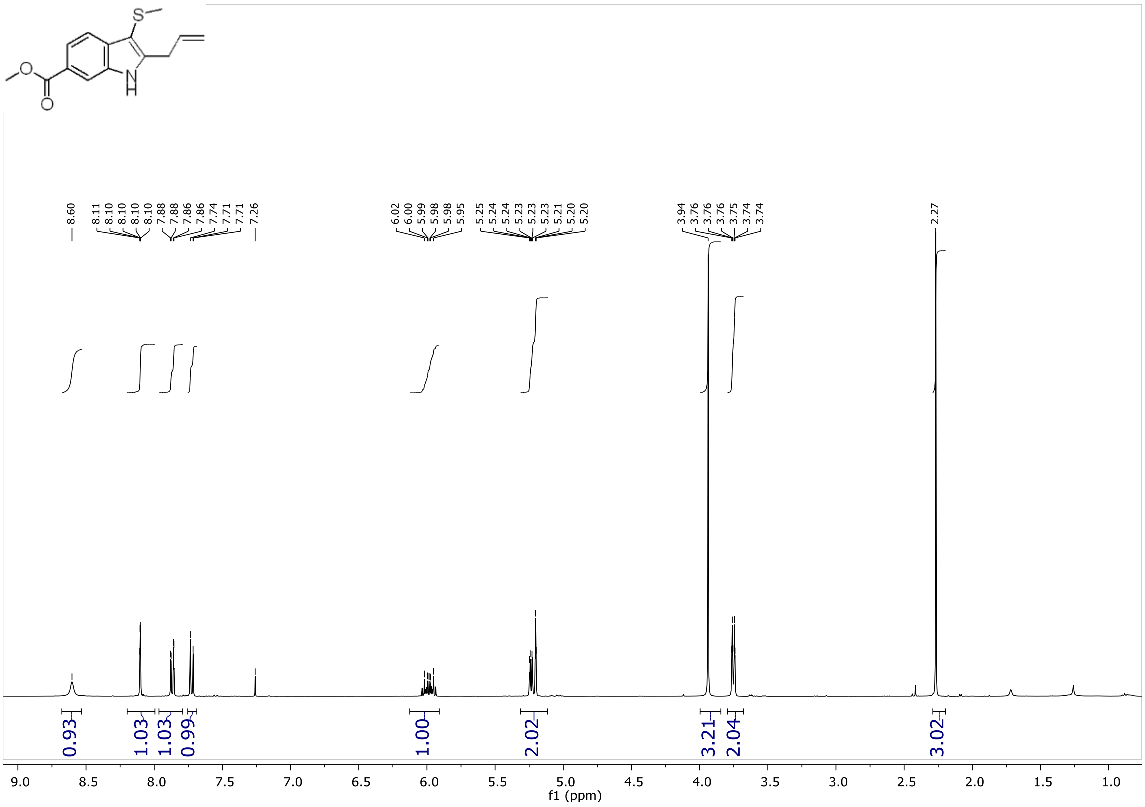

^{13}C NMR (101 MHz, Chloroform-*d*)

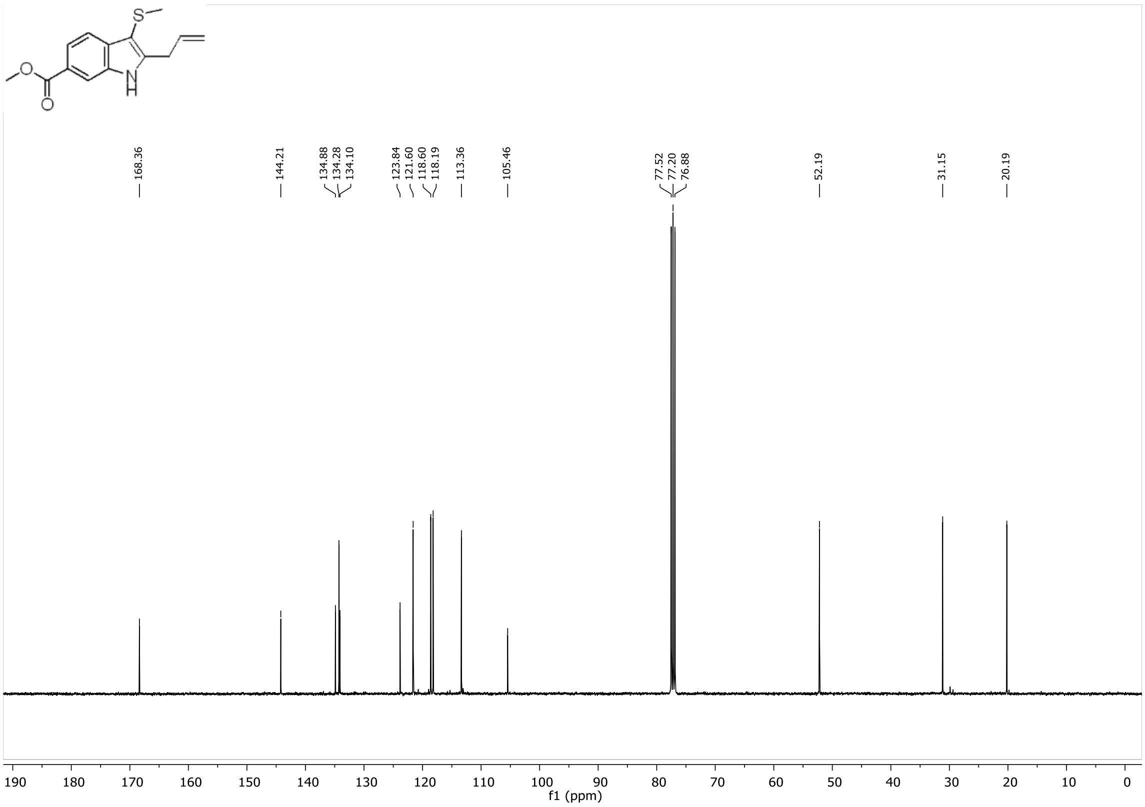

3g

^{1}H NMR (500 MHz, Chloroform-*d*)

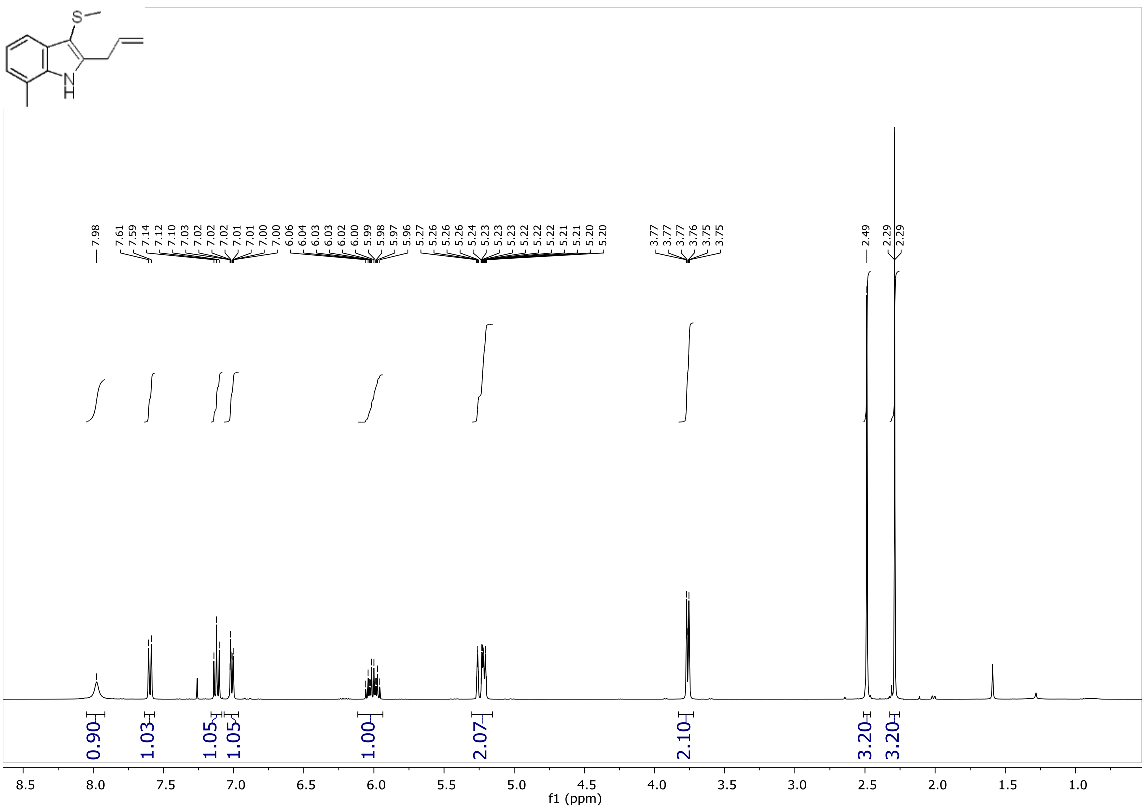

^{13}C NMR (101 MHz, Chloroform-*d*)

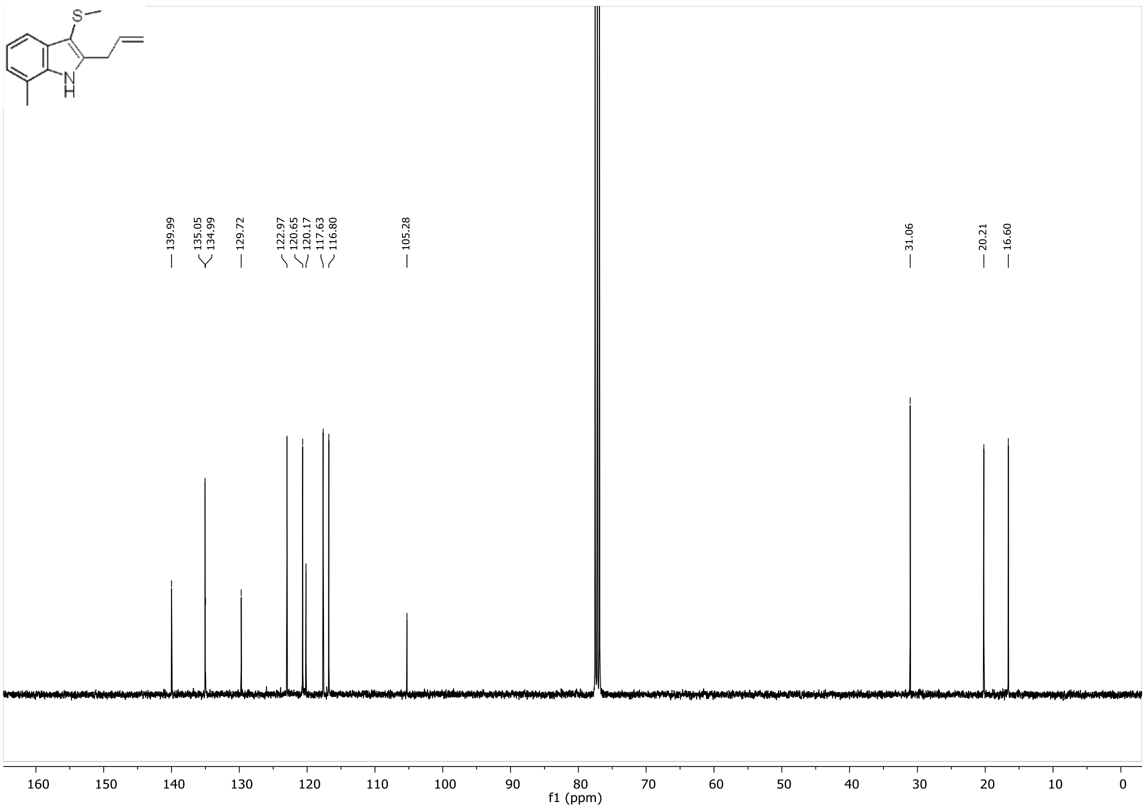

3h

^{1}H NMR (400 MHz, Chloroform-*d*)

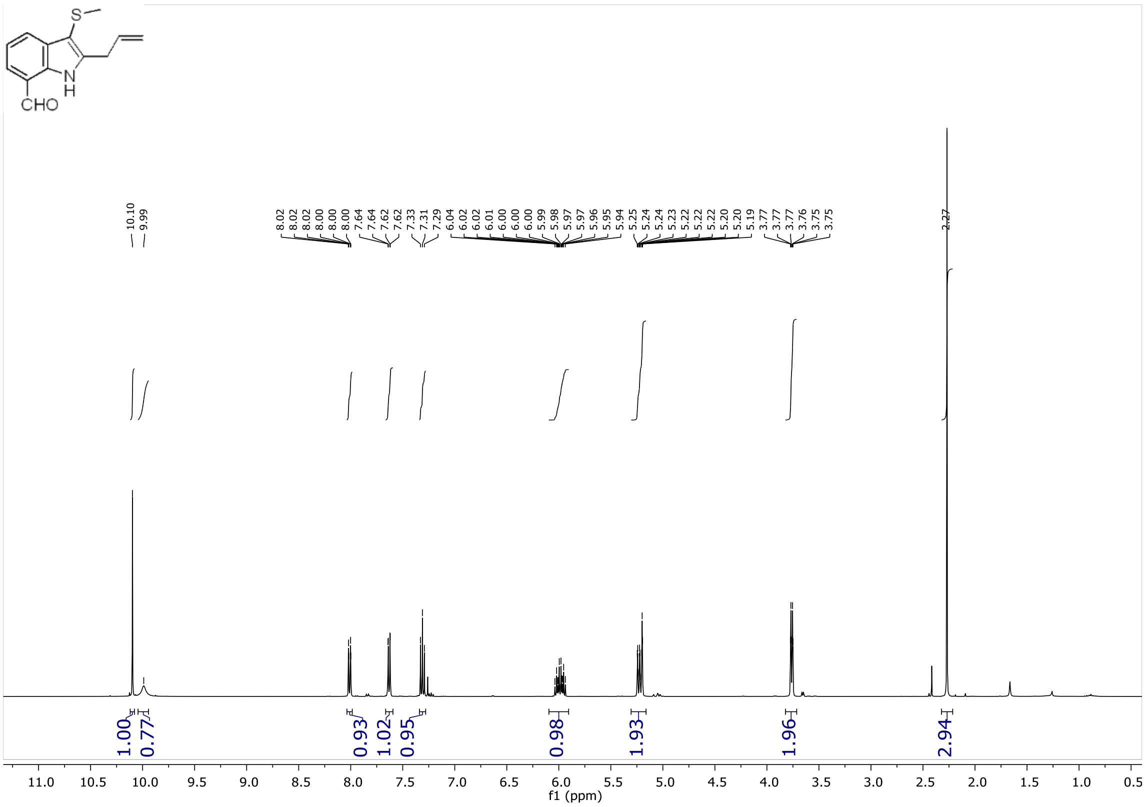

^{13}C NMR (101 MHz, Chloroform-*d*)

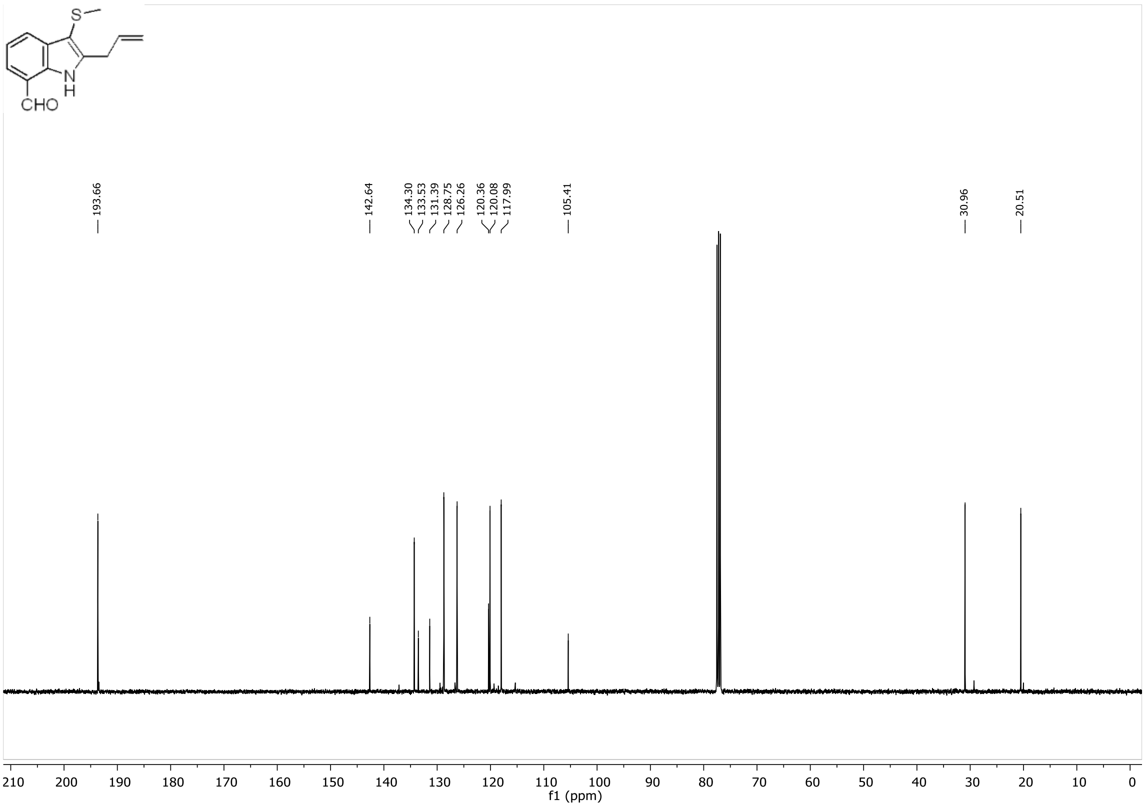

3i

^{1}H NMR (400 MHz, Chloroform-*d*)

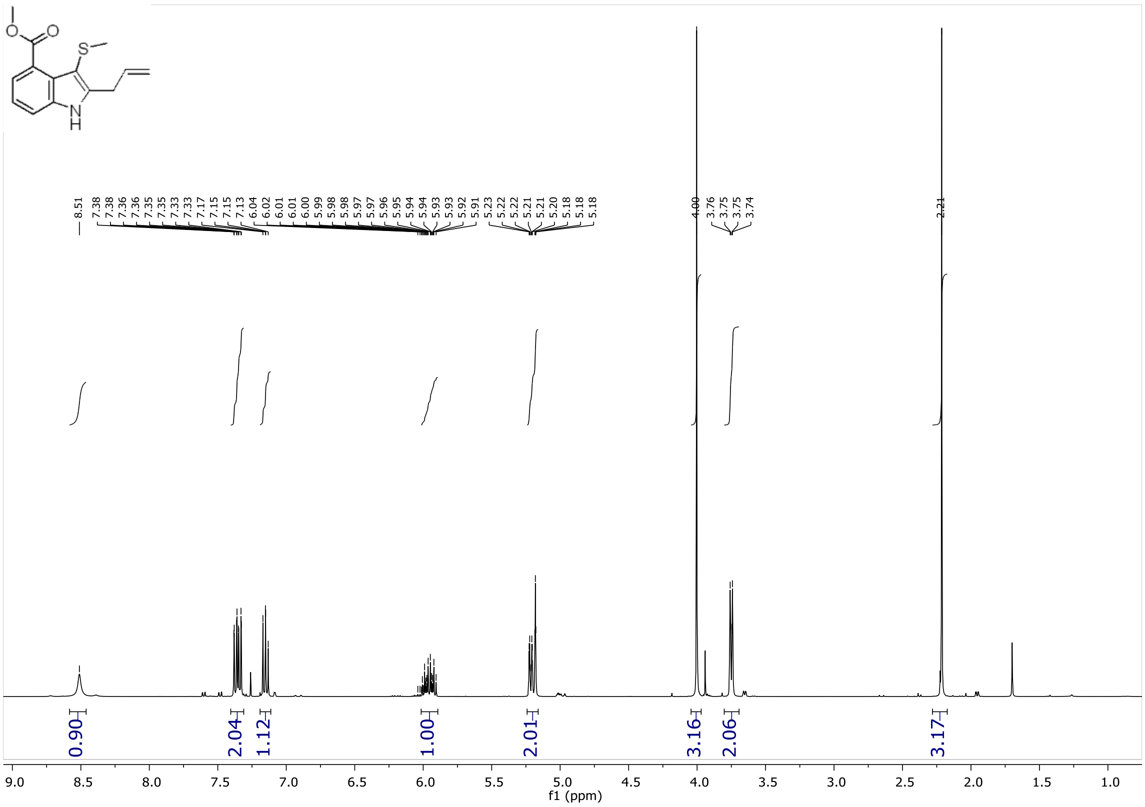

^{13}C NMR (101 MHz, Chloroform-*d*)

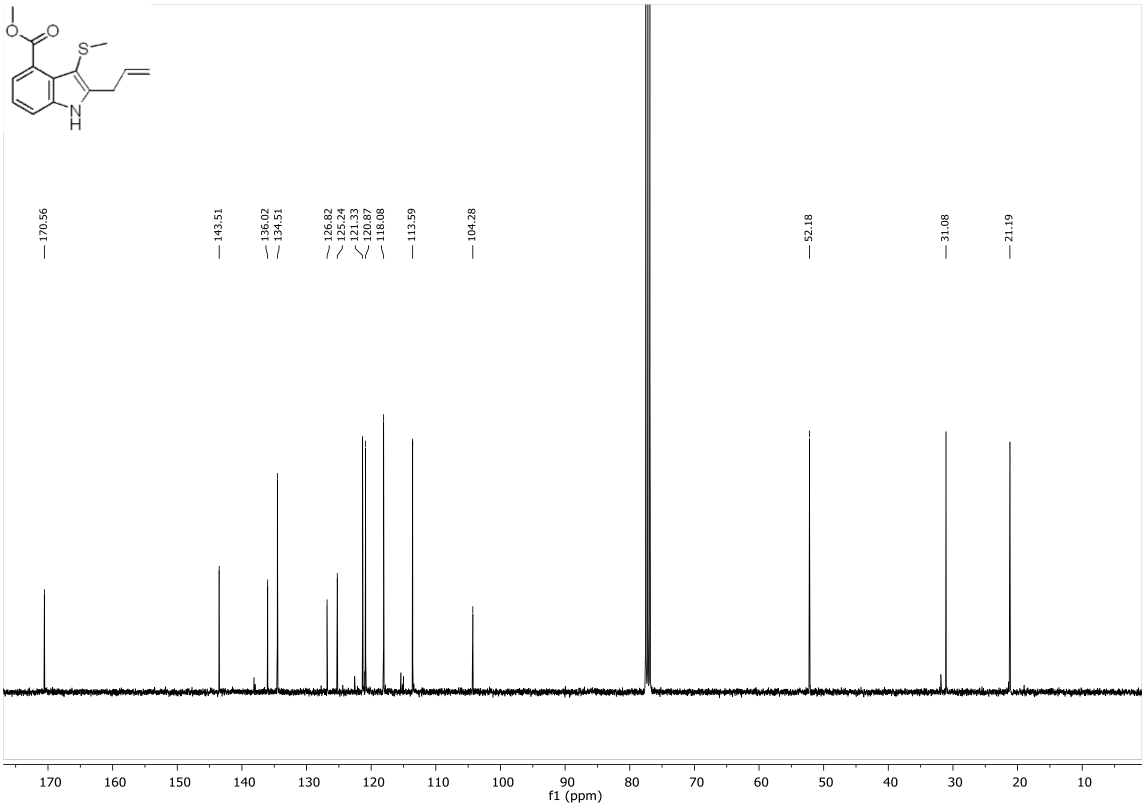

3j

^{1}H NMR (500 MHz, Chloroform-*d*)

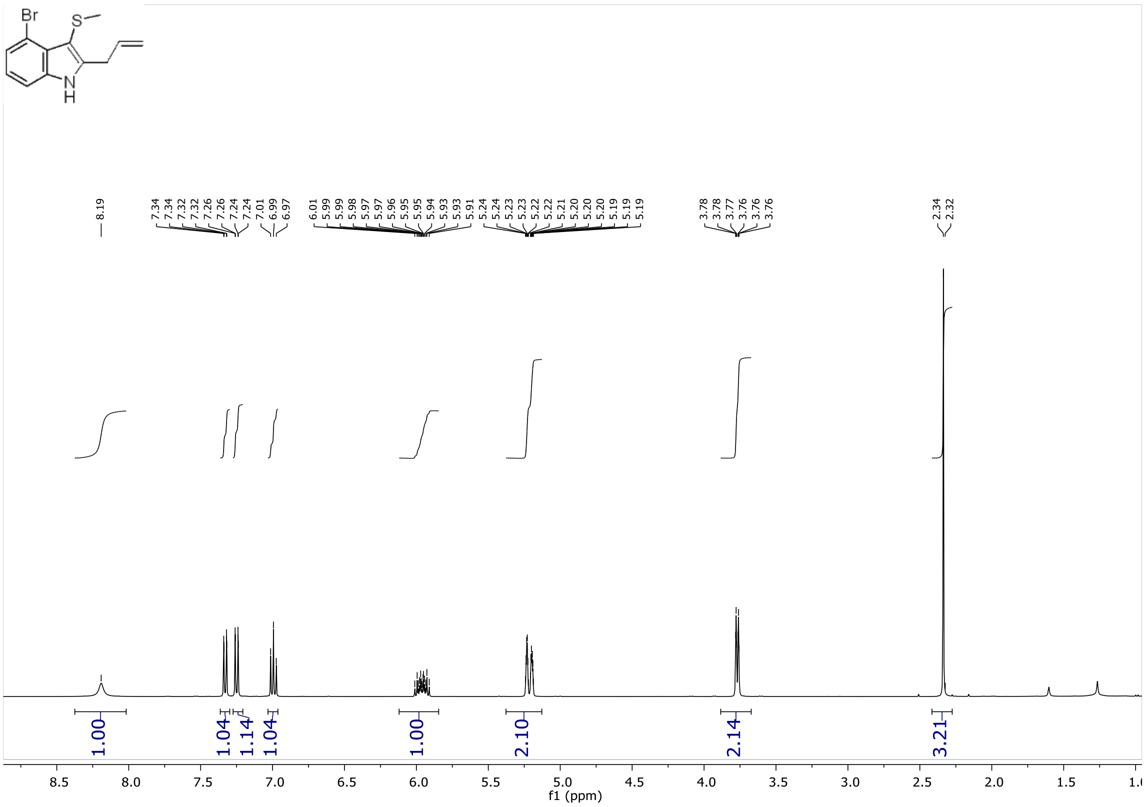

^{13}C NMR (101 MHz, Chloroform-*d*)

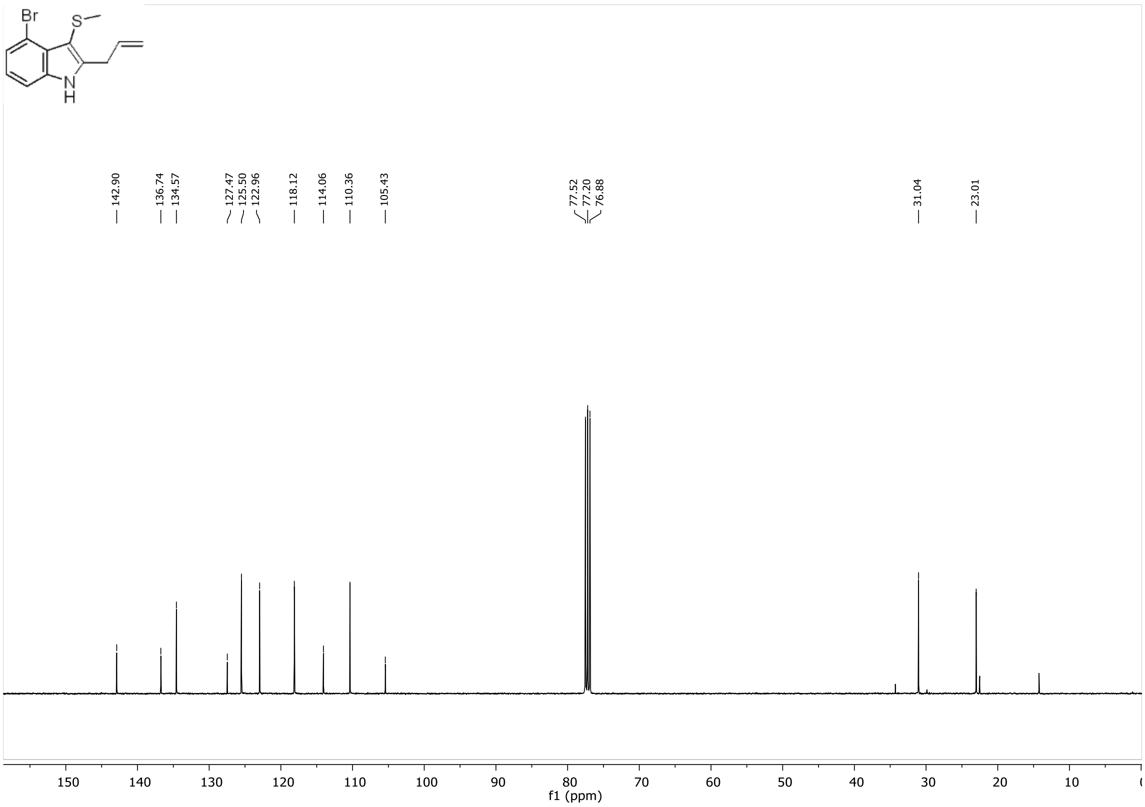

3k

^{1}H NMR (400 MHz, Chloroform-*d*)

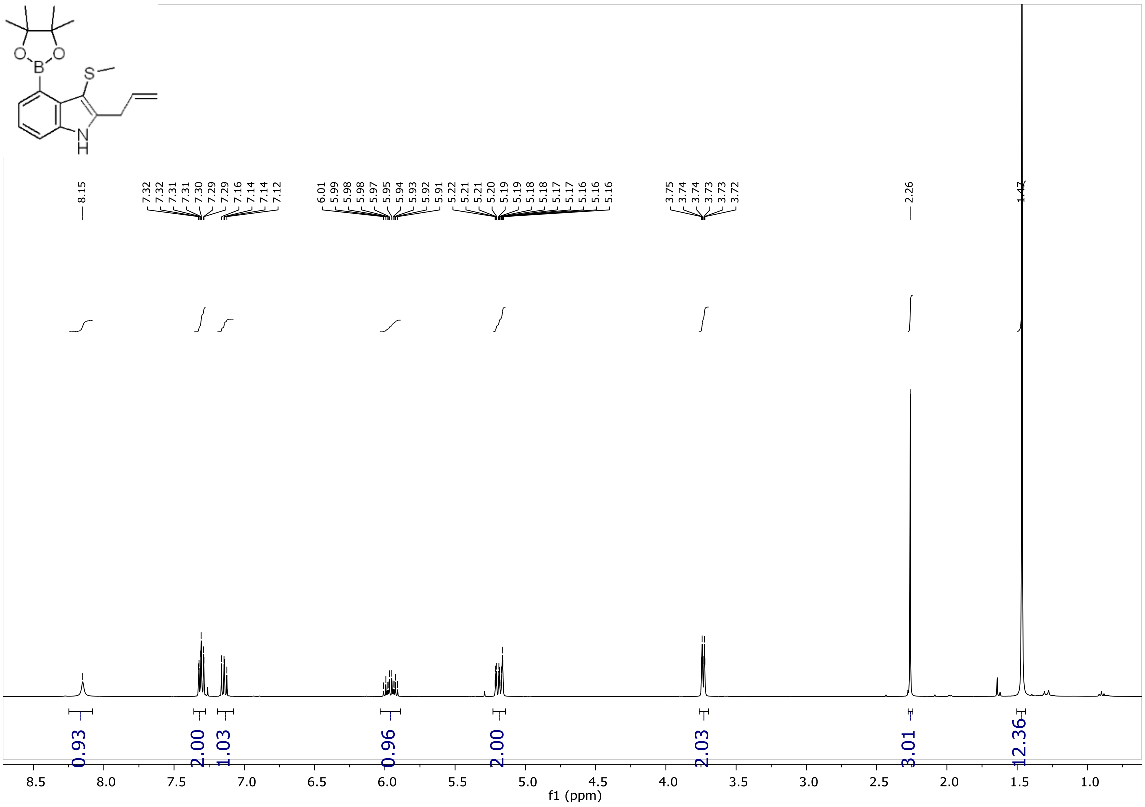

^{13}C NMR (101 MHz, Chloroform-*d*)

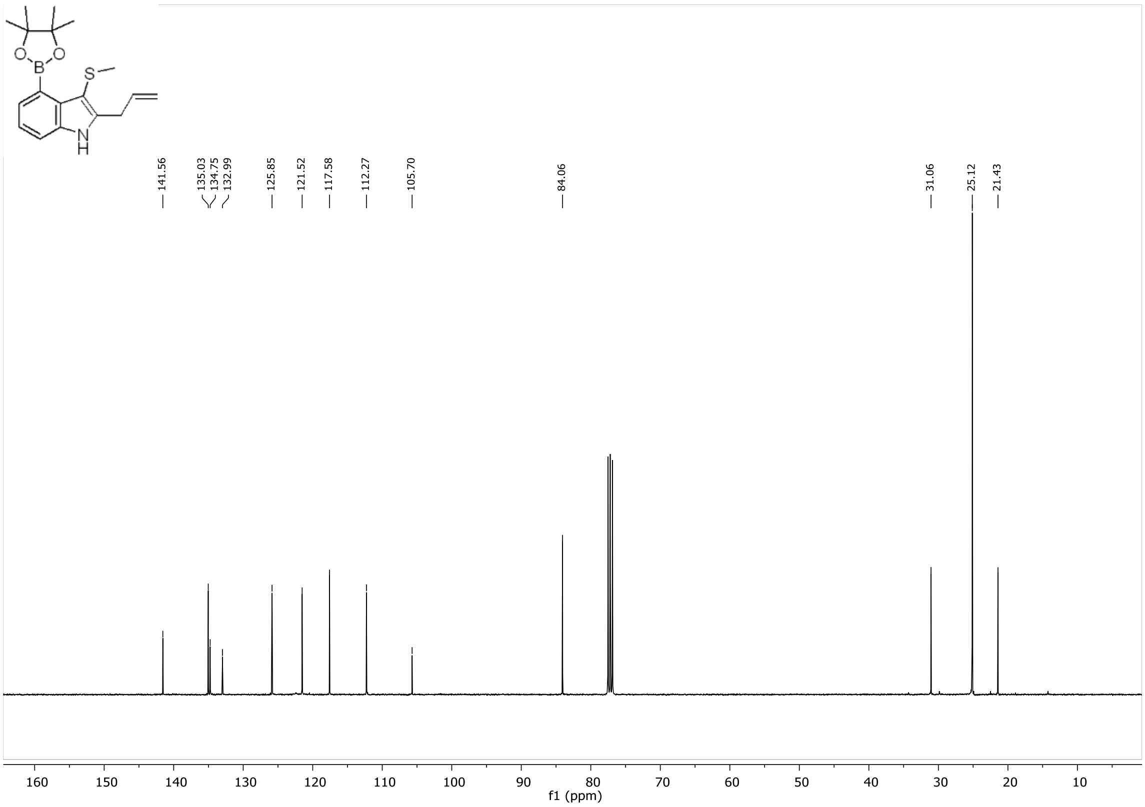

3l

^{1}H NMR (400 MHz, Chloroform-*d*)

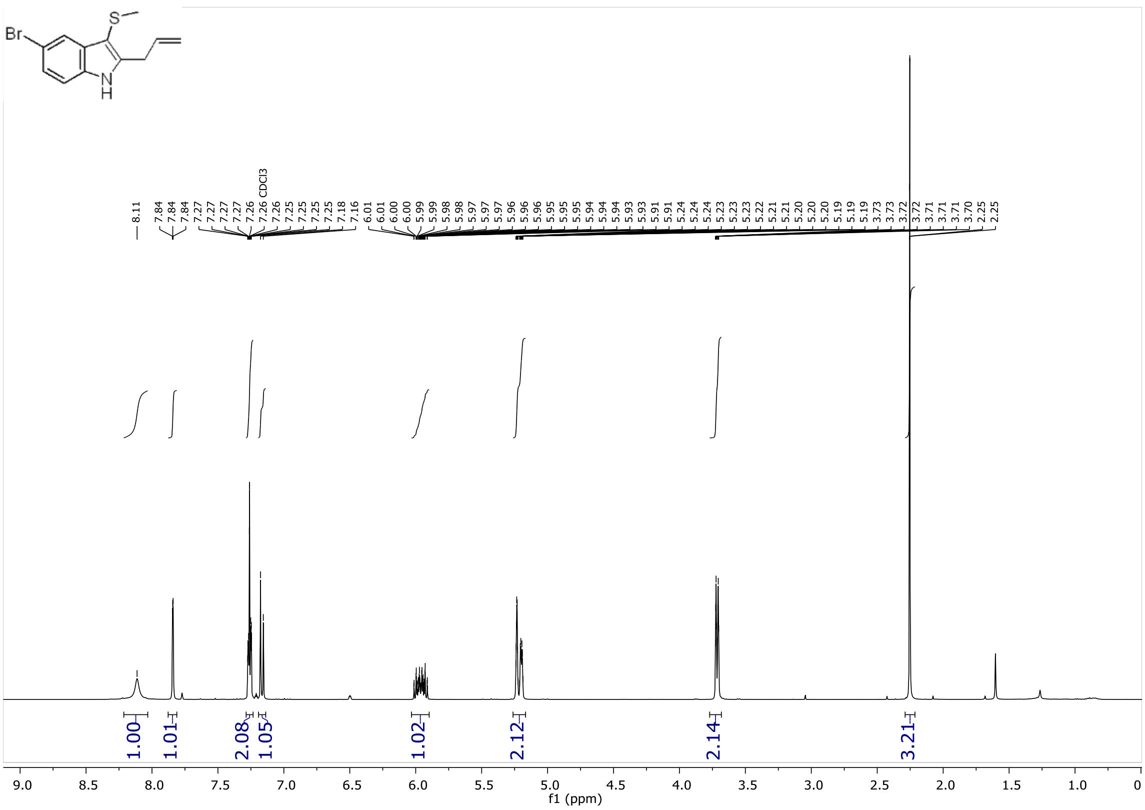

^{13}C NMR (101 MHz, Chloroform-*d*)

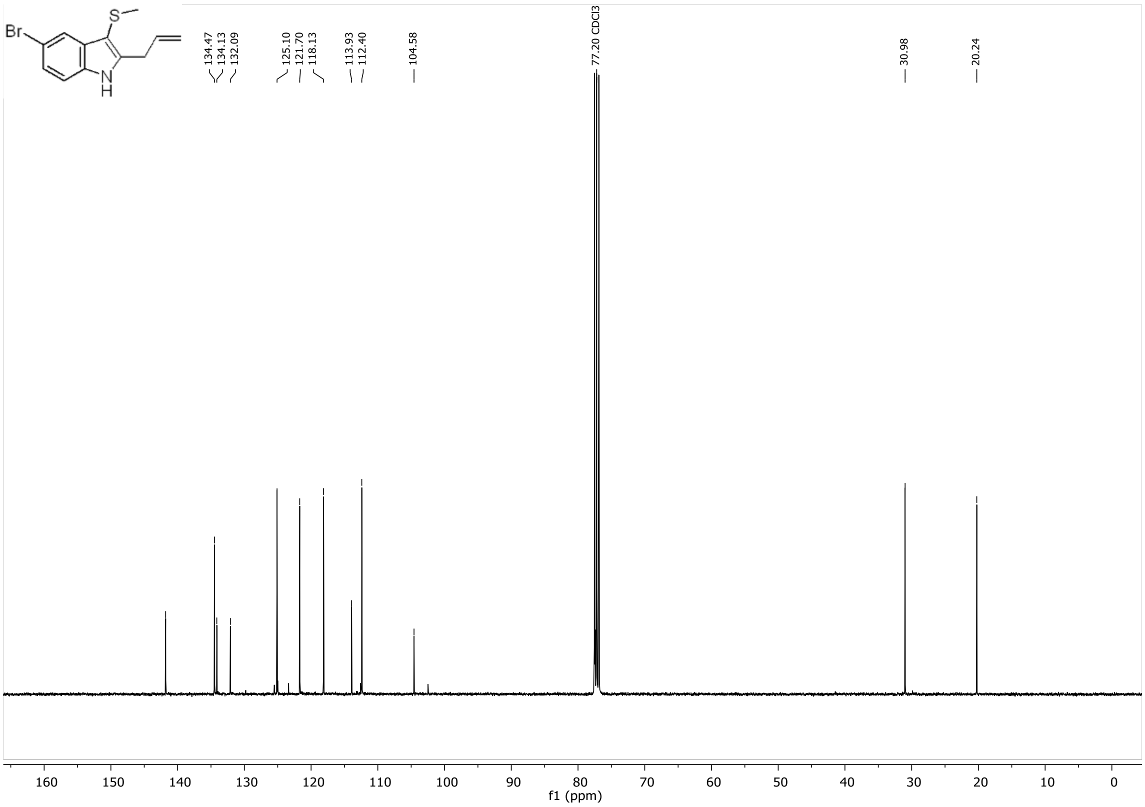

3m

¹H NMR (500 MHz, Chloroform-*d*)

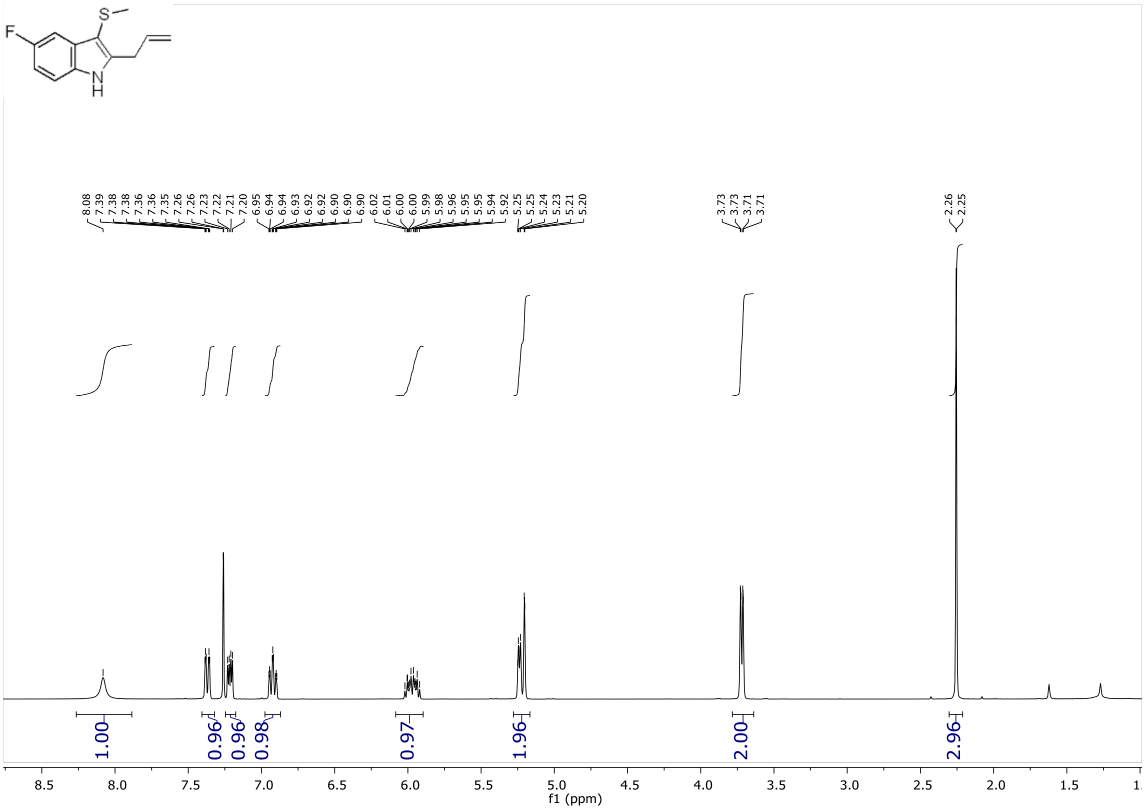

¹³C NMR (101 MHz, Chloroform-*d*)

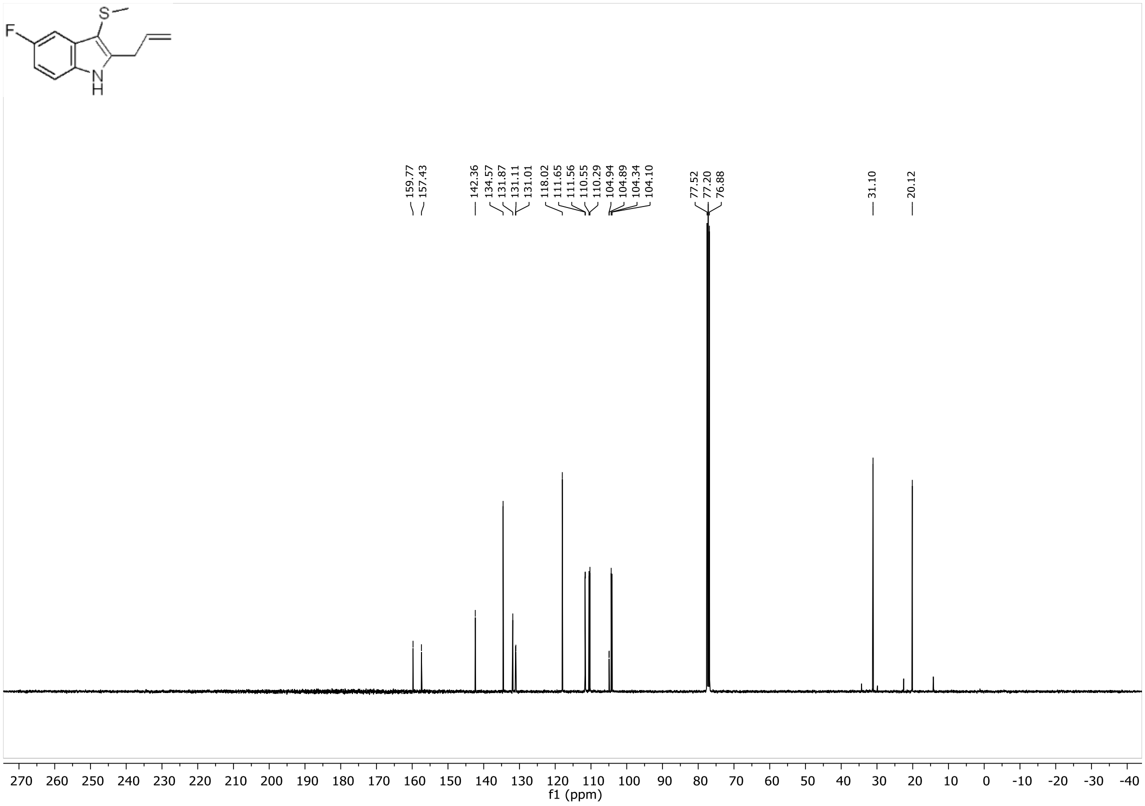

^{19}F NMR (376 MHz, Chloroform-d)

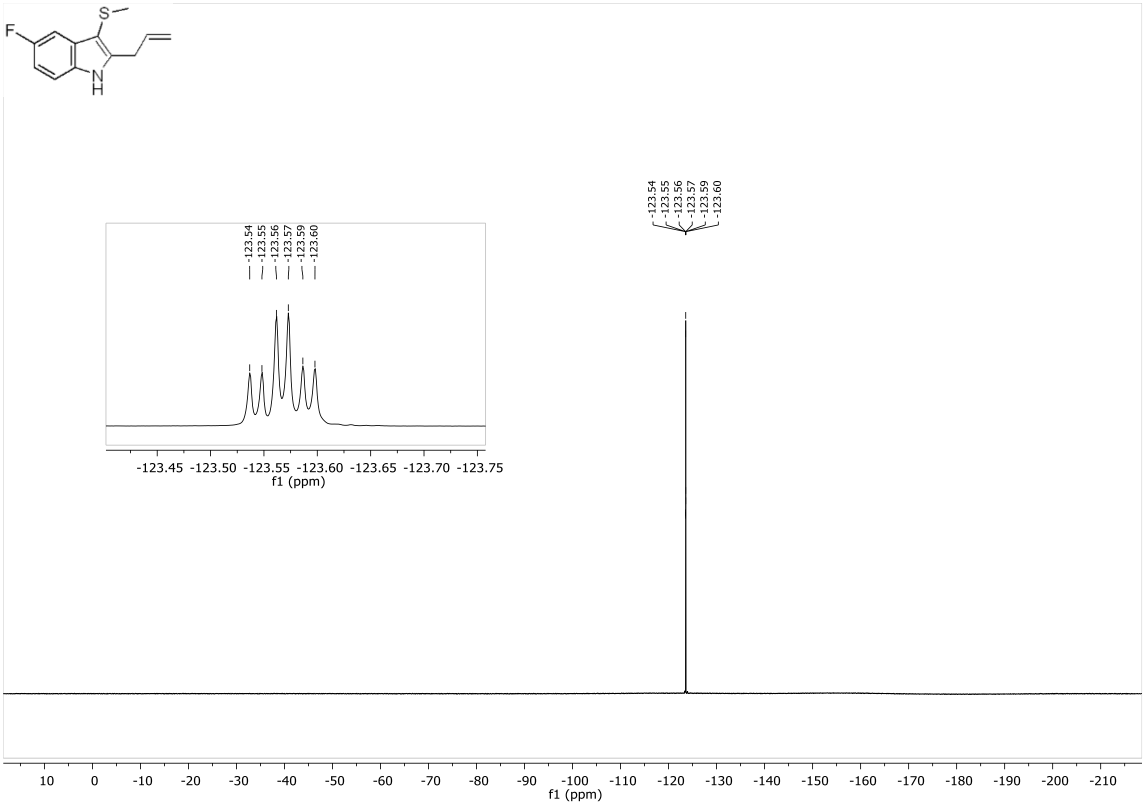

3n

^{1}H NMR (400 MHz, Chloroform-*d*)

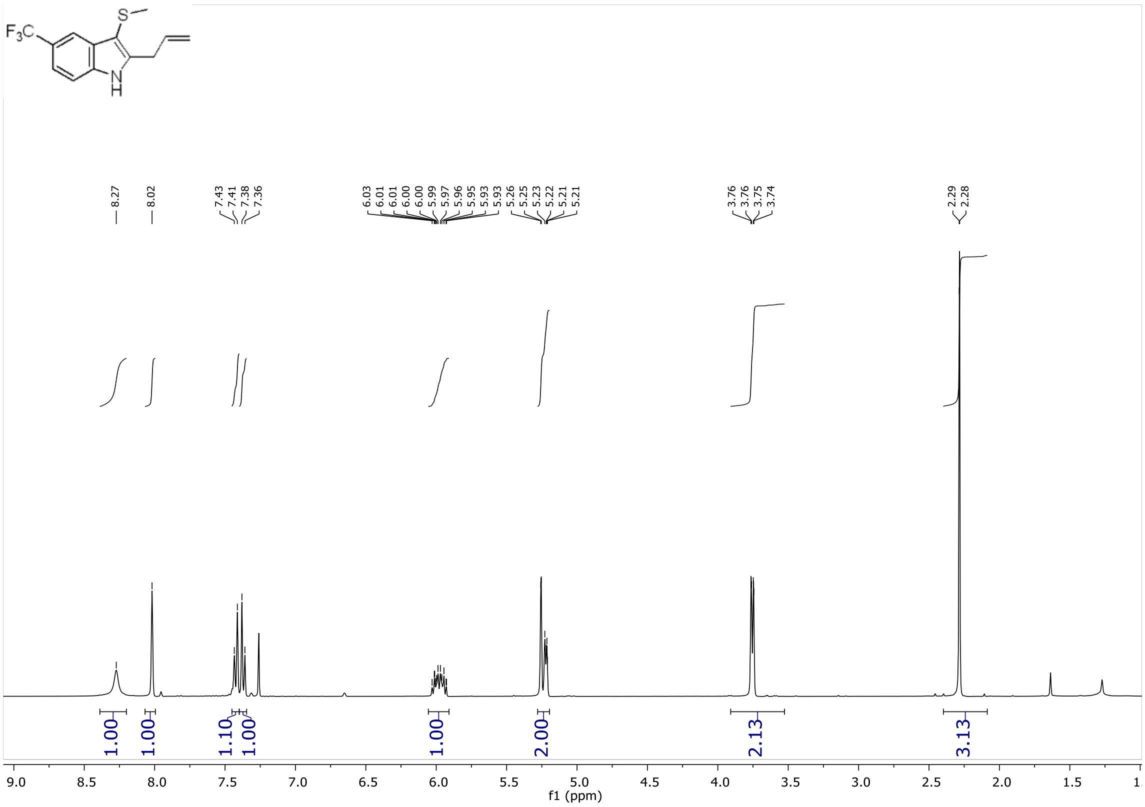

^{13}C NMR (101 MHz, Chloroform-*d*)

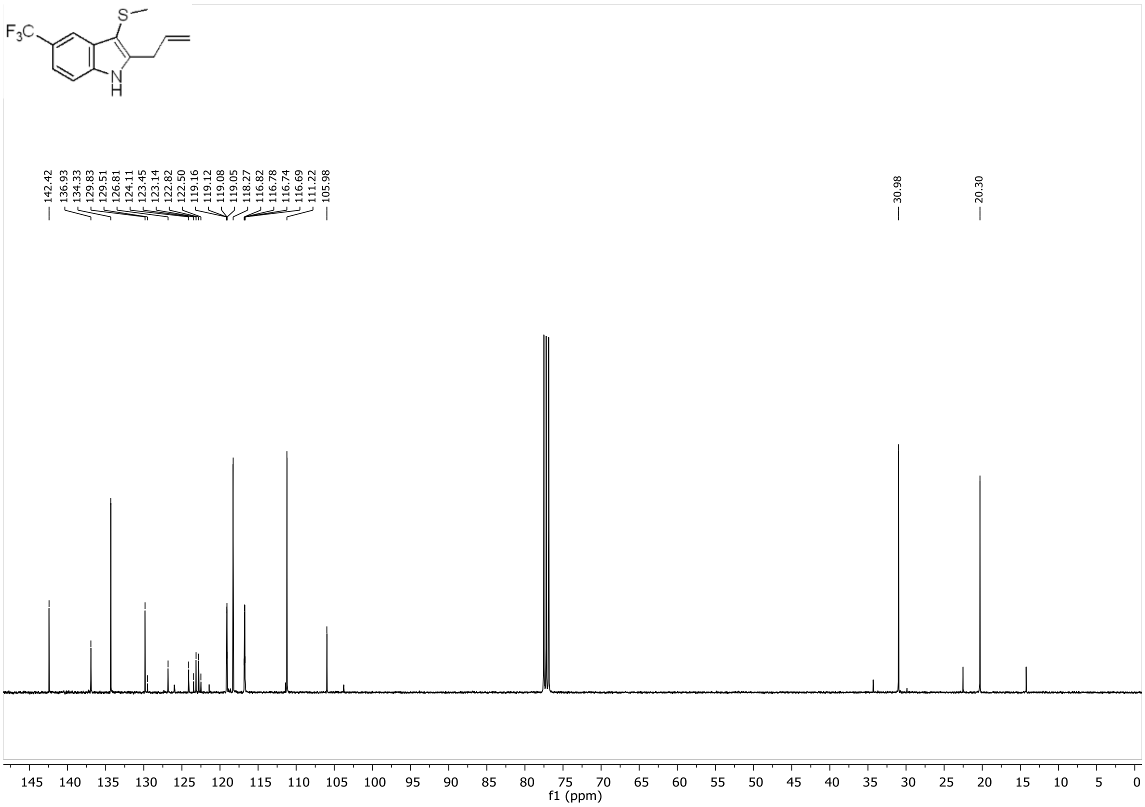

^{19}F NMR (376 MHz, Chloroform-d)

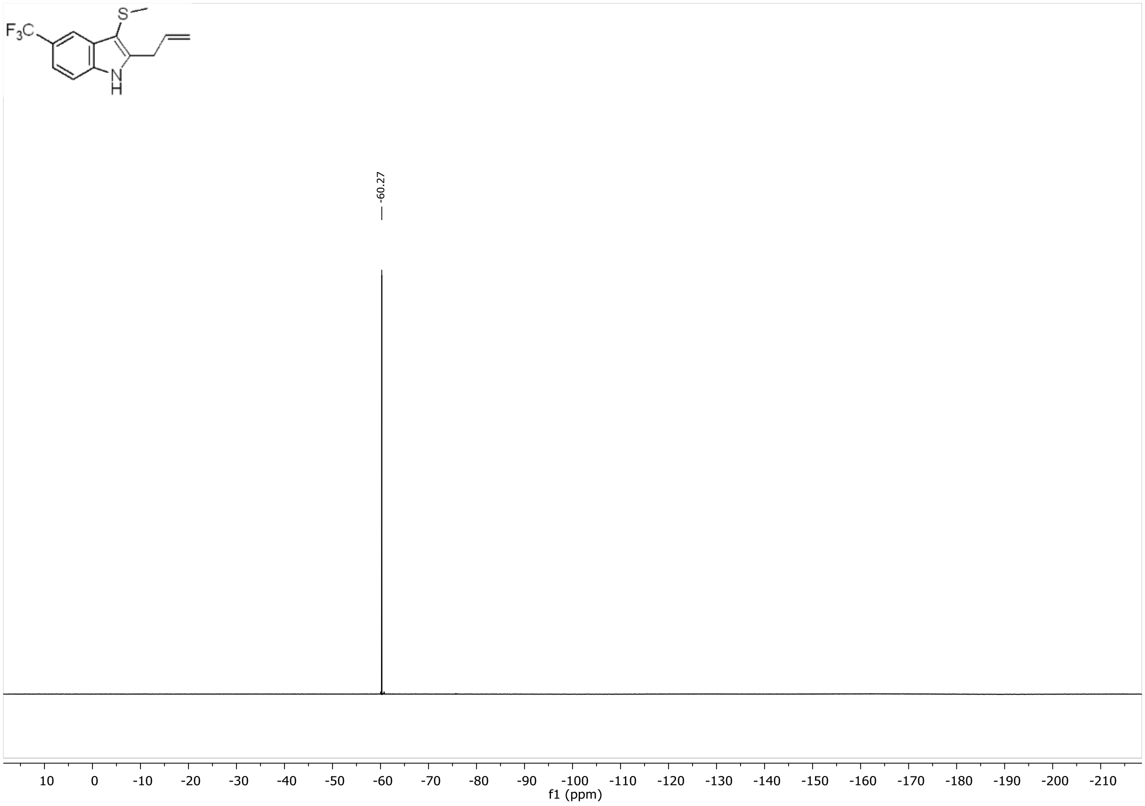

3o

^{1}H NMR (400 MHz, Chloroform-*d*)

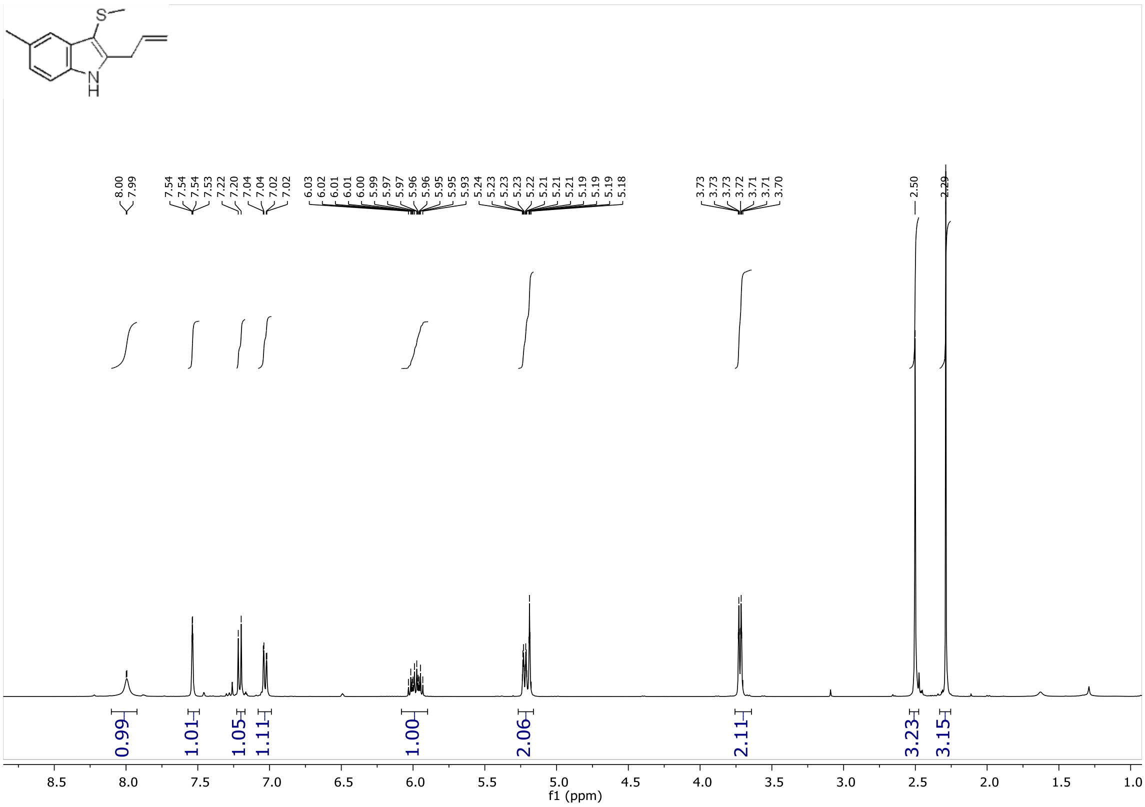

^{13}C NMR (101 MHz, Chloroform-*d*)

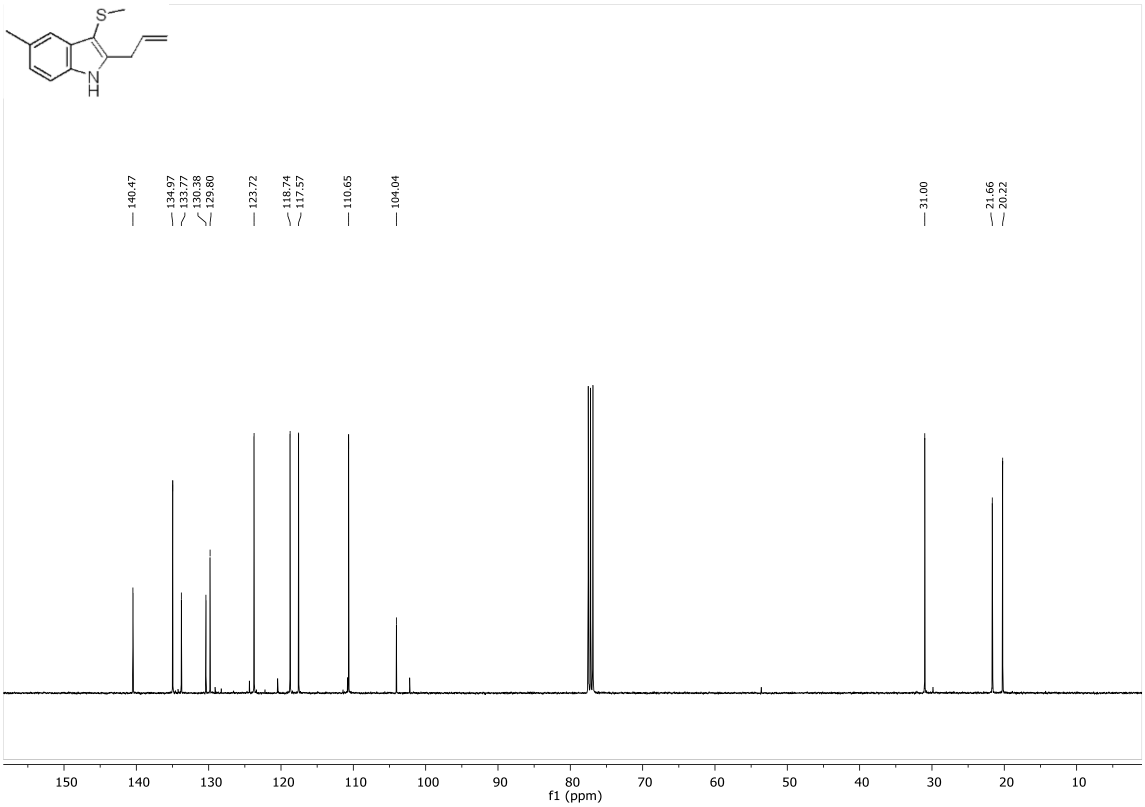

3p

¹H NMR (400 MHz, Chloroform-*d*)

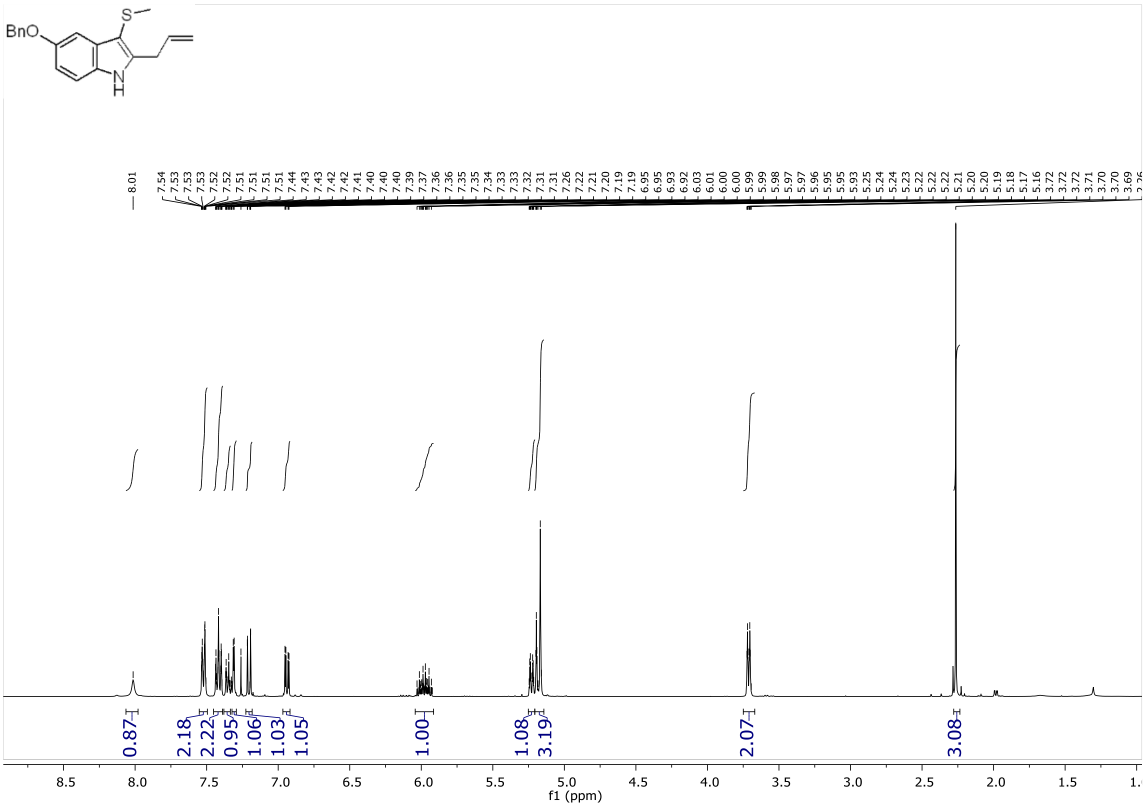

¹³C NMR (101 MHz, Chloroform-*d*)

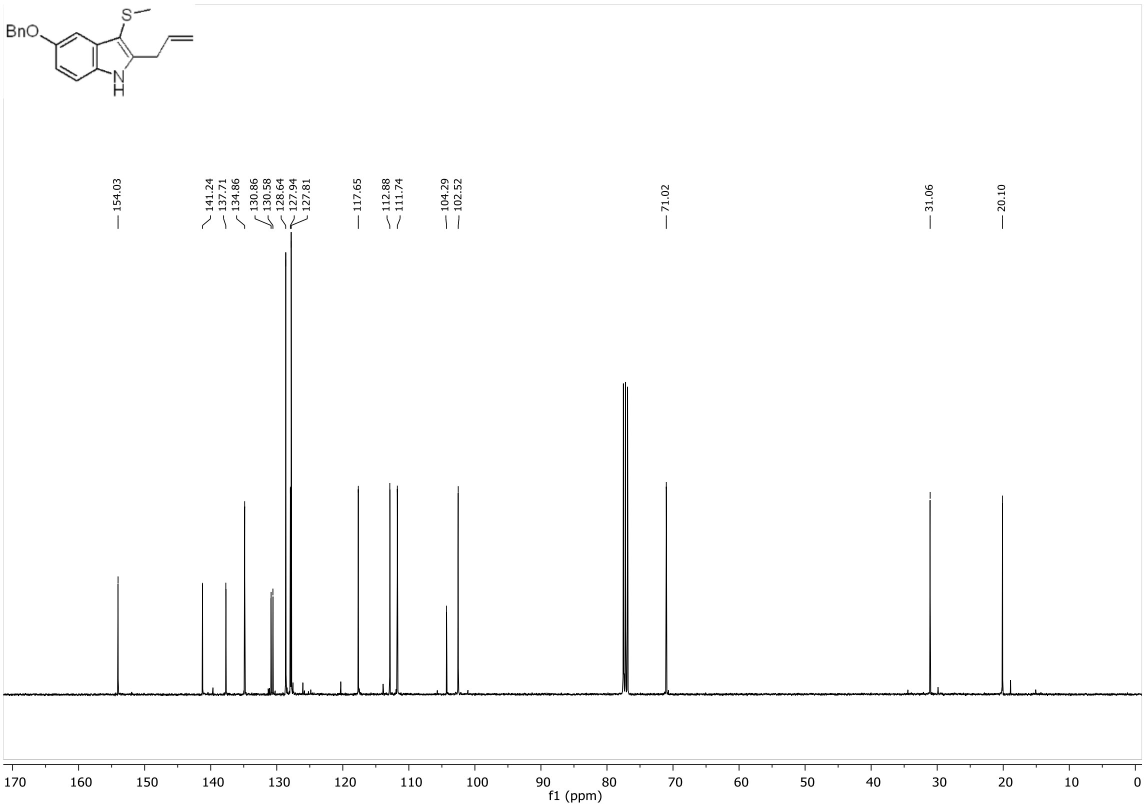

3q

^{1}H NMR (400 MHz, Chloroform-*d*)

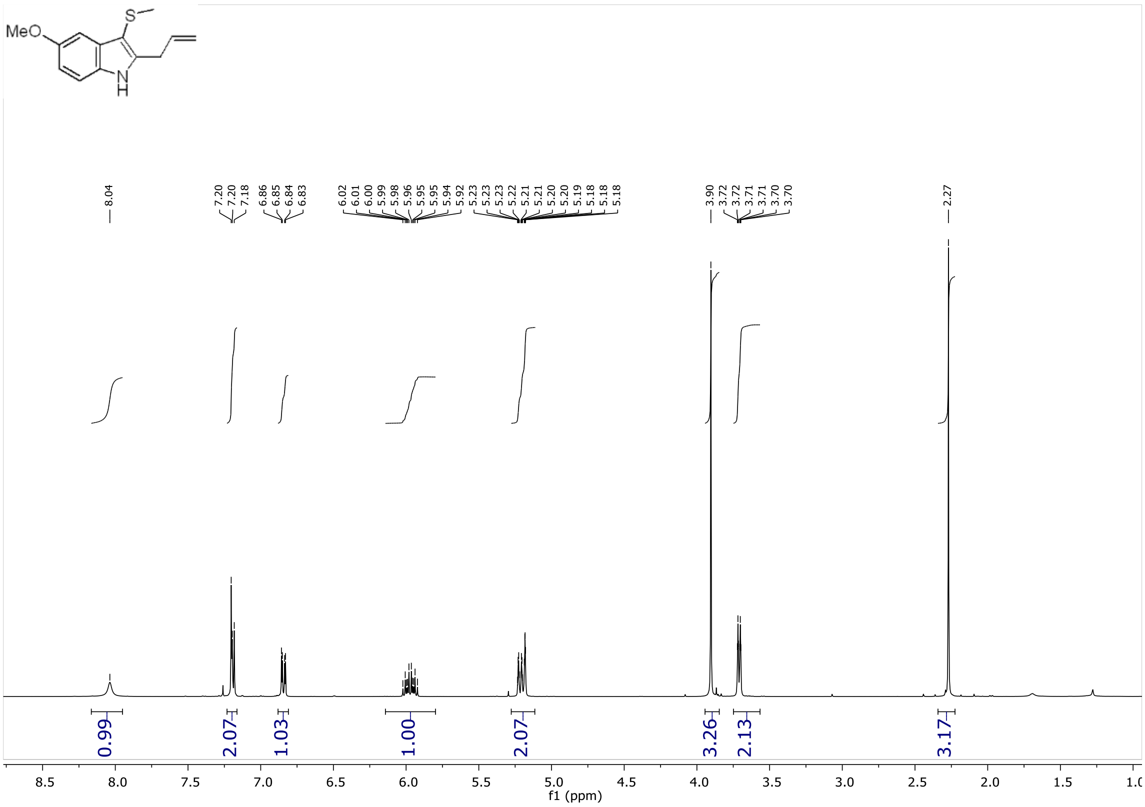

^{13}C NMR (101 MHz, Chloroform-*d*)

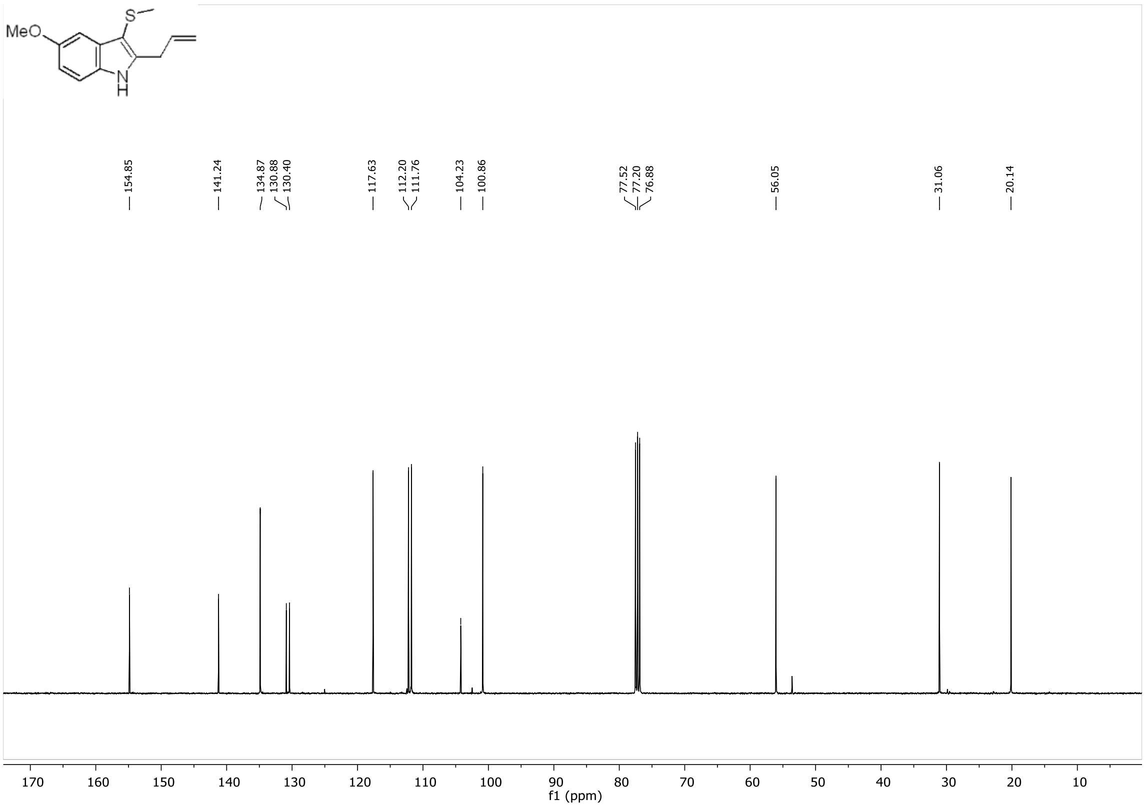

3r

^{1}H NMR (400 MHz, Chloroform-*d*)

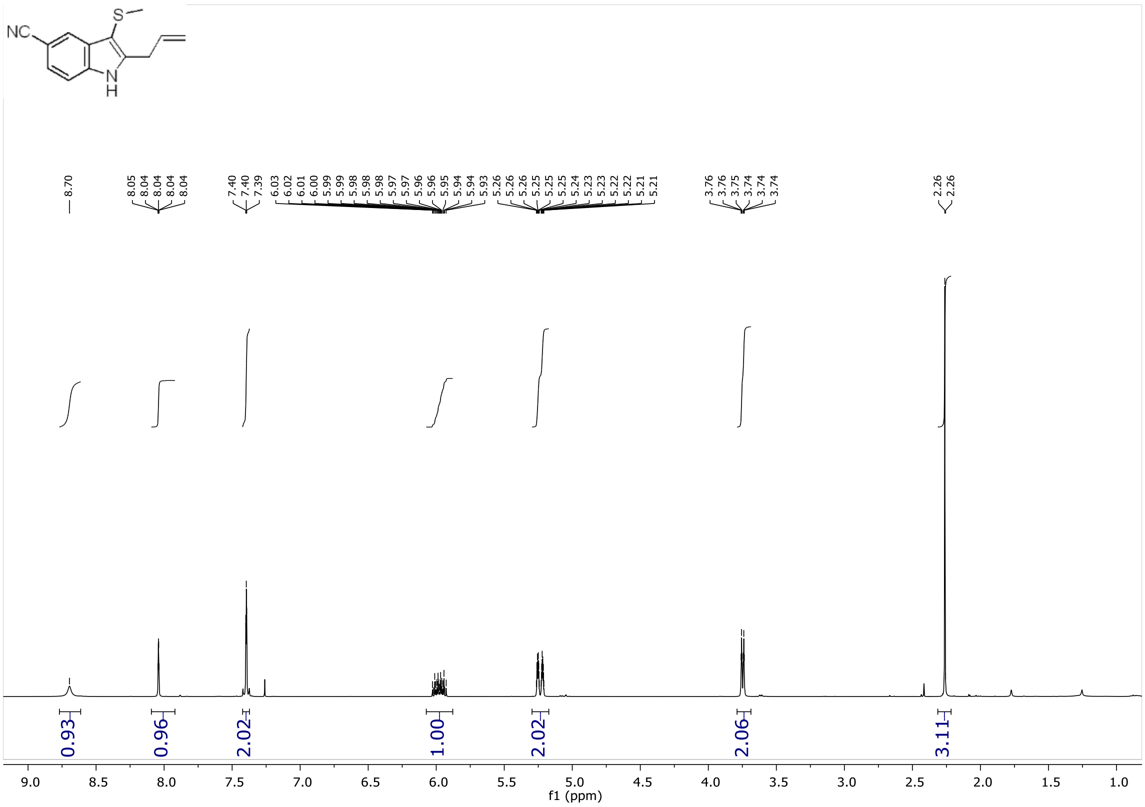

^{13}C NMR (101 MHz, Chloroform-*d*)

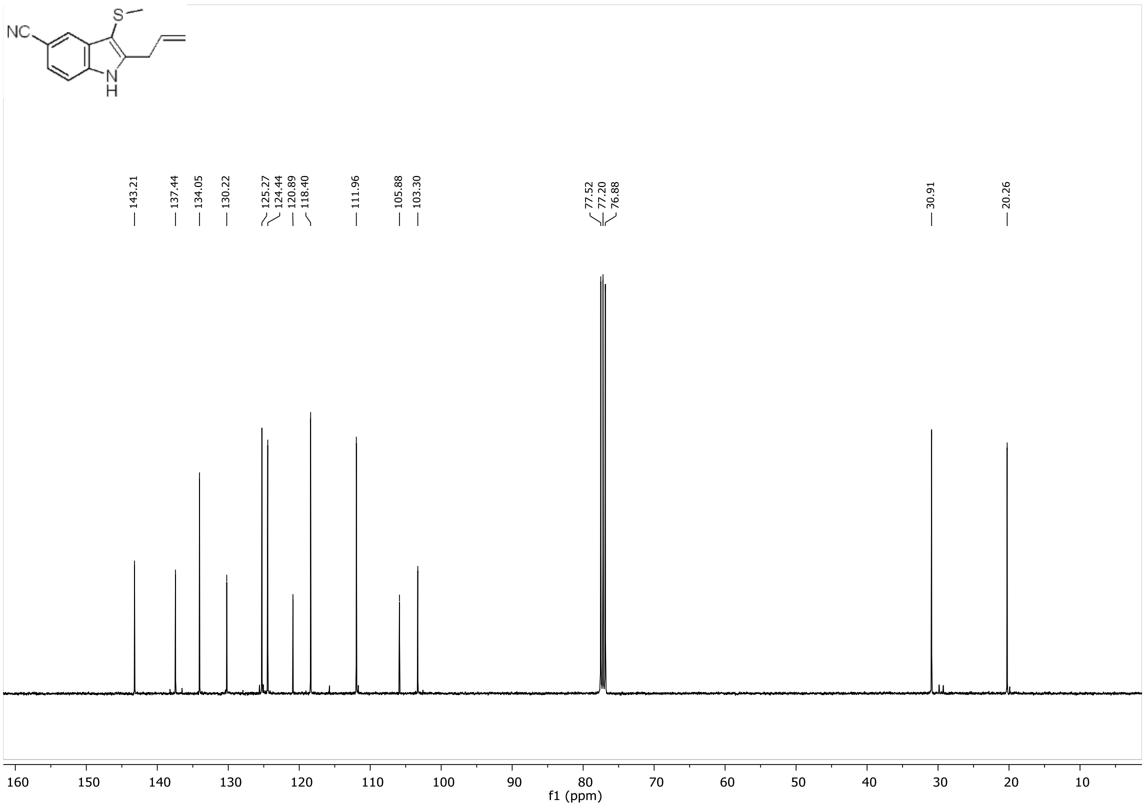

3s

¹H NMR (400 MHz, Chloroform-*d*)

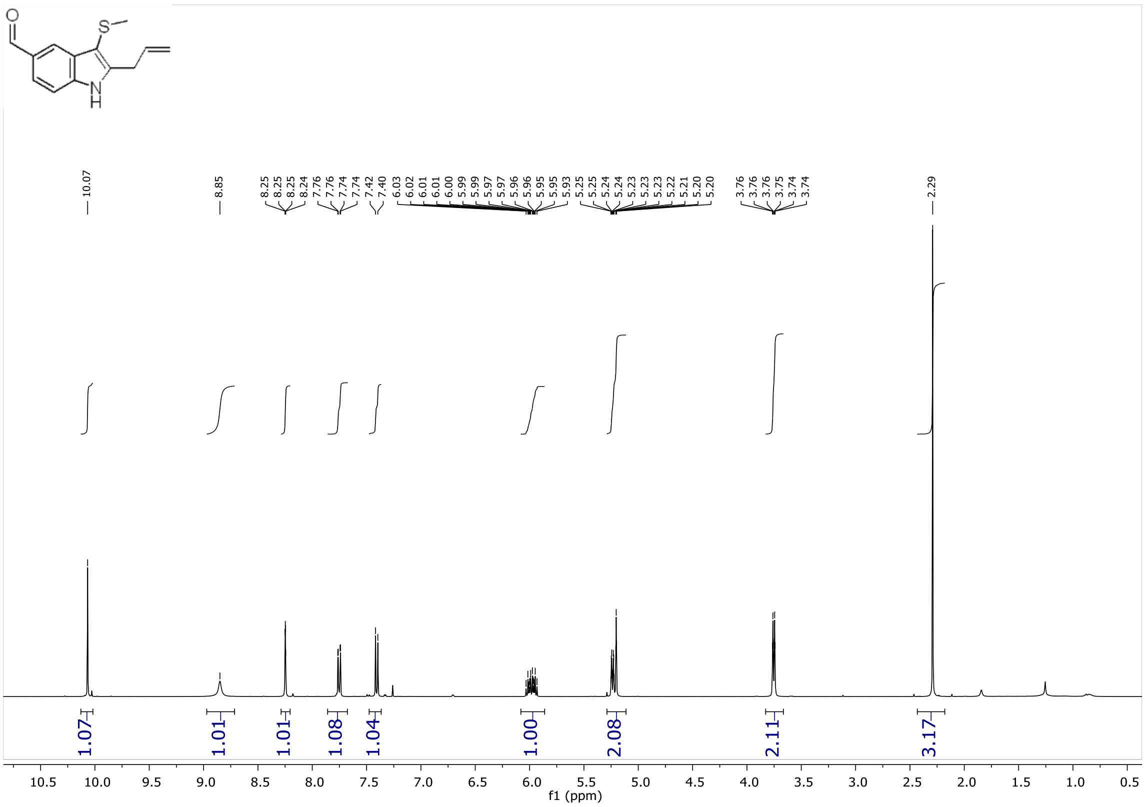

¹³C NMR (101 MHz, Chloroform-*d*)

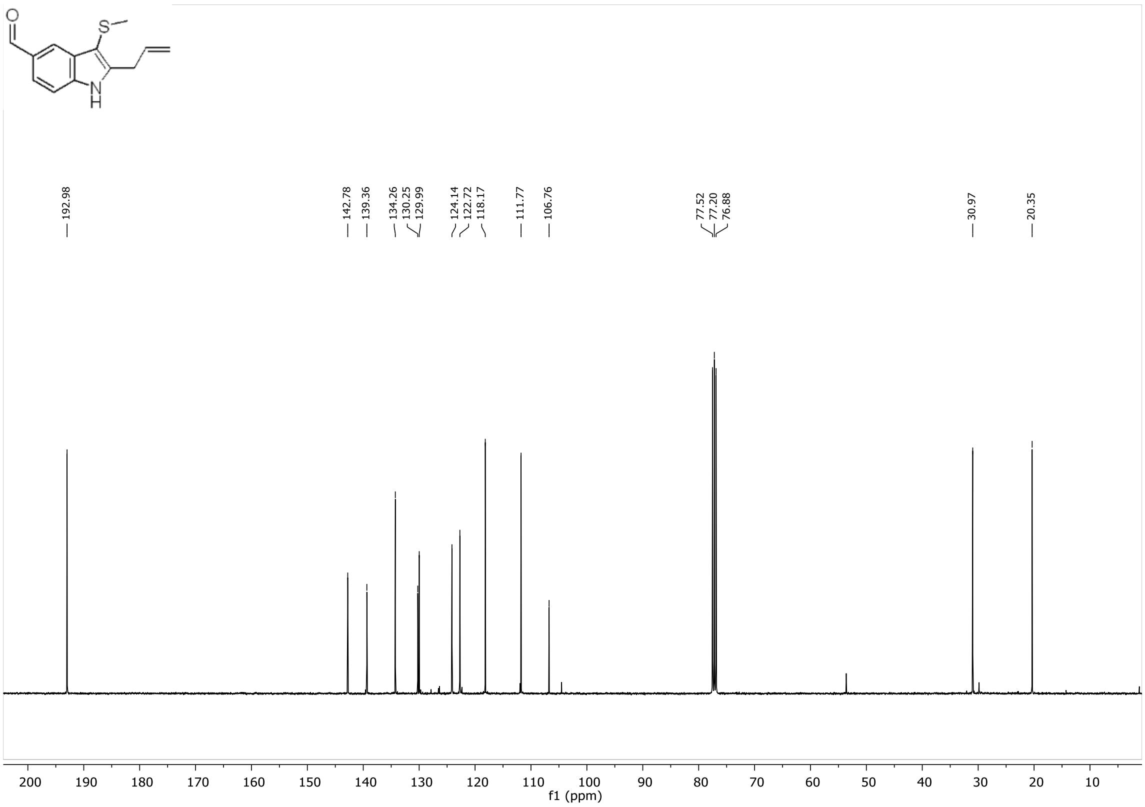

3t

^{1}H NMR (400 MHz, Chloroform-*d*)

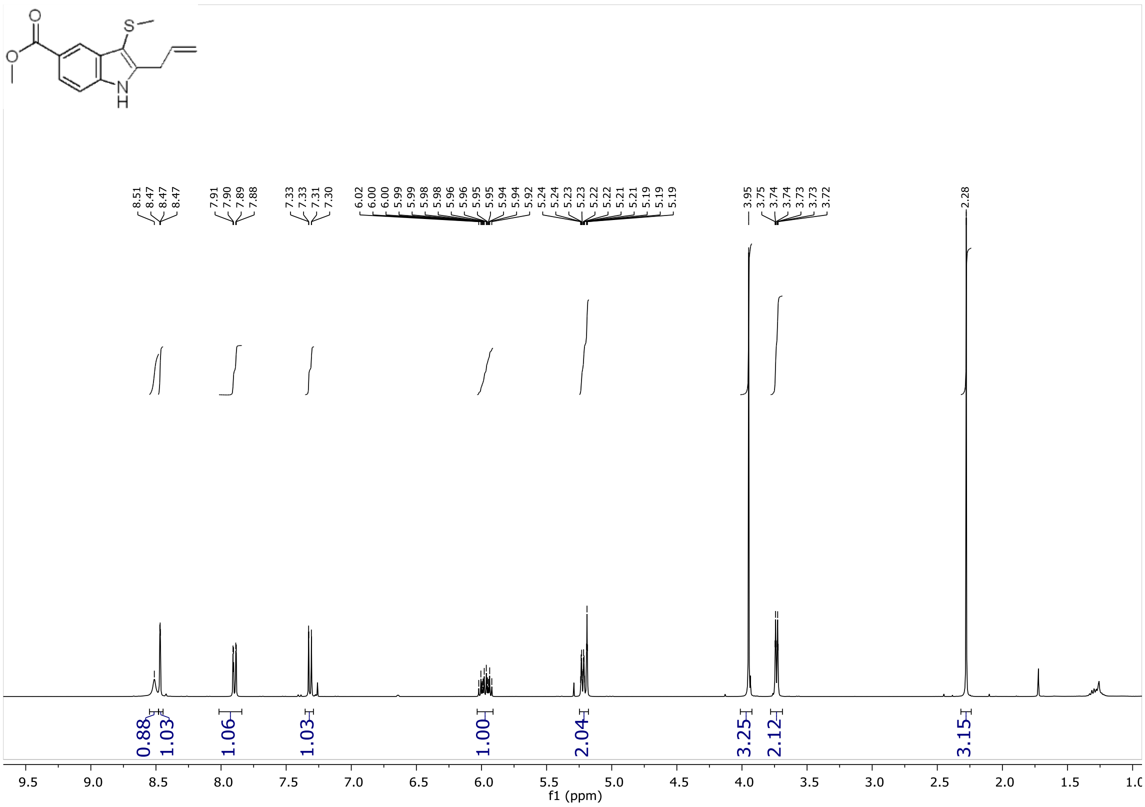

^{13}C NMR (101 MHz, Chloroform-*d*)

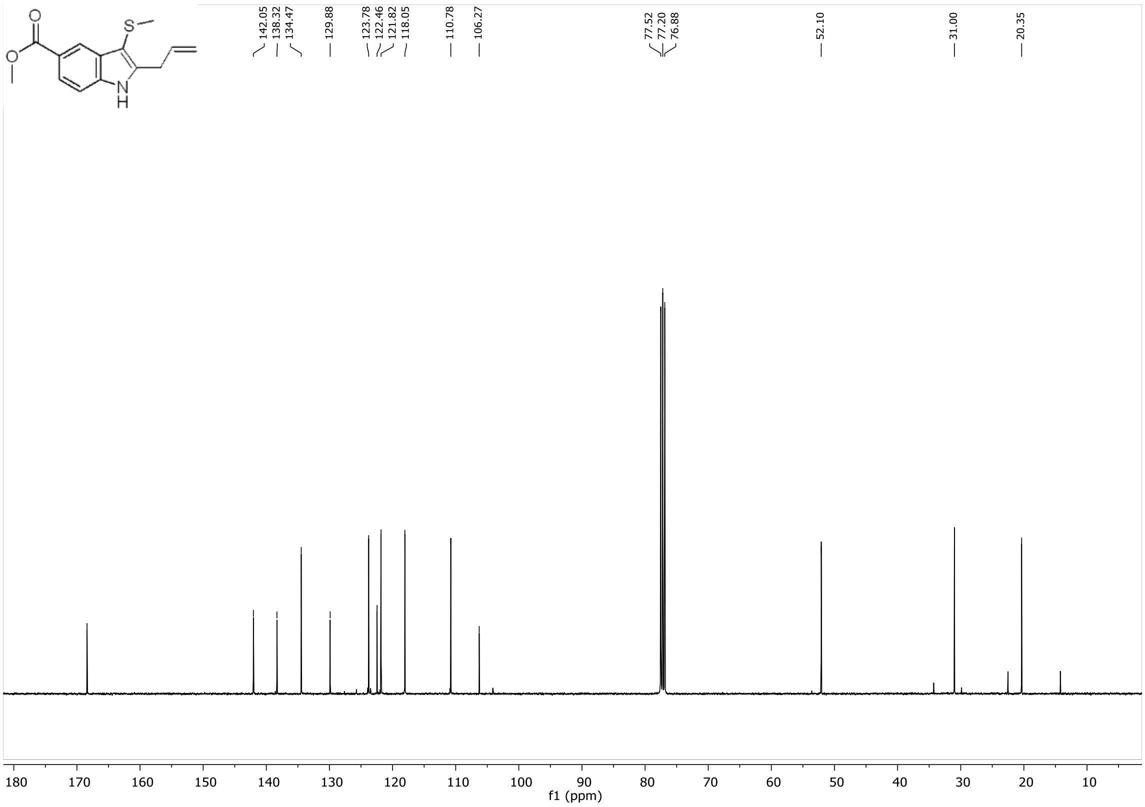

3u

^{1}H NMR (400 MHz, Chloroform-*d*)

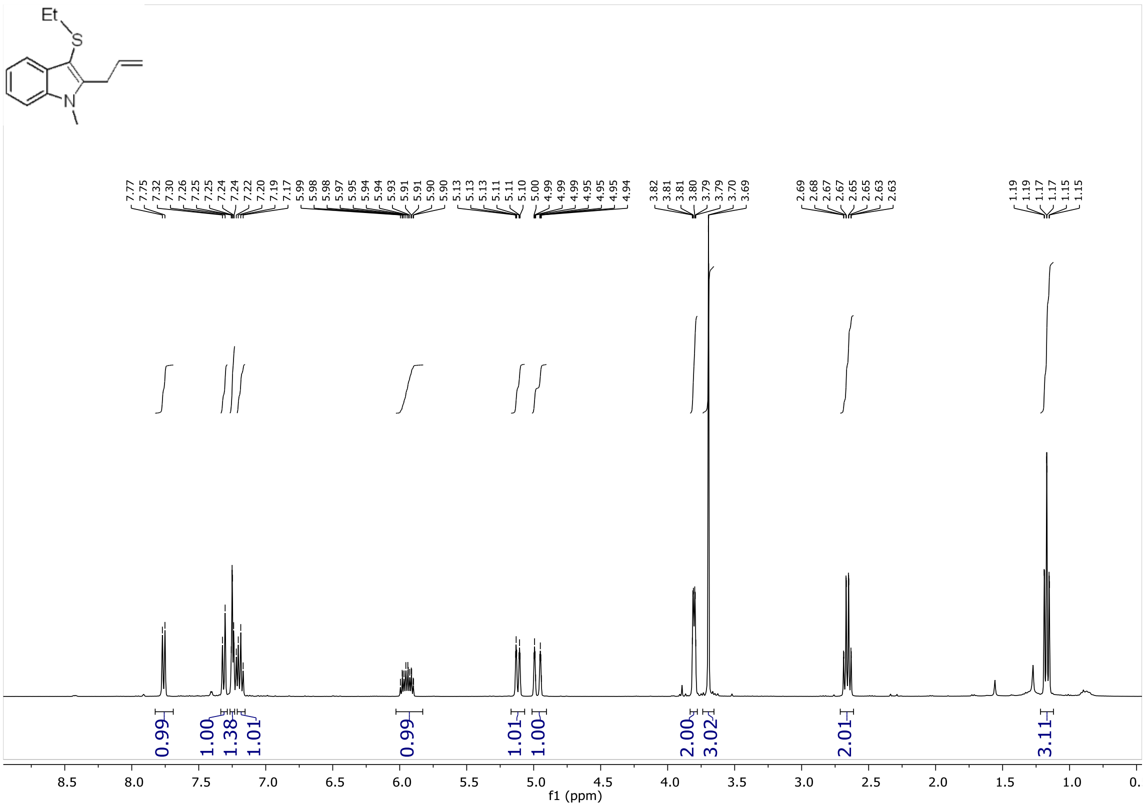

^{13}C NMR (101 MHz, Chloroform-*d*)

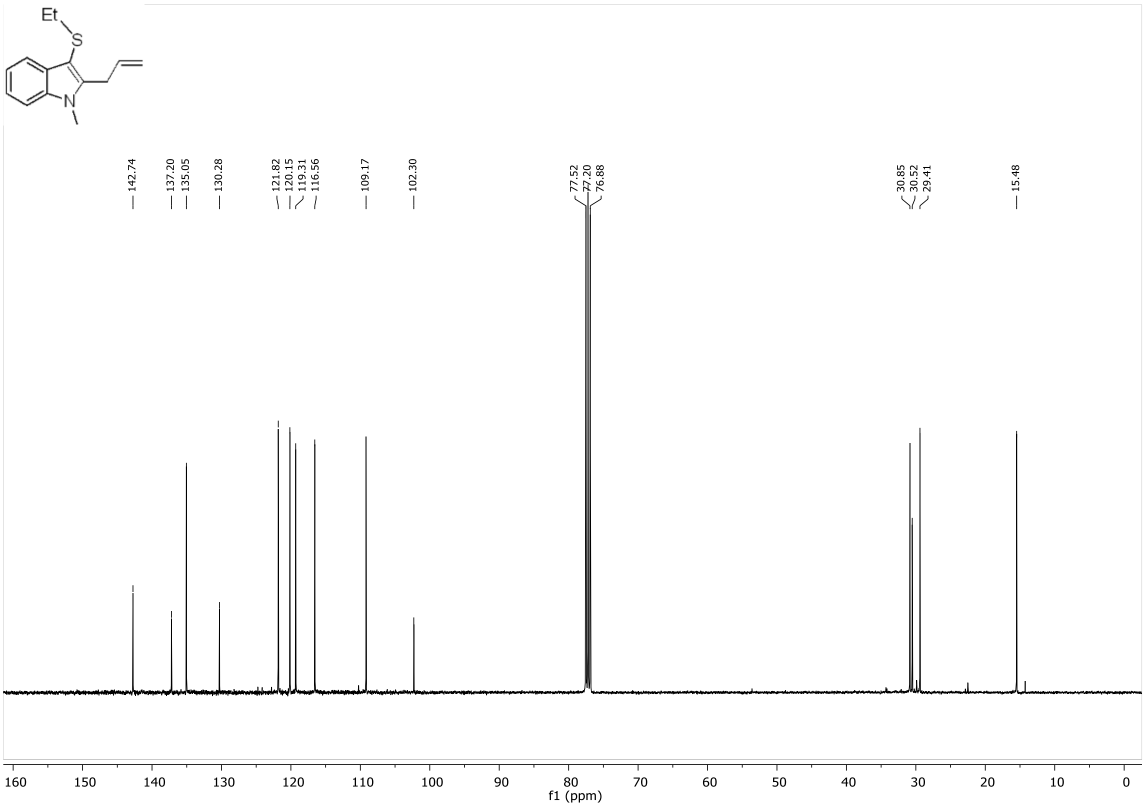

3v

^{1}H NMR (400 MHz, Chloroform-*d*)

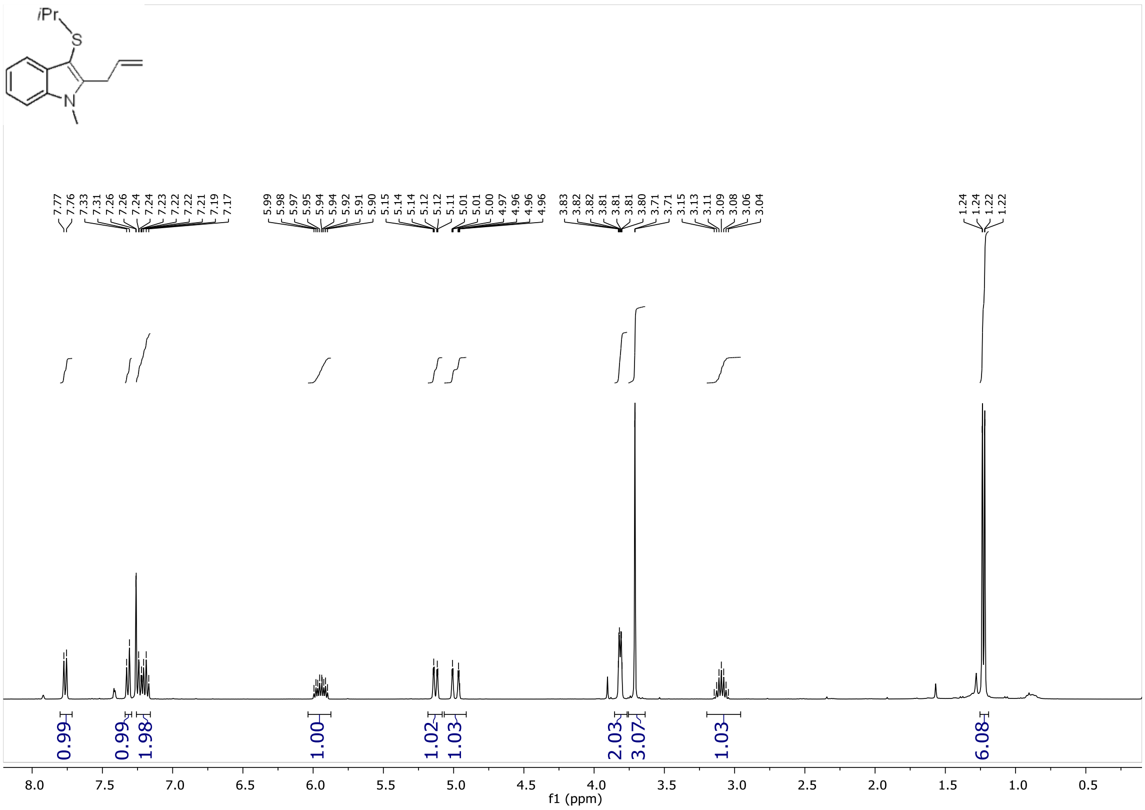

^{13}C NMR (101 MHz, Chloroform-*d*)

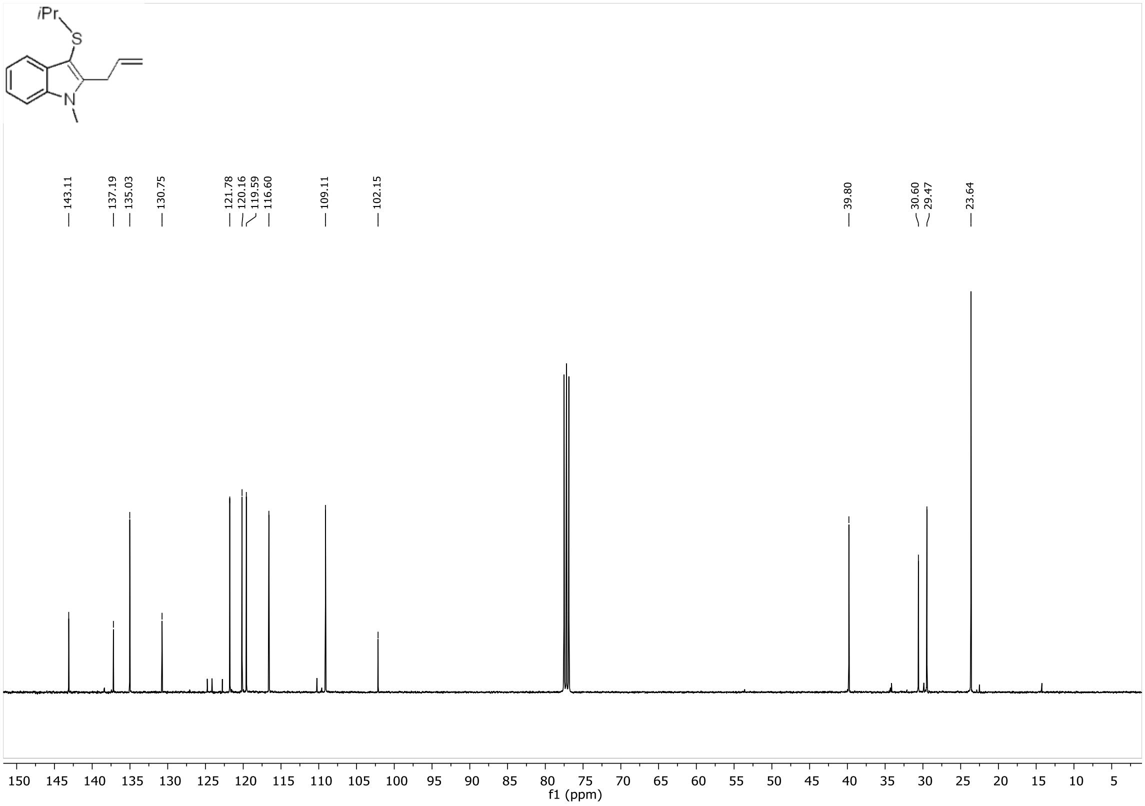

3w

^{1}H NMR (400 MHz, Chloroform-*d*)

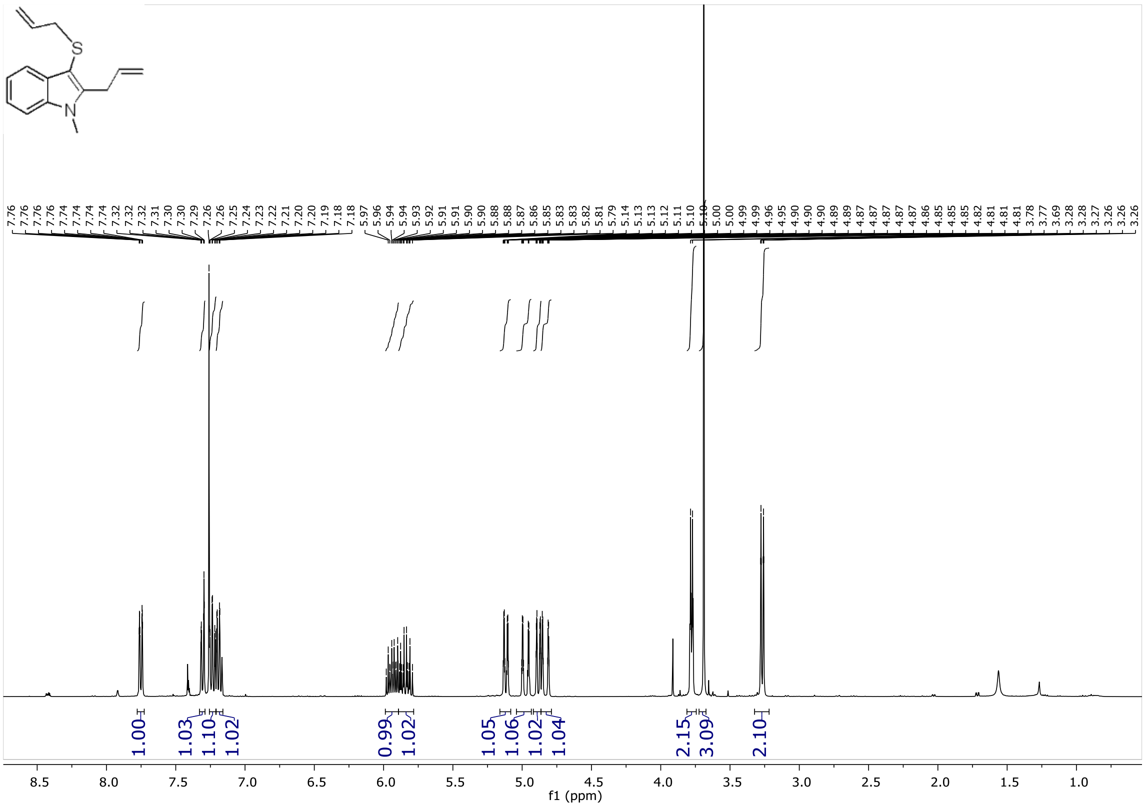

^{13}C NMR (101 MHz, Chloroform-*d*)

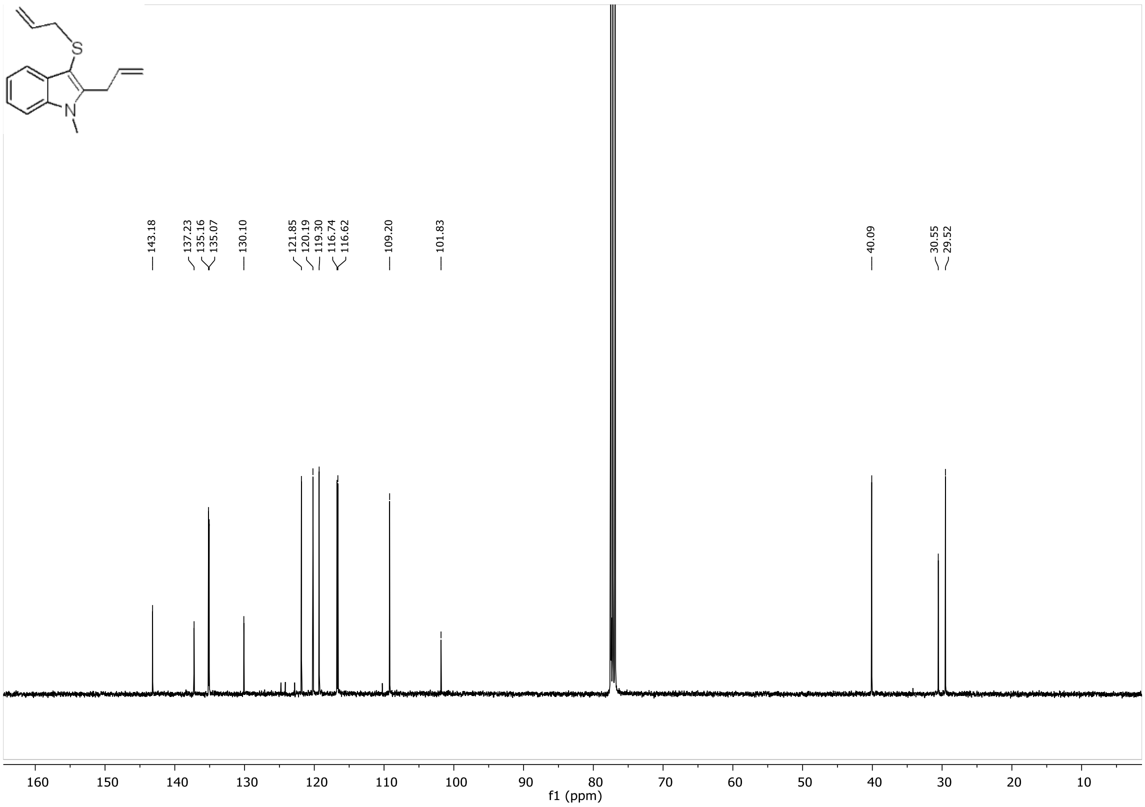

3x

^{1}H NMR (400 MHz, Chloroform-*d*)

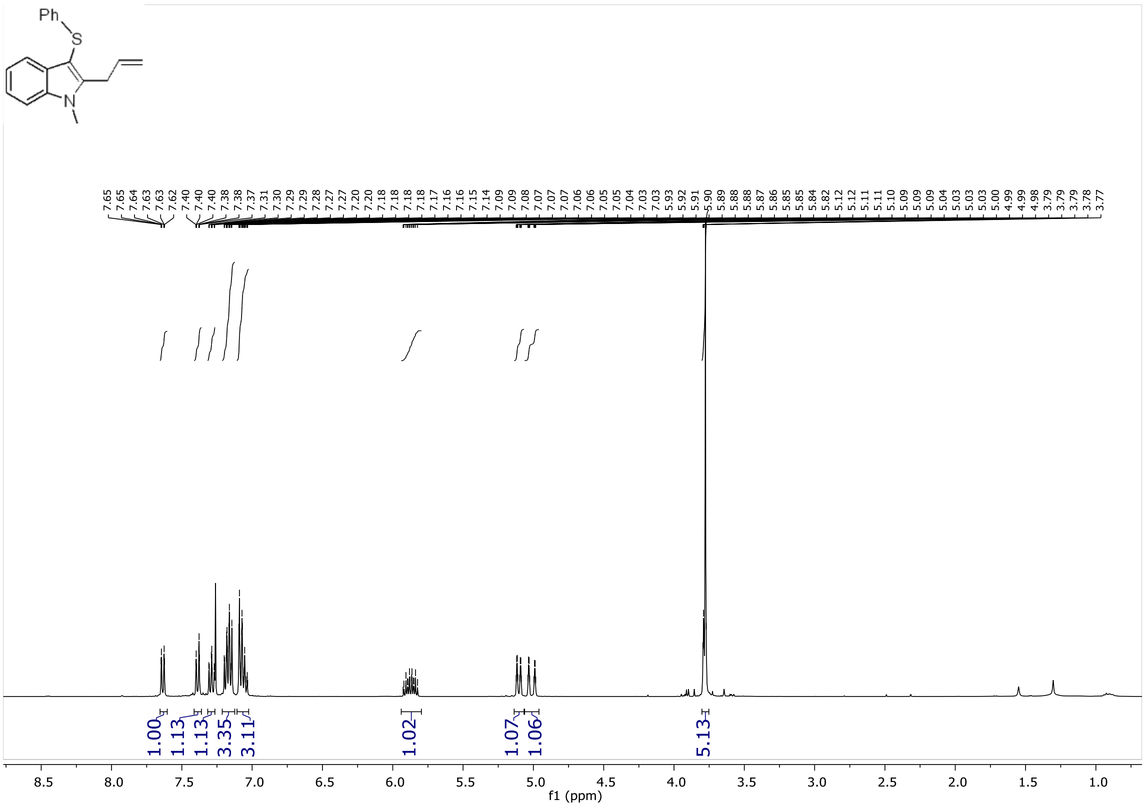

^{13}C NMR (101 MHz, Chloroform-*d*)

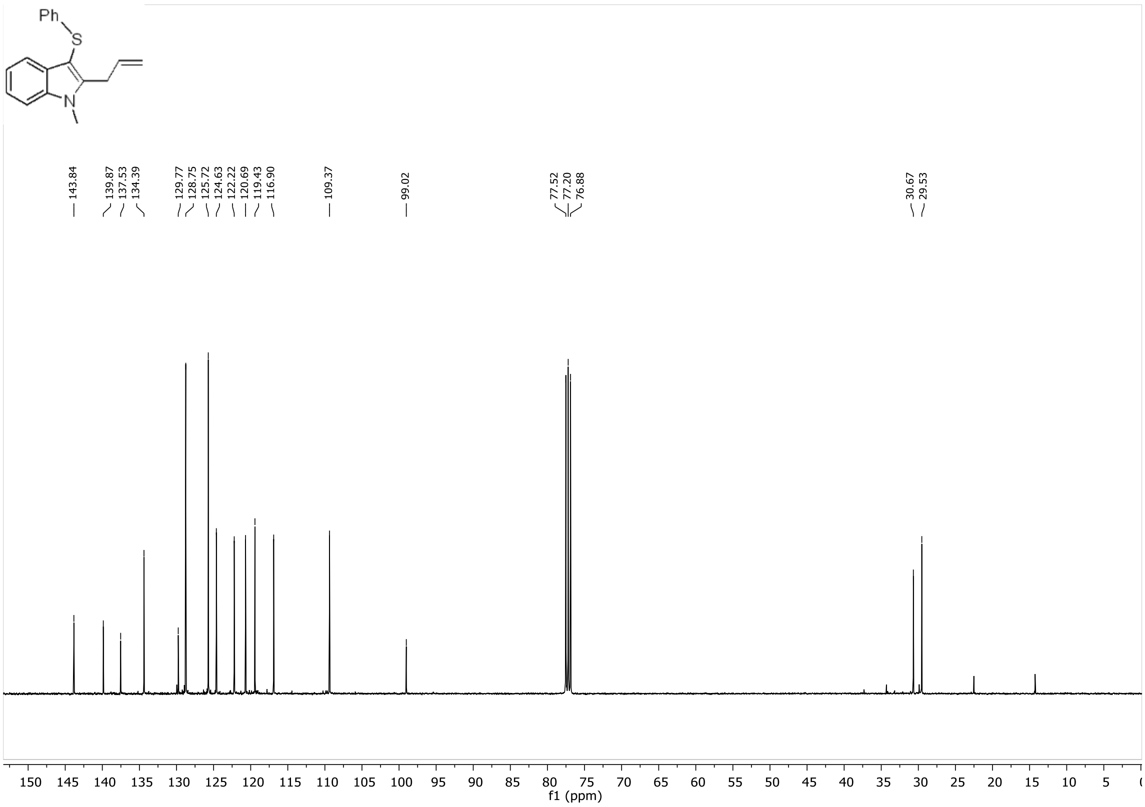

3y

^{1}H NMR (400 MHz, Chloroform-*d*)

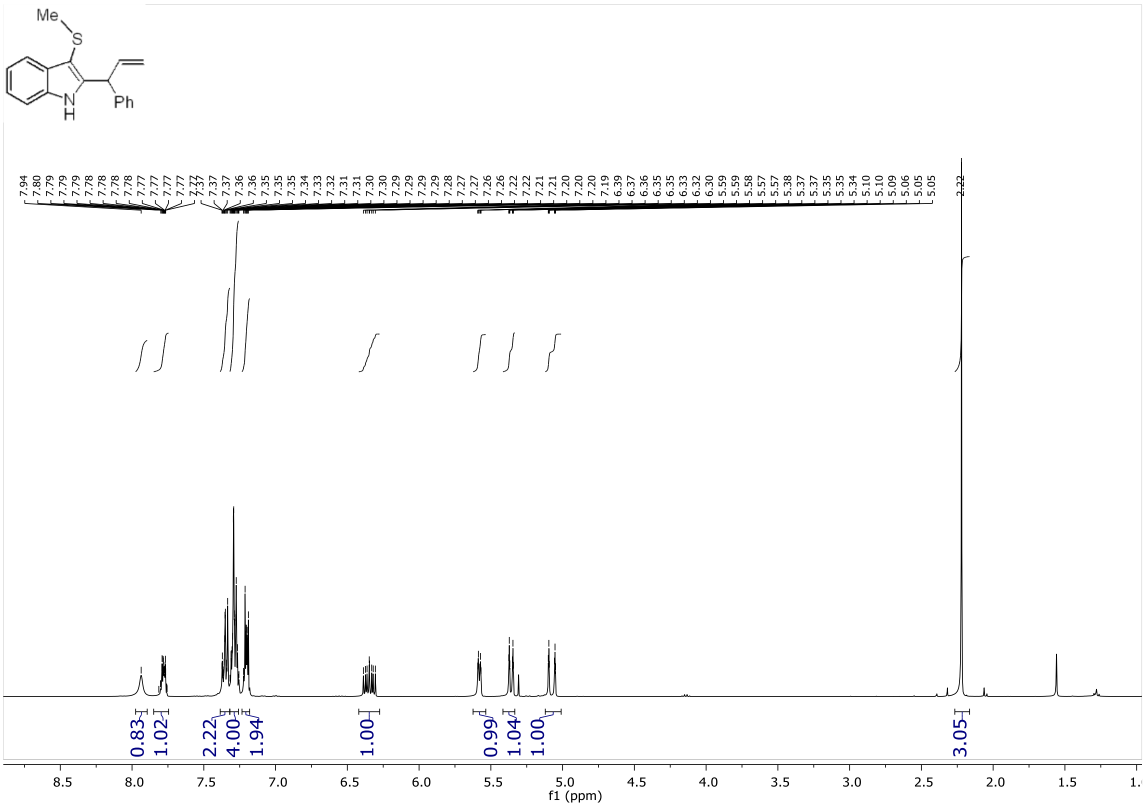

^{13}C NMR (101 MHz, Chloroform-*d*)

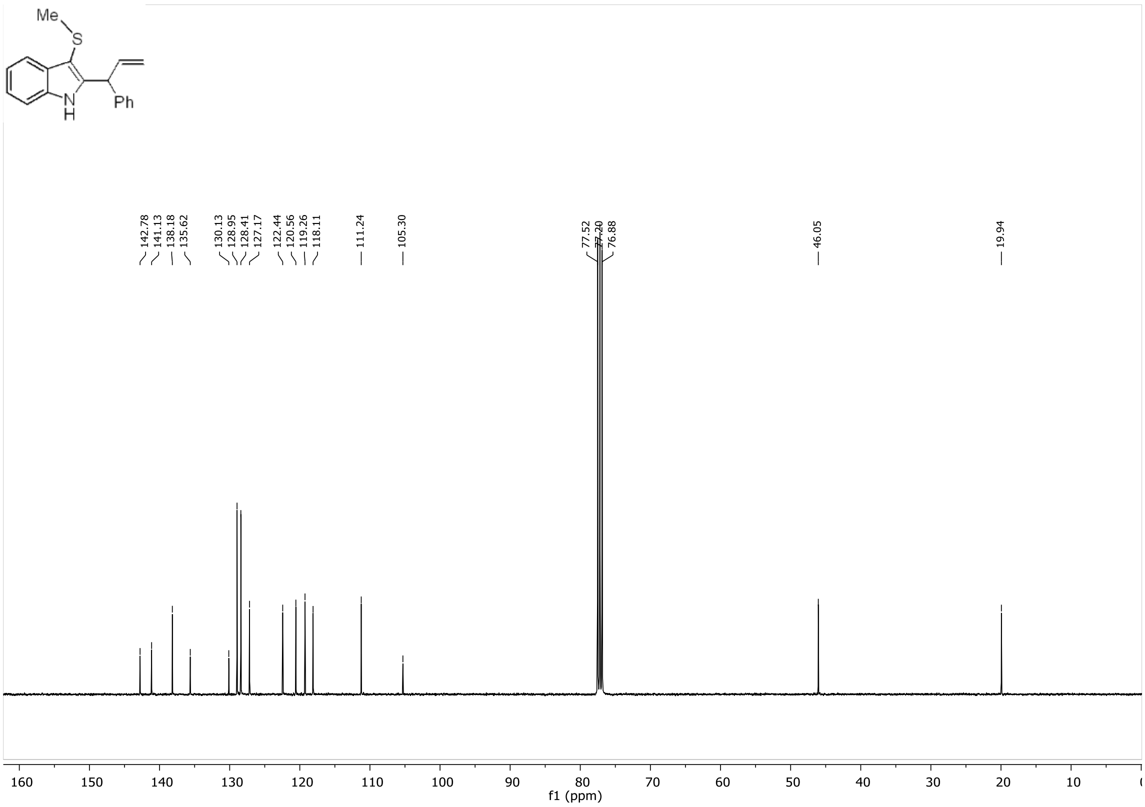

4y

^{1}H NMR (400 MHz, Chloroform-*d*)

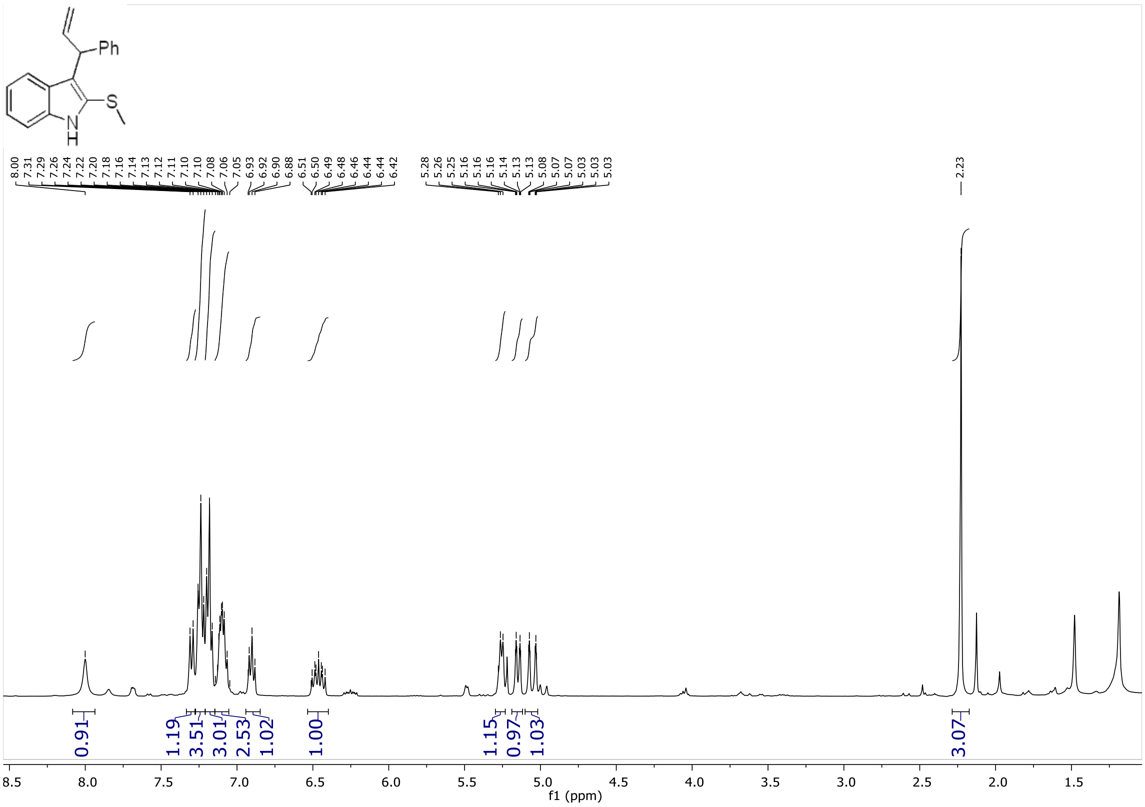

^{13}C NMR (101 MHz, Chloroform-*d*)

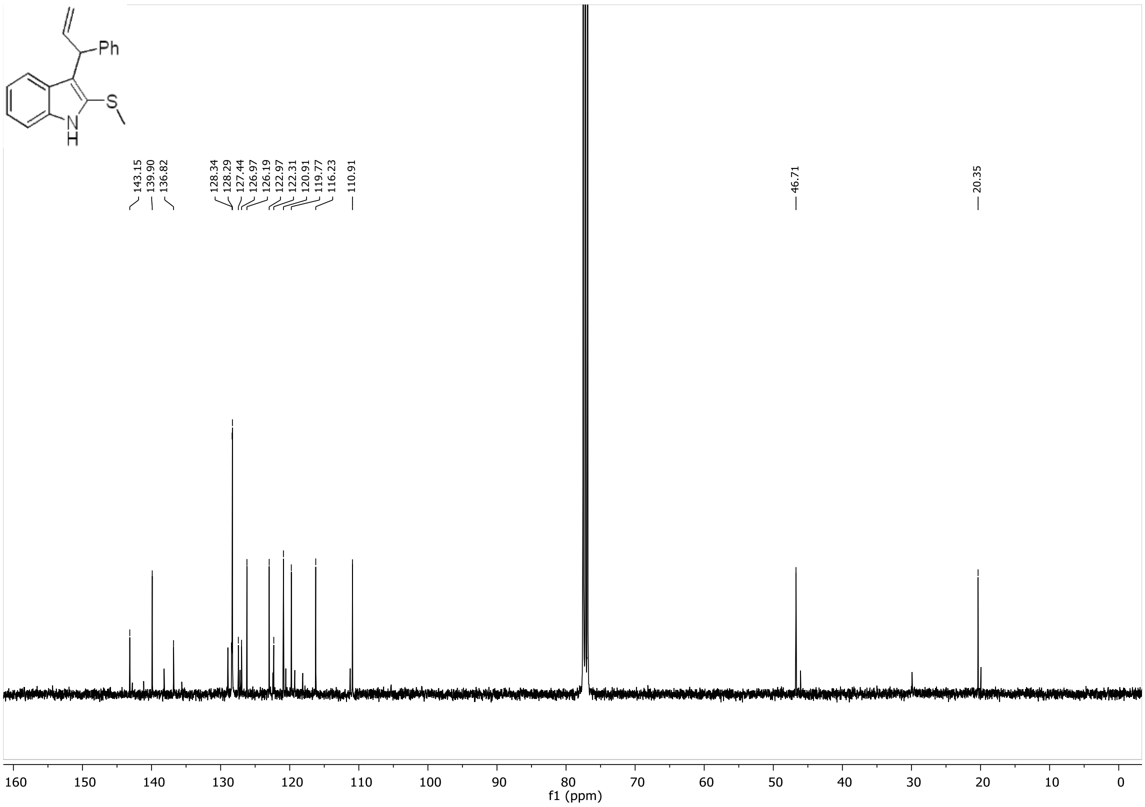

3z

¹H NMR (400 MHz, Chloroform-*d*)

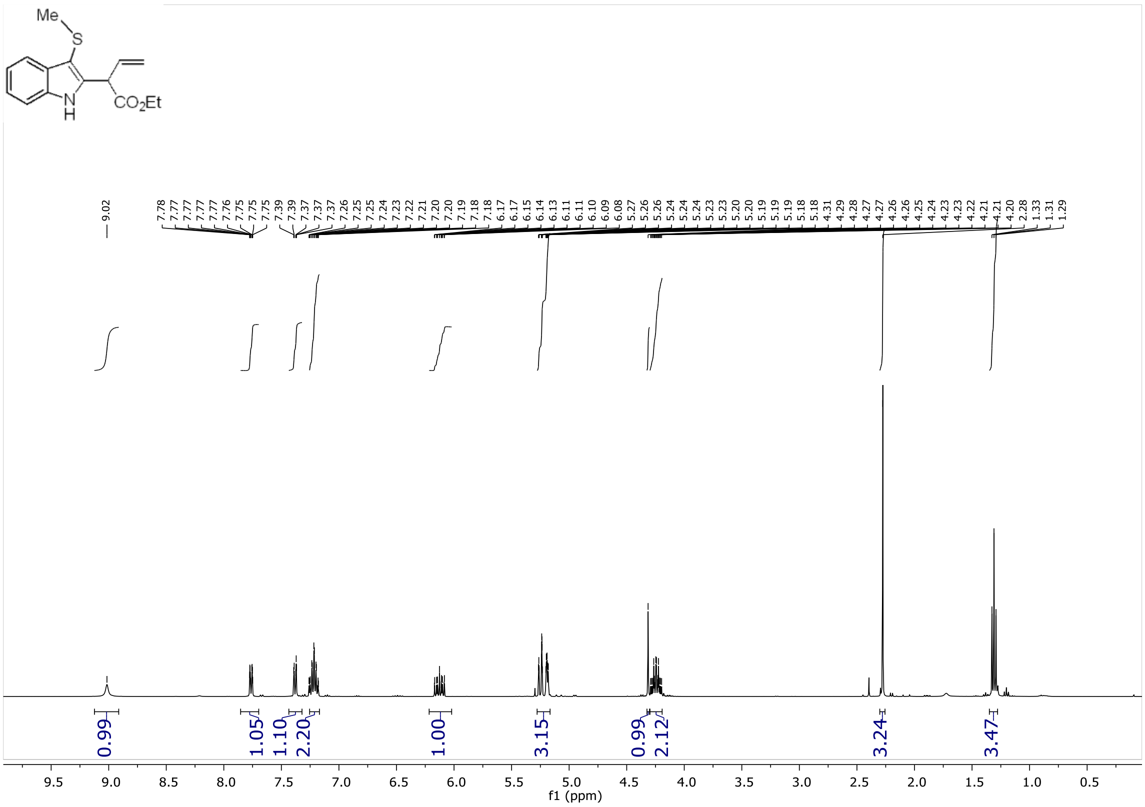

¹³C NMR (101 MHz, Chloroform-*d*)

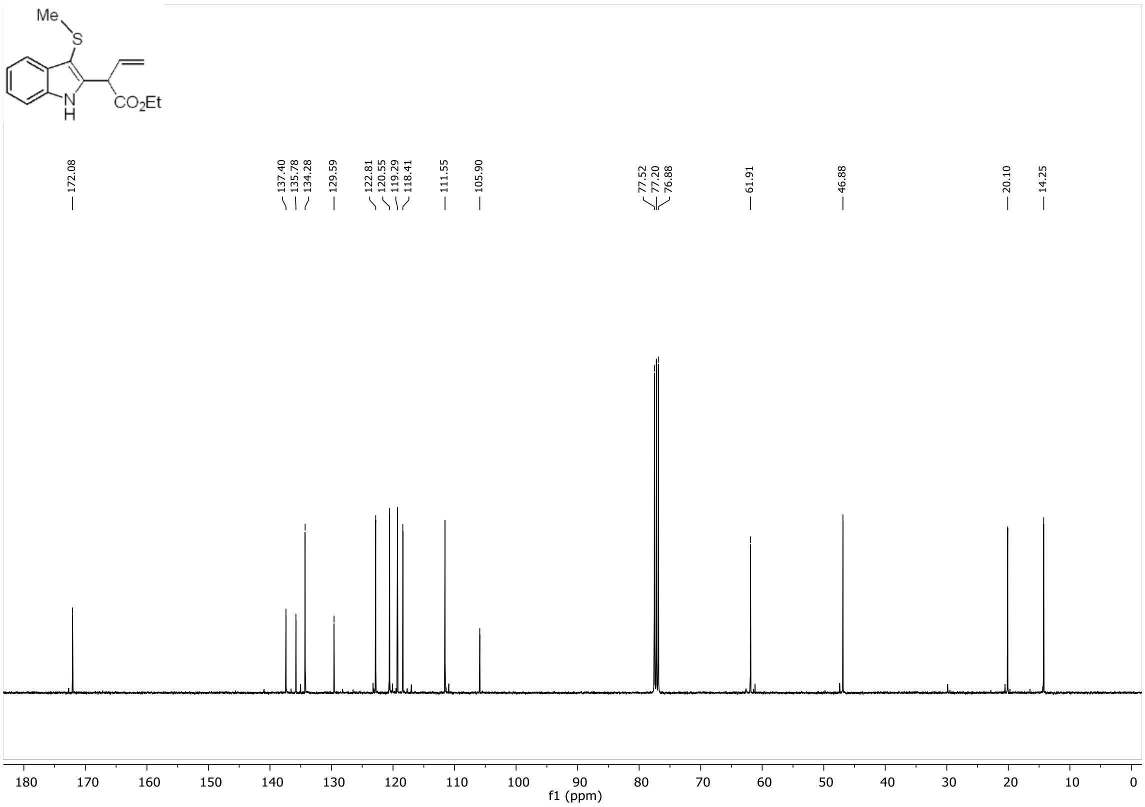

3aa 4aa

^{1}H NMR (400 MHz, Chloroform-*d*)

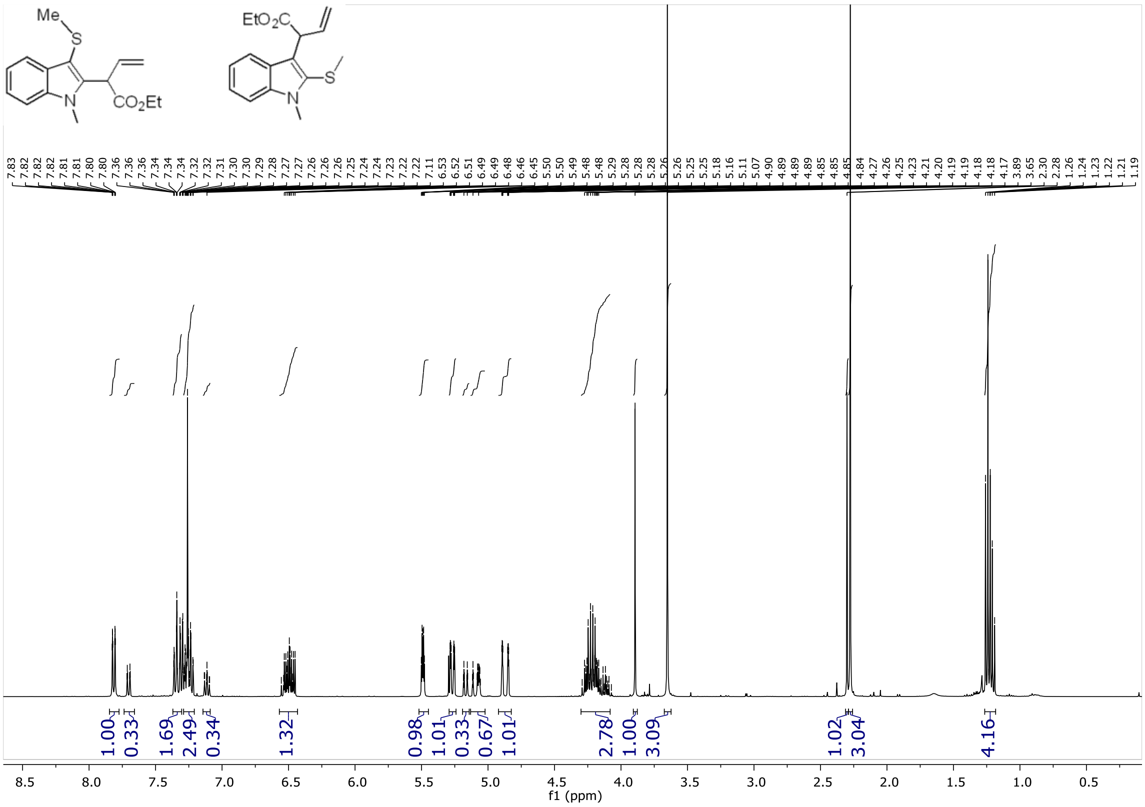

^{13}C NMR (101 MHz, Chloroform-*d*)

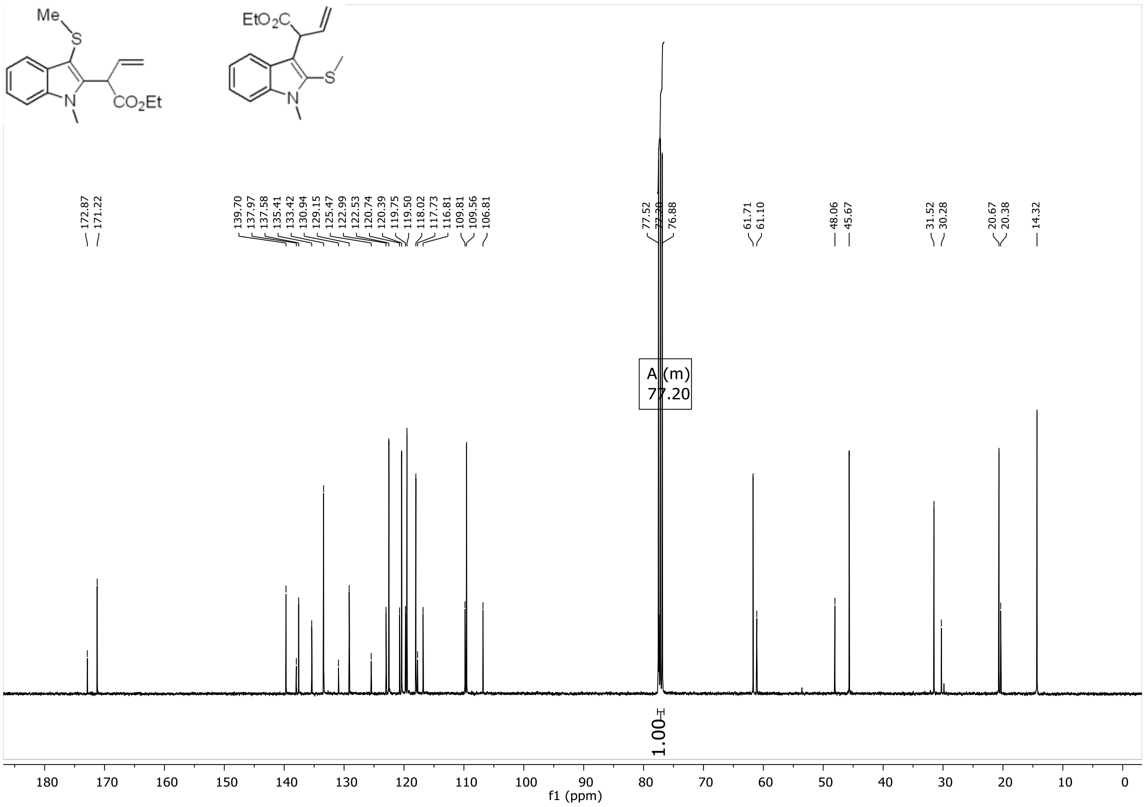

^{1}H-^{13}C HMBC

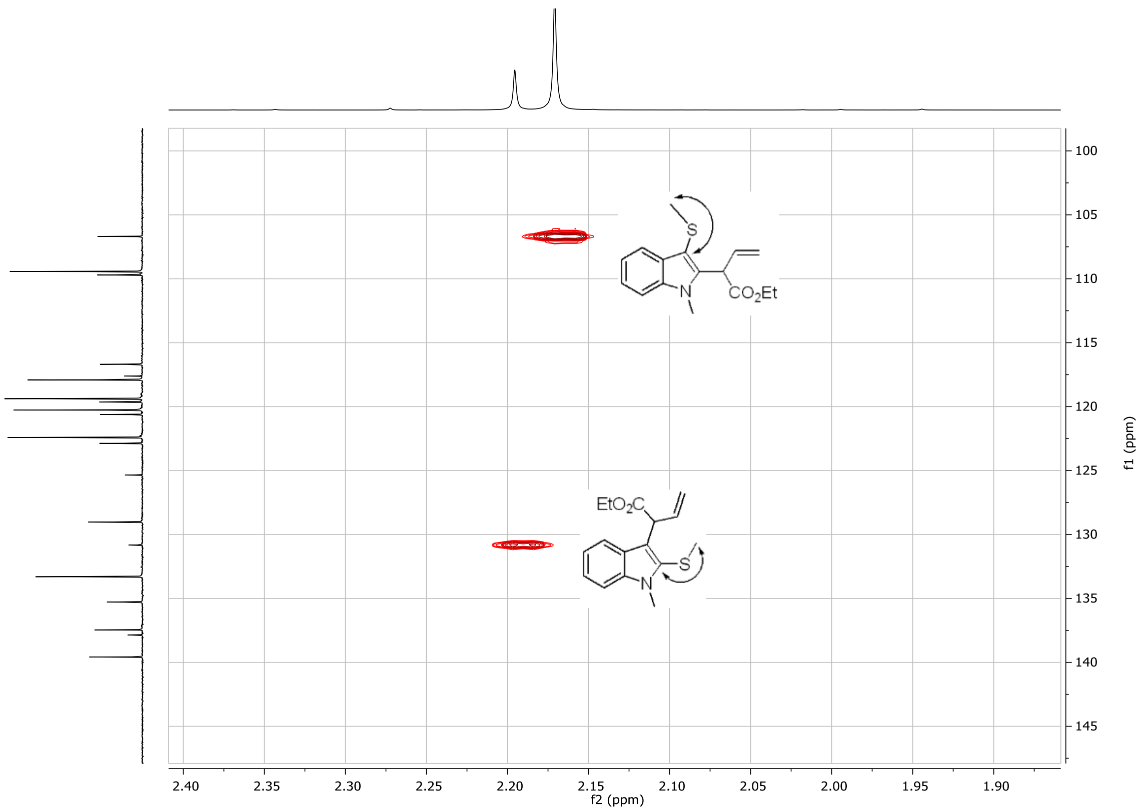
S
CO_2Et
N
EtO_2C
S
N
100
105
110
115
120
125
130
135
140
145
f1 (ppm)
2.40
2.35
2.30
2.25
2.20
2.15
2.10
2.05
2.00
1.95
1.90
f2 (ppm)

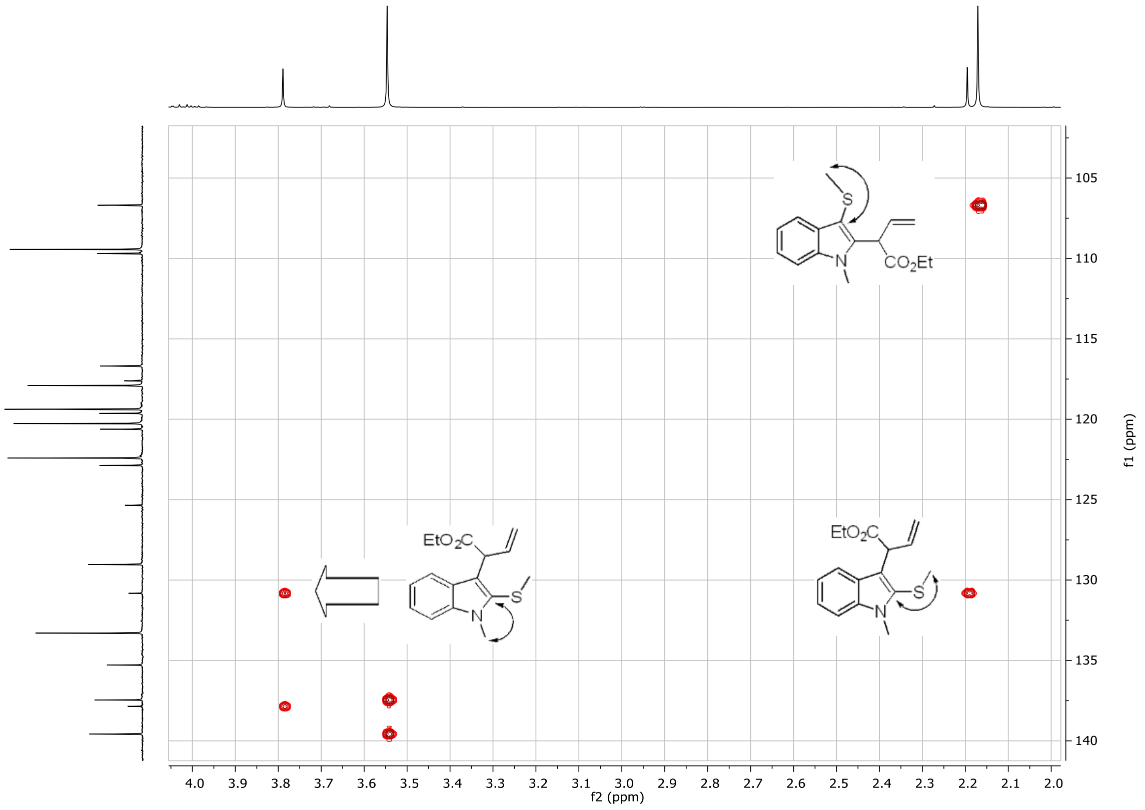
S
N
CO_2Et
EtO_2C
S
N
EtO_2C
S
N
105
110
115
120
125
130
135
140
f1 (ppm)
4.0
3.9
3.8
3.7
3.6
3.5
3.4
3.3
3.2
3.1
3.0
2.9
2.8
2.7
2.6
2.5
2.4
2.3
2.2
2.1
2.0
f2 (ppm)

3ab

^{1}H NMR (400 MHz, Chloroform-*d*)

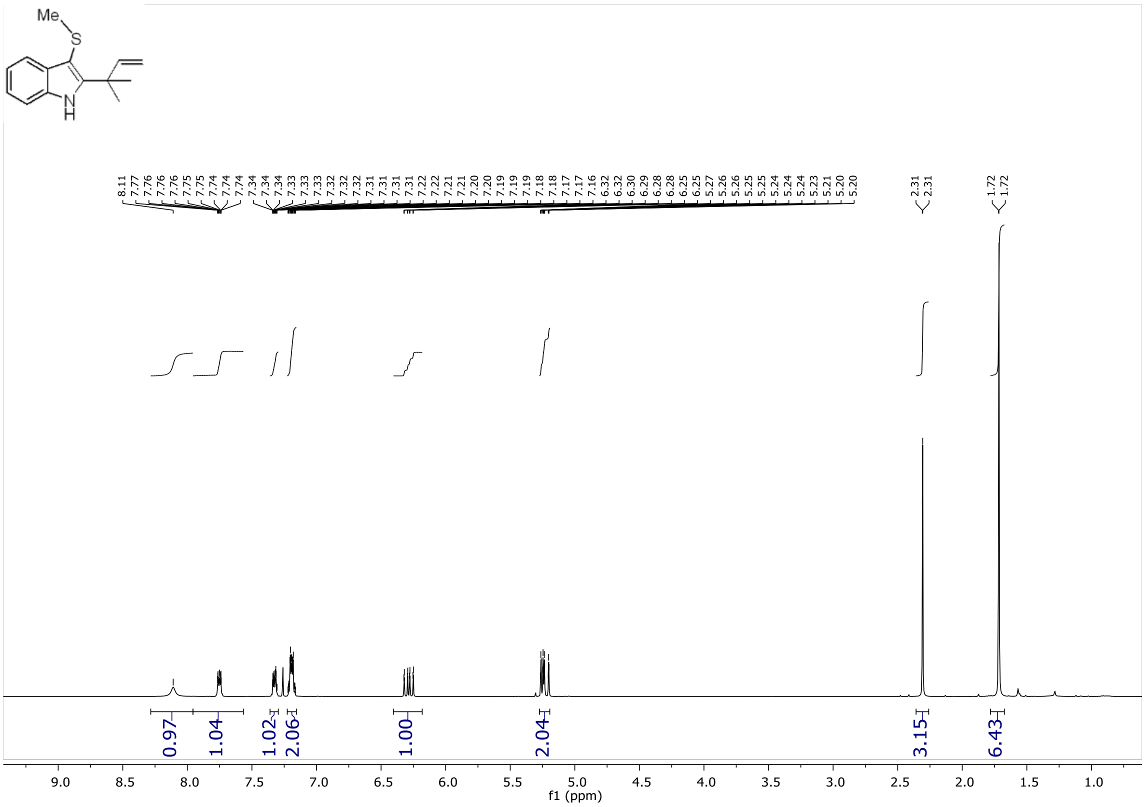

^{13}C NMR (101 MHz, Chloroform-*d*)

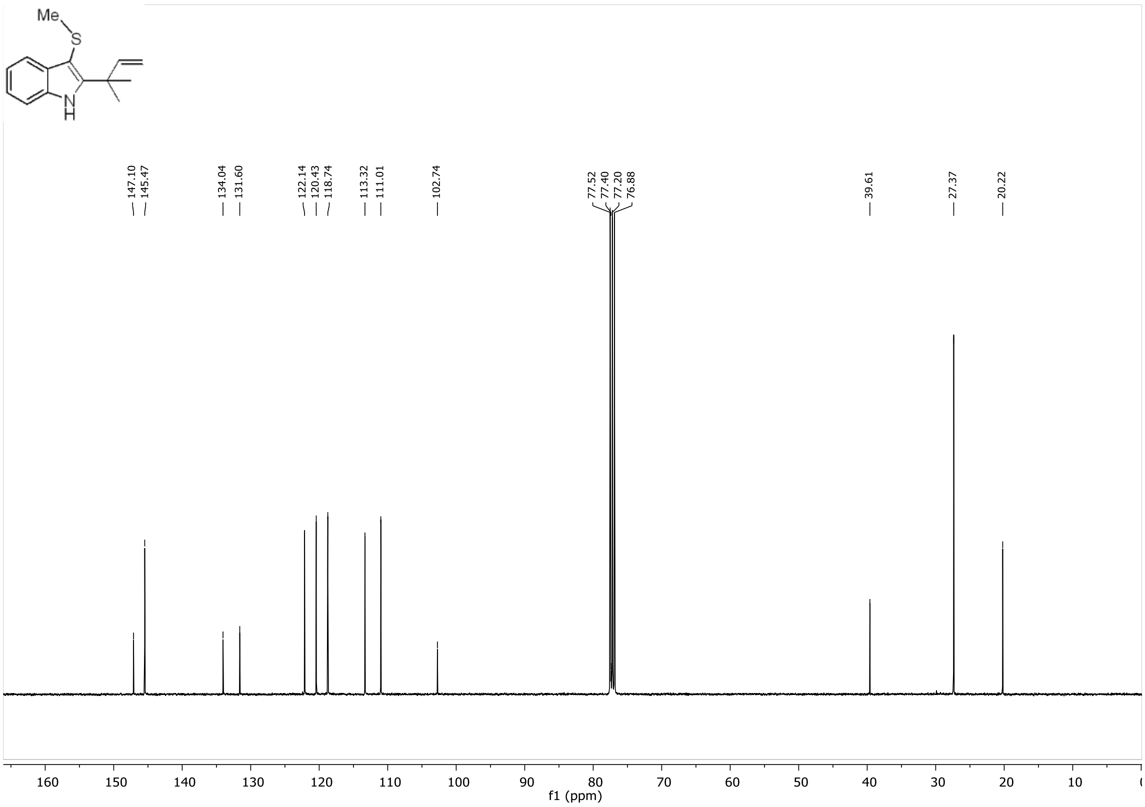

3ac 4ac

^{1}**H NMR** (400 MHz, Chloroform-*d*)

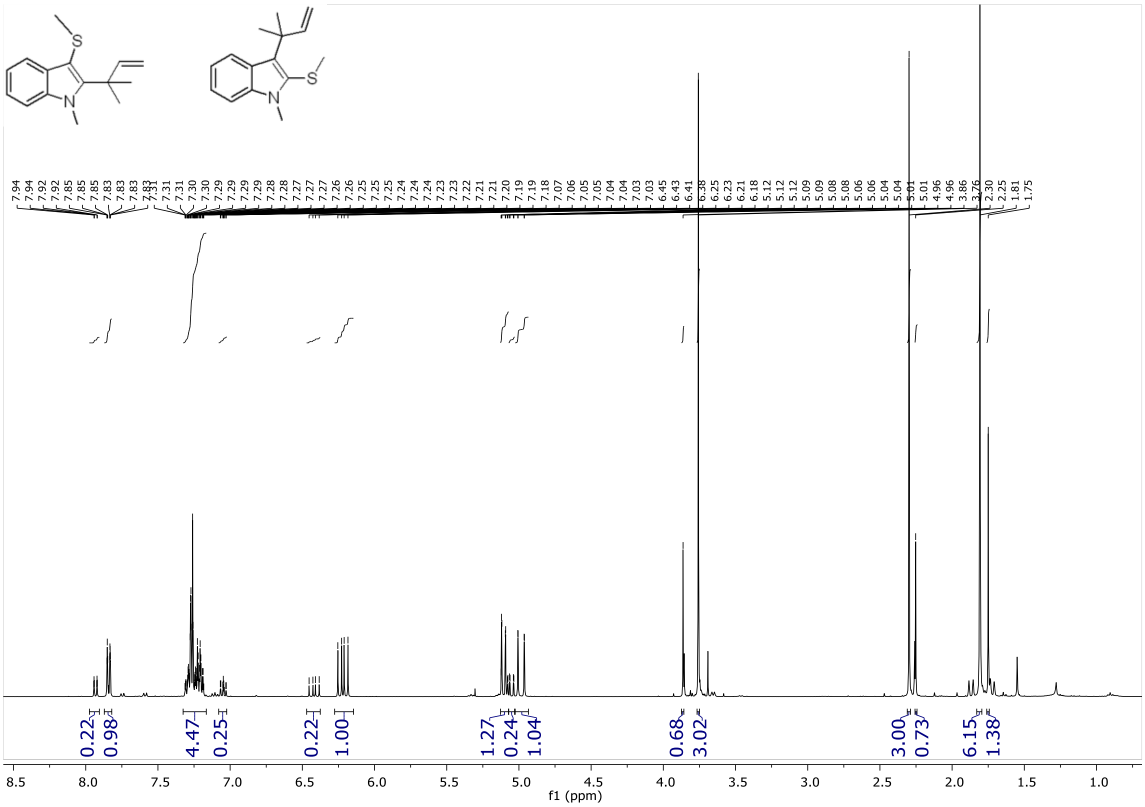

^{13}**C NMR** (101 MHz, Chloroform-*d*)

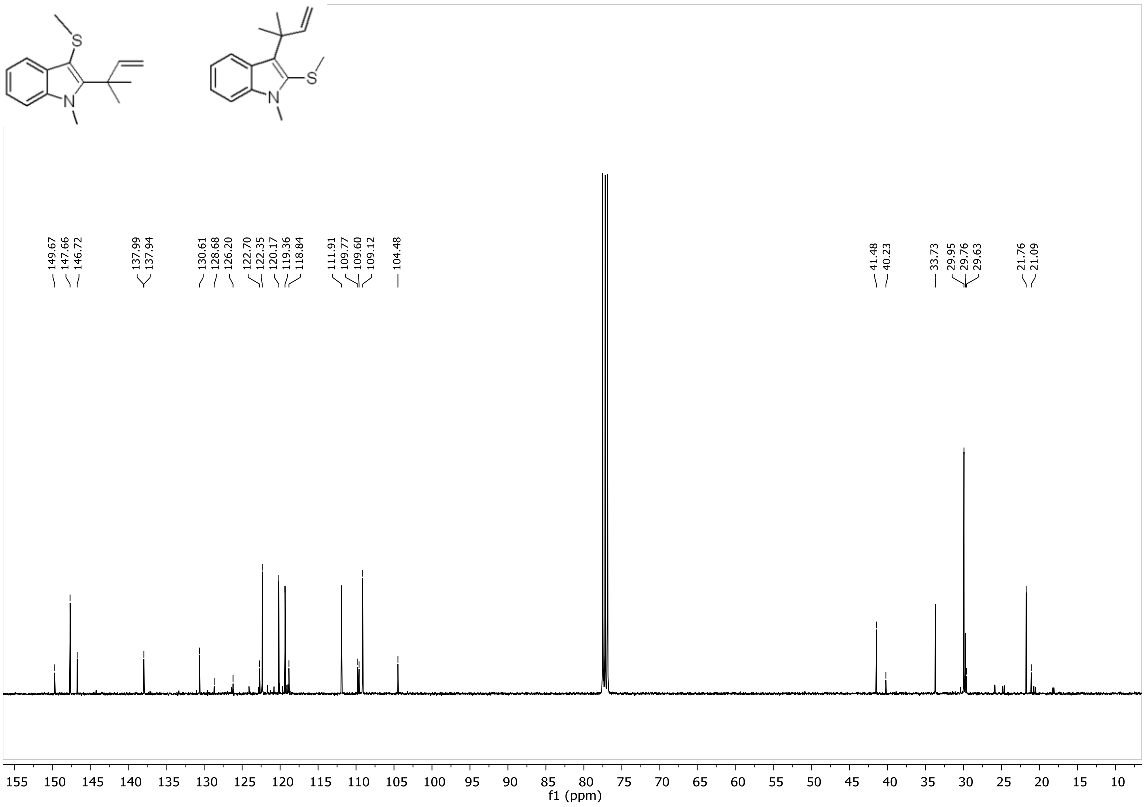

^{1}H-^{13}C HMBC

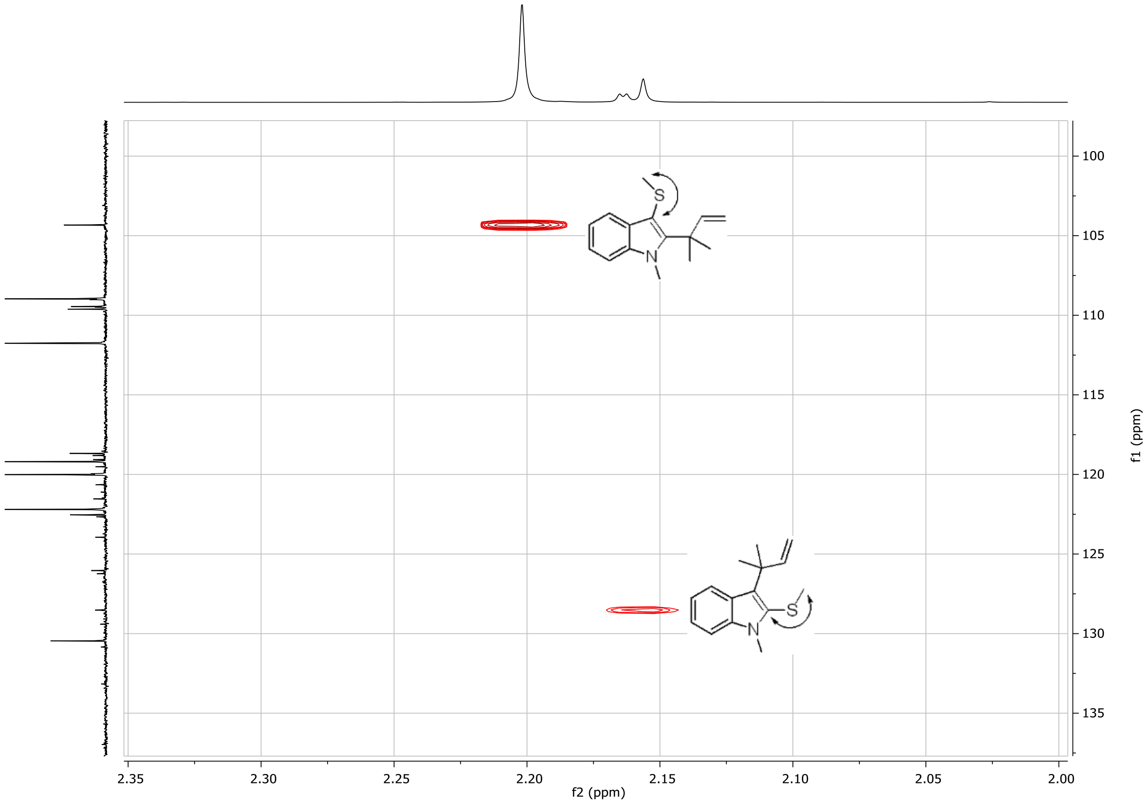

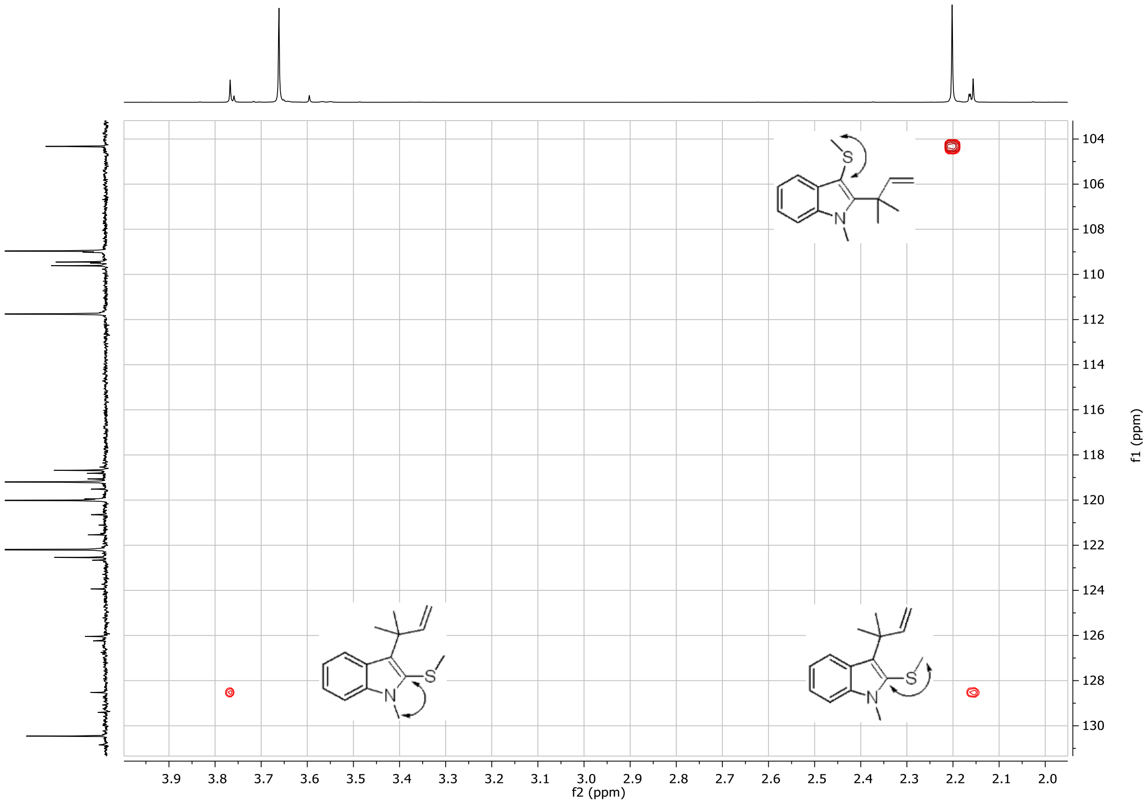

3ad

^{1}H NMR (400 MHz, Chloroform-*d*)

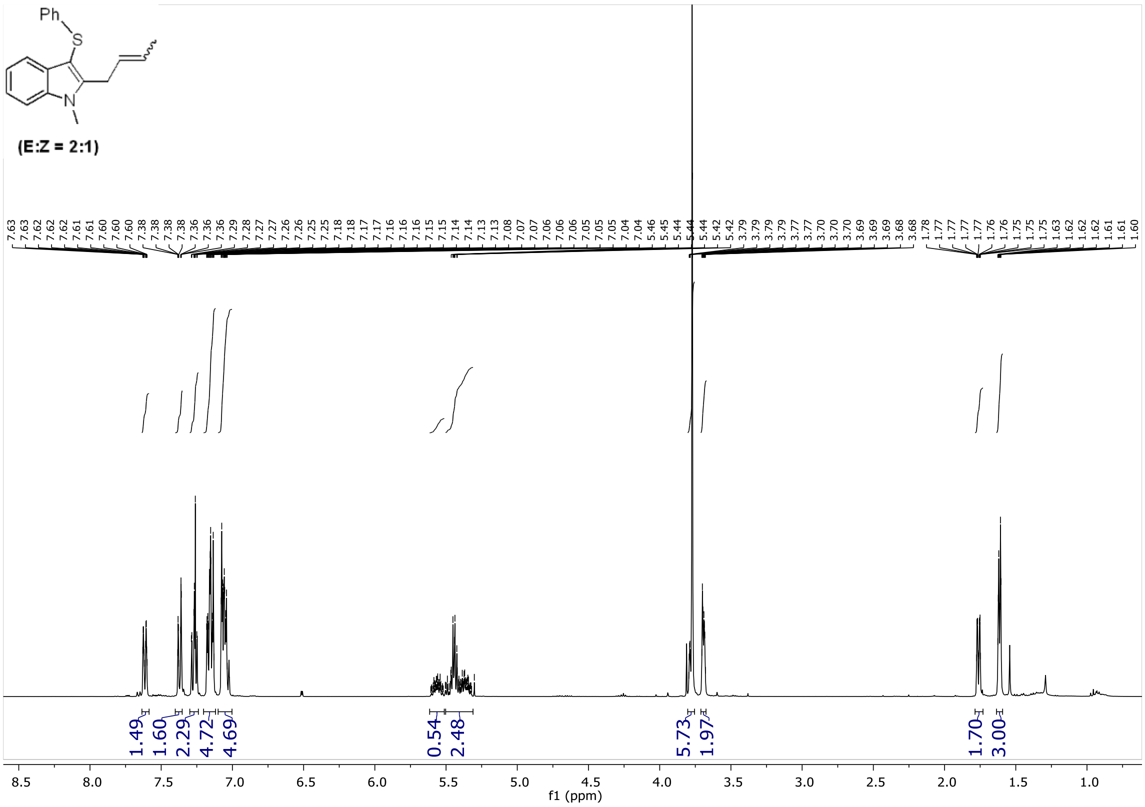

^{13}C NMR (101 MHz, Chloroform-*d*)

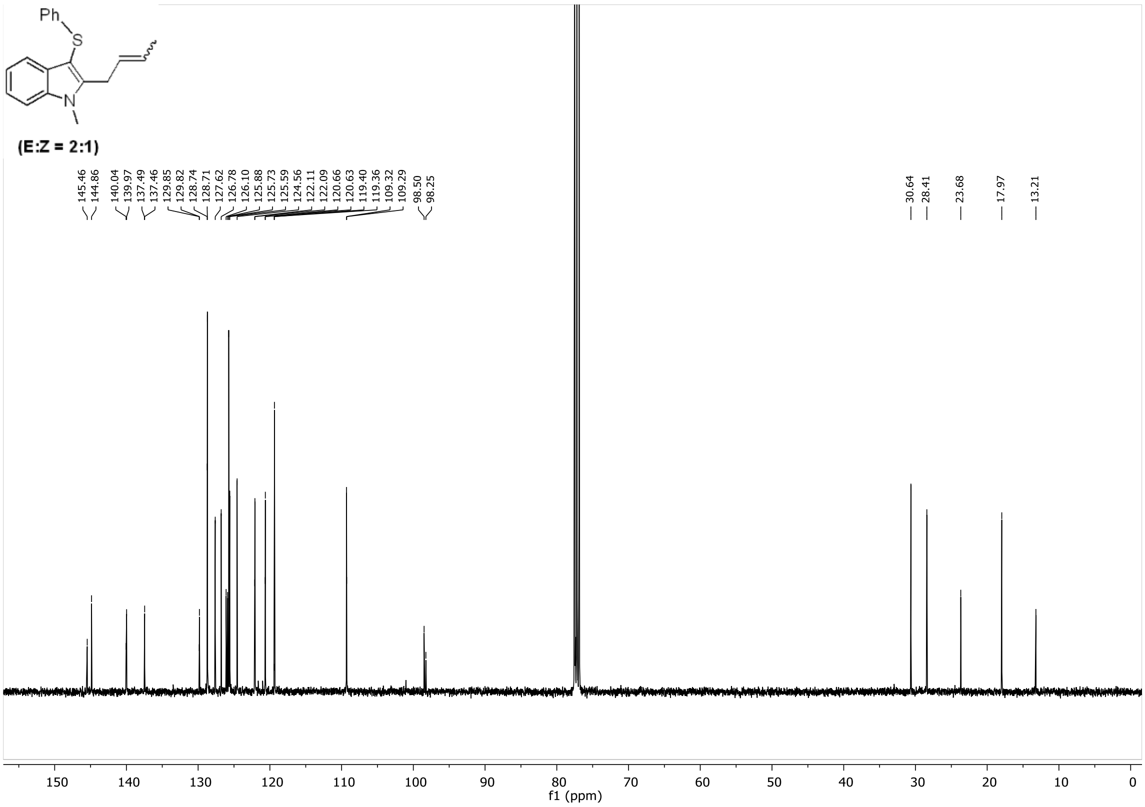

3ae

^{1}H NMR (400 MHz, Chloroform-*d*)

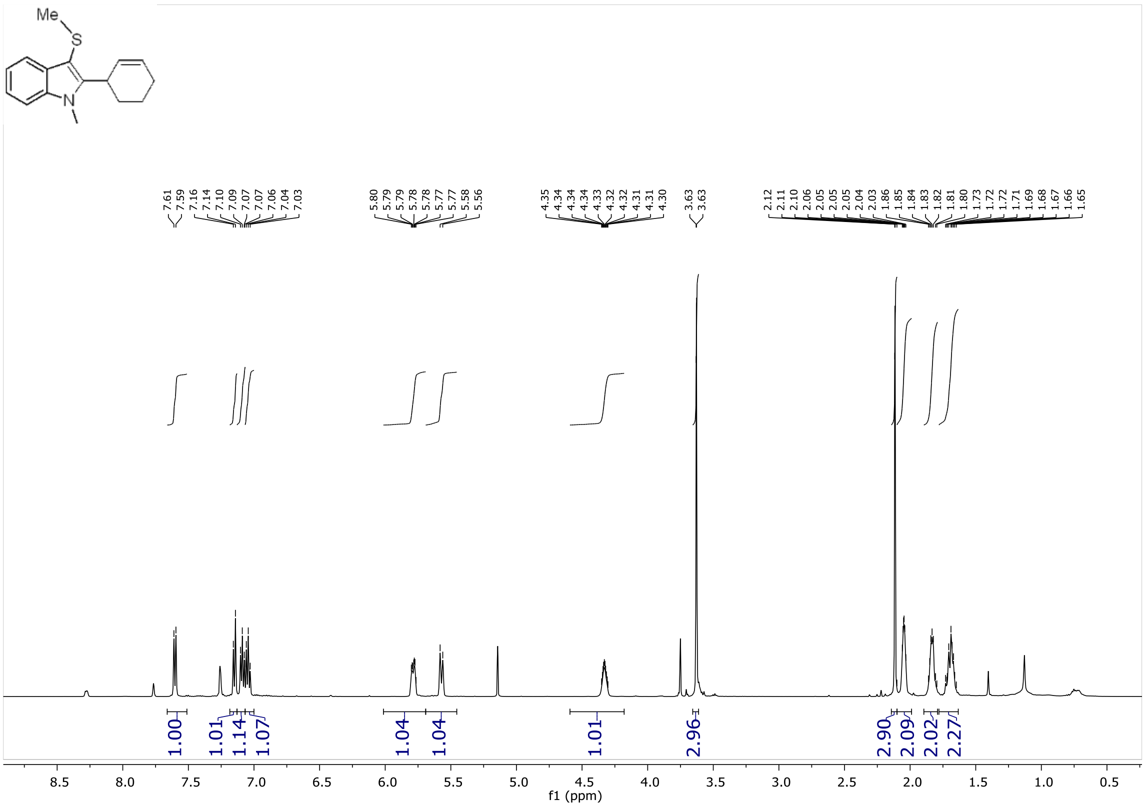

^{13}C NMR (126 MHz, Chloroform-*d*)

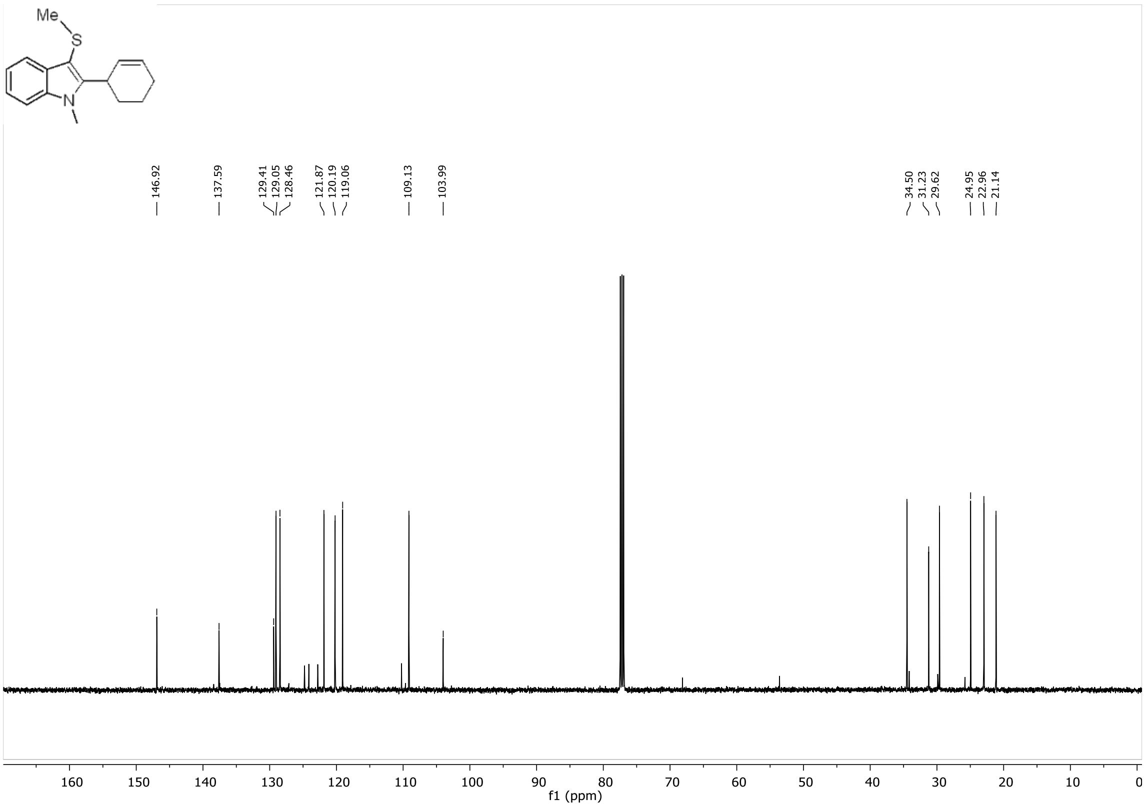

3af

^{1}H NMR (400 MHz, Chloroform-*d*)

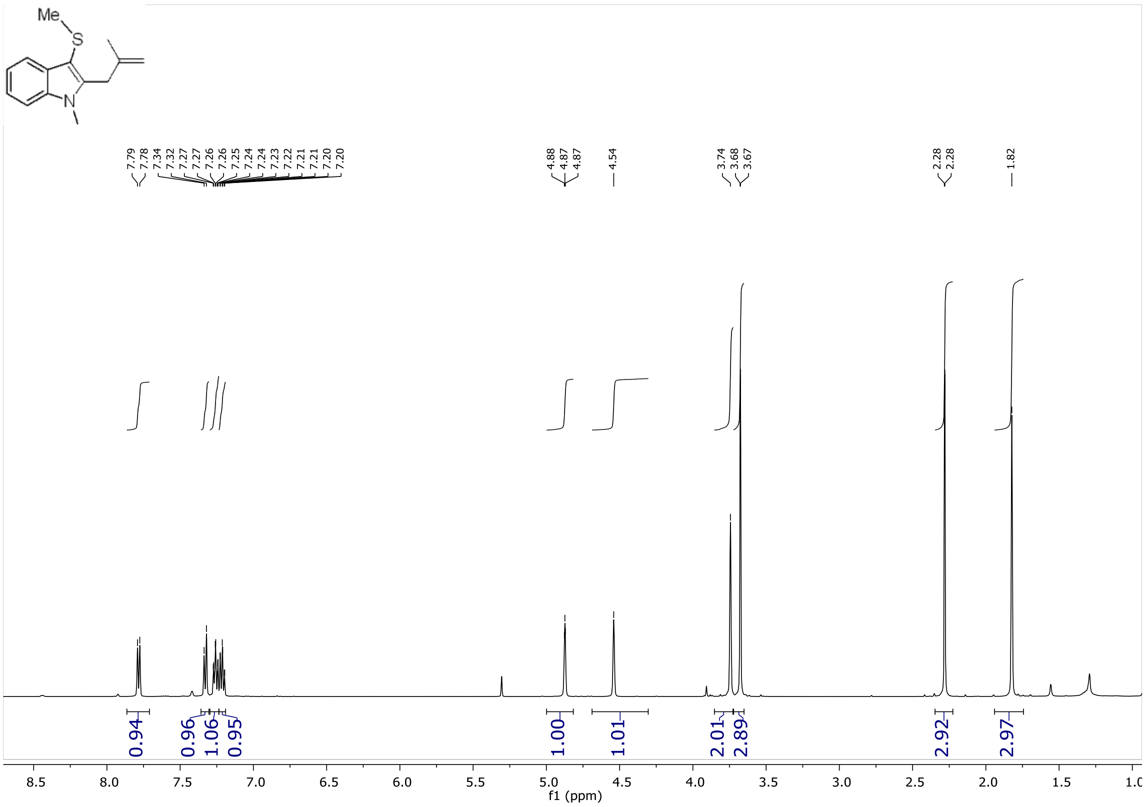

^{13}C NMR (126 MHz, Chloroform-*d*)

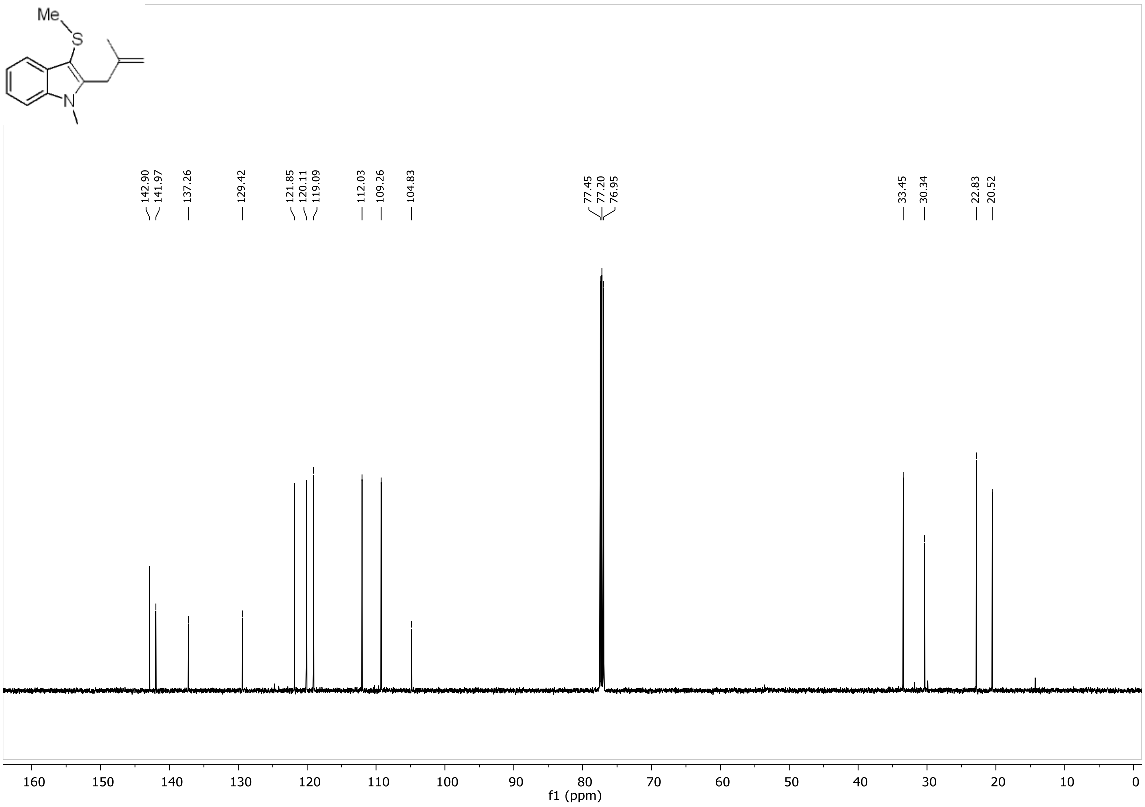

3ag

^{1}H NMR (400 MHz, Chloroform-*d*)

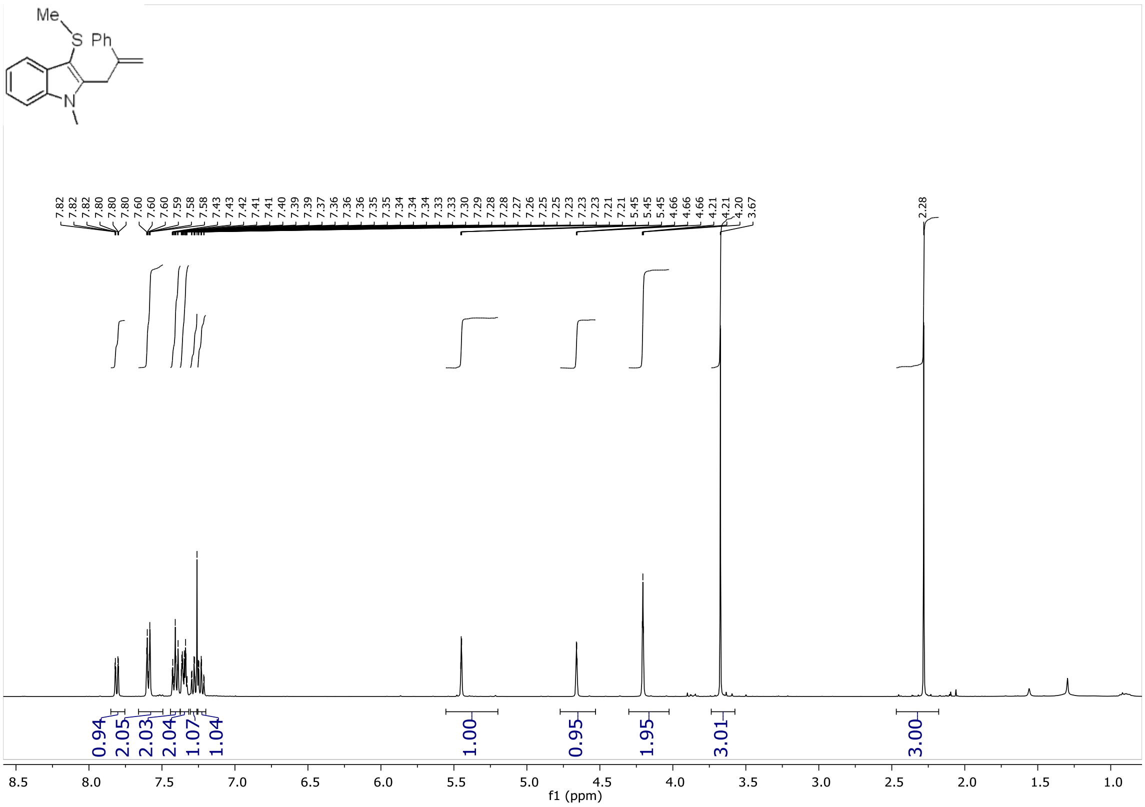

^{13}C NMR (101 MHz, Chloroform-*d*)

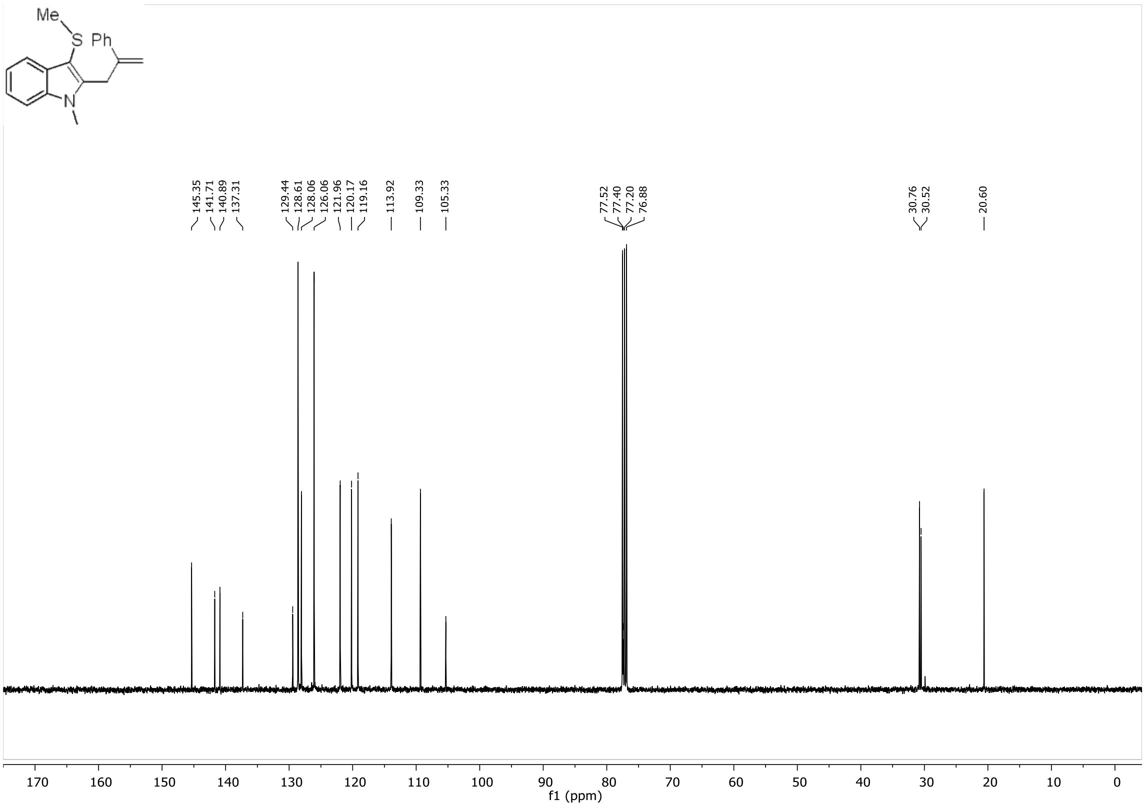

3ah

¹H NMR (400 MHz, Chloroform-*d*)

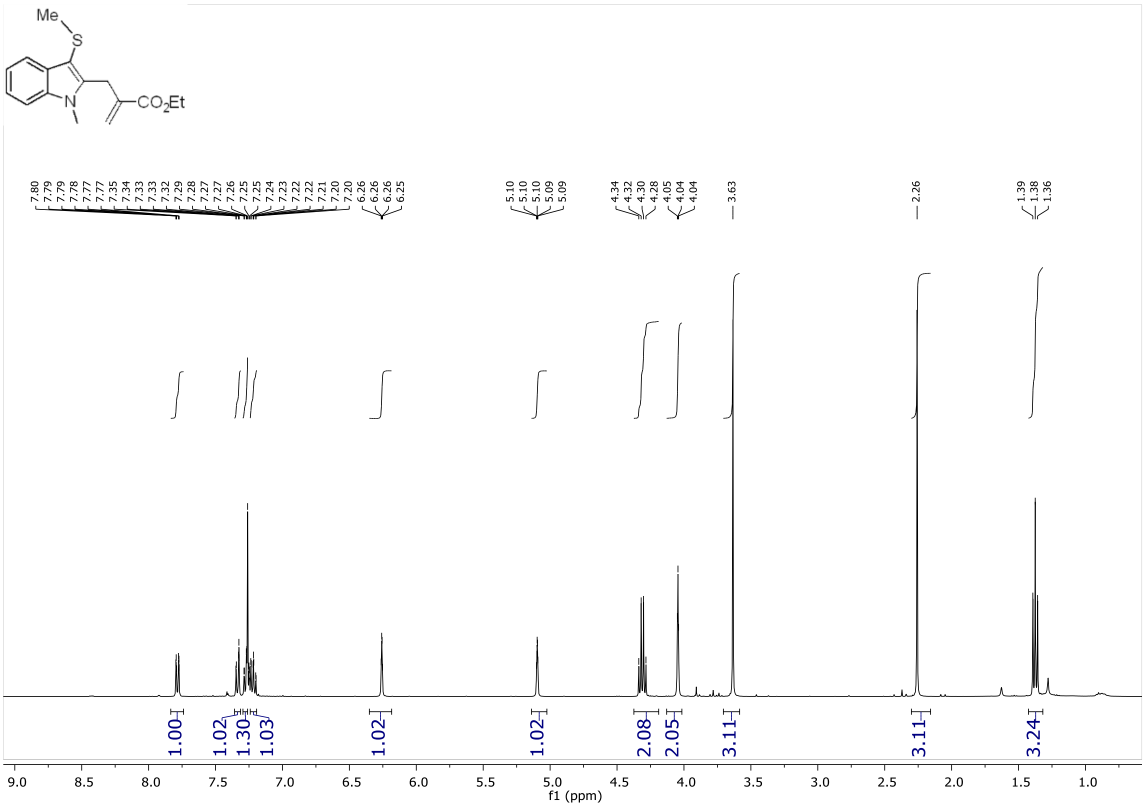

¹³C NMR (101 MHz, Chloroform-*d*)

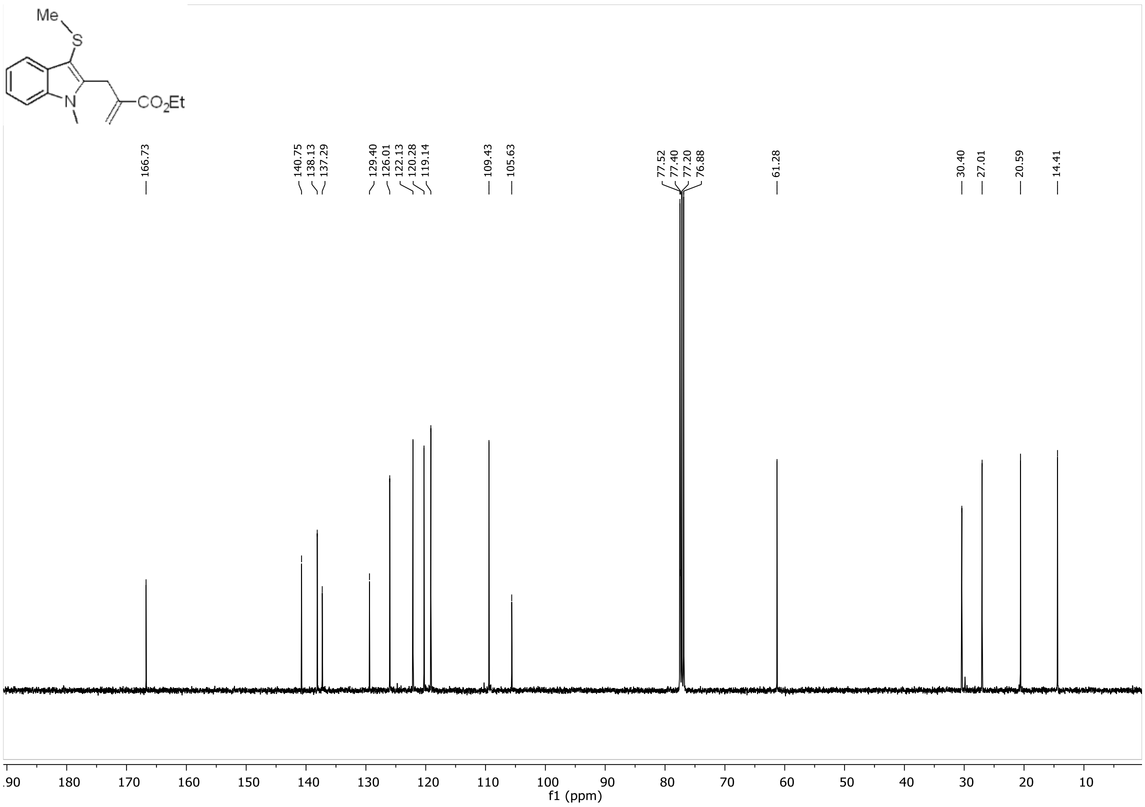

3ai

^{1}H NMR (400 MHz, Chloroform-*d*)

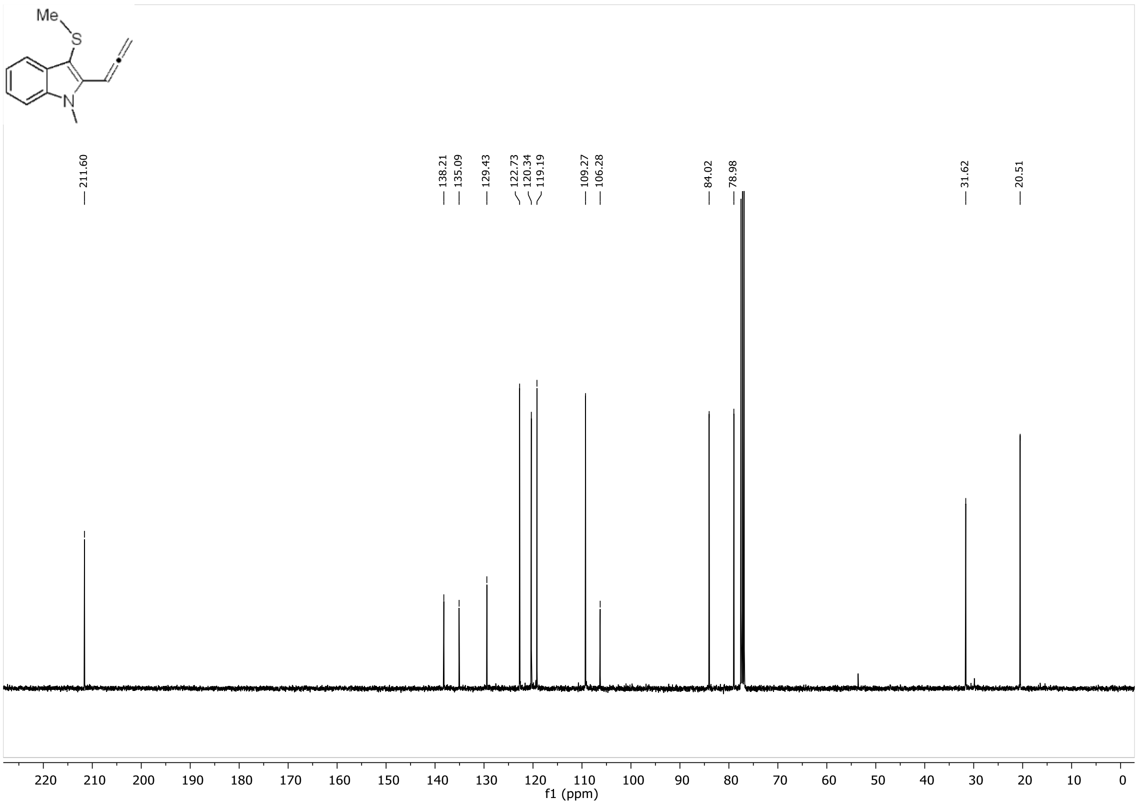

^{13}C NMR (101 MHz, Chloroform-*d*)

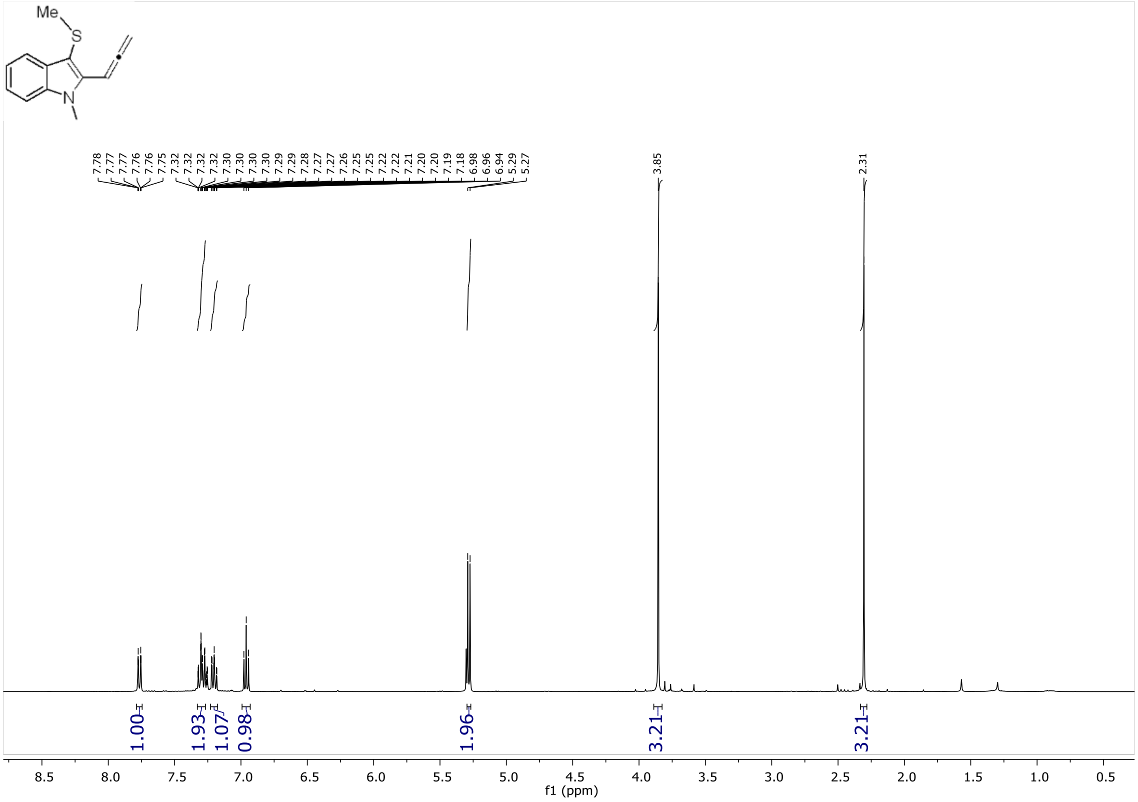

3aj

^{1}H NMR (400 MHz, Chloroform-*d*)

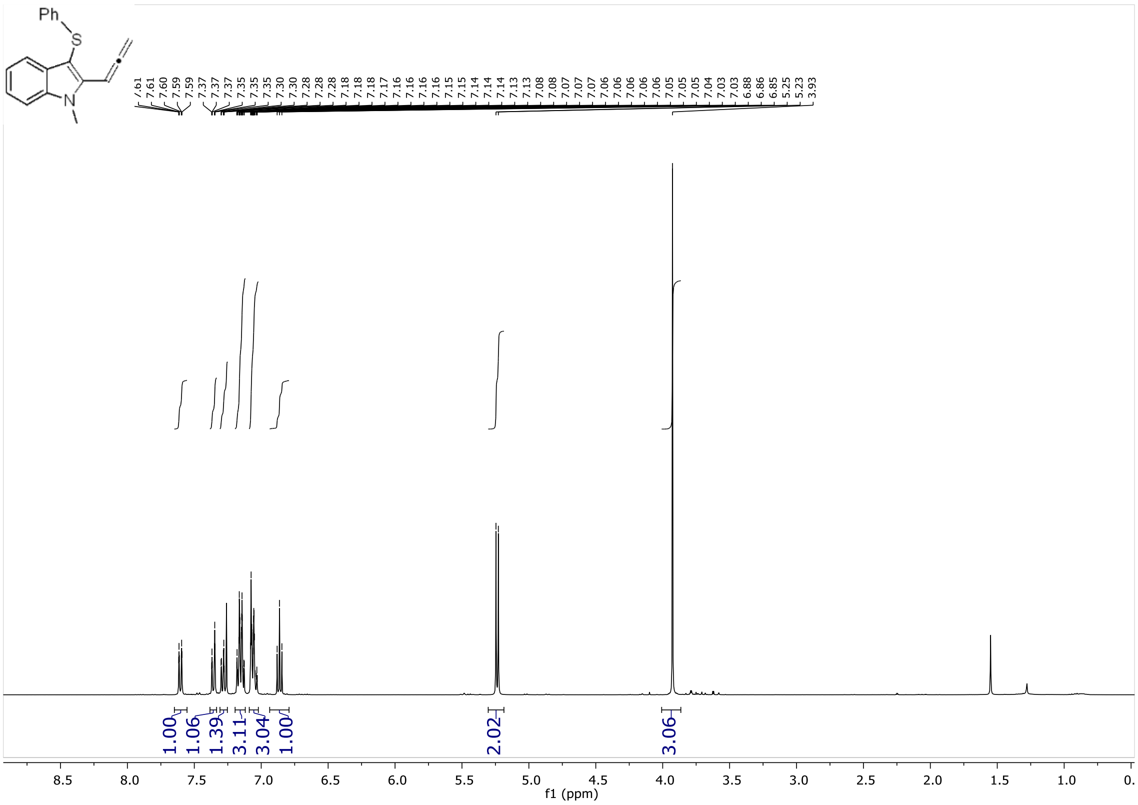

^{13}C NMR (101 MHz, Chloroform-*d*)

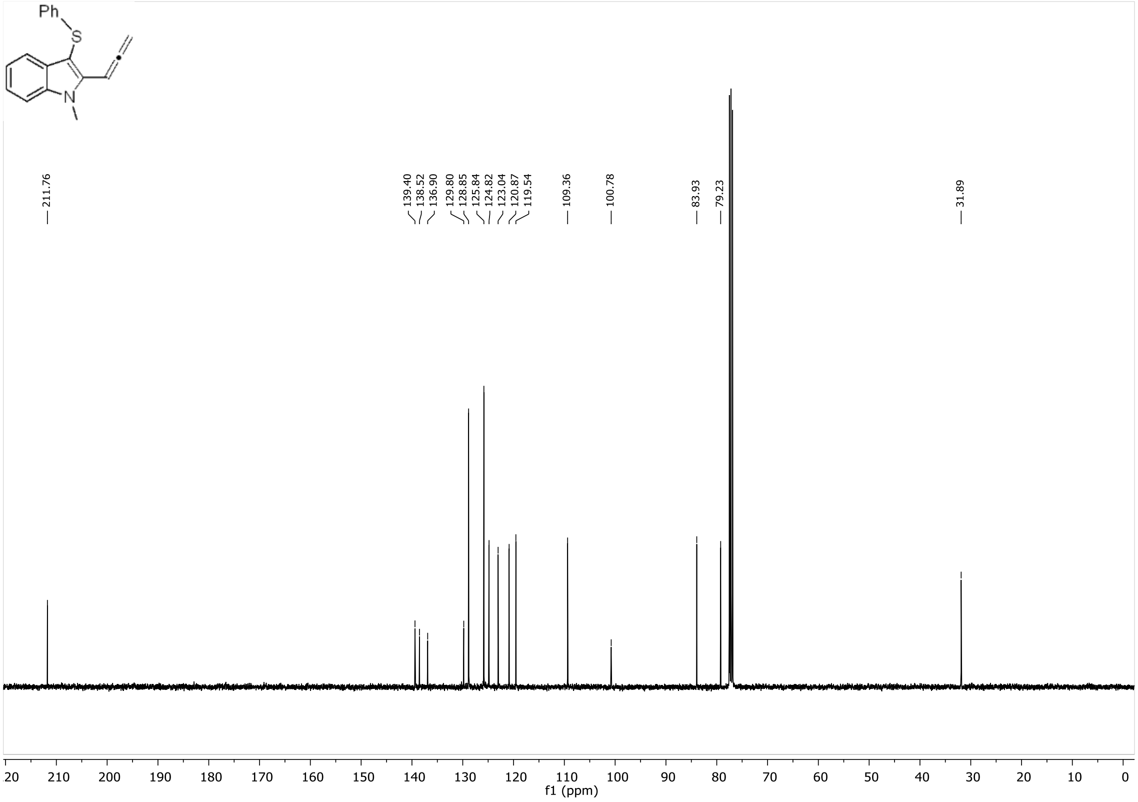

3ak

^{1}H NMR (400 MHz, Chloroform-*d*)

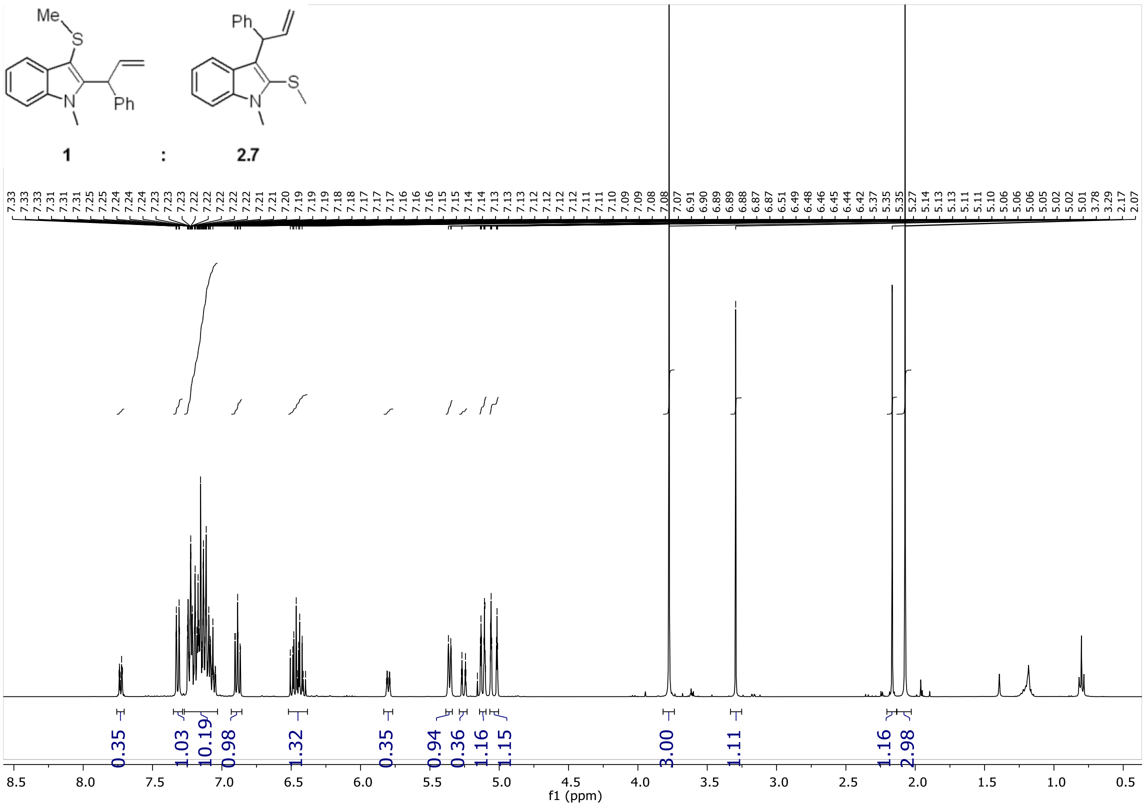

^{13}C NMR (101 MHz, Chloroform-*d*)

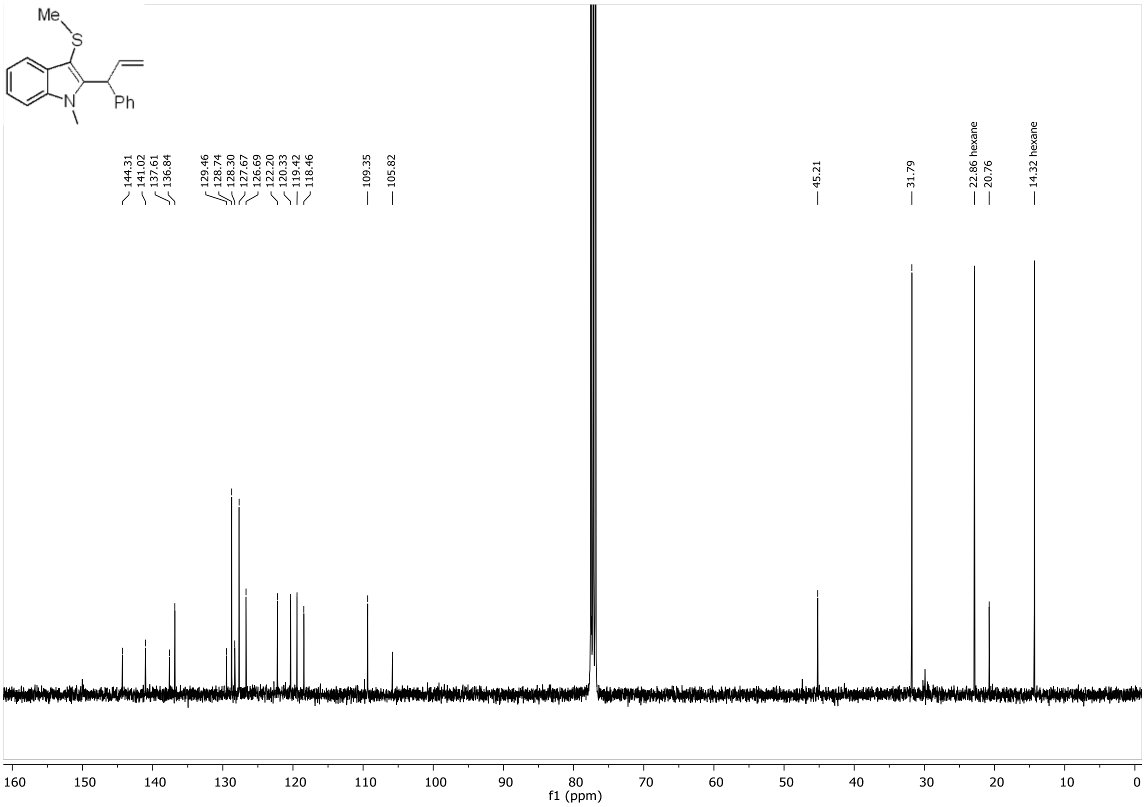

^{1}H-^{13}C NMR HMBC

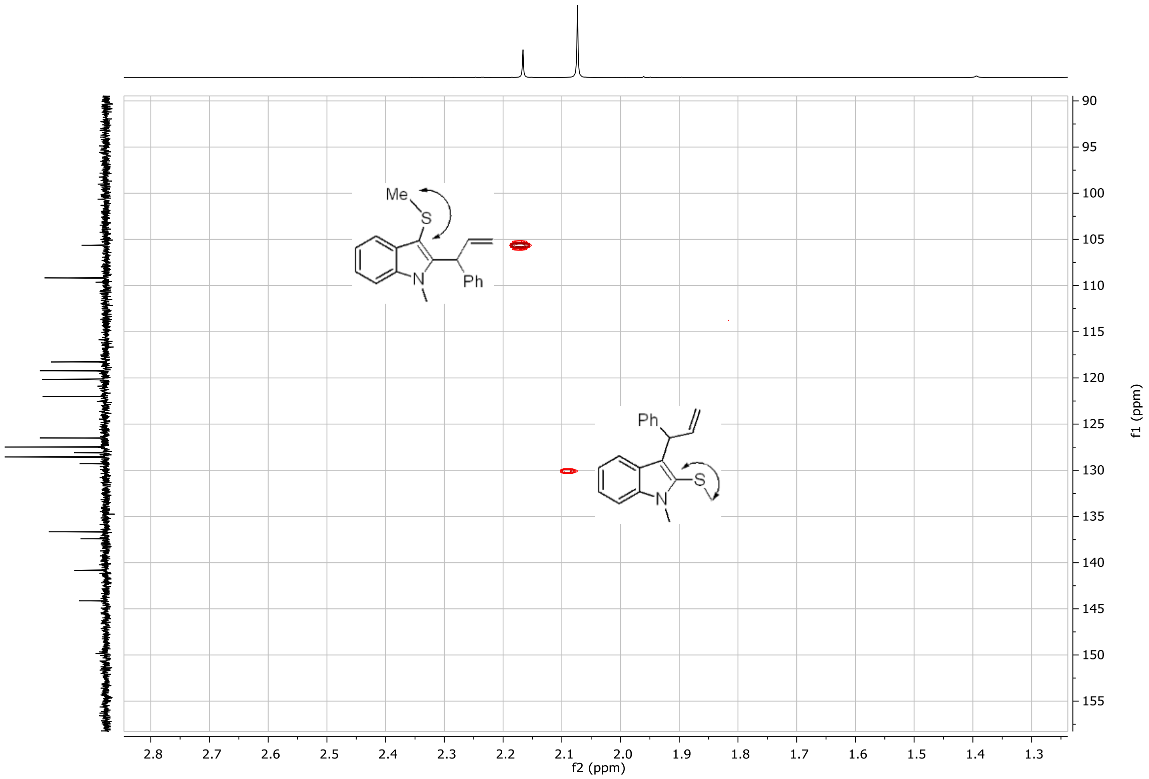

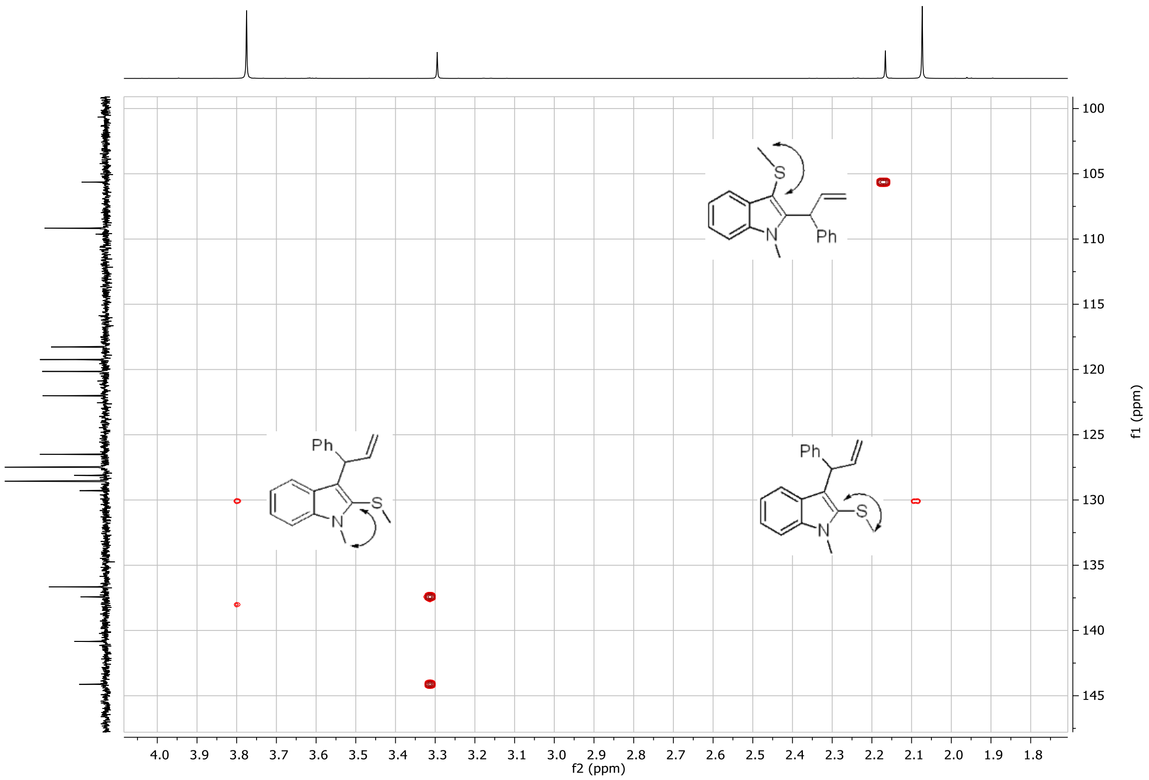

4ak

^{1}H NMR (400 MHz, Chloroform-*d*)

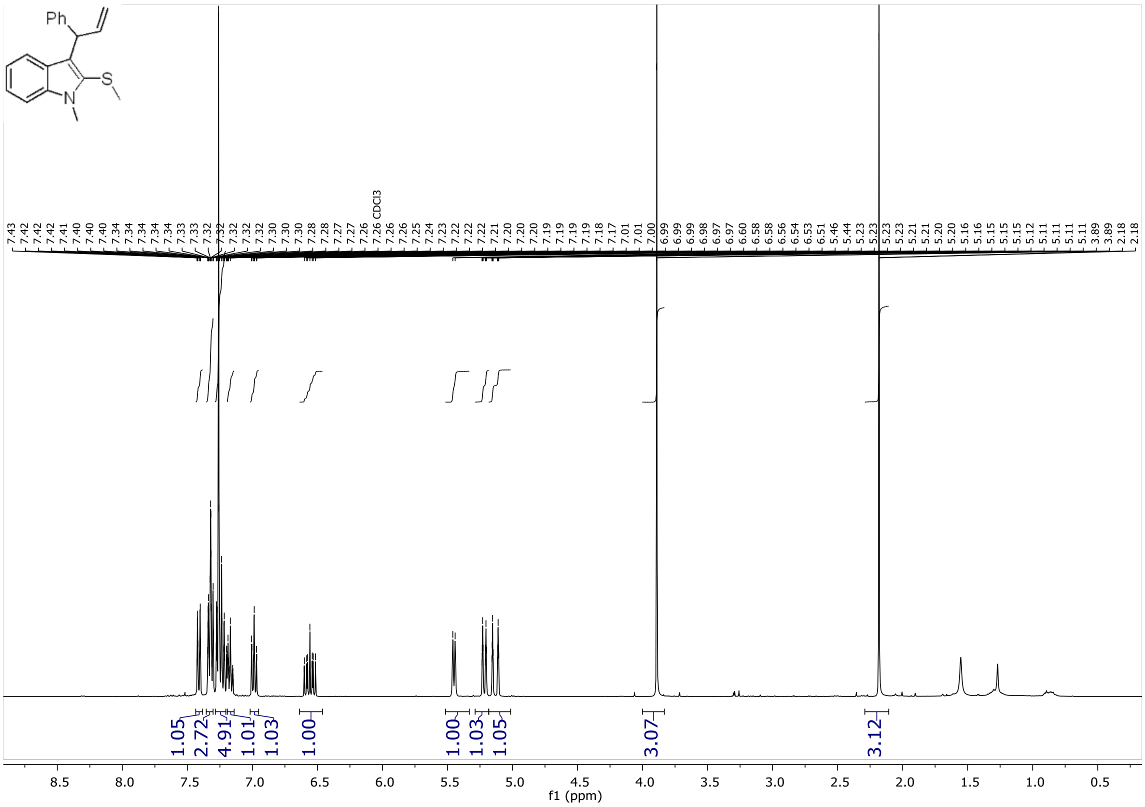

^{13}C NMR (101 MHz, Chloroform-*d*)

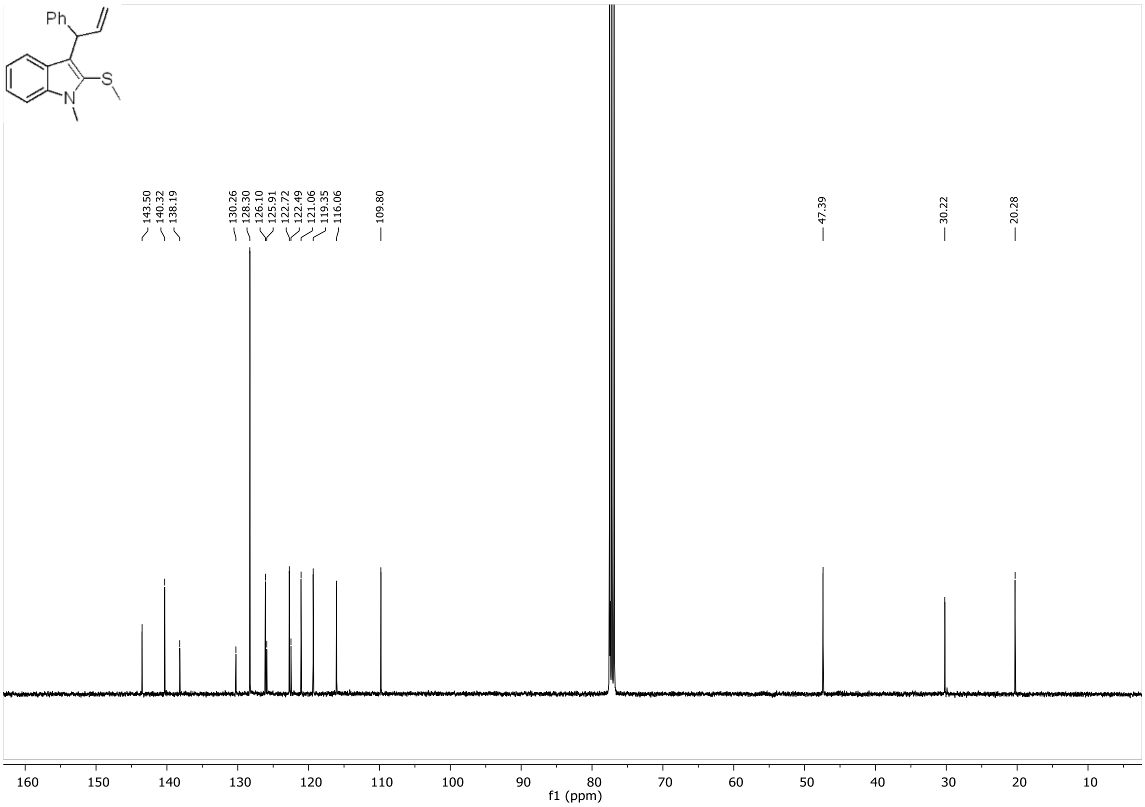

^{1}H-^{13}C NMR HMBC

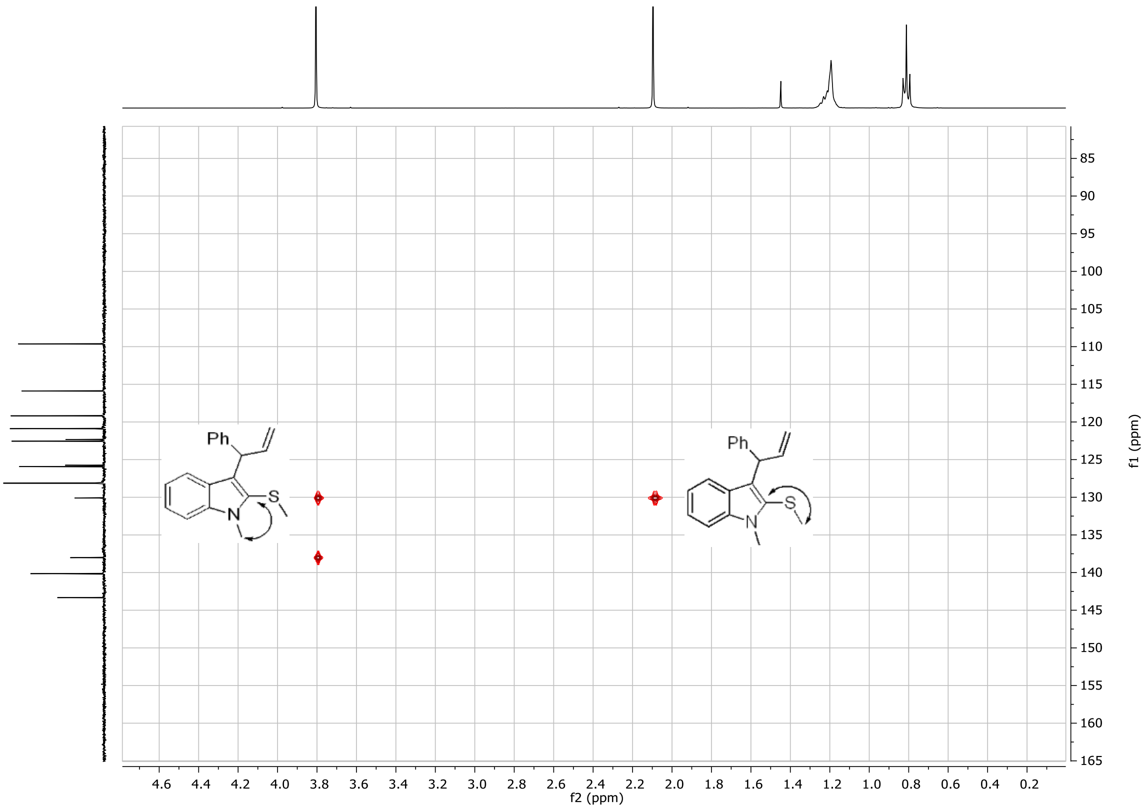

4al

¹H NMR (400 MHz, Chloroform-*d*)

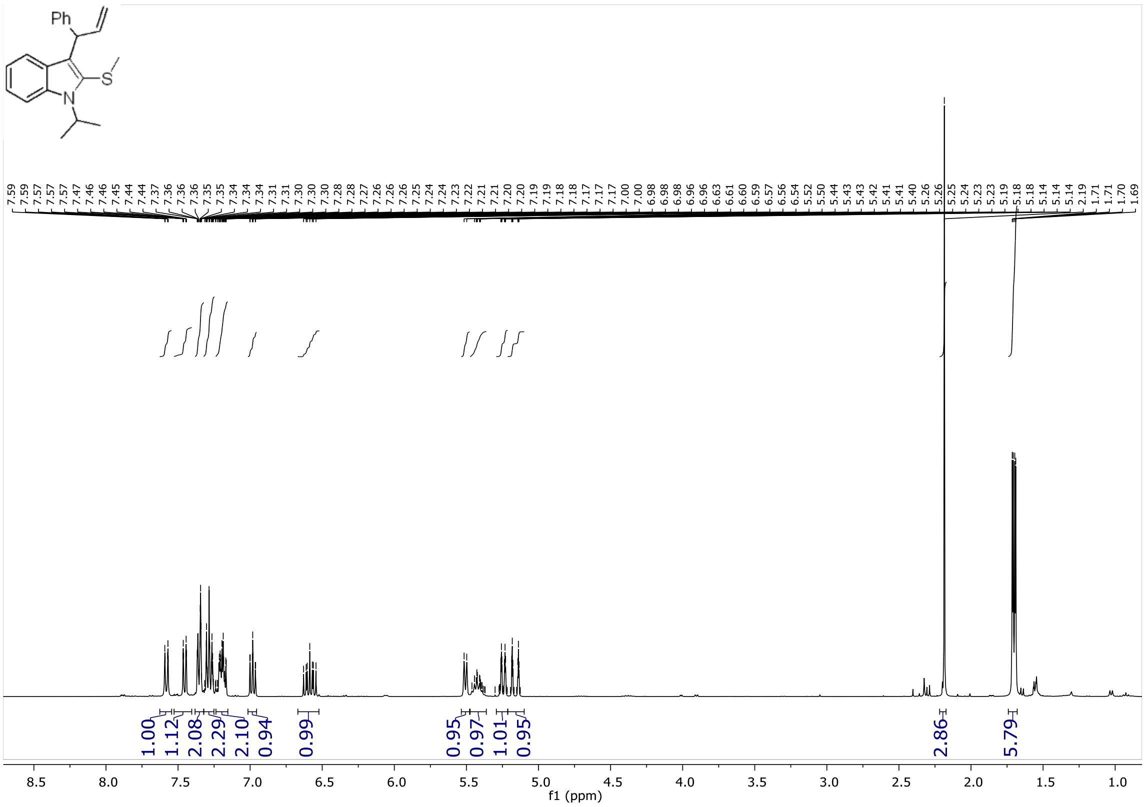

¹³C NMR (101 MHz, Chloroform-*d*)

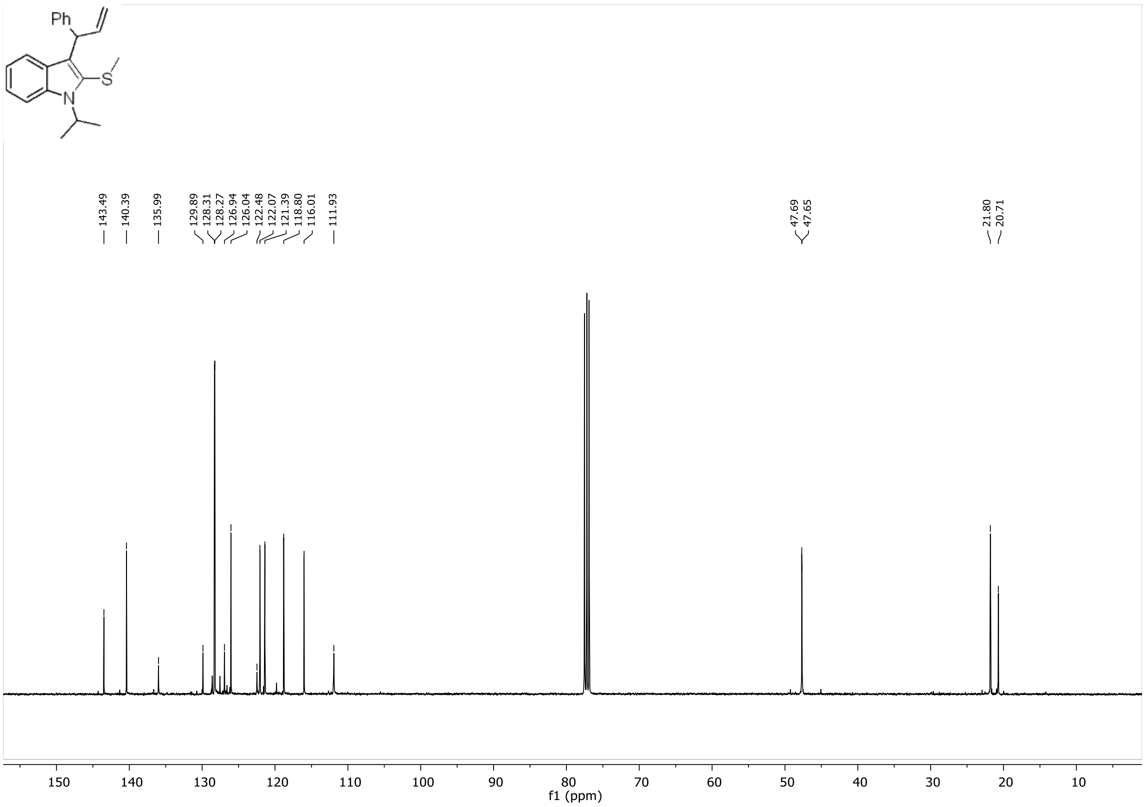

^1H-^{13}C NMR HMBC

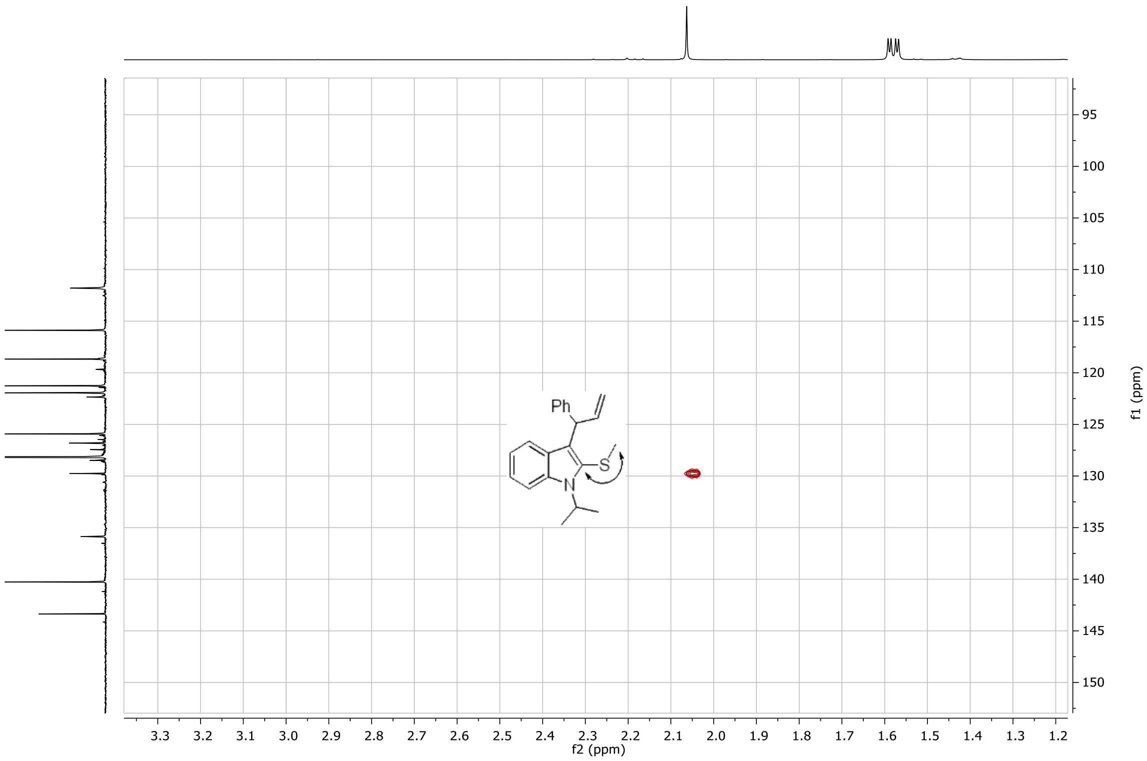

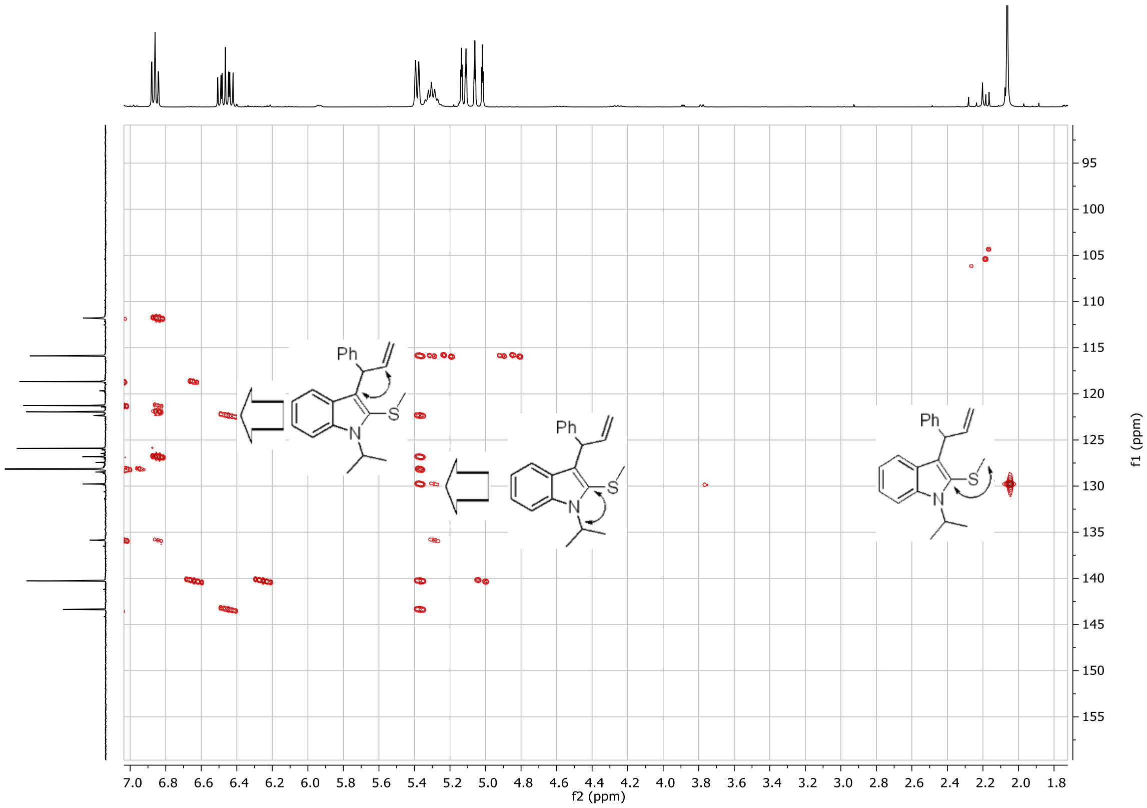

3am

^{1}H NMR (400 MHz, Chloroform-*d*)

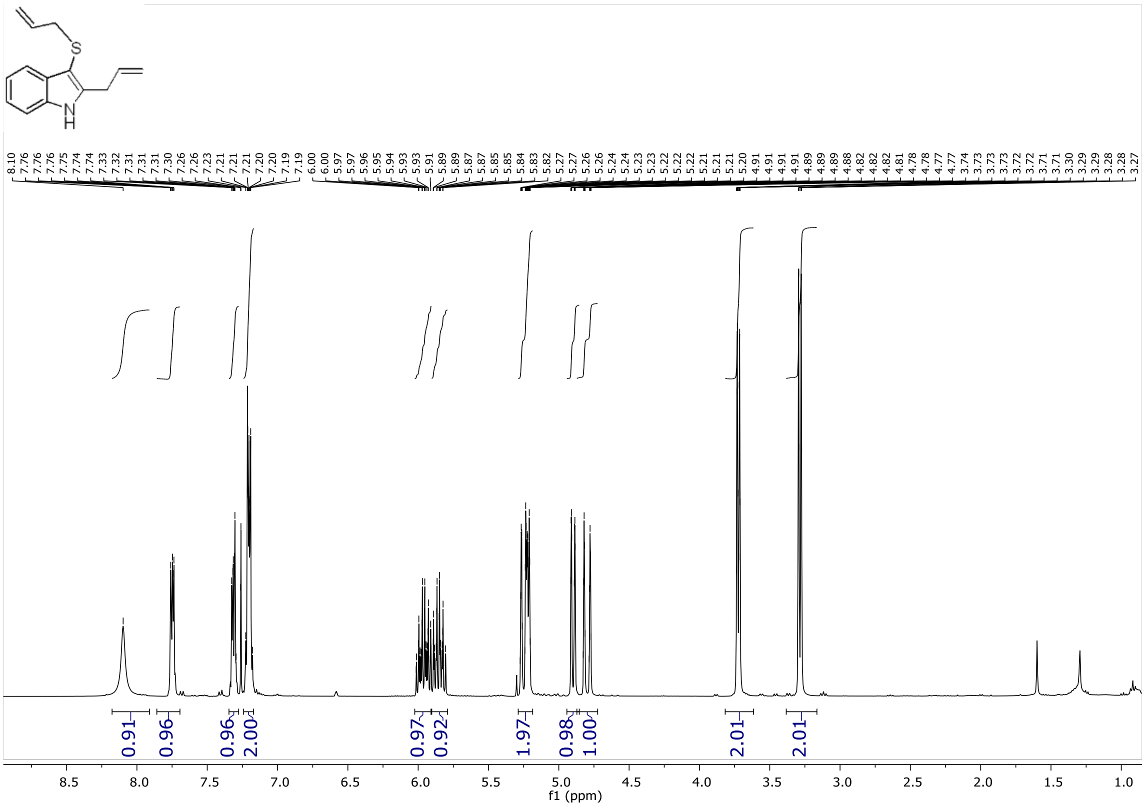

^{13}C NMR (101 MHz, Chloroform-*d*)

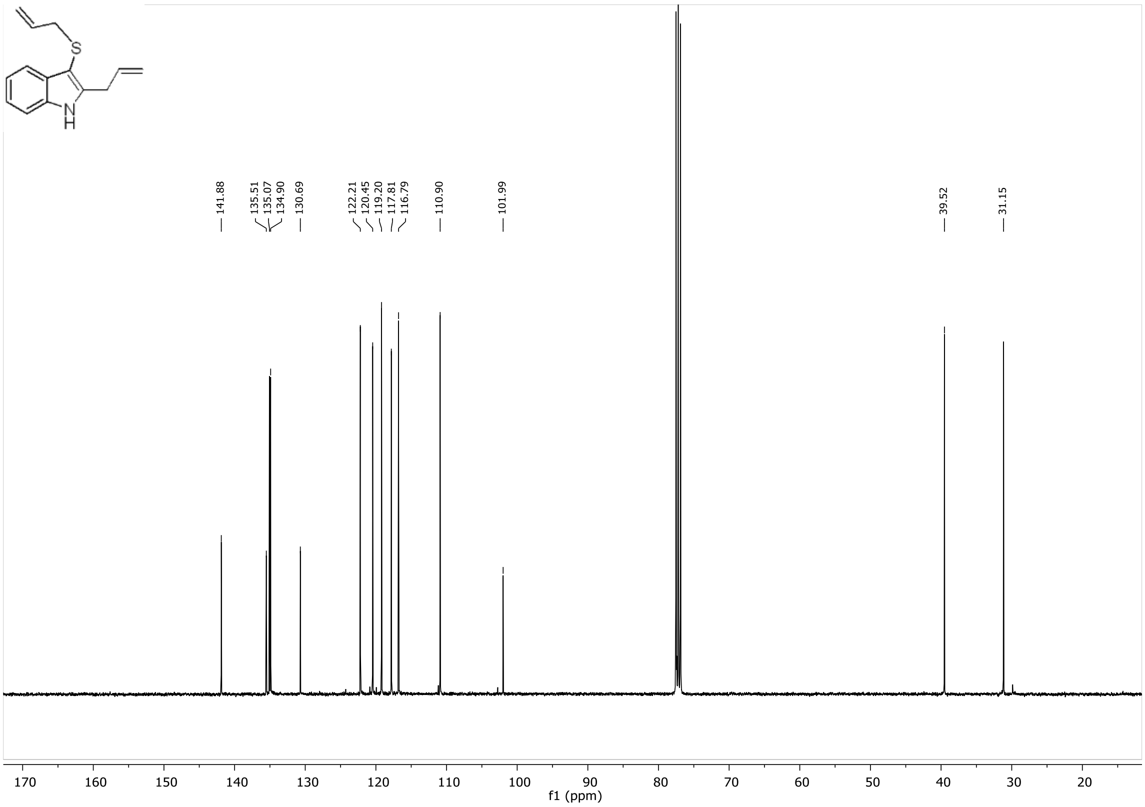

2d

^{1}H NMR (400 MHz, Chloroform-*d*)

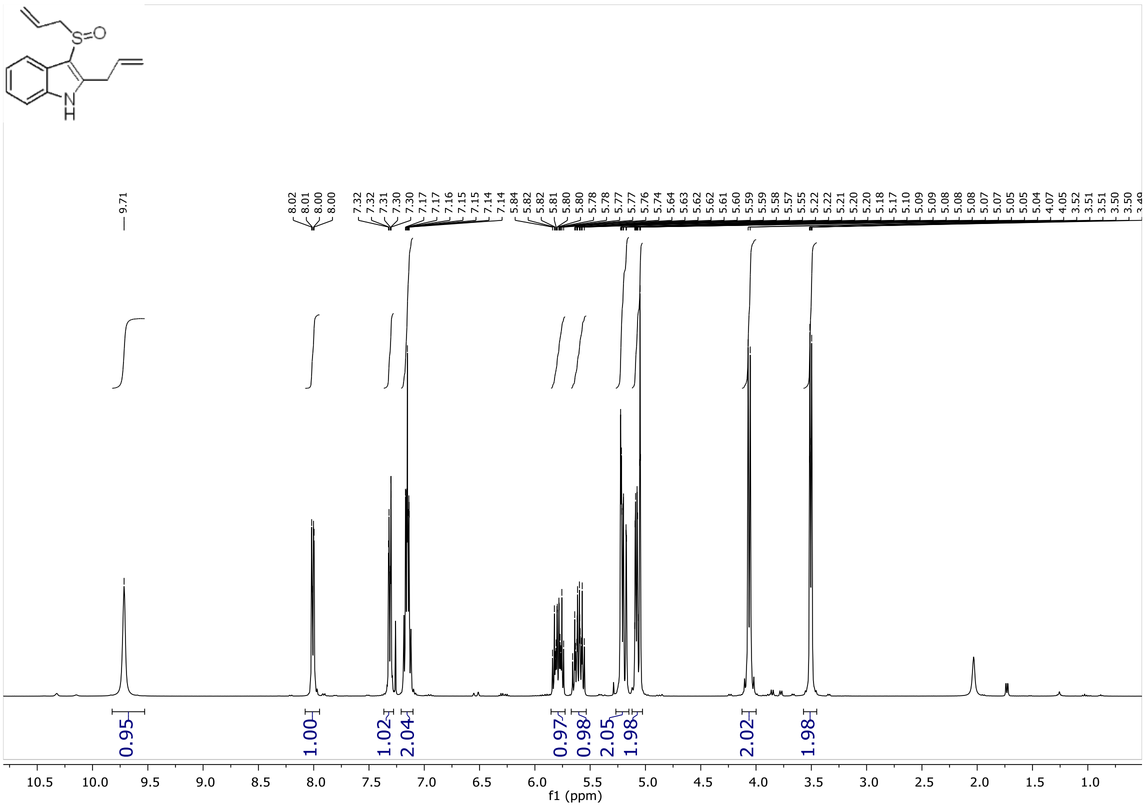

^{13}C NMR (101 MHz, Chloroform-*d*)

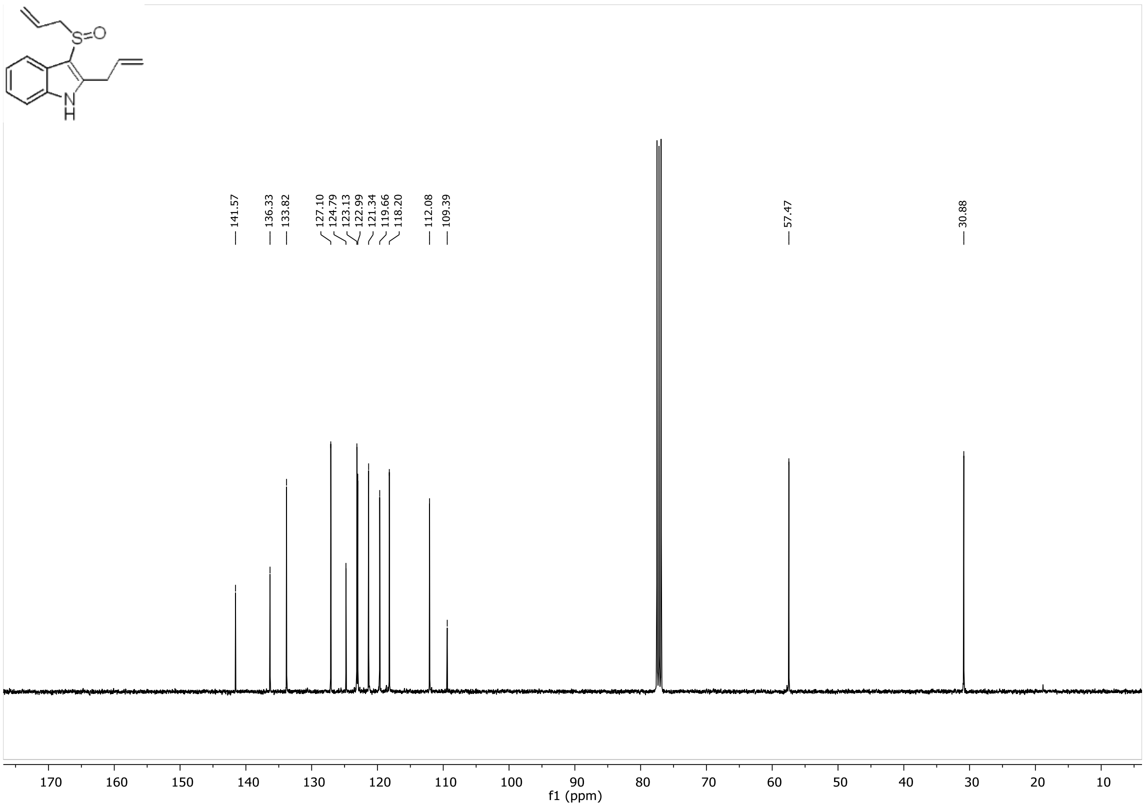

2e

^{1}H NMR (400 MHz, Chloroform-*d*)

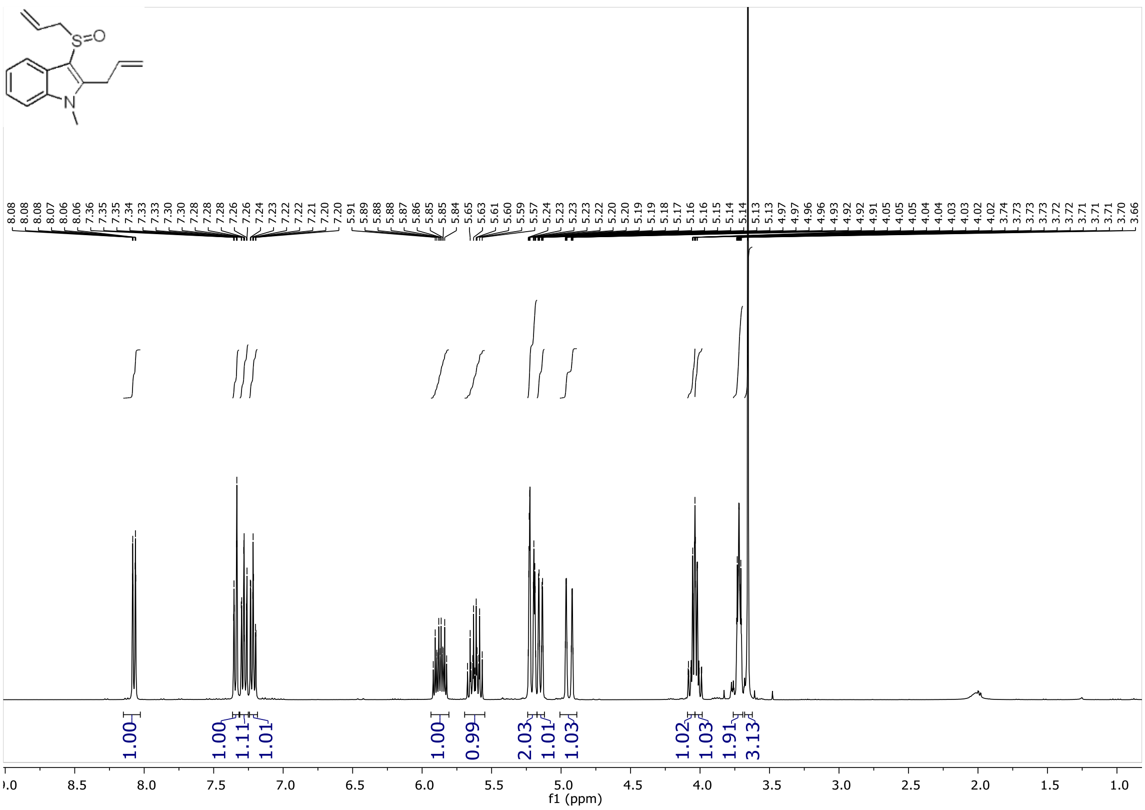

^{13}C NMR (101 MHz, Chloroform-*d*)

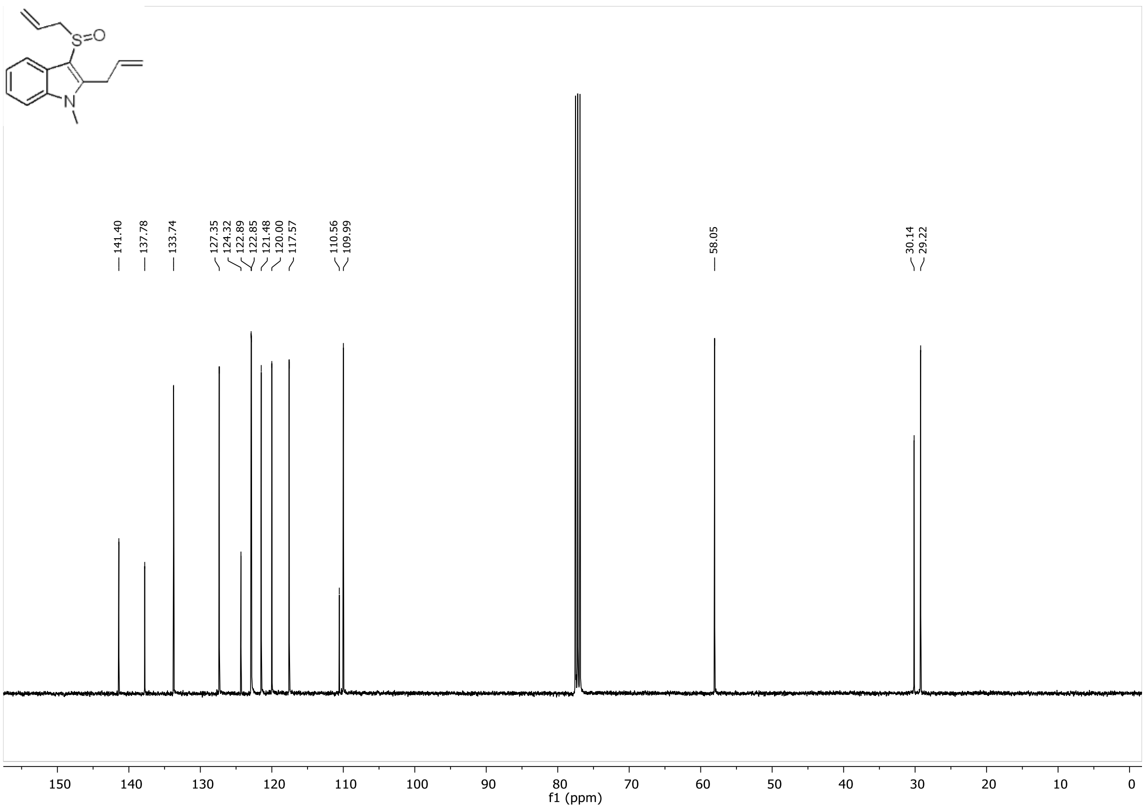

3an

¹H NMR (400 MHz, Chloroform-*d*)

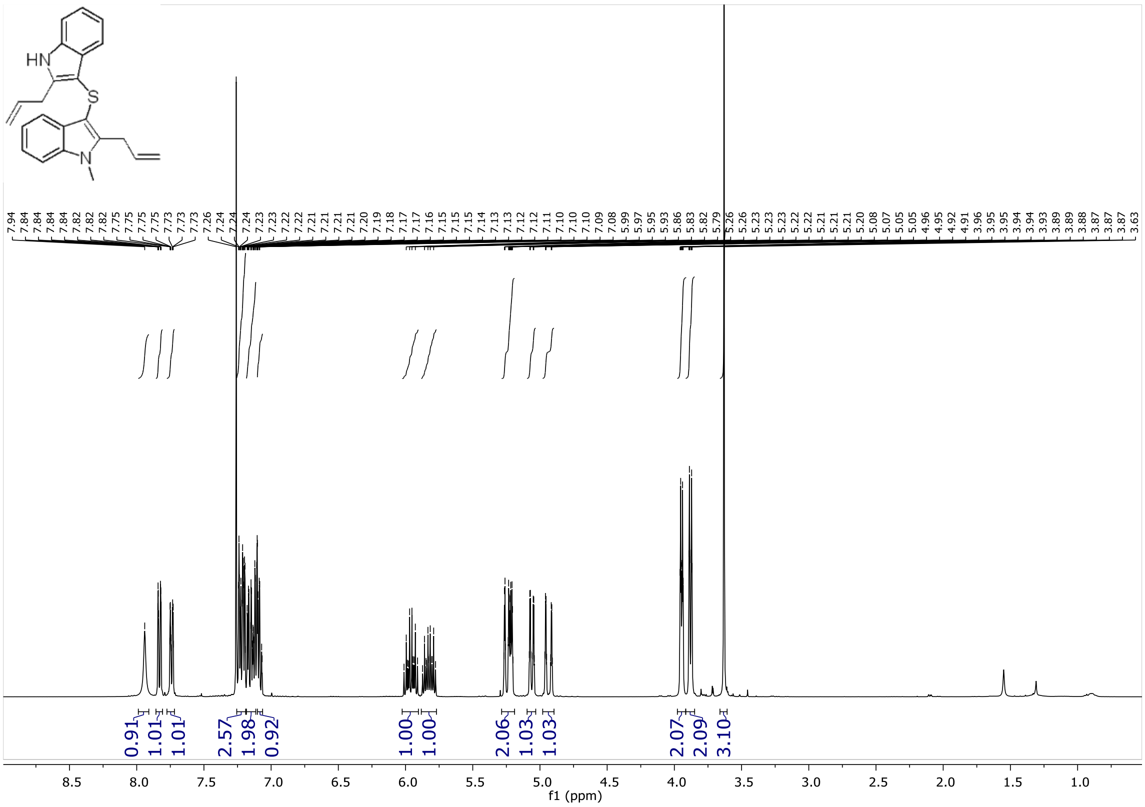

¹³C NMR (101 MHz, Chloroform-*d*)

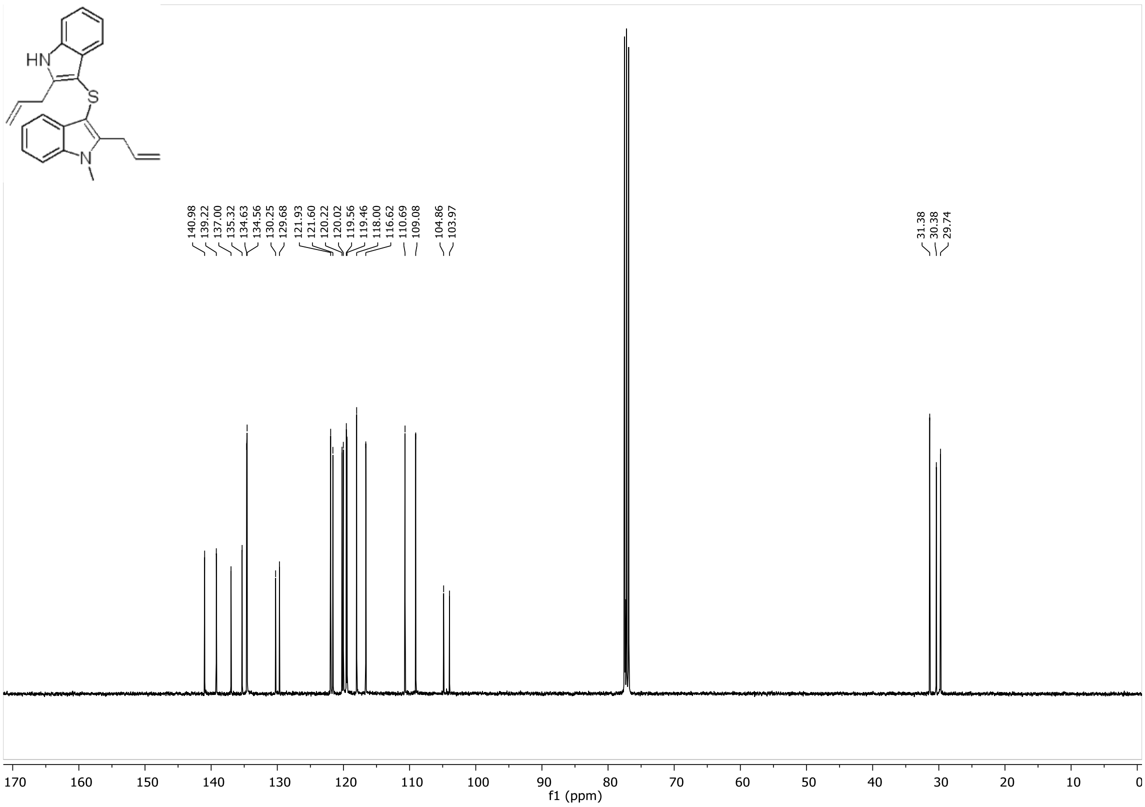

3ao

^{1}H NMR (400 MHz, Chloroform-*d*)

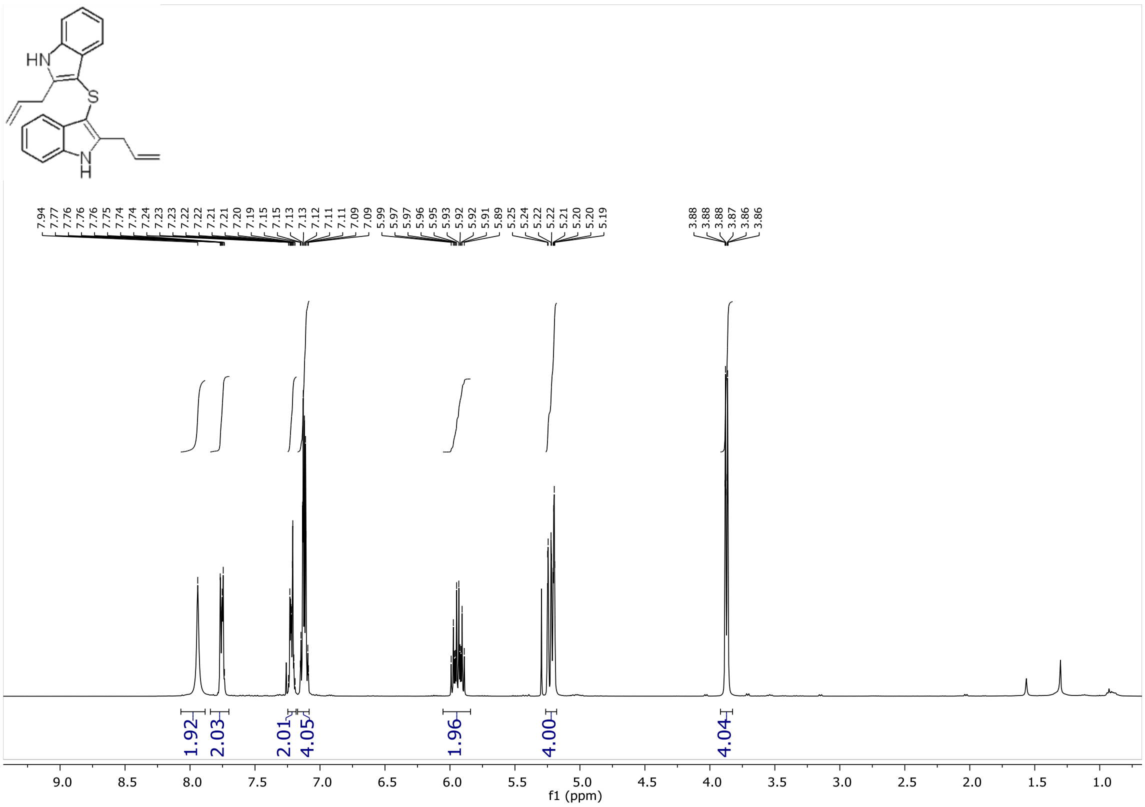

^{13}C NMR (101 MHz, Chloroform-*d*)

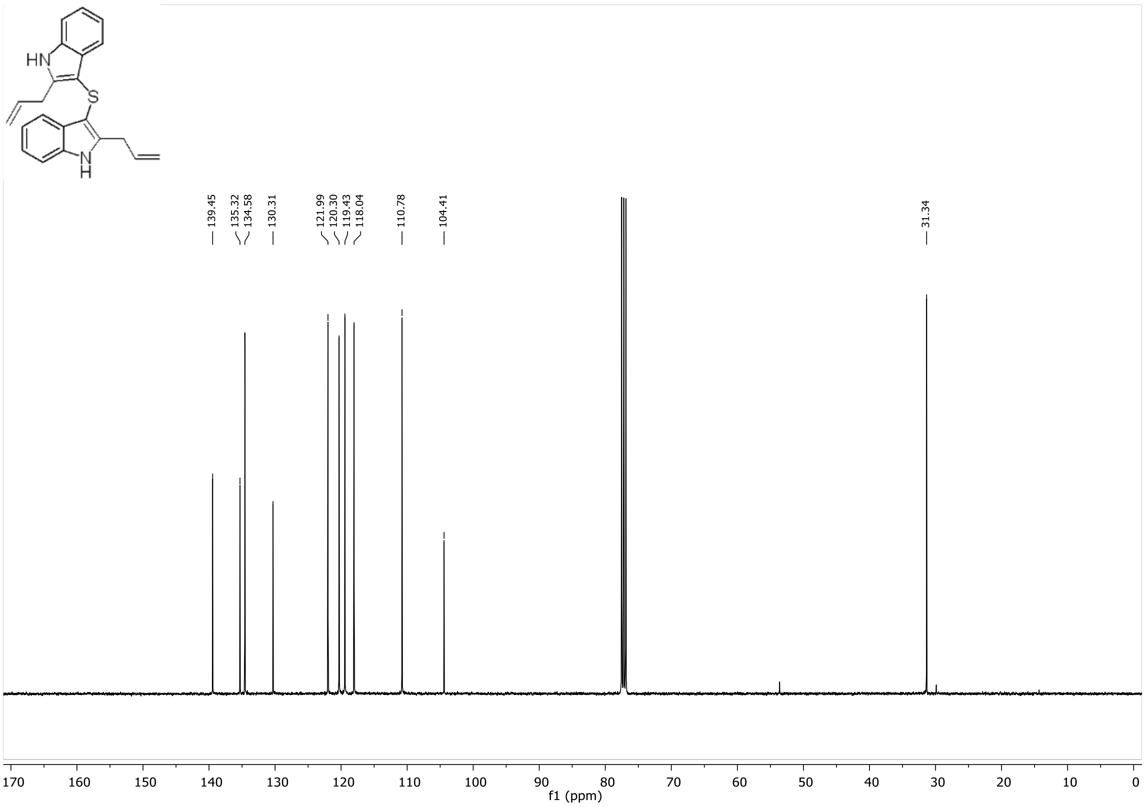

3ap

^{1}H NMR (400 MHz, Chloroform-*d*)

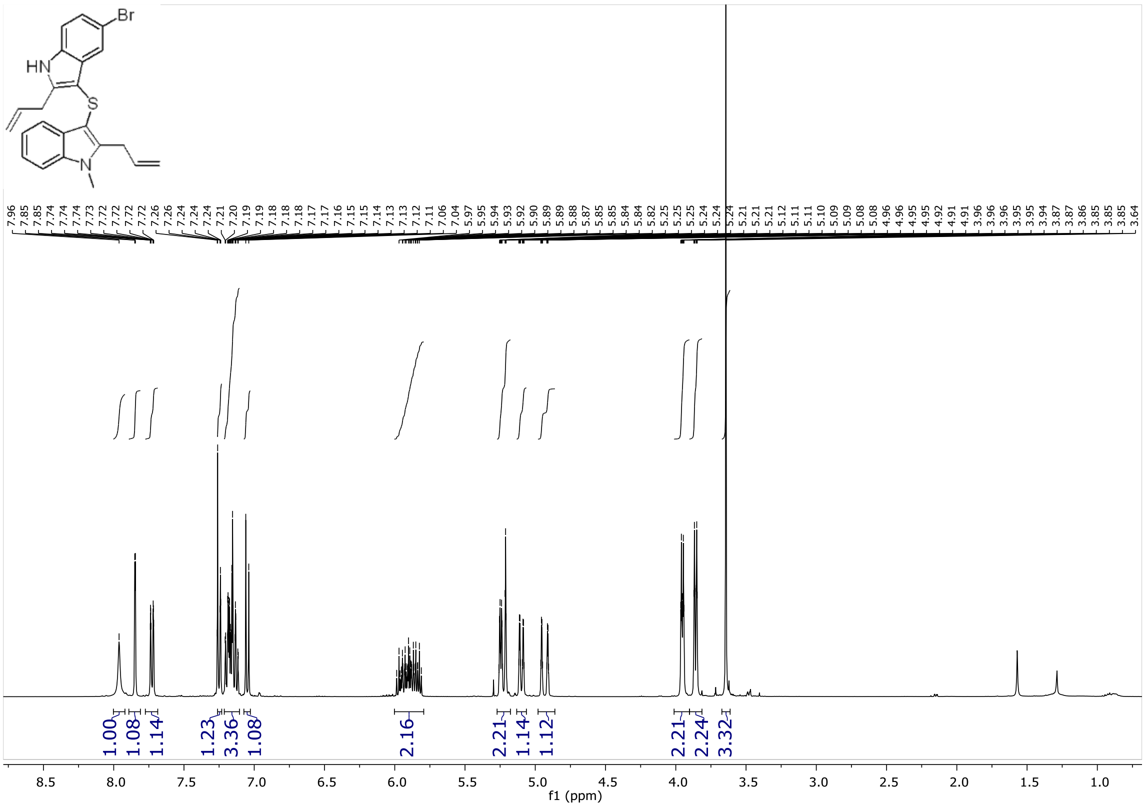

^{13}C NMR (101 MHz, Chloroform-*d*)

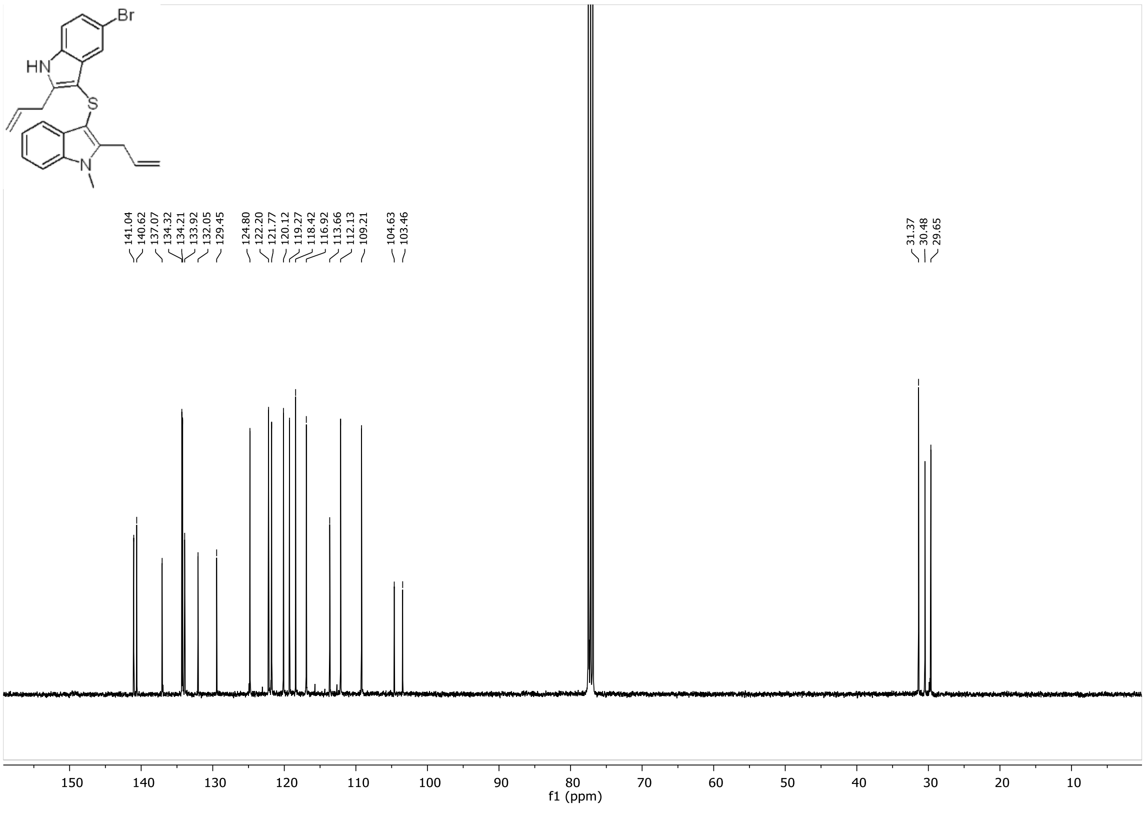

6a

¹H NMR (400 MHz, Chloroform-*d*)

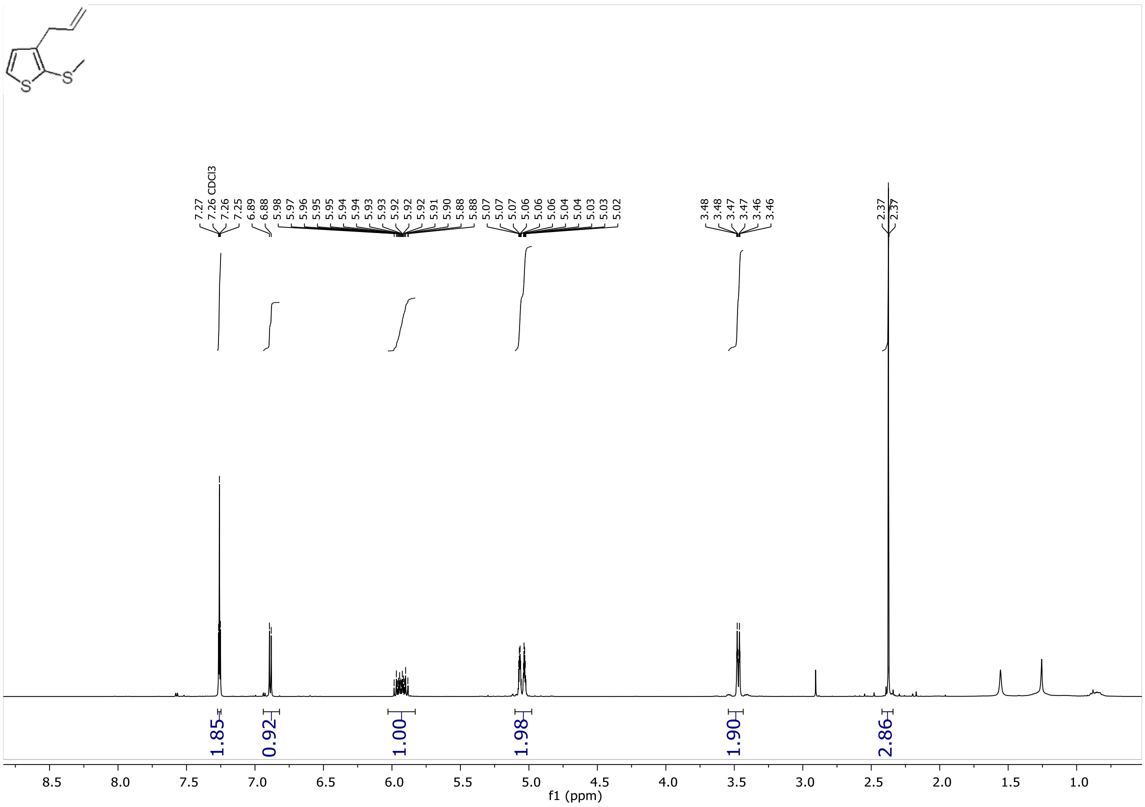

¹³C NMR (101 MHz, Chloroform-*d*)

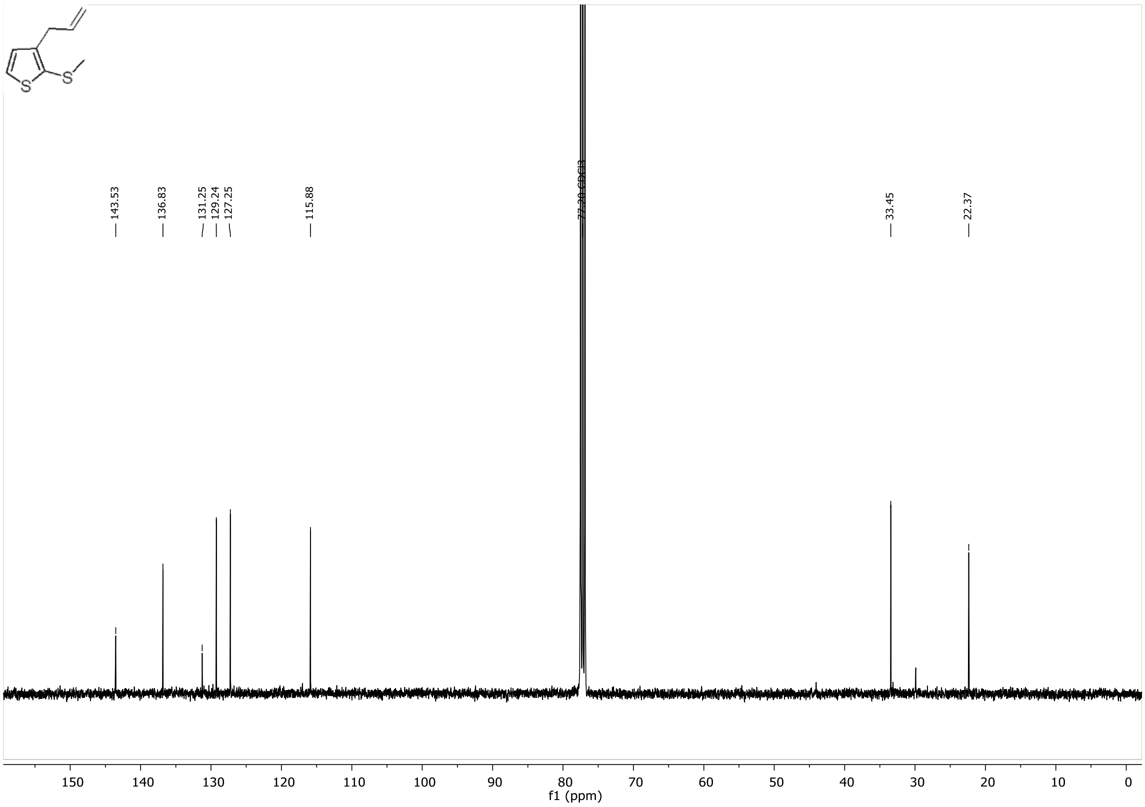

6b

^{1}H NMR (400 MHz, Chloroform-*d*)

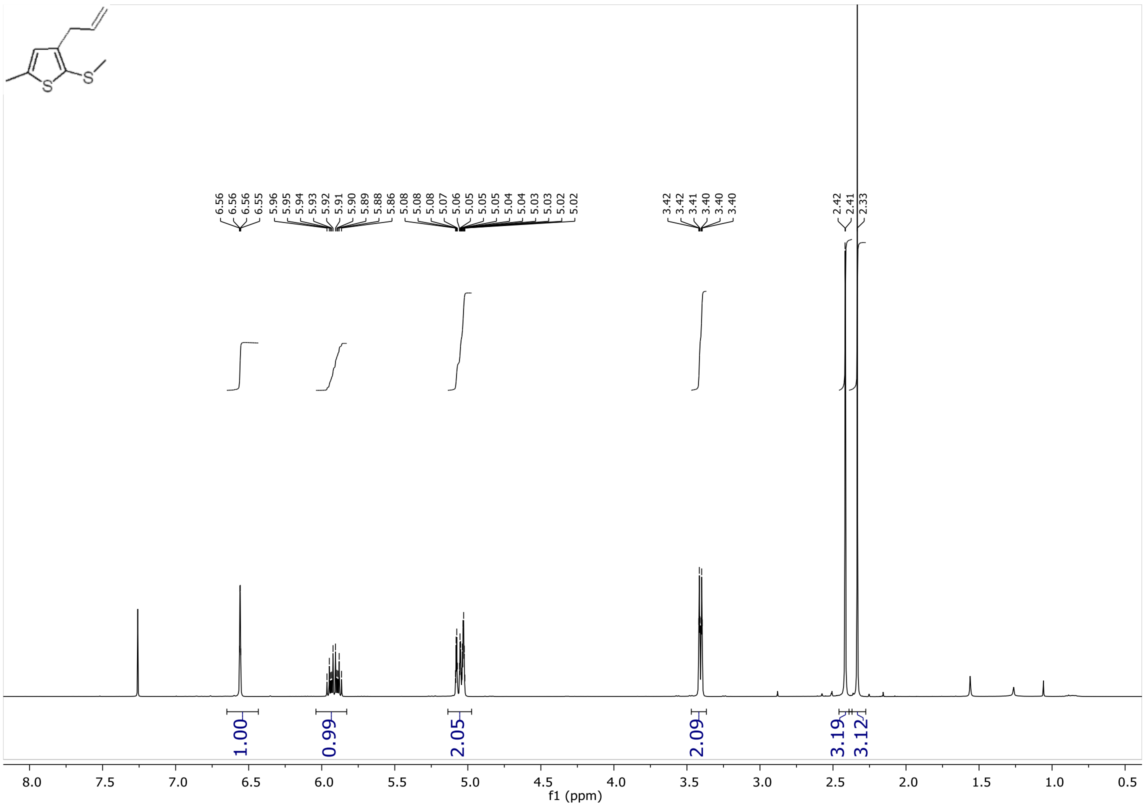

^{13}C NMR (101 MHz, Chloroform-*d*)

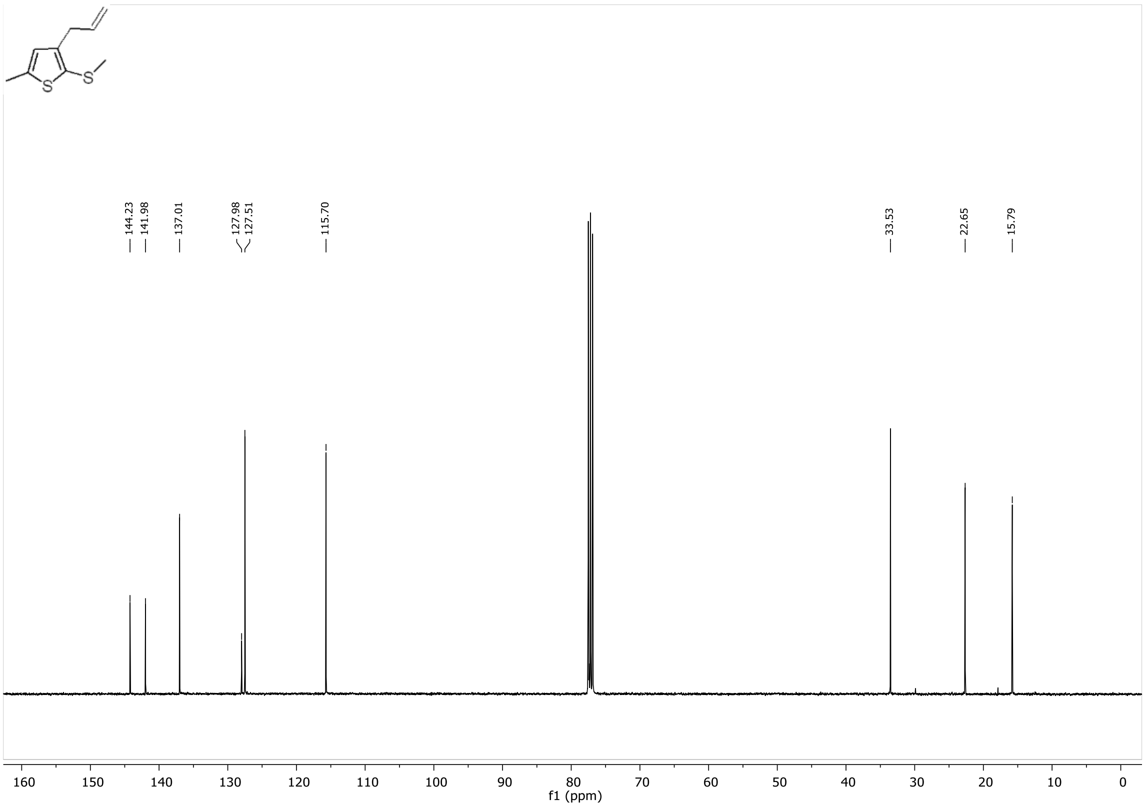

6c

^{1}H NMR (400 MHz, Chloroform-*d*)

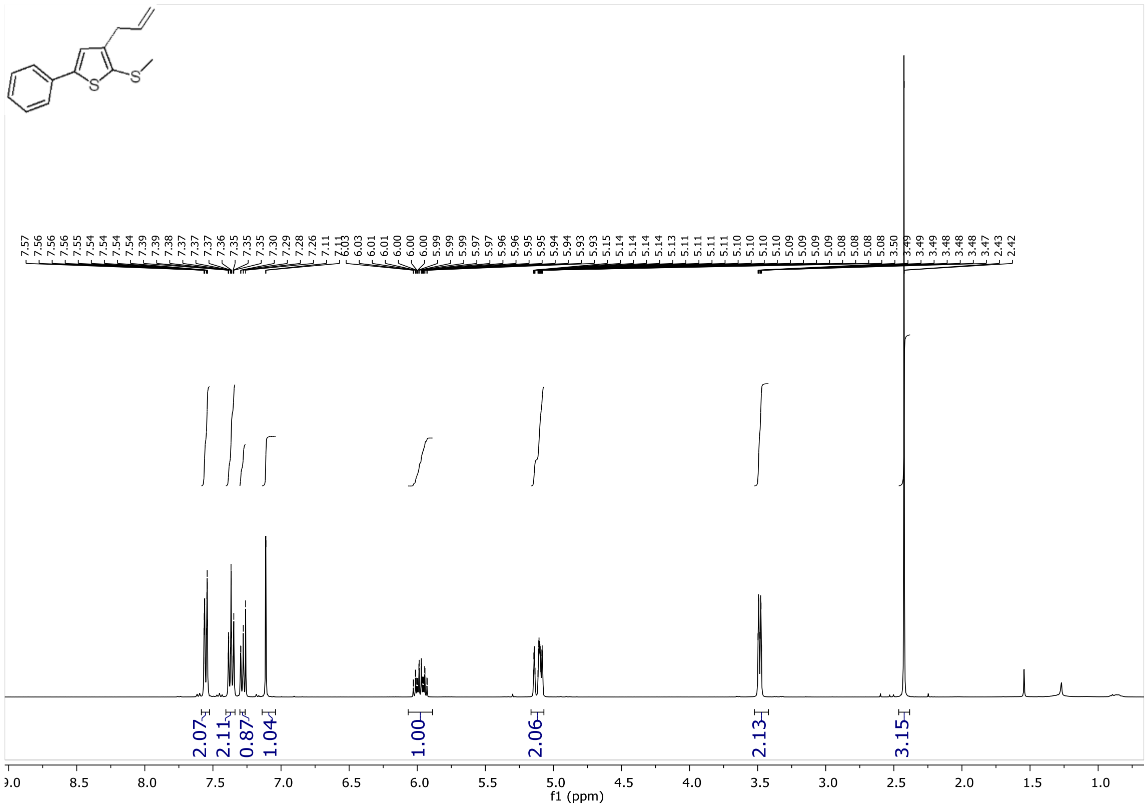

^{13}C NMR (101 MHz, Chloroform-*d*)

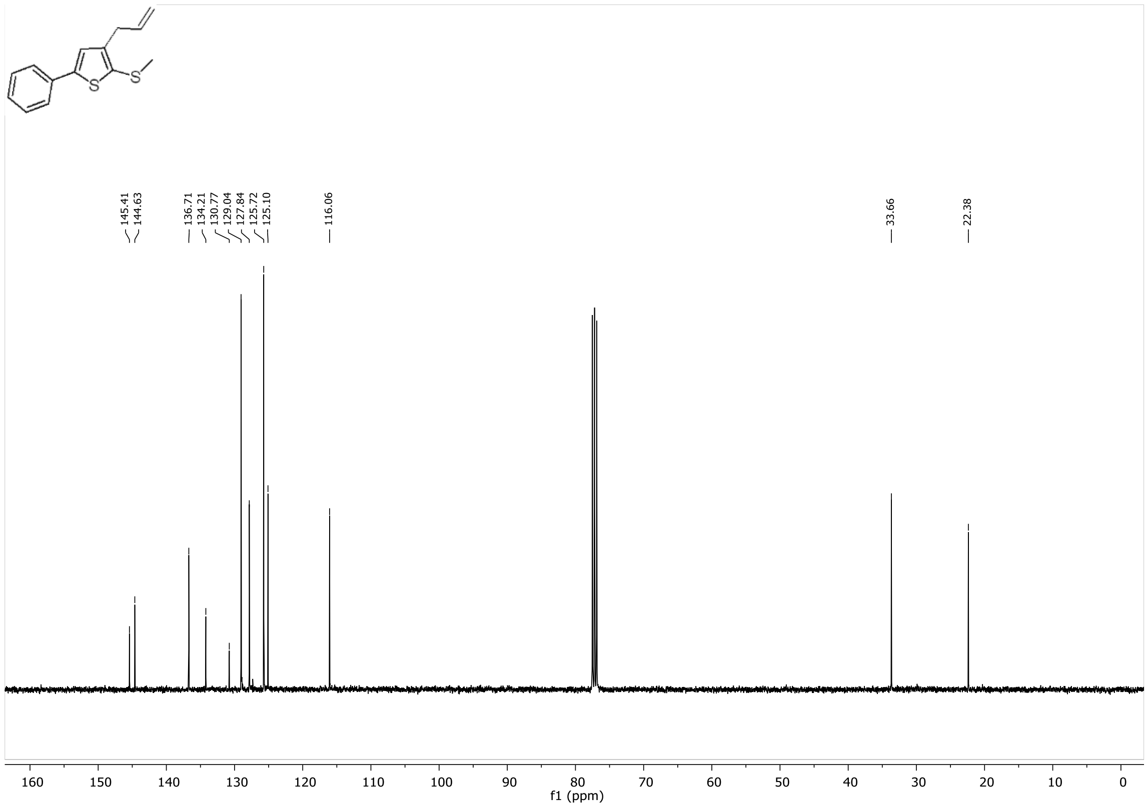

6d

^{1}H NMR (400 MHz, Chloroform-*d*)

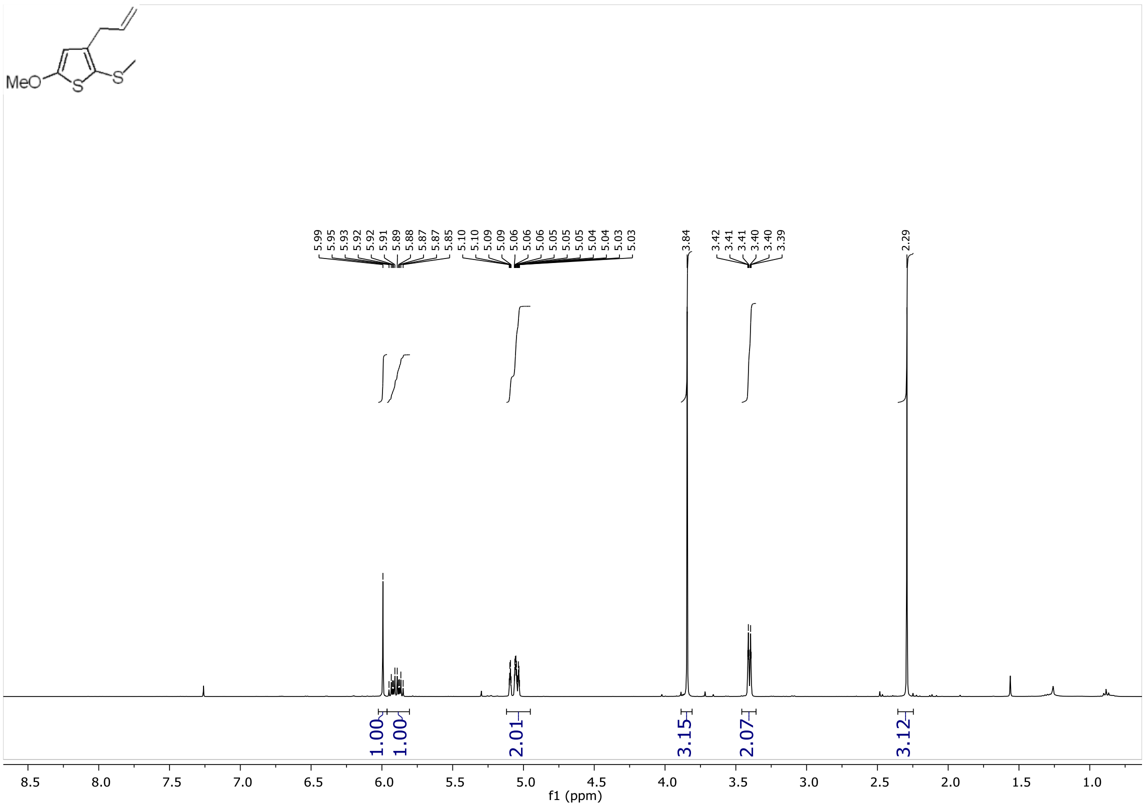

^{13}C NMR (101 MHz, Chloroform-*d*)

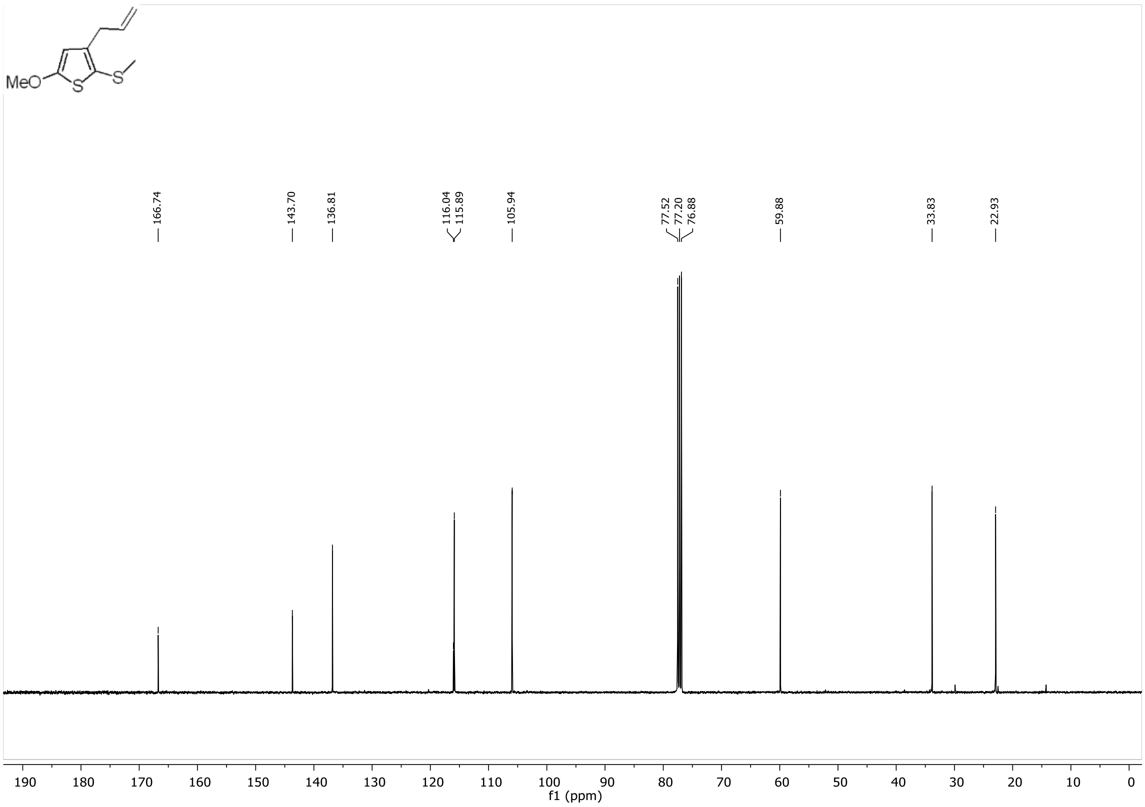

6e

^{1}H NMR (400 MHz, Chloroform-*d*)

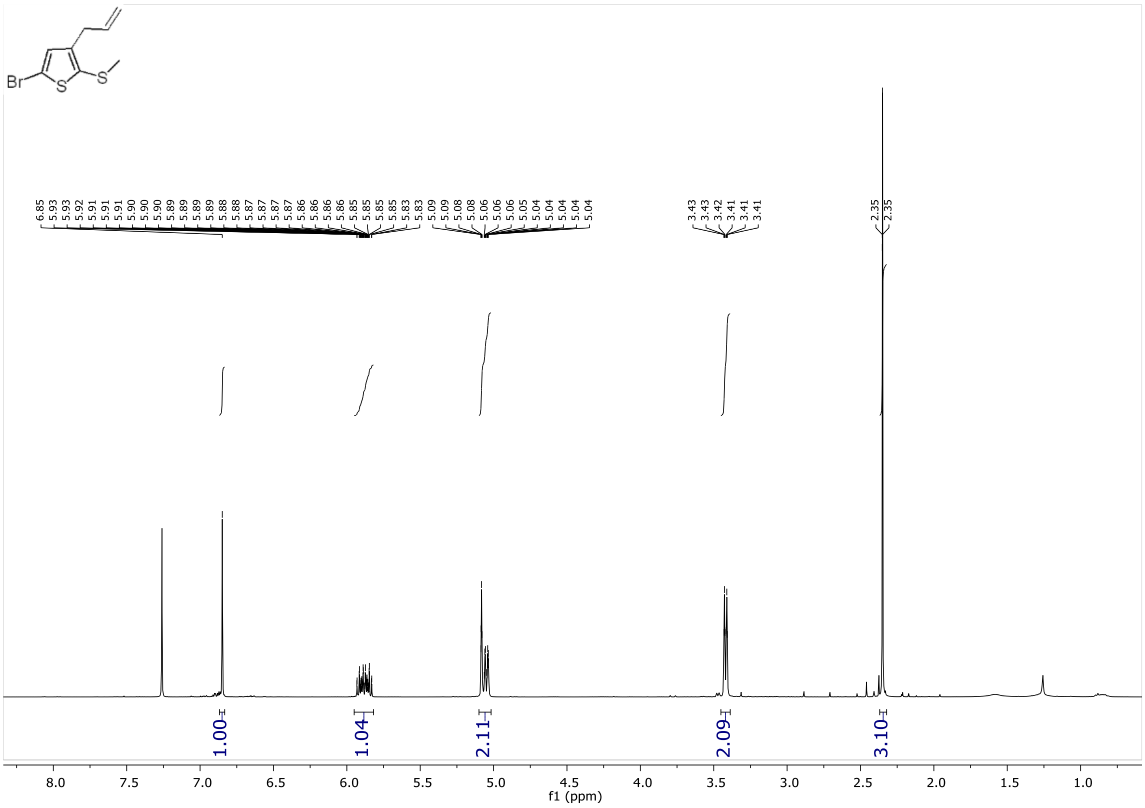

^{13}C NMR (101 MHz, Chloroform-*d*)

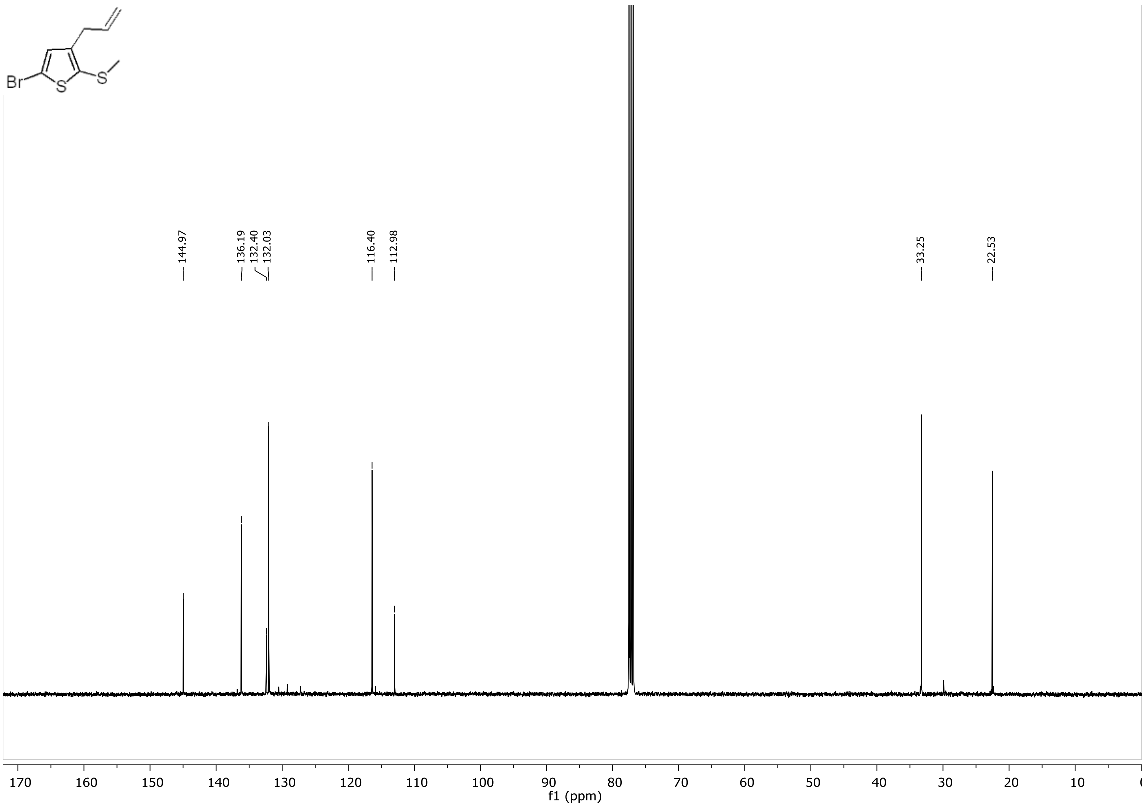

6f

^{1}H NMR (400 MHz, Chloroform-*d*)

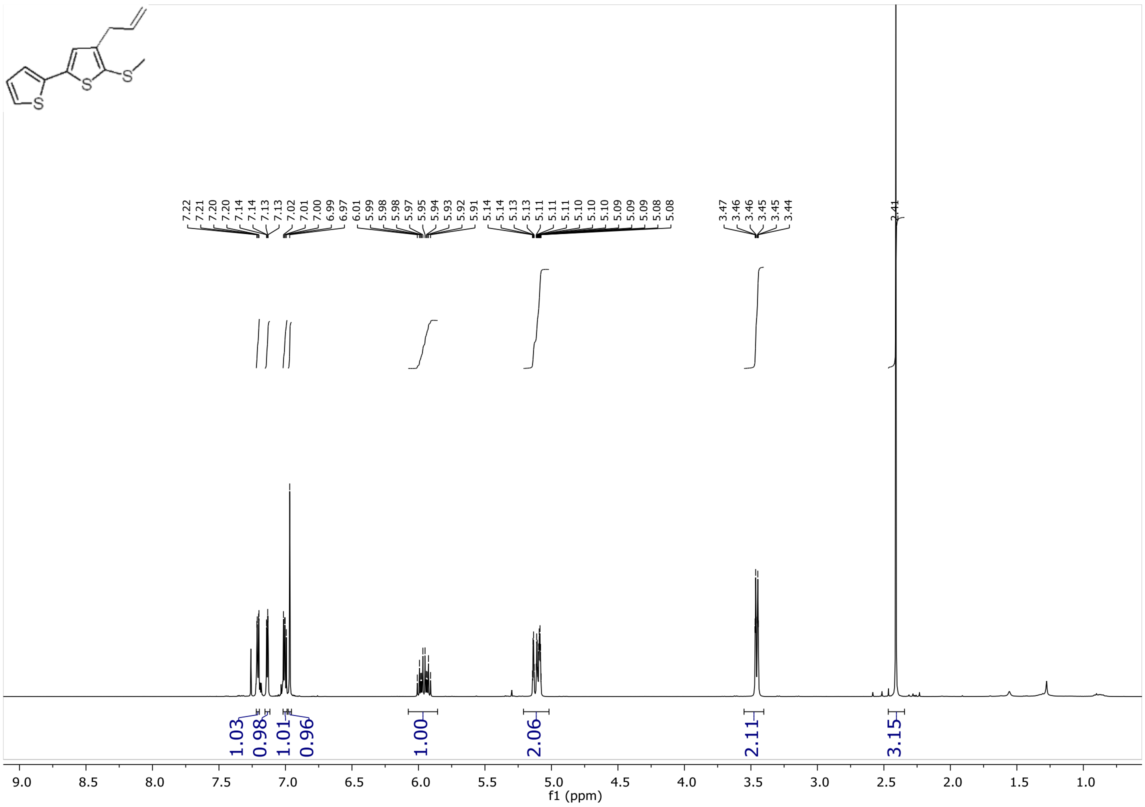

^{13}C NMR (101 MHz, Chloroform-*d*)

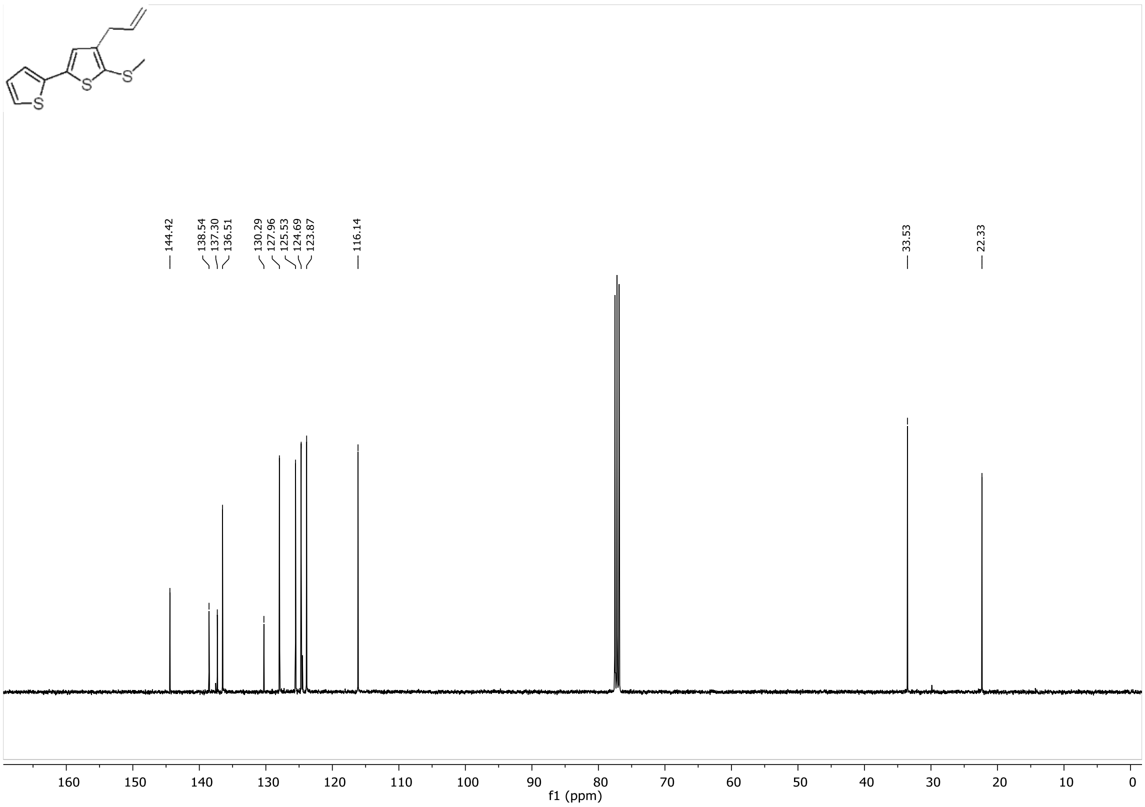

6g

^{1}H NMR (400 MHz, Chloroform-*d*)

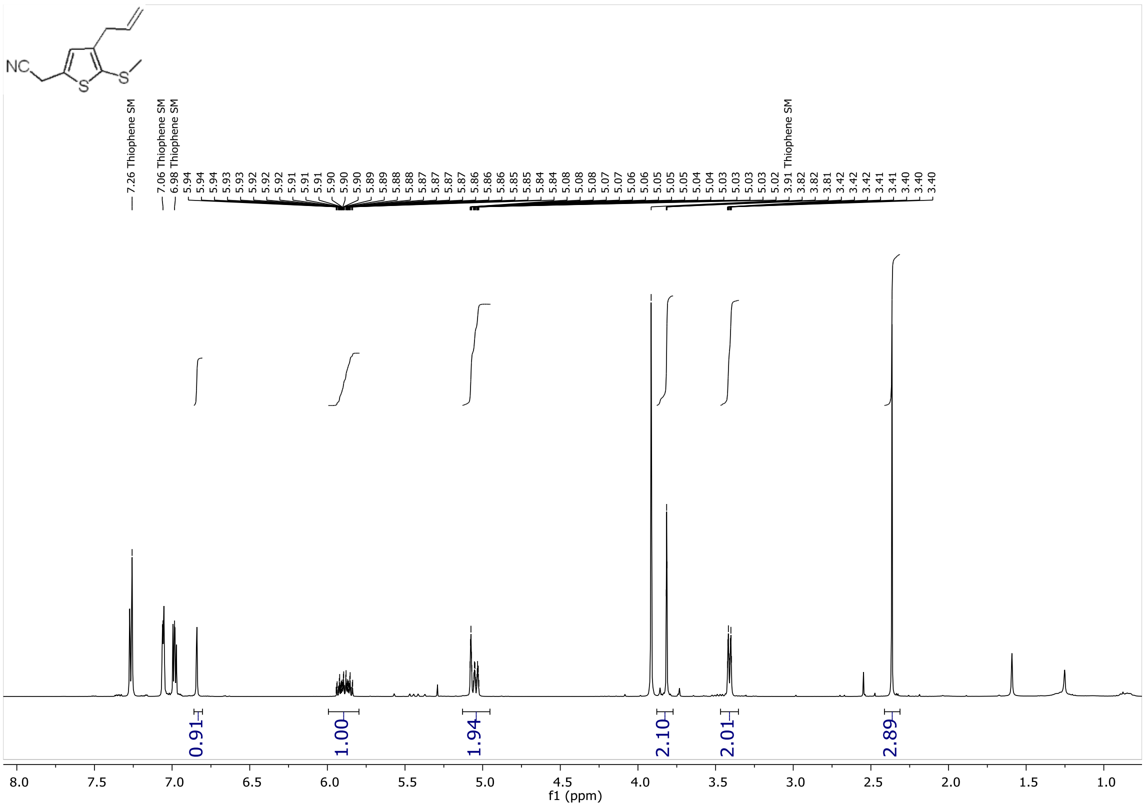

^{13}C NMR (101 MHz, Chloroform-*d*)

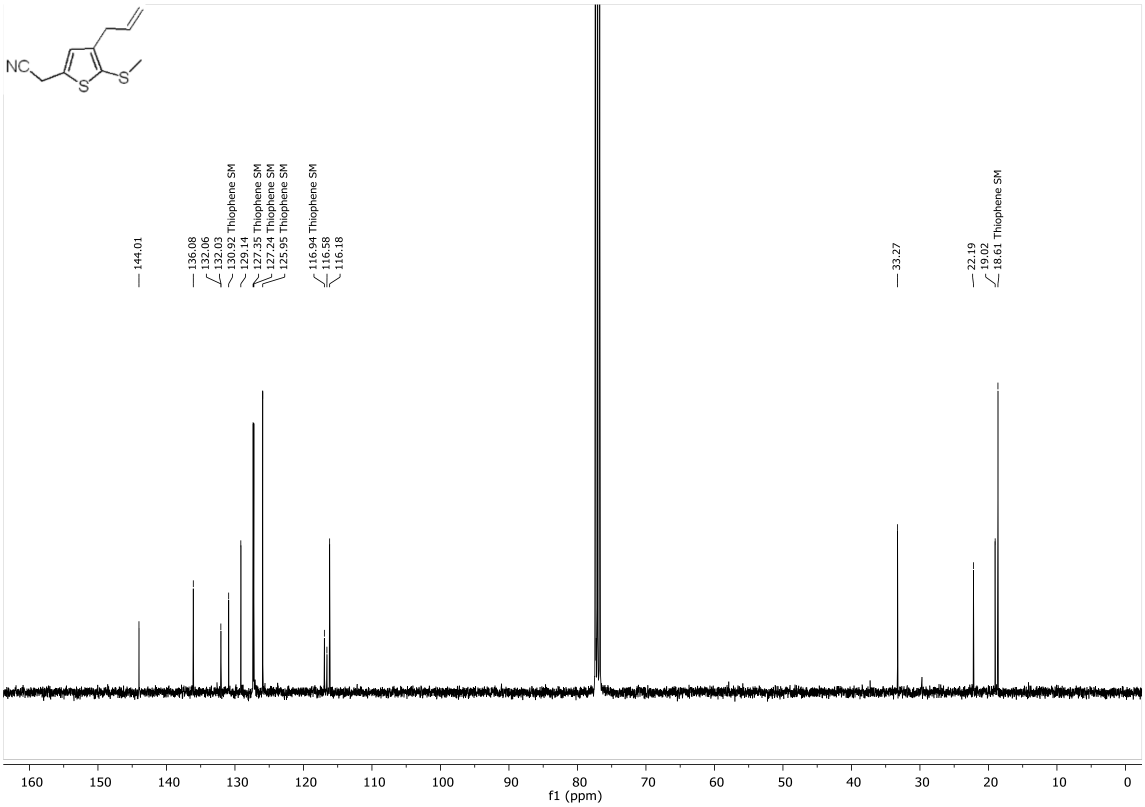

6h

^{1}H NMR (400 MHz, Chloroform-*d*)

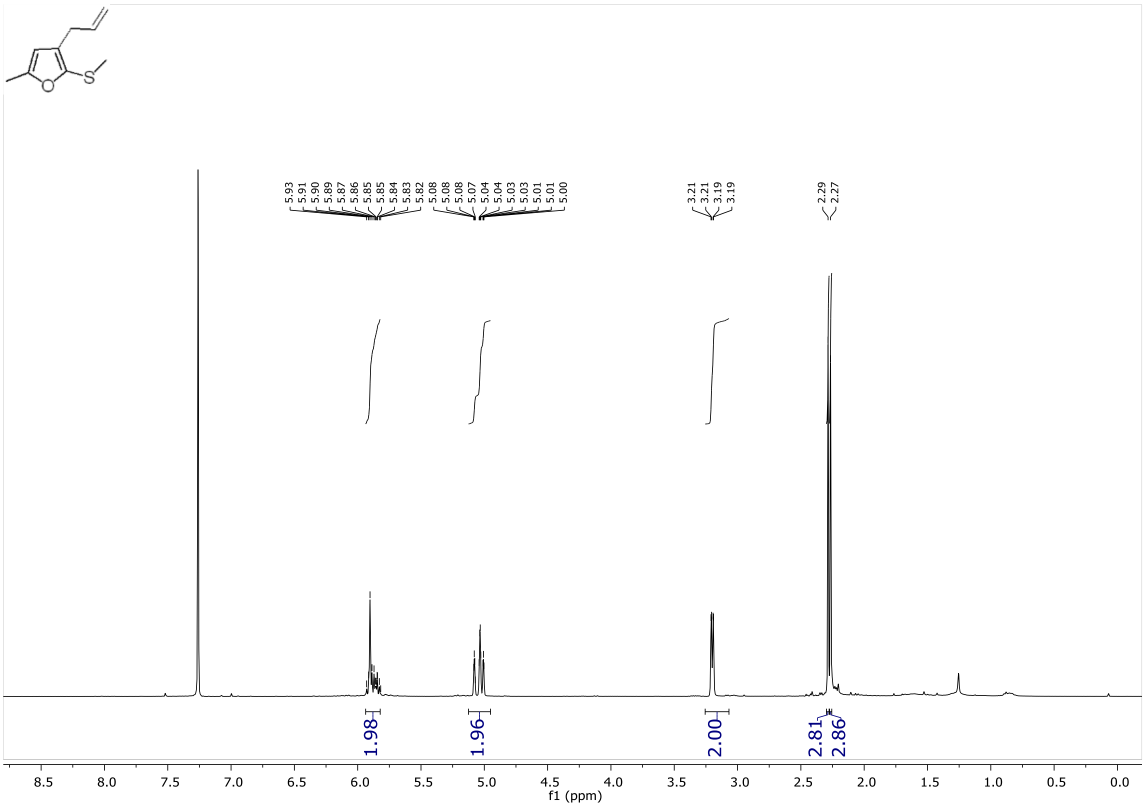

^{13}C NMR (101 MHz, Chloroform-*d*)

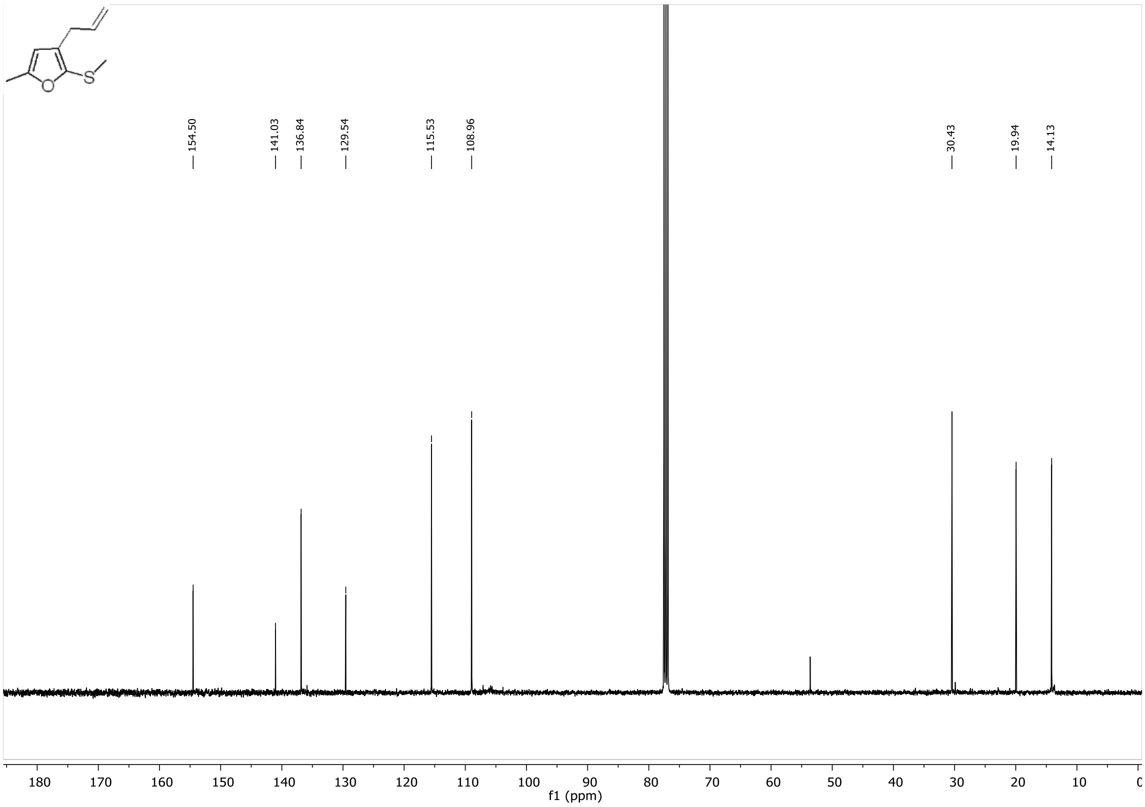

6i

^{1}H NMR (400 MHz, Chloroform-*d*)

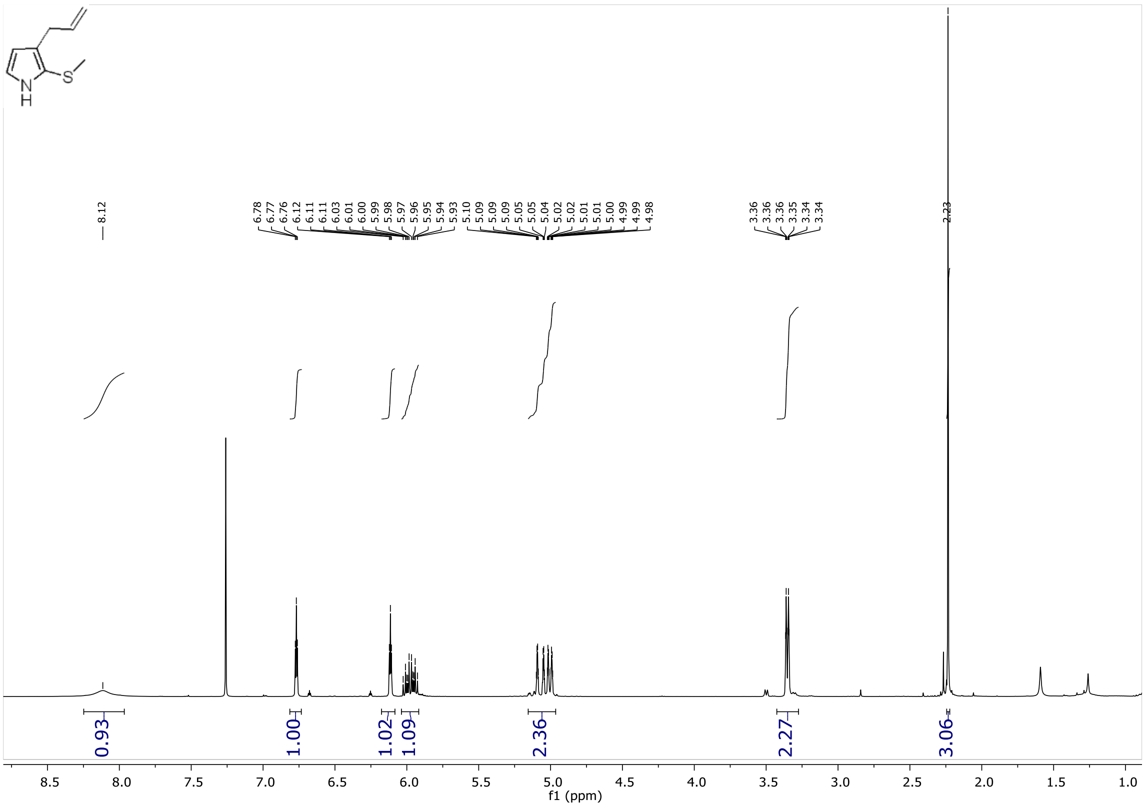

^{13}C NMR (101 MHz, Chloroform-*d*)

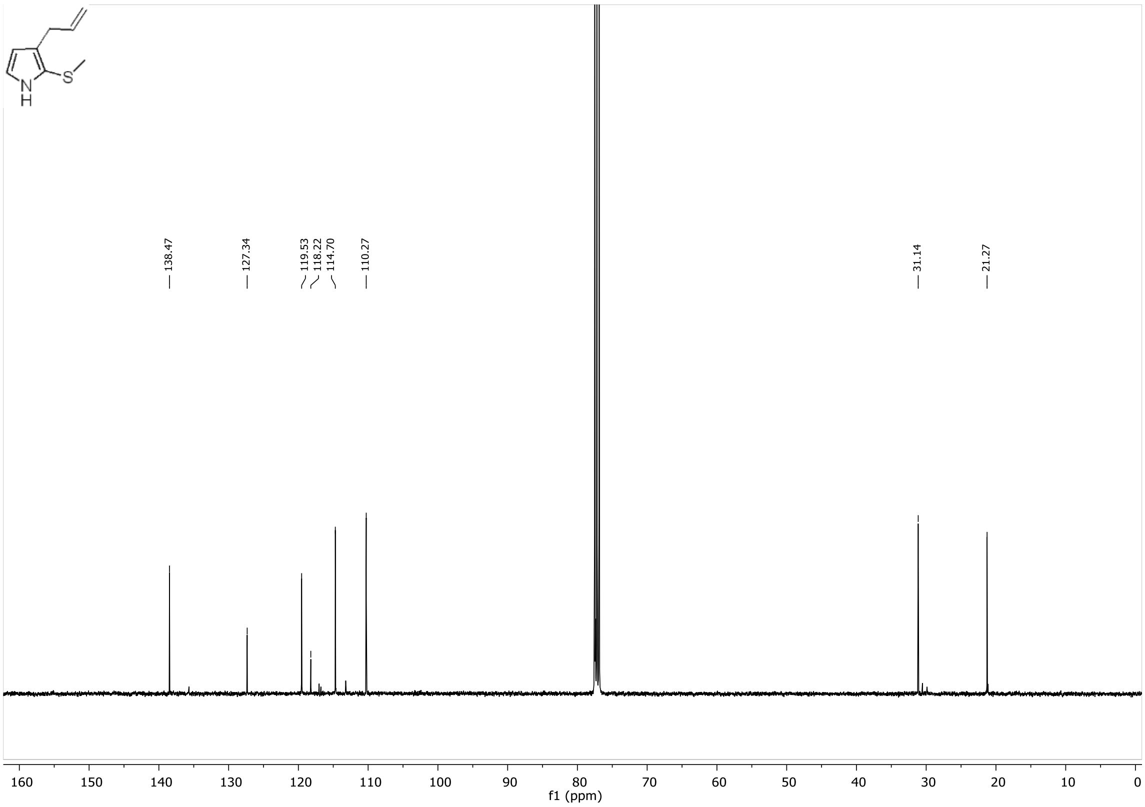

6j

^{1}H NMR (400 MHz, Chloroform-*d*)

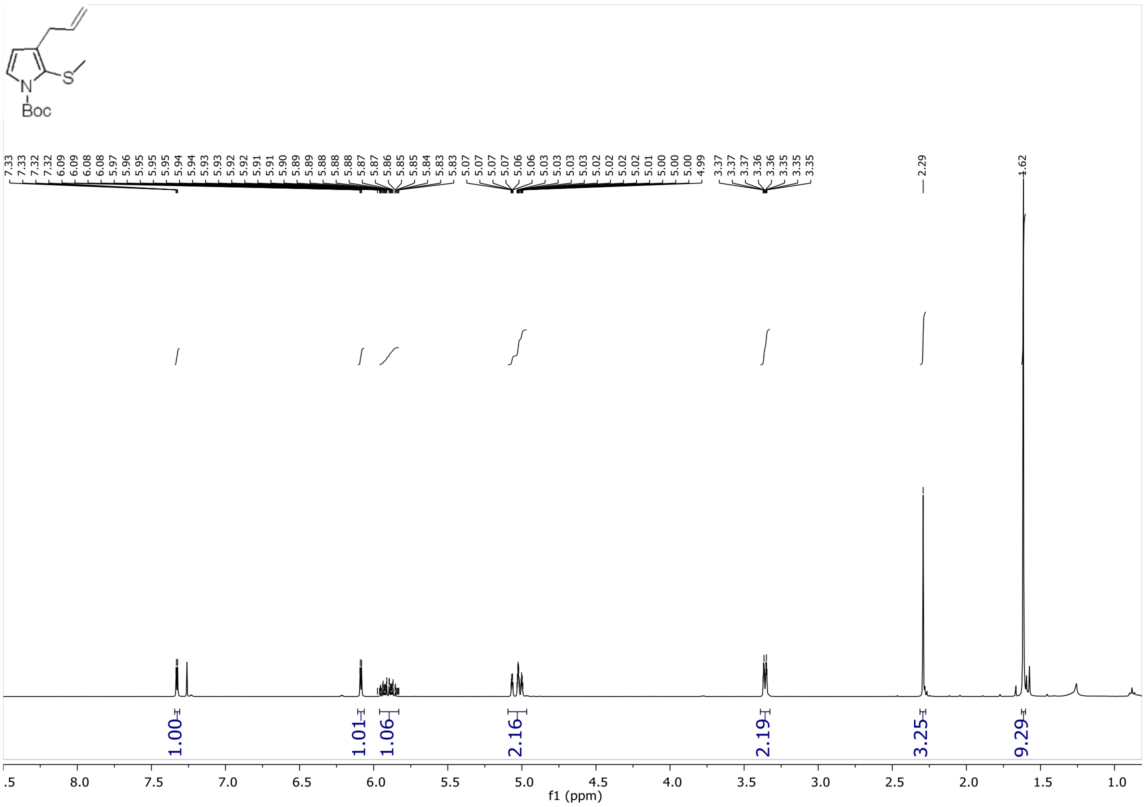

^{13}C NMR (101 MHz, Chloroform-*d*)

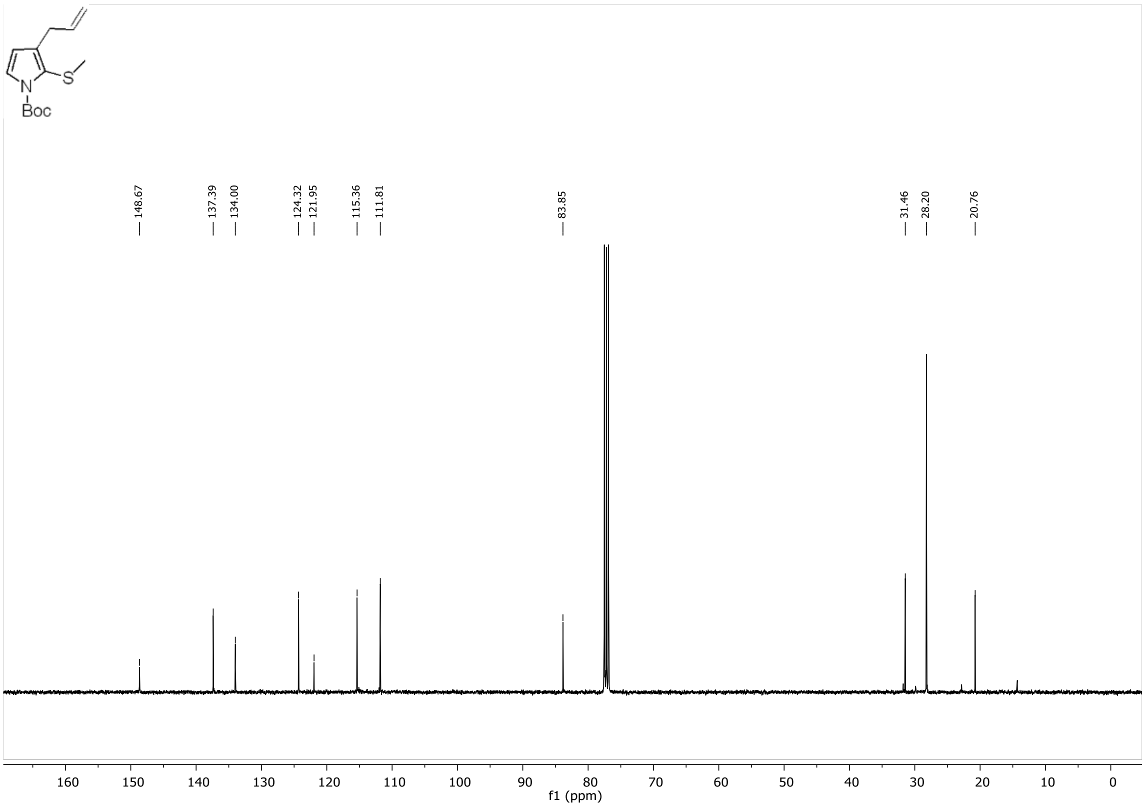

6k

^{1}H NMR (400 MHz, Chloroform-*d*)

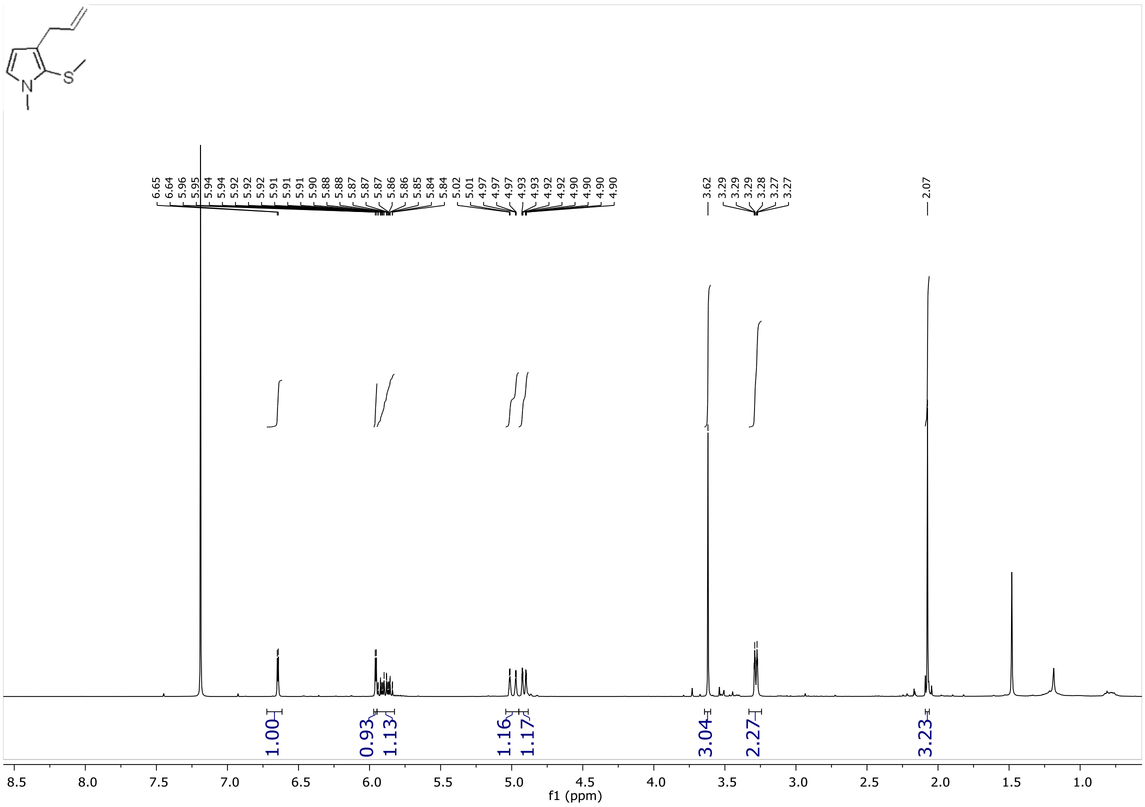

^{13}C NMR (101 MHz, Chloroform-*d*)

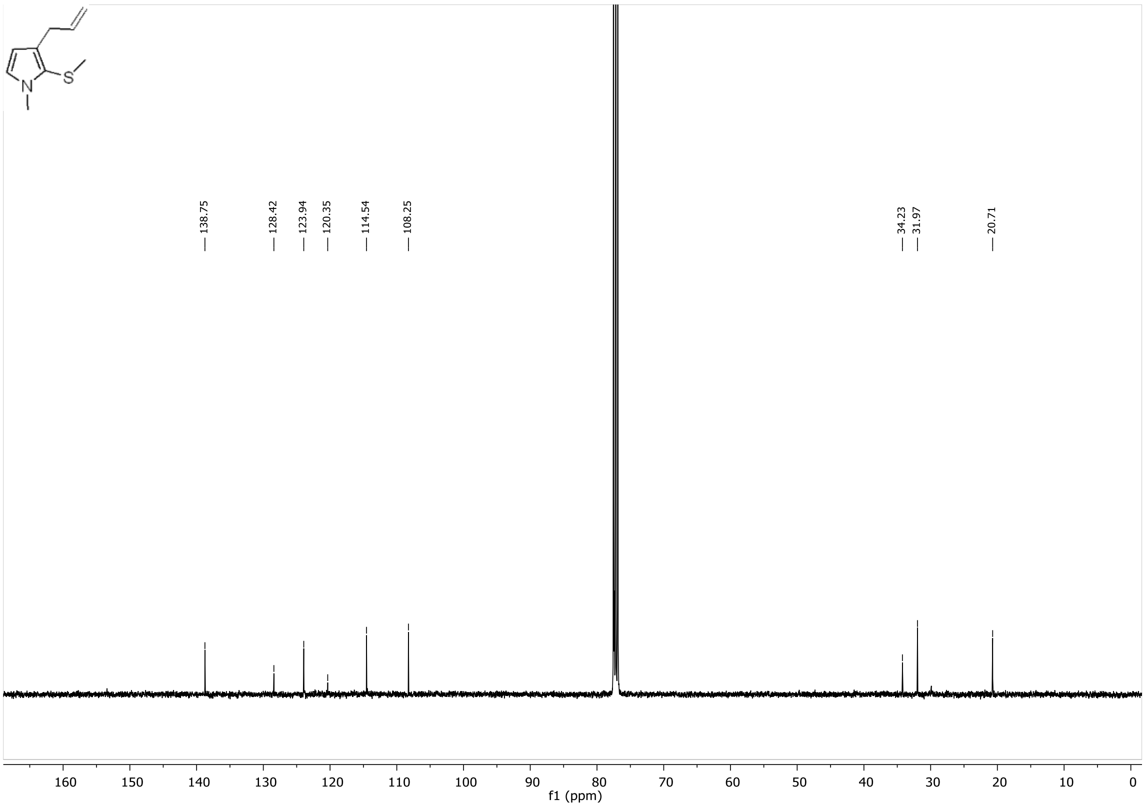

^1H-^{13}C HMBC

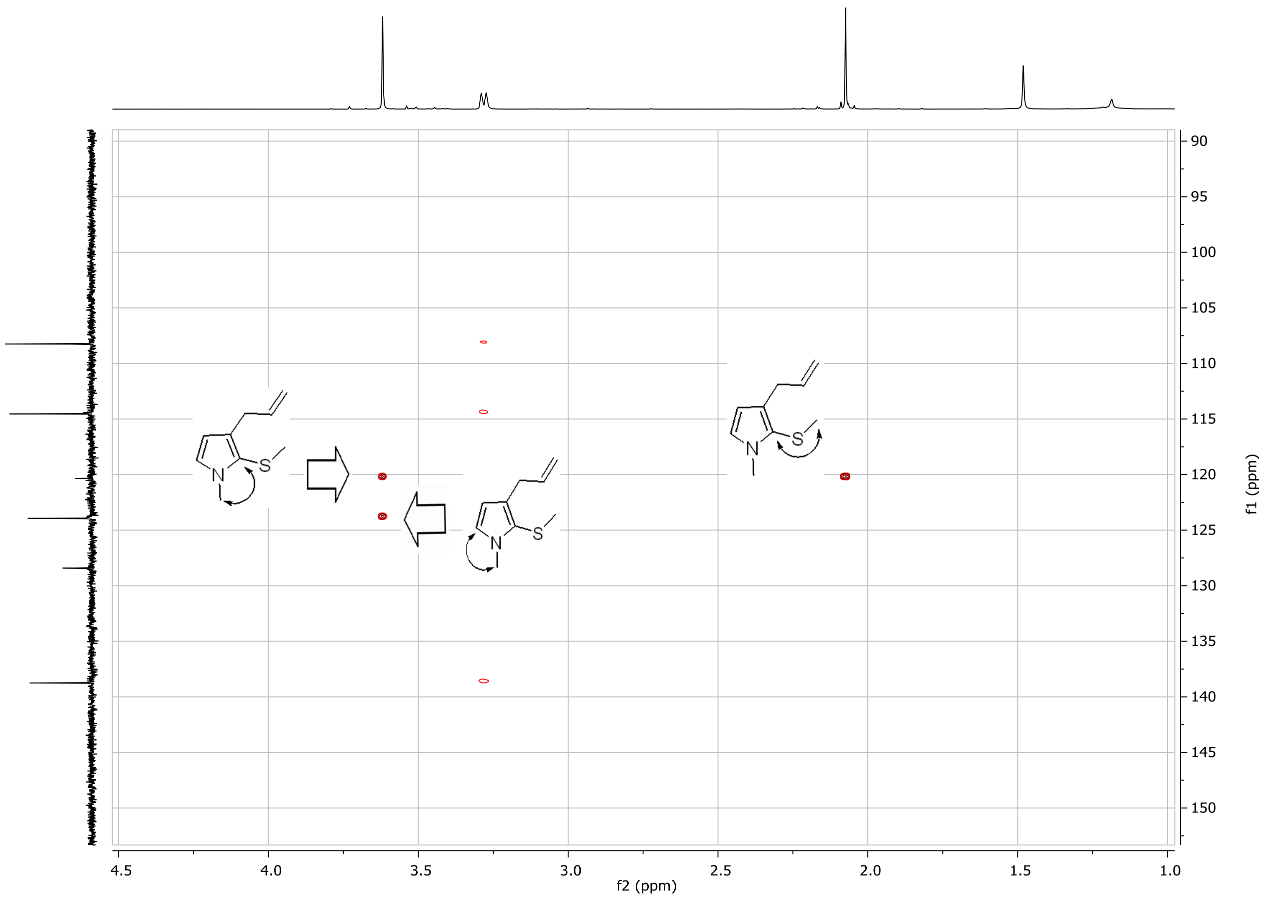

N
S
f2 (ppm)
f1 (ppm)
4.5
4.0
3.5
3.0
2.5
2.0
1.5
1.0
90
95
100
105
110
115
120
125
130
135
140
145
150

7k

^{1}H NMR (400 MHz, Chloroform-*d*)

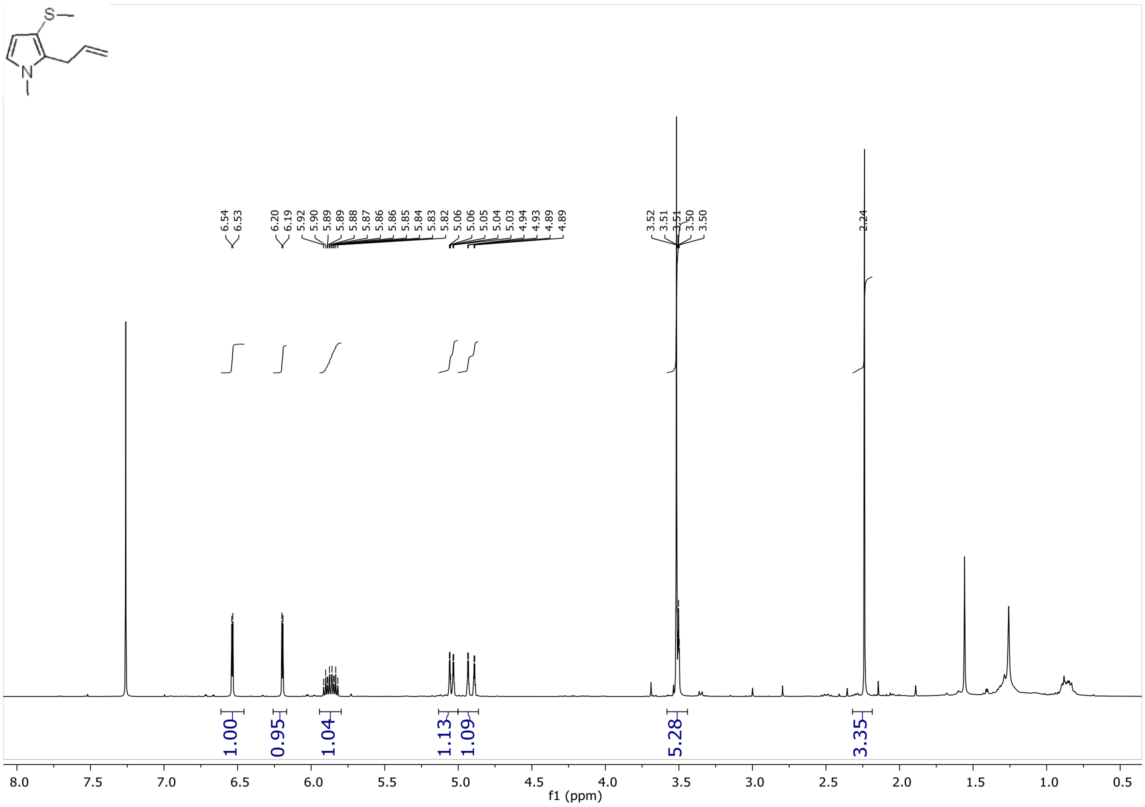

^{13}C NMR (101 MHz, Chloroform-*d*)

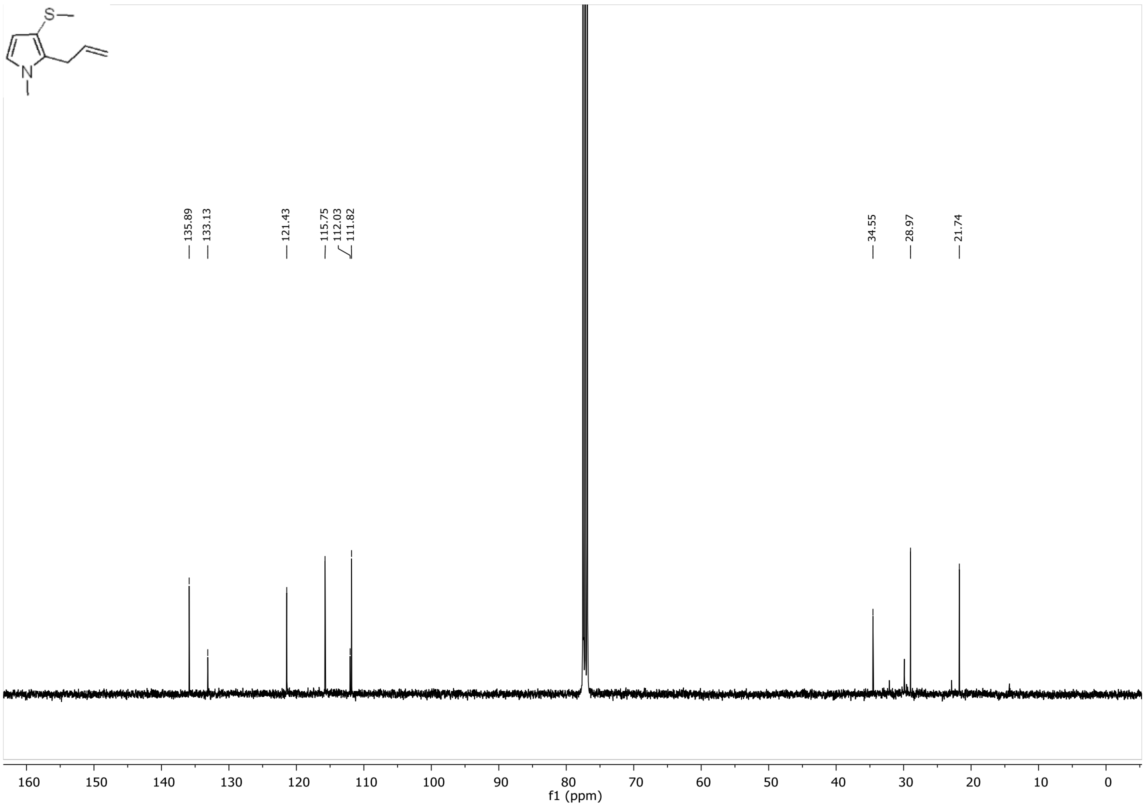

^{1}H-^{13}C HMBC

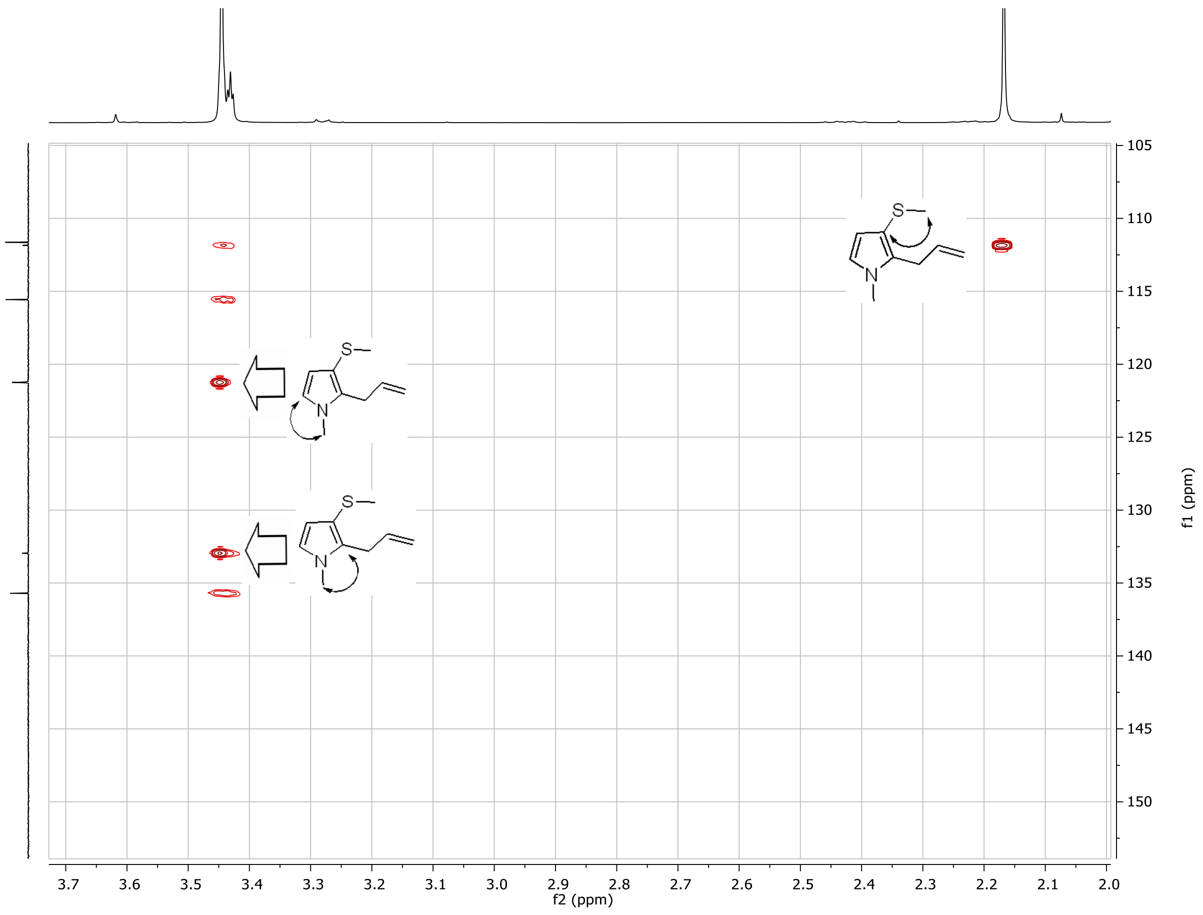

6l

^{1}H NMR (400 MHz, Chloroform-*d*)

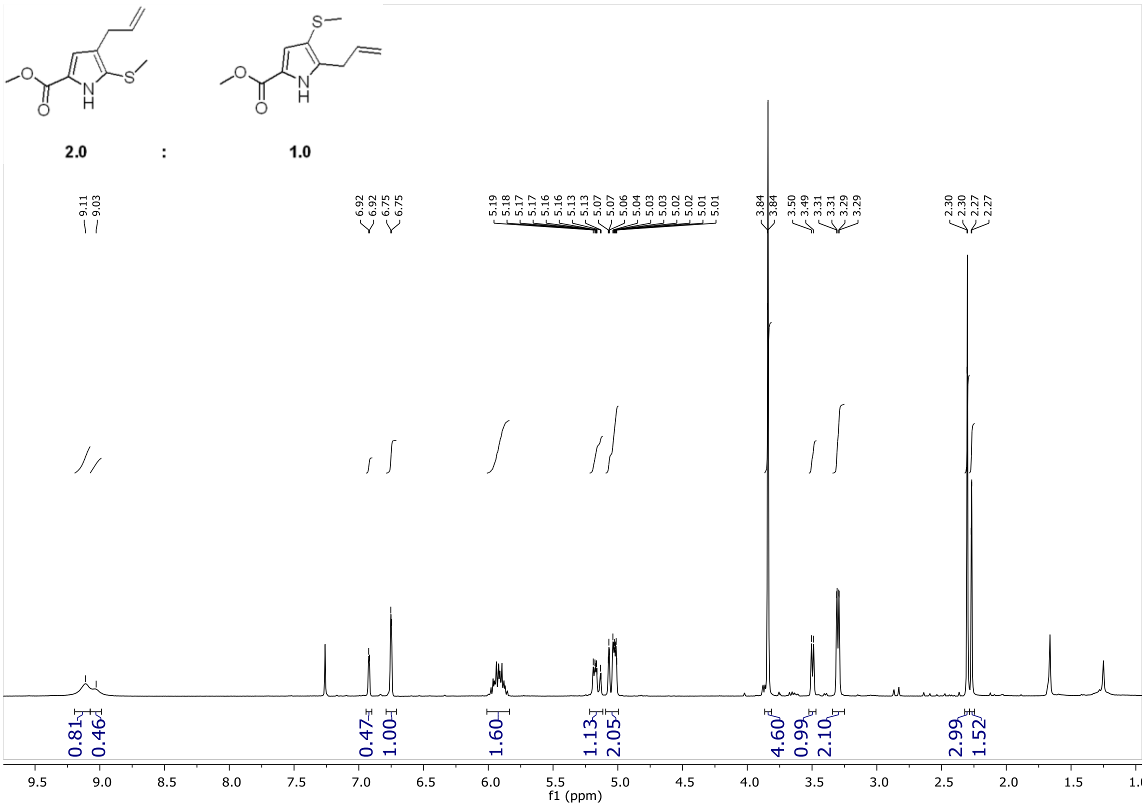

^{13}C NMR (101 MHz, Chloroform-*d*)

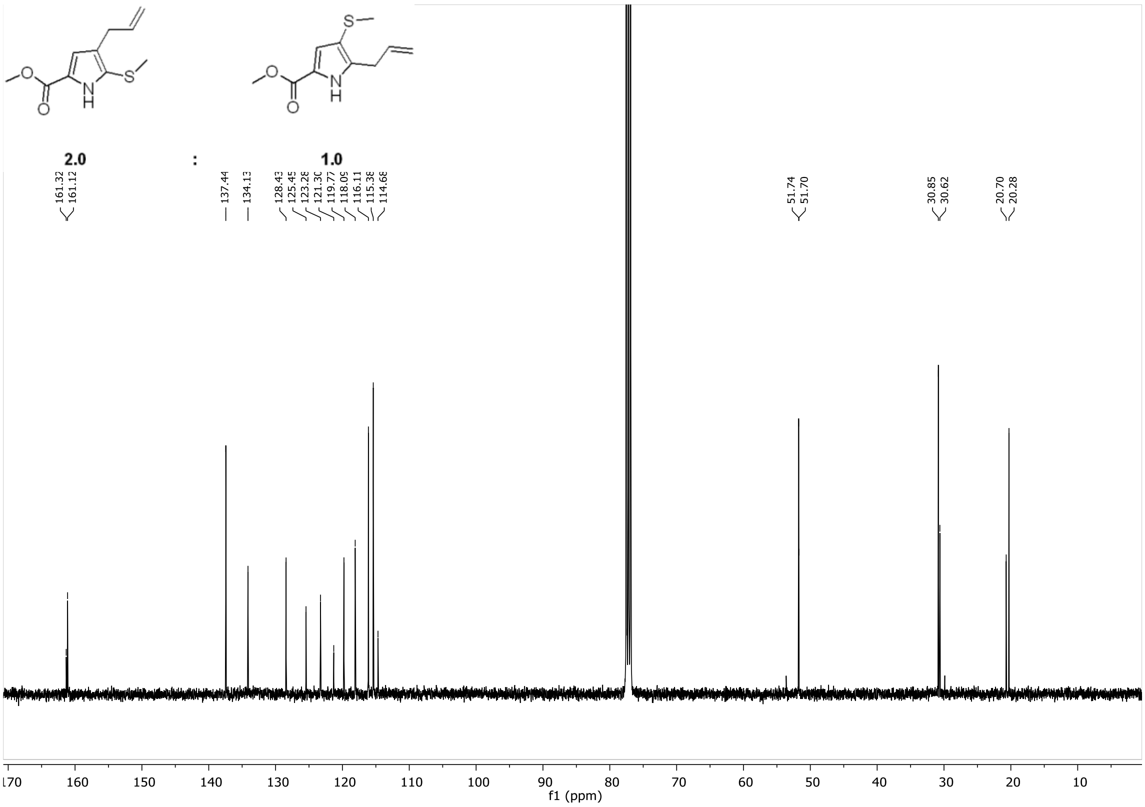

^{1}H-^{13}C HMBC

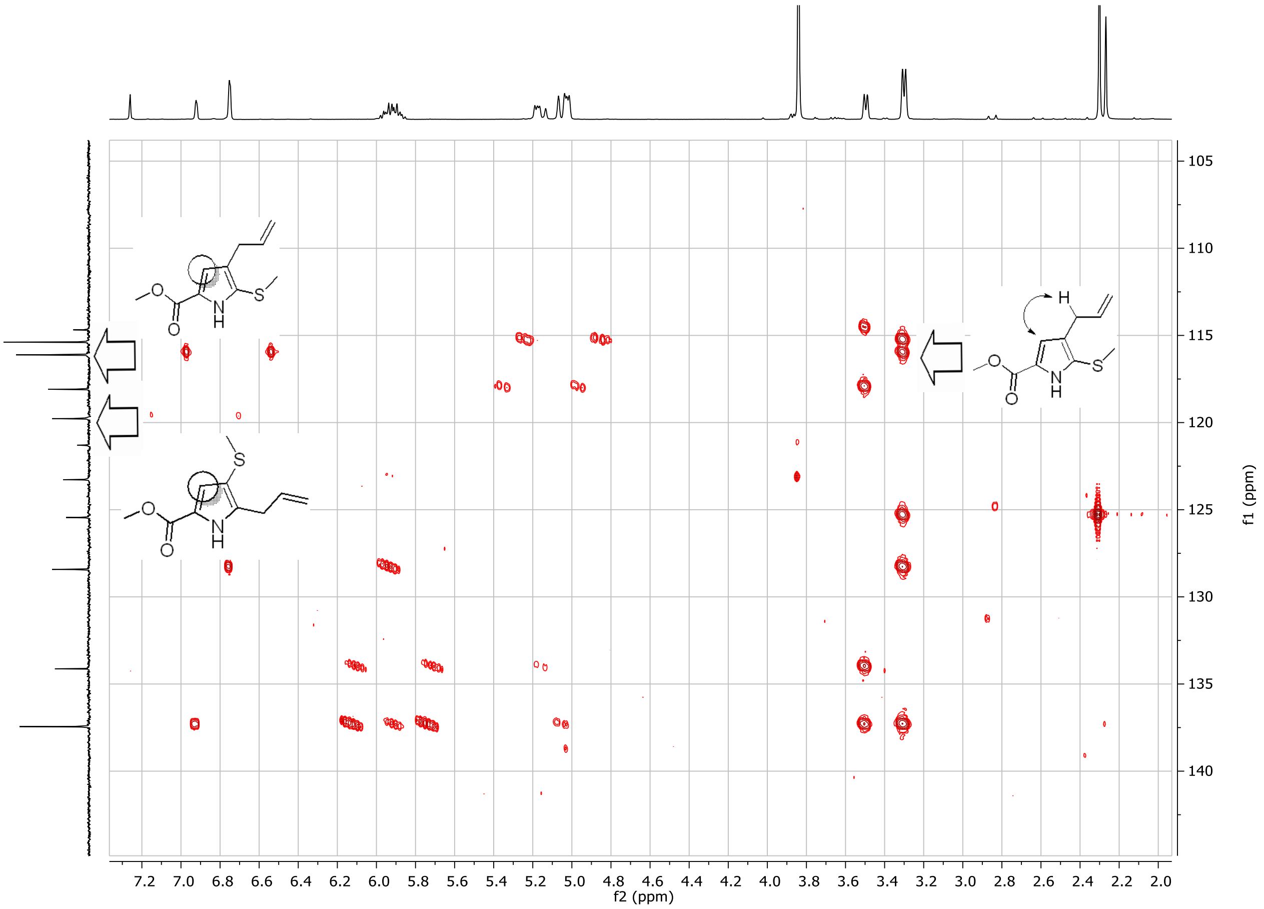

6m

^{1}H NMR (400 MHz, Chloroform-*d*)

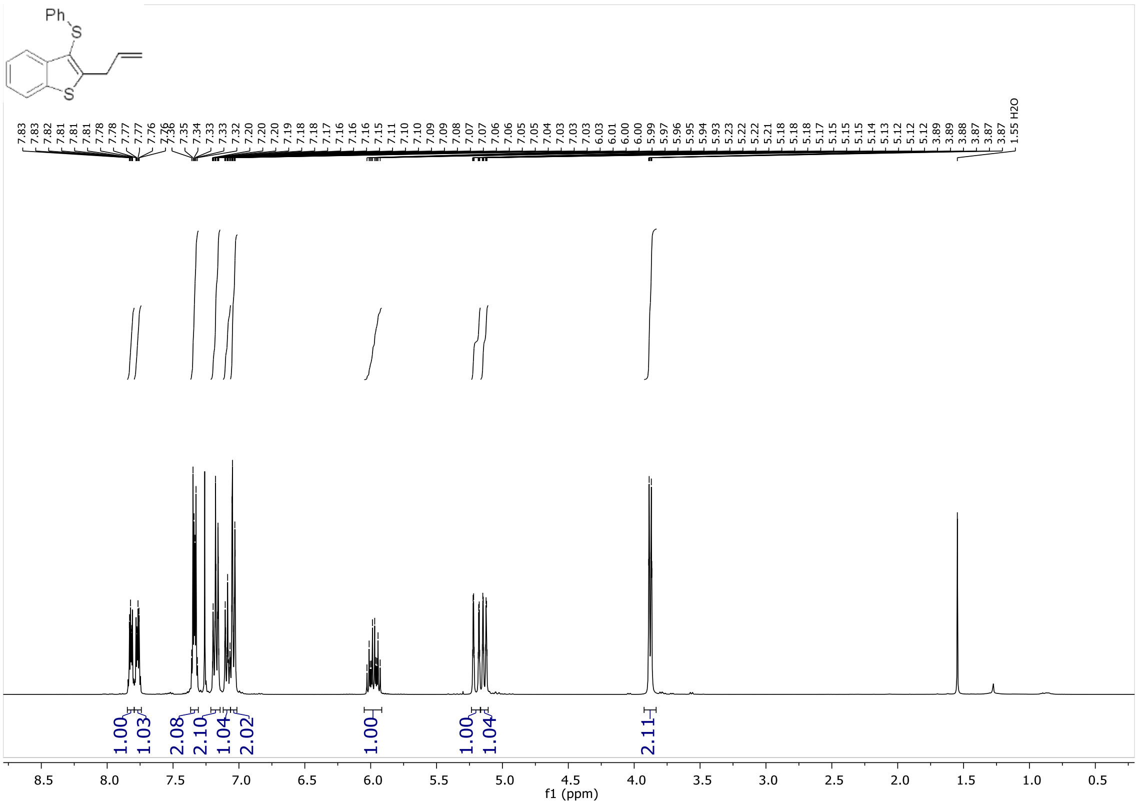

^{13}C NMR (101 MHz, Chloroform-*d*)

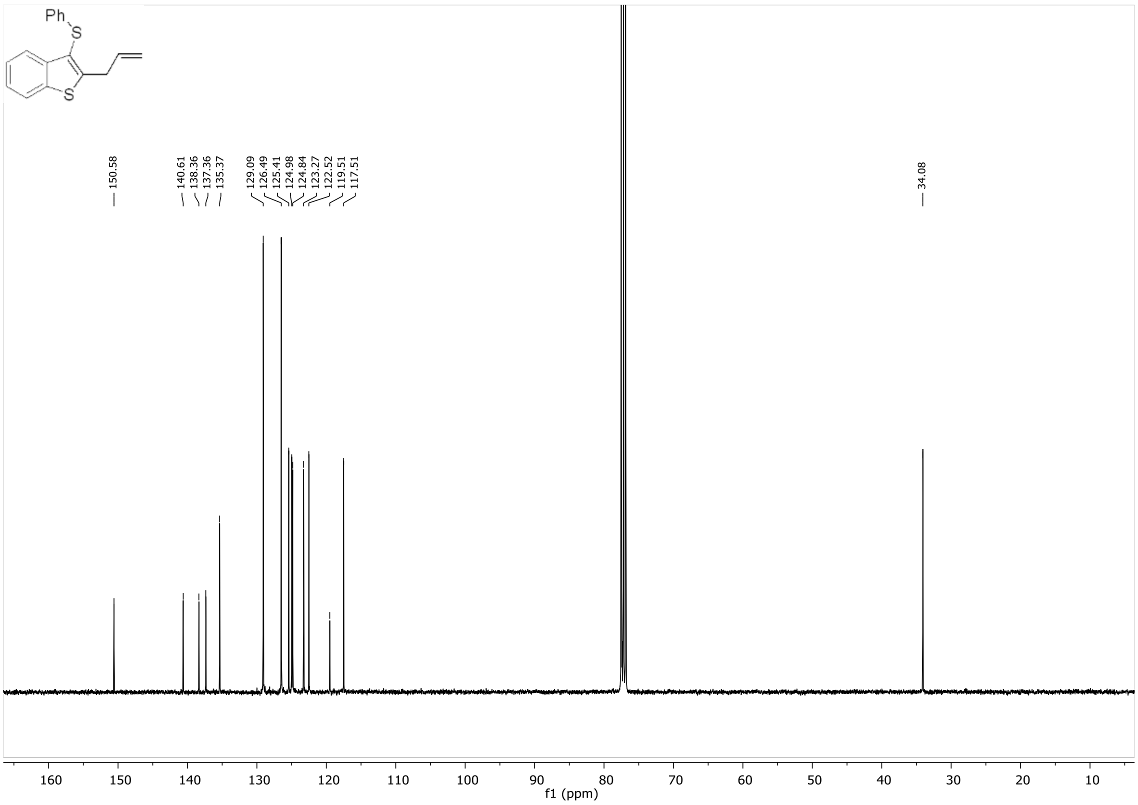

^1H-^{13}C HMBC

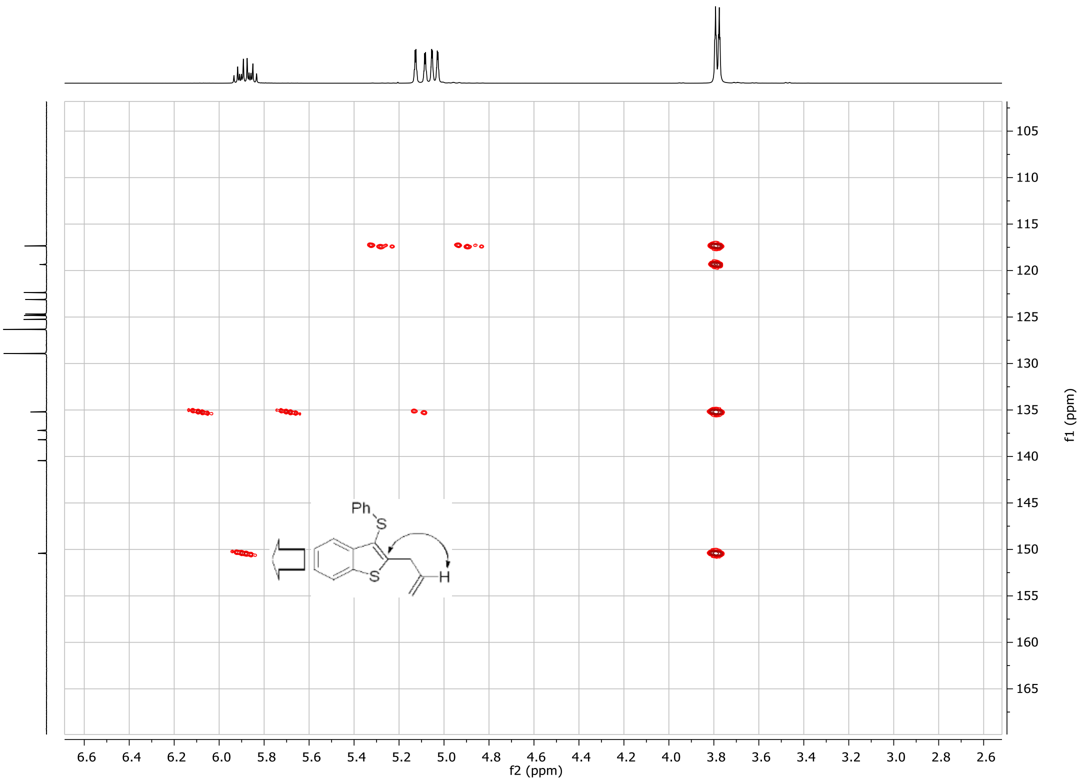

Ph
S
S
H
f1 (ppm)
105
110
115
120
125
130
135
140
145
150
155
160
165
6.6 6.4 6.2 6.0 5.8 5.6 5.4 5.2 5.0 4.8 4.6 4.4 4.2 4.0 3.8 3.6 3.4 3.2 3.0 2.8 2.6
f2 (ppm)

6n

^{1}H NMR (400 MHz, Chloroform-*d*)

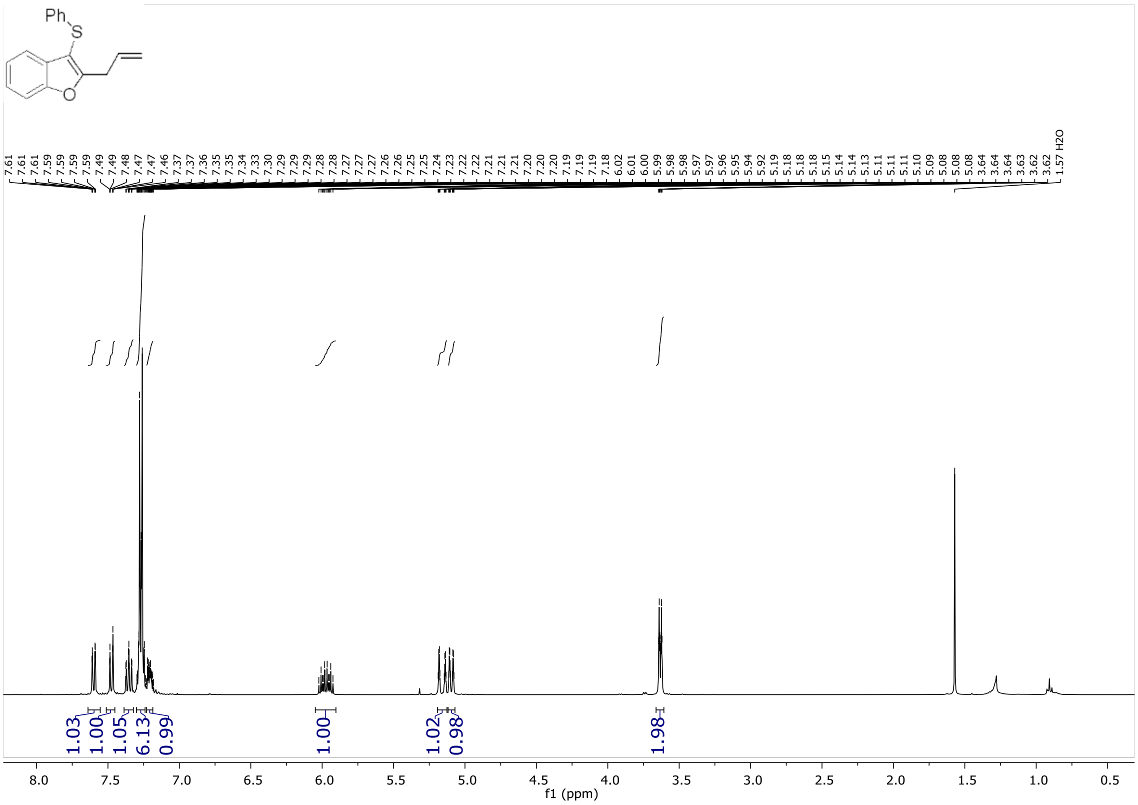

^{13}C NMR (101 MHz, Chloroform-*d*)

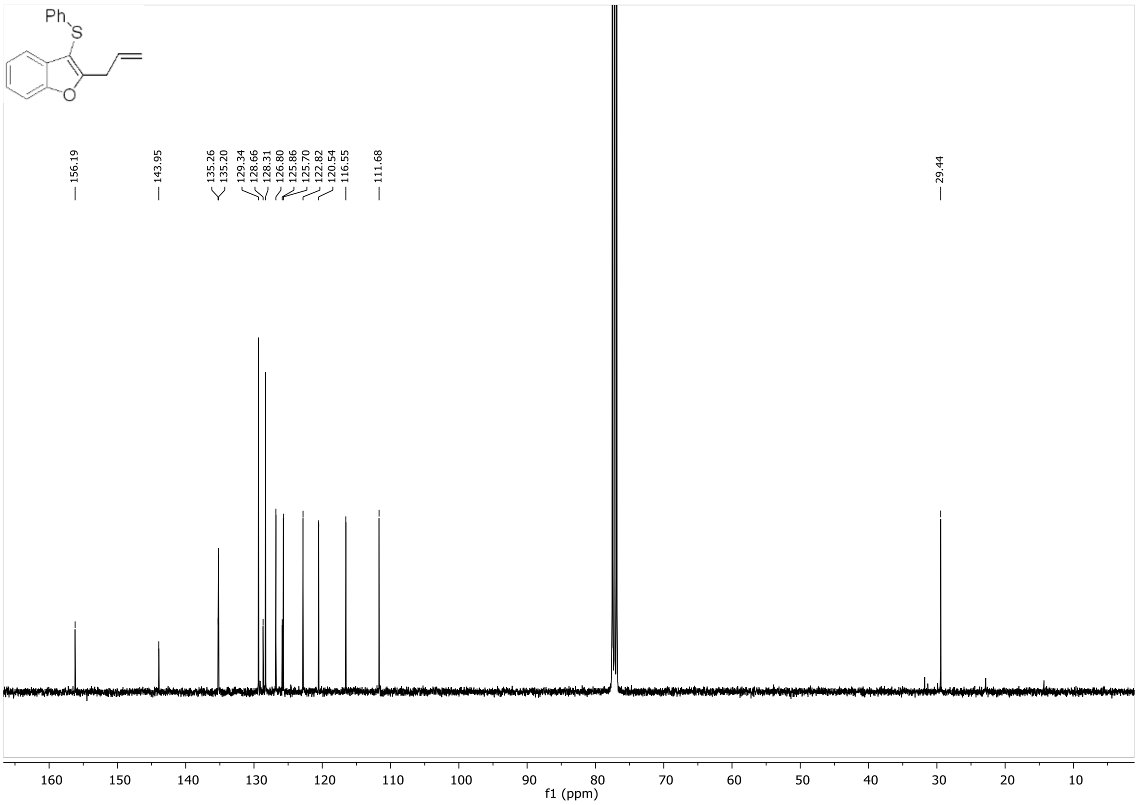

^{1}H-^{13}C HMBC

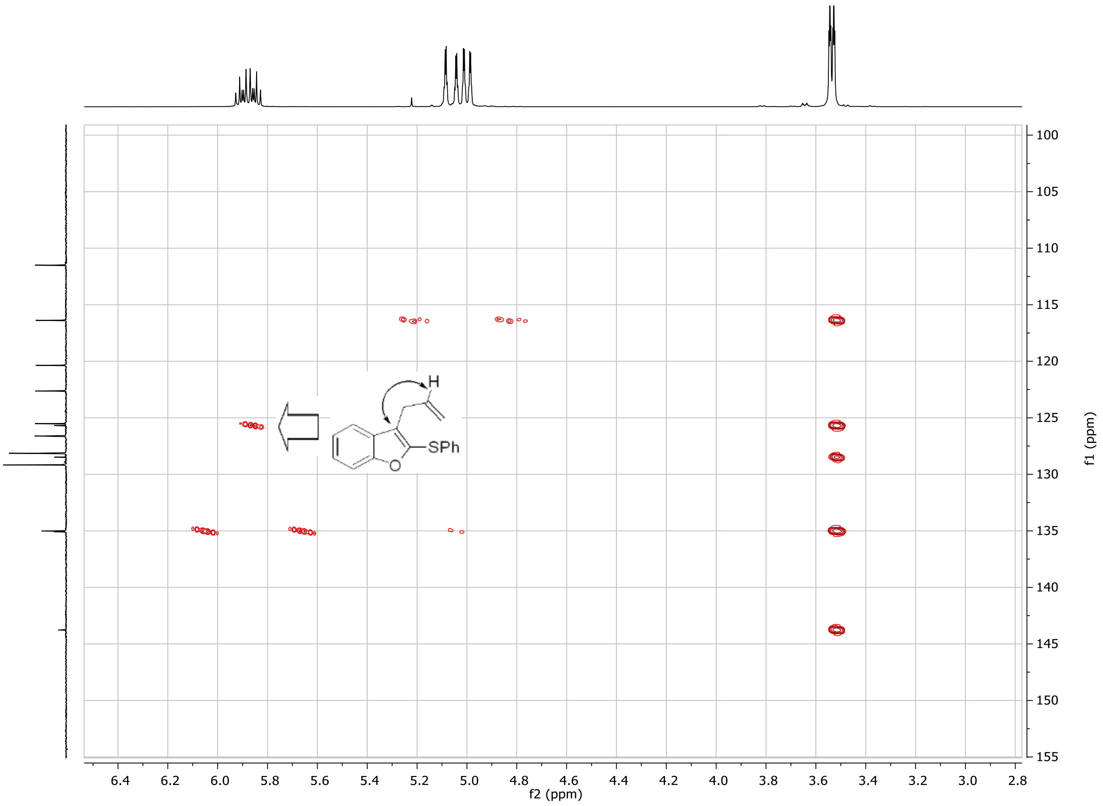

X-ray structures

CCDC 1570504

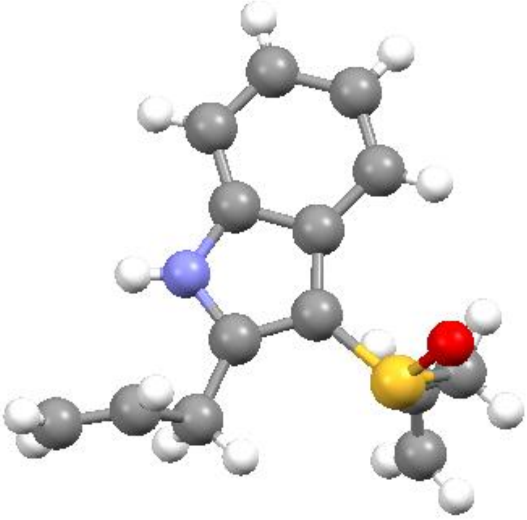
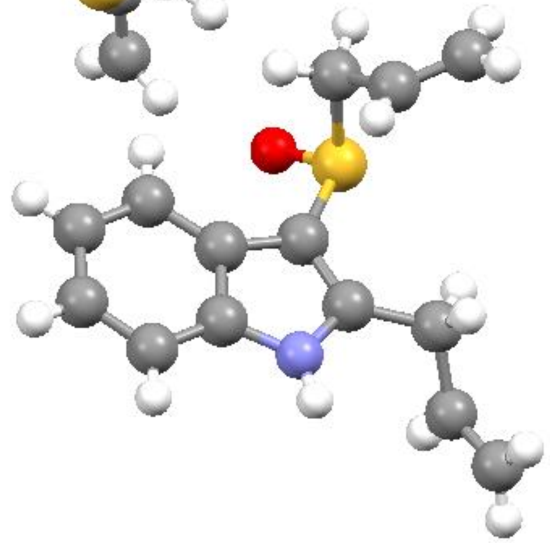

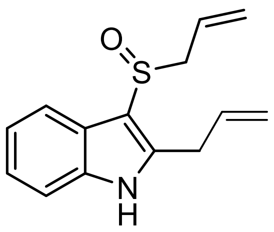

2d

CCDC 1570698

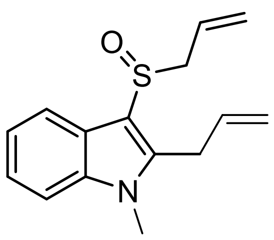

2e

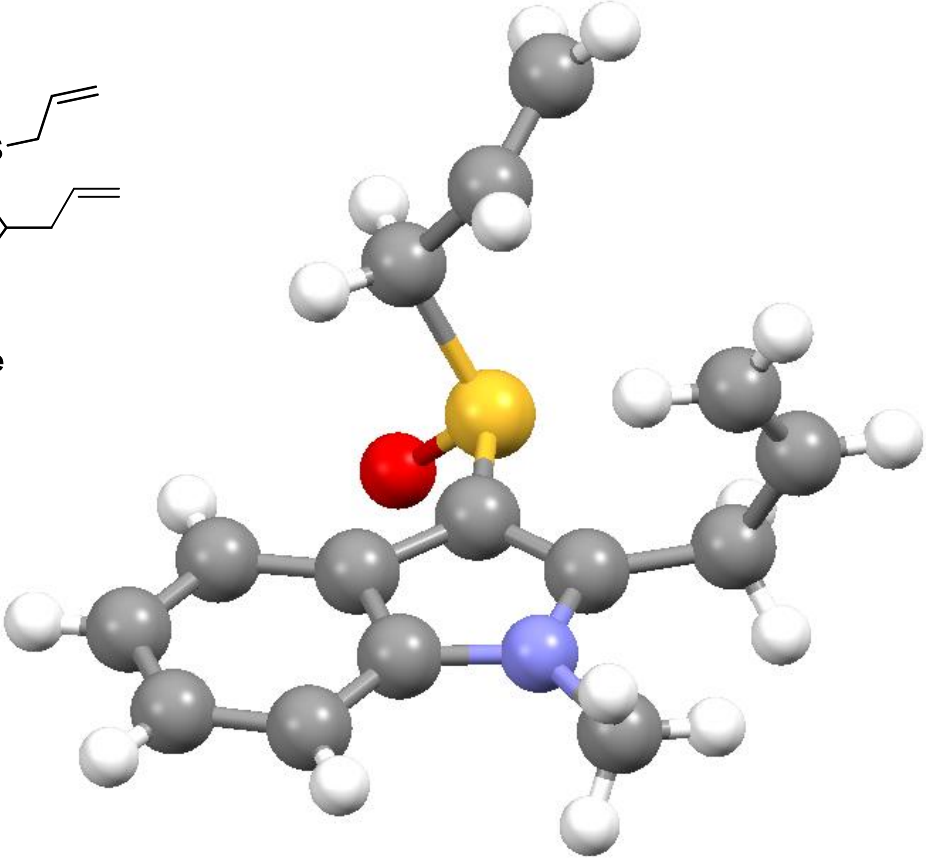

References

1 M. S. Morales-Rios, J. Espineira and P. Joseph-Nathan, *Magn. Reson. Chem.*, 1987, **25**, 377–395.

Full list of authors for references 4-6 in the main text:

4 a) T. M. Williams, T. M. Ciccarone, S. C. MacTough, C. S. Rooney, S. K. Balani, J. H. Condra, E. A. Emini, M. E. Goldman, W. J. Greenlee, L. R. Kauffman, J. A. O'Brien, S. V. V. Sardana, W. A. Schleif, A. D. Theoharides, P. S. Anderson, *J. Med. Chem*. 1993, **36**, 1291-1294; b) R. Silvestri, G. D. Martino, G. L. Regina, M. Artico, S. Massa, L. Vargiu, M. Mura, A. G. Loi, T. Marceddu, P. L. Colla, *J. Med. Chem.* 2003**, 46***,* 2482-2493; c) R. Ragno, M. Artico, G. D. Martino, G. L. Regina, A. Coluccia, A. D. Pasquali, R. Silvestri, *J. Med. Chem.* 2005**, 48***,* 213-223; d) R. Ragno, A. Coluccia, G. L. Regina, G. D. Martino, F. Piscitelli, A. Lavecchia, E. Novellino, A. Bergamini, C. Ciaprini, A. Sinistro, G. Maga, E. Crespan, M. Artico, R. Silvestri, *J. Med. Chem.* 2006**, 49***,* 3172-3184; e) Z. Zhao, S. E. Wolkenberg, M. Lu, V. Munshi, G. Moyer, M. Feng, A. V. Carella, L. T. Ecto, L. J. Gabryelski, M.-T. Lai, S. G. Prasad, Y. Yan, G. B. McGaughey, M. D. Miller, C. W. Lindsley, G. D. Hartman, J. P. Vacca, T. M. Williams, *Bio. Med. Chem. Lett.* 2008, **18**, 554-559; f) V. Famiglini, G. La Regina, A. Coluccia, S. Pelliccia, A. Brancale, G. Maga, W. Crespan, R. Badia, E. Riveira-Muñoz, J. A. Esté, R. Ferretti, R. Cirilli, C. Zamperini, M. Botta, D. Schols, V. Limongelli, B. Agostino, E. Novellino, R. Silvestri, *J. Med. Chem*. 2014, **57**, 9945–9957.

5 a) G. De Martino, M. C. Edler, G. La Regina, A. Coluccia, M. Chiara Barbera, D. Barrow, R. I. Nicholson, G. Chiosis, A. Brancale, E. Hamel, M. Artico, R. Silvestri, *J. Med. Chem.* 2006**, 49***,* 947-954; b) G. La Regina, R. Bai, W. Rensen, A. Coluccia, F. Piscitelli, V. Gatti, A. Bolognesi, A. Lavecchia, I. Granata, A. Porta, B. Maresca, A. Soriani, M. L. Iannitto, M. Mariani, A. Santoni, A. Brancale, C. Ferlini, G. Dondio, M. Varasi, C. Mercurio, E. Hamel, P. Lavia, E. Novellino, R. Silvestri, *J. Med. Chem*. 2011, **54**, 8394–8406; c) G. La Regina, R. Bai, W. M. Rensen, E. Di Cesare, A. Coluccia, F. Piscitelli, V. Famiglini, A. Reggio, M. Nalli, S. Pelliccia, E. Da Pozzo, B. Costa, I. Granata, A. Porta, B. Maresca, A. Soriani, M. L. Iannitto, A. Santoni, J. Li, M. M. Cona, F. Chen, Y. Ni, A. Brancale, G. Dondio, S. Vultaggio, M. Varasi, C. Mercurio, C. Martini, E. Hamel, P. Lavia, E. Novellino, R. Silvestri, *J. Med. Chem*. 2013, **56**, 123–149; d) Q. Guan, C. Han, D. Zuo, M. Zhai, Z. Li, Q. Zhang, Y. Zhai, X. Jiang, K. Bao, Y. Wu, W. Zhang, *Eur. J. Med. Chem*. 2014, **87**, 306-315; e) M. P. Fortes, P. B. N. da Silva, T. G. da Silva, T. S. Kaufman, G. C. G. Militão, C. C. Silveira, *Eur. J. Med. Chem*. 2016, **118**, 21-26.

6 a) M. Nuth, H. Guan, N. Zhukovskaya, Y. L. Saw, R. P. Ricciardi, *J. Med. Chem.* 2013, **56**, 3235–3246; b) L. M. Miller, W.-J. Keune, D. Castagna, L. C. Young, E. L. Duffy, F. Potjewyd, F. Salgado-Polo, P. E. García, D. Semaan, J. M. Pritchard, A. Perrakis, S. J. F. Macdonald, C. Jamieson, A. J. B. Watson, *J. Med. Chem*. 2017, **60**, 722–748; c) J. A. Campbell, V. Bordunov, C. A. Broka, M. F. Browner, J. M. Kress, T. Mirzadegan, C. Ramesha, B. F. Sanpablo, R. Stabler, P. Takahara, A. Villasenor, K. A. M. Walker, J.-H. Wang, M. Welch, P. Weller, *Bio. Med. Chem. Lett*. 2004, **14**, 4741-4745; d) Y. Lamotte, P. Martres, N. Faucher, A. Laroze, D. Grillot, N. Ancellin, Y. Saintillan, V. Beneton, R. T. Gampe Jr., *Bio. Med. Chem. Lett*. 2010, **20**, 1399-1404; e) S. Daly, K. Hayden, I. Malik, N. Porch, H. Tang, S. Rogelj, L. V. Frolova, K. Lepthien, A. Kornienko, I. V. Magedov, *Bio. Med. Chem. Lett*. 2011, **21**, 4720-4723; f) T. Luker, R. Bonnert, S. Brough, A. R. Cook, M. R. Dickinson, I. Dougall, C. Logan, R. T. Mohammed, S. Paine, H. J. Sanganee, C. Sargent, J. A. Schmidt, S. Teague, S. Thom, *Bio. Med. Chem. Lett*. 2011, **21**, 6288-6292; g) J. H. Hutchinson, D. Riendeau, C. Brideau, C. Chan, D. Delorme, D. Denis, J.-P. Falgueyret, R. Fortin, J. Guay, P. Hamel, T. R. Jones, D. Macdonald, C. S. McFarlane, H. Piechuta, J. Scheigetz, P. Tagari, M. Thgrien, Y. Girard, *J. Med. Chem*. 1993, **36**, 2771-2787.
